# Hepatitis B-related hepatocellular carcinoma: classification and prognostic model based on programmed cell death genes

**DOI:** 10.3389/fimmu.2024.1411161

**Published:** 2024-05-10

**Authors:** Jinyue Tian, Jiao Meng, Zhenkun Yang, Li Song, Xinyi Jiang, Jian Zou

**Affiliations:** Department of Clinical Laboratory, Wuxi People’s Hospital Affiliated Nanjing Medical University, Wuxi, China

**Keywords:** hepatocellular carcinoma, hepatitis B virus infection, programmed cell death, clinical characteristics, prognostic model

## Abstract

**Instruction:**

Hepatitis B virus (HBV) infection is a major risk factor for hepatocellular carcinoma (HCC). Programmed cell death (PCD) is a critical process in suppressing tumor growth, and alterations in PCD-related genes may contribute to the progression of HBV-HCC. This study aims to develop a prognostic model that incorporates genomic and clinical information based on PCD-related genes, providing novel insights into the molecular heterogeneity of HBV-HCC through bioinformatics analysis and experimental validation.

**Methods:**

In this study, we analyzed 139 HBV-HCC samples from The Cancer Genome Atlas (TCGA) and validated them with 30 samples from the Gene Expression Omnibus (GEO) database. Various bioinformatics tools, including differential expression analysis, gene set variation analysis, and machine learning algorithms were used for comprehensive analysis of RNA sequencing data from HBV-HCC patients. Furthermore, among the PCD-related genes, we ultimately chose *DLAT* for further research on tissue chips and patient cohorts. Besides, immunohistochemistry, qRT-PCR and Western blot analysis were conducted.

**Results:**

The cluster analysis identified three distinct subgroups of HBV-HCC patients. Among them, Cluster 2 demonstrated significant activation in DNA replication-related pathways and tumor-related processes. Analysis of copy number variations (CNVs) of PCD-related genes also revealed distinct patterns in the three subgroups, which may be associated with differences in pathway activation and survival outcomes. *DLAT* in tumor tissues of HBV-HCC patients is upregulated.

**Discussion:**

Based on the PCD-related genes, we developed a prognostic model that incorporates genomic and clinical information and provided novel insights into the molecular heterogeneity of HBV-HCC. In our study, we emphasized the significance of PCD-related genes, particularly *DLAT*, which was examined in vitro to explore its potential clinical implications.

## 1 Introduction

Chronic hepatitis B virus (HBV) infection is a major risk factor for the development of Hepatocellular carcinoma (HCC), particularly in regions with high HBV prevalence (1, 2). Despite advances in treatment, the prognosis of HBV-related HCC remains poor, with a high rate of recurrence and metastasis (3, 4). Therefore, there is an urgent need to identify novel prognostic biomarkers and therapeutic targets for HBV-related HCC.

Programmed cell death (PCD) is a critical process in the regulation of tissue homeostasis and the elimination of damaged or abnormal cells (5). Several types of PCD have been identified, including apoptosis, necroptosis, pyroptosis, and ferroptosis (6). Recently, several studies have suggested that PCD plays a critical role in the development and progression of HCC (7–9). However, the role of different types of PCD in HBV-related HCC and their clinical significance remains unclear.

In this study, we aimed to identify distinct subgroups of HBV-HCC patients based on clinical characteristics and expression profiles of PCD-related genes. Various bioinformatics tools, including differential expression analysis, gene set variation analysis, and machine learning algorithms were used for comprehensive analysis of RNA sequencing data from HBV-HCC patients. The identification of subgroups with distinct clinical characteristics, immune microenvironments, metabolic states, and drug sensitivities may facilitate the development of effective therapies for HBV-HCC. Furthermore, among the PCD-related genes, we ultimately chose *DLAT* for further research on tissue chips and patient cohorts. Upon analyzing the relationship between *DLAT* and patient survival prognosis, it was discovered that patients with deep *DLAT* staining had significantly shorter survival times than those with light *DLAT* staining. Through our study, we can classify HBV-HCC subgroups based on different PCD-related genes and construct prognostic models. It suggests that PCD-related genes can serve as potential biomarkers for patient stratification and personalized treatment.

## 2 Material and methods

### 2.1 NMF unsupervised clustering of HBV-HCC samples

We analyzed 139 HBV-HCC samples from TCGA using non-negative matrix factorization (NMF) to perform unsupervised clustering. The cophenetic value and clustering heatmap were used to determine the optimal number of clusters, and we found that three clusters showed the greatest inter-group variability and the least intra-group variability. We then compared the overall survival (OS) rates of the three clusters using the “survival” and “survminer” packages in R software. The expression of genes related to programmed cell death (PCD) in the three clusters was visualized using a heatmap generated with the “pheatmap” package. We also analyzed the clinical characteristics of the three clusters using a stacked bar plot generated with the “ggplot2” package.

### 2.2 Immune cell infiltration analysis and prediction of response to immunotherapy

Analysis of immune cell infiltration was performed using TIMER2.0 (http://timer.cistrome.org), which utilizes gene expression data to estimate the abundance of various immune cell types in tumor tissues. Seven different methods were used to evaluate immune cell infiltration. Heatmap visualization of differentially expressed immune cells was generated using the “pheatmap” package. The microenvironment of each cluster was evaluated using the “estimate” package, and boxplots were generated using the “ggpubr” package to compare the stromal score, immune score, and ESTIMATE score between clusters. The Tumor Immune Dysfunction and Exclusion (TIDE) algorithm (http://tide.dfci.harvard.edu) was used to predict the response to immune checkpoint blockade therapy, and the results were visualized using violin plots and boxplots with the “ggpubr” package.

### 2.3 GSVA and CNV analysis of PCD-related genes in HBV-HCC

Gene set variation analysis (GSVA) was performed using the “GSVA” and “GSEABase” packages in R to calculate the pathway activity of HALLMARK gene sets in the three HBV-HCC subgroups. Heatmaps were generated to visualize the pathway activity using the “pheatmap” package. The HALLMARK gene sets were obtained from the Molecular Signatures Database (MSigDB) (http://www.gsea-msigdb.org/gsea/msigdb). Additionally, Copy number variations (CNVs) of PCD-related genes in HBV-HCC subgroups were analyzed using CNV data obtained from the UCSC Xena browser. Lollipop charts were generated to visualize the CNV variations using the “ggplot2” package.

### 2.4 Differential gene expression analysis and enrichment analysis of HBV-HCC subgroups

RNA sequencing data from three subgroups of HBV-HCC were obtained and analyzed using R software. Differential expression analysis was performed using the “limma” package to identify differentially expressed genes (DEGs) between each subgroup. DEGs with an adjusted p-value < 0.05 and a log2 fold change > 1 were considered significant. Gene ontology (GO) and Kyoto Encyclopedia of Genes and Genomes (KEGG) enrichment analysis were performed on the DEGs for each subgroup using the “clusterProfiler” package in R. The enriched GO terms and KEGG pathways with a p-value < 0.05 were considered significant. To visualize the GO enrichment results, GO circle plots were generated using the “circlize” and “ComplexHeatmap” packages in R.

### 2.5 Identification of survival-associated genes and construction of a prognostic model

The univariate cox regression analysis was performed to identify genes significantly associated with OS with p-value<0.05. Two methods, LASSO regression and random survival forest (RSF), were used for further screening of survival-associated genes. The optimal lambda value was used to select genes in the LASSO regression, and the top 10 genes with the highest importance score based on the Gini coefficient were selected in the RSF analysis. The intersection of genes selected by the two methods was used for further analysis. A stepwise multiple cox regression was conducted to build a prognostic model using the selected genes, and hub genes were identified. Risk scores were calculated based on the expression levels and coefficients of the hub genes for distinguishing patients into high- and low-risk groups. Kaplan-Meier survival curves and receiver operating characteristic (ROC) curves were used to evaluate the performance of the model. Principal component analysis (PCA) and t-distributed stochastic neighbor embedding (t-SNE) were used to explore the expression pattern of the hub genes and to visualize the clustering of patients in the high- and low-risk groups.

### 2.6 Evaluation of prognostic value and gene expression patterns in high- and low-risk groups

We used the same risk score formula derived from the stepwise multivariate Cox regression analysis in the training and validation sets. Patients were ranked according to their risk scores, and the risk score distribution and survival curves were plotted to evaluate the prognostic value of the risk score. In addition, to compare the gene expression patterns between the high- and low-risk groups, we selected the top five genes from the multivariate Cox regression analysis and compared their expression levels in the two groups. The expression levels were presented as a heatmap using the “pheatmap” package.

### 2.7 Immune cell and immune process enrichment analysis and evaluation of immune therapy and MSI score in HBV-HCC

GSVA method was used to evaluate the relative enrichment score of 29 immune cell types and immune processes in HBV-HCC samples. The ssGSEA score of each immune cell type and immune process was calculated using the “GSVA” and “GSEABase” packages in R. The ImmunCellAI algorithm was used to evaluate the sensitivity of immune therapy for HBV-HCC patients. The immune cell score, immunotherapy exclusion score, and cytotoxic score were obtained through the ImmunCellAI web tool (http://bioinfo.life.hust.edu.cn/ImmuCellAI#!/analysis). The tumor immune dysfunction and exclusion (TIDE) algorithm was used to evaluate the MSI (Microsatellite instability, MSI) score of HBV-HCC samples. MSI is an important factor in the occurrence and development of tumors. The results were visualized using violin plots and boxplots with the “ggpubr” package.

### 2.8 Drug sensitivity analysis using IC50 data from the GDSC database

Drug IC50 data were obtained from the Genomics of Drug Sensitivity in Cancer (GDSC) database. Drug sensitivity analysis was conducted with “pRRophetic” and box plots were drawn by “ggplot2” in R software. IC50 values between the high- and low-risk groups were compared using the t-test. Drugs with significantly lower IC50 values in the low-risk group were considered potentially suitable for low-risk patients, while drugs with significantly lower IC50 values in the high-risk group were considered potentially suitable for high-risk patients.

### 2.9 Construction and evaluation of clinical prediction model for HBV-HCC patients

Patients with complete clinical information and survival data were included in this study. Univariate cox regression analysis was performed to extract factors with p<0.05 with “graphics” package and construct a multivariate cox regression model with “StepReg” and “regplot” packages. The ROC curve was evaluated to assess the discrimination ability of the model using “timeROC” package. The calibration curve was plotted with “timeROC” package to evaluate the calibration of the model. The clinical prediction model was divided into high and low-risk groups based on the model with “survival” package.

### 2.10 Western blot and qRT-PCR

Liver cancer cells were lysed using RIPA buffer (Cell Signal Technology, MA), centrifuged for the supernatant. The protein concentration was measured using bicinchoninic acid (BCA) assay (Cwbio, Beijing, China). The lysates were then diluted in loading buffer and denatured by heating at 100°C. Standard Western blot assay were performed using *DLAT* antibody (Proteintech, 68303) and GAPDH antibody (Abcam, ab77109) as the loading control.

Total RNA was extracted from the liver cancer cells using Trizol reagent (Invitrogen, USA) and cDNA was synthesized using the M-MLV Reverse Transcriptase Kit (Cwbio) following the manufacturer’s instructions. RT-PCR was performed using Real SYBR Mixture (Cwbio) on a Lightcycler 480 II instrument (Roche Applied Science, USA). GAPDH severed as the internal control.

*DLAT* forward primer: 5′-CCGCCGCTATTACAGTCTTCC-3′;
*DLAT* reverse primer: 5′-CTCTGCAATTAGGTCACCTTCAT-3′.
*GAPDH* forward primer: 5′-TGTTGCCATCAATGACCCCTT-3′;
*GAPDH* reverse primer: 5′-CTCCACGACGTACTCAGCG-3′

### 2.11 Tissue microarray and immunohistochemistry

Immunohistochemistry staining was performed using the streptavidin-peroxidase method according to the manufacturer’s instructions (Ultrasensitive; MaiXin, Fuzhou, China). The tissue microarray HLivH180Su09 which was related to HBV infection were incubated with an anti-*DLAT* antibody (mouse anti-human; dilution, 1:2000; HPA040786) at 4°C overnight, followed by the biotinylated anti-mouse IgG secondary antibody. The result of IHC were independently scored by two investigators who were blinded to the clinical data. The scores were obtained by evaluating the staining intensity and percentage of positive cells in representative areas. We used the following strategy to assess the results: intensity, 0 (no signal), 1 (weak), 2 (moderate), or 3 (high); percentage of cells, 0%-100%. We multiplied the scores of the staining intensity and percentage to obtain a final score (range 0–3). When the IHC score≥1.5, they had a high *DLAT* expression. When the IHC score<1.5, they were defined as low *DLAT* expression.

## 3 Results

### 3.1 Identification of three subgroups of HBV-HCC samples with distinct clinical characteristics and survival outcomes

**Figure 1:**
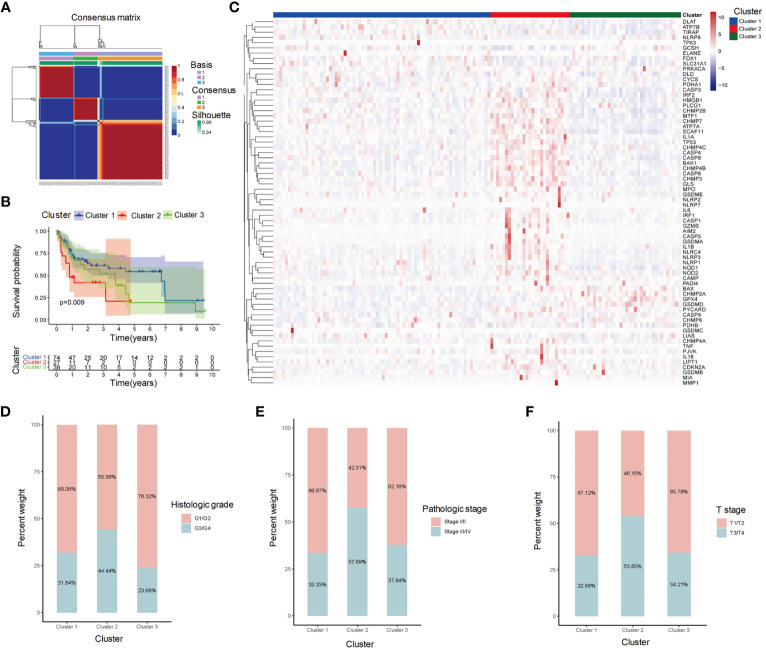
Clustering analysis of HBV-HCC samples based on gene expression profiles. **(A)** Clustering heatmap shows the identification of three distinct subgroups of HBV-HCC samples, labeled as Cluster 1, Cluster 2, and Cluster 3. **(B)** Kaplan-Meier survival analysis shows the overall survival (OS) of patients in each cluster. **(C)** Heatmap shows the expression levels of PCD-related genes in each cluster. **(D-F)** Stacked bar plot shows the distribution of histological grade, pathological stage, and T stage in each cluster. OS, Overall survival; PCD, Programmed cell death.

We collected 139 HBC-HCC samples from TCGA-LIHC which contained clinical information and survival outcomes. According to NMF unsupervised clustering (**Figure 1A**), the samples were divided into three subgroups, labeled Cluster 1, Cluster 2, and Cluster 3. Among them, Cluster 2 showed the worst Overall Survival (OS) probability (**Figure 1B**), and contained more overexpressed PCD-related genes in the heatmap (**Figure 1C**). Moreover, exploration of clinical characteristics revealed that Cluster 2 had the largest proportion of high-risk groups: more samples were at stage of G3/G4, III/IV and T3/T4 grade in histological grade, pathological stage, and T stage, respectively (**Figures 1D-F**).

### 3.2 Microenvironment and immunotherapy sensitivity evaluation

**Figure 2:**
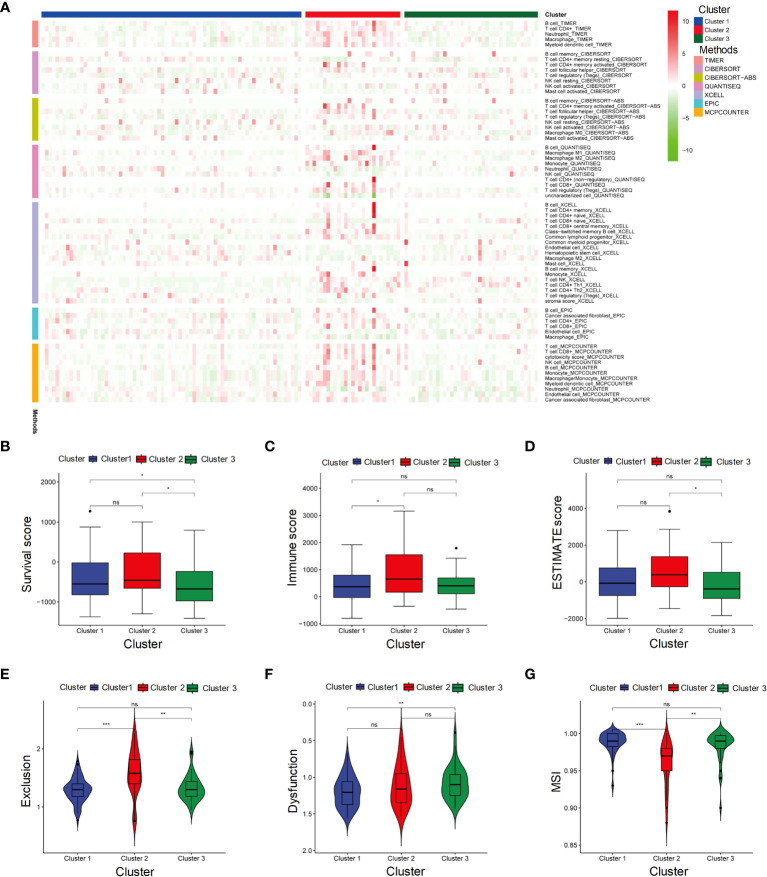
Microenvironment and immunotherapy sensitivity evaluation of three subgroups. **(A)** Heatmap represents immune cell infiltration in three subgroups of HBV-HCC using seven algorithms. **(B-G)** Stromal score, Immune score, ESTIMATE score, Exclusion score, Dysfunction score and MSI in Cluster 1, Cluster 2, and Cluster 3. MSI, Microsatellite instability. It signifies a lack of significant differences. *p≤0.05, **p≤0.01, ***p≤0.001. ns means p>0.05.

We perform 7 algorithms to evaluate the immune infiltration of three clusters (**Figure 2A**). Cluster 2 displayed higher abundance of immune cells compared to the other clusters. Consistent with the above results, Cluster 2 had the highest stromal score, immune score and estimate score (**Figures 2B-D**), indicating that more active microenvironments existed in Cluster 2. The TIDE evaluation revealed that the exclusion score of Cluster 2 was also the highest (**Figure 2E**). Although Cluster 2 contained more immune cells, the cells were undergoing immune rejection and were unable to infiltrate. In the evaluation of dysfunctions, Cluster 1 received the lowest score (**Figure 2F**). This indicates that Cluster 1 was supposed to have the least immune rejection and dysfunction, which could be associated with better survival outcomes. Meanwhile, both Cluster 1 and Cluster 3 had a higher MSI score compared to Cluster 2, suggesting that immunotherapy was least effective in Cluster 2 (**Figure 2G**).

### 3.3 Pathway and CNV analysis reveals differences among HBV-HCC subgroups

**Figure 3:**
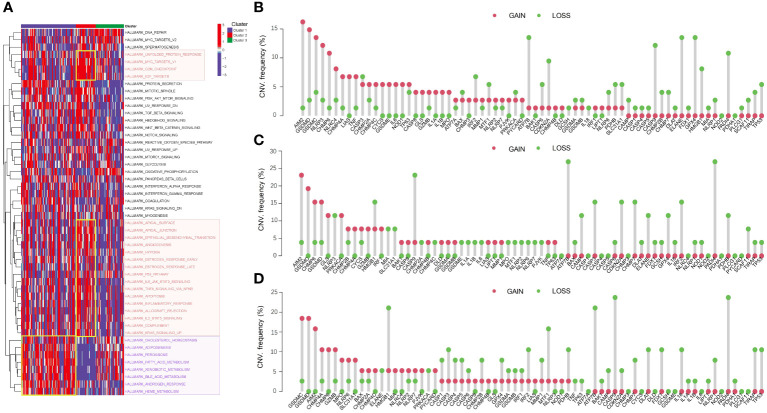
Pathway activation and CNV analysis reveal differences among HBV-HCC subgroups. **(A)** GSVA analysis shows the differences in pathway activity among the three HBV-HCC subgroups. The color red denotes DNA replication pathways, whereas purple signifies pathways related to metabolism. **(B-D)** Frequencies of CNV gain, loss, and non-CNV among PCD-related genes in the three HBV-HCC subgroups. CNV, Copy number variation; GSVA, Gene set variation analysis.

We further explored the pathway activation in three distinct clusters (**Figure 3A**). The heatmap revealed that metabolism-related pathways, such as fatty acid and bile acid metabolism, were significantly activated in Cluster 1. Cluster 2 exhibited activation of DNA replication pathways, including the G2M checkpoint, and tumor-related processes, such as p53 pathway, were significantly activated in Cluster 2, suggesting that Cluster 2 may have a closer relation to tumor progression. Analysis of CNVs in PCD-related genes revealed distinct patterns in the three HBV-HCC subgroups. In Cluster 1 (**Figure 3B**), 32 genes had more samples with amplifications in gene copy number compared to losses, while Cluster 2 and Cluster 3 had 22 and 24 genes, respectively (**Figures 3C, D**). These CNV variations may be correlated with the differences in pathway activation and survival outcomes among the subgroups.

### 3.4 Metabolic differences between HBV-HCC subgroups

**Figure 4:**
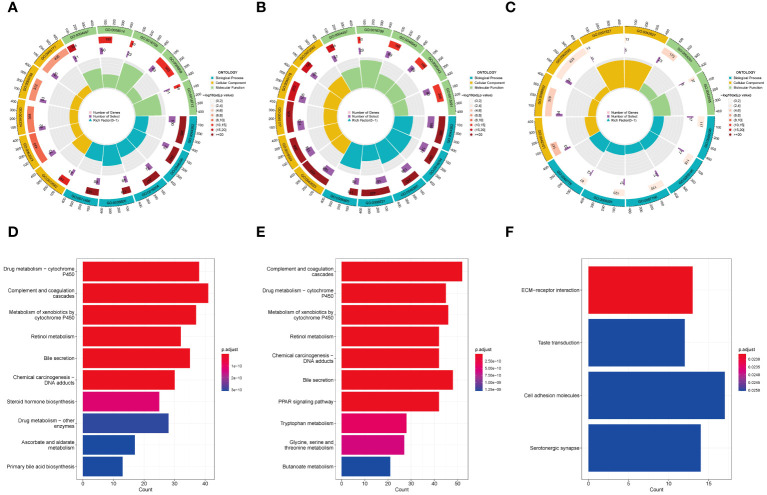
GO and KEGG analysis in HBV-HCC subgroups. GO enrichment analysis of Cluster 1 **(A)**, Cluster 2 **(B)**, and Cluster 3 **(C)**. KEGG pathway analysis of Cluster 1 **(D)**, Cluster 2 **(E)**, and Cluster 3 **(F)**. GO, Gene ontology; KEGG, Kyoto encyclopedia of genes and genomes.

We conducted GO and KEGG enrichment analyses on the differentially expressed genes within the three subgroups. The results of the GO enrichment analysis indicated that both Cluster 1 and Cluster 2 exhibited significant enrichment in GO terms related to metabolism (**Figures 4A-C**). However, the biological processes and signaling pathways related to metabolism were activated (GO z-scores > 0) in Cluster 1, while they were inhibited (GO z-scores < 0) in Cluster 2. KEGG pathway analysis showed that both Cluster 1 and Cluster 2 also shared significant enrichment in metabolic pathways (**Figures 4D-F**). However, the enriched pathways were mainly upregulated in Cluster 1, while they were downregulated in Cluster 2. These results suggest that the three subgroups have distinct metabolic states, with Cluster 1 showing activated metabolism, Cluster 2 showing inhibited metabolism, and Cluster 3 showing a different metabolic state.

### 3.5 Identification and validation of prognostic gene signature for HBV-HCC

**Figure 5:**
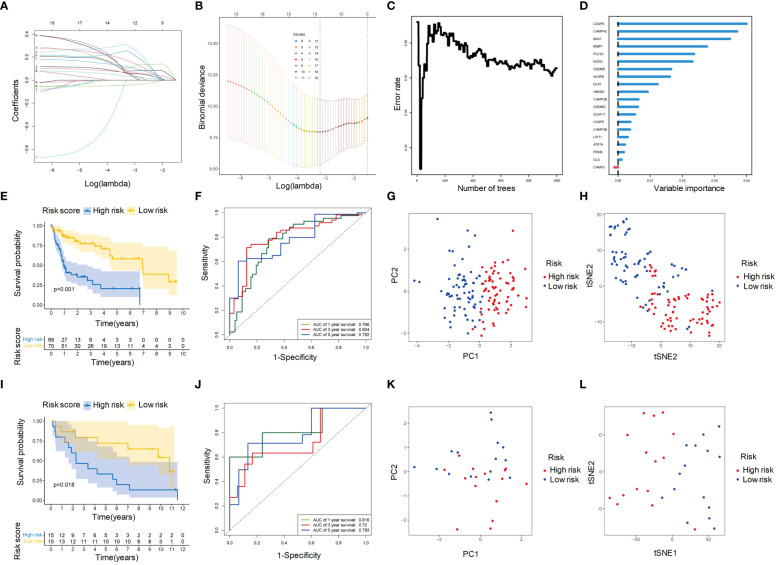
Identification of hub genes and construction of PCD-related prognostic model for HBV-HCC. **(A)** Univariate Cox regression analysis profiles 20 genes significantly associated with OS. **(B)** LASSO regression showed that when the error of the model was minimized, 12 variables were selected for further logistic regression analysis. **(C)** Variable importance plot for the top 10 genes identified by RSF analysis. **(D)** Classification error rates of the RSF analysis for different numbers of genes. **(E)** Kaplan-Meier survival curves for patients in the high- and low-risk groups defined by the five-gene prognostic model. **(F)** ROC curve analysis of the five-gene prognostic model for 1-year, 3-year and 5-year OS. **(G)** PCA analysis of the high- and low-risk groups based on the five-gene prognostic model. **(H)** tSNE analysis of the high- and low-risk groups based on the five-gene prognostic model. **(I-L)** The prognostic value of the five-gene signature was validated in an independent cohort. The Kaplan-Meier survival curves **(I)**, ROC curve analysis **(J)**, PCA analysis **(K)**, and tSNE analysis **(L)** showed consistent results with those of the training cohort. OS, Overall survival; LASSO, Least absolute shrinkage and selection operator; RSF, Random survival forest; ROC, Receiver operating characteristic curve; PCA, Principal components analysis; tSNE, t-distributed stochastic neighbor embedding.

We screened 20 PCD-related genes associated with OS using univariate Cox regression analysis. Two screening methods were used to identify potential genes: (1) According to LASSO regression, we selected 12 genes with the optimal lambda value (**Figures 5A, B**); (2) RSF analysis ranked the genes based on their importance, and we selected the top 10 genes (**Figure 5C**). The intersection of these two methods resulted in nine genes, which were further analyzed using multivariable Cox regression analysis. From this analysis, five genes (*CHMP4C, DLAT, MMP1, NLRP6, and NOD2*), were found to be associated with OS (**Figure 5D**). The risk score was calculated based on these five genes, and patients were divided into high-risk and low-risk groups. The KM survival curves showed that the high-risk group had significantly poorer OS than the low-risk group (p < 0.05) (**Figure 5E**). The ROC curves showed that the risk score had good accuracy in predicting 1-year (AUC: 0.766), 3-year (AUC: 0.804) and 5-year (AUC: 0.782) survival (**Figure 5F**). Additionally, PCA and tSNE analyses showed that the high-risk and low-risk groups were well separated based on their risk scores, indicating that the risk score represented the major differences in the patient samples (**Figures 5G, H**). These findings were consistent with those in the independent validation cohort (**Figures 5I-L**).

### 3.6 Identification and validation of prognostic gene signature for HBV-HCC

**Figure 6:**
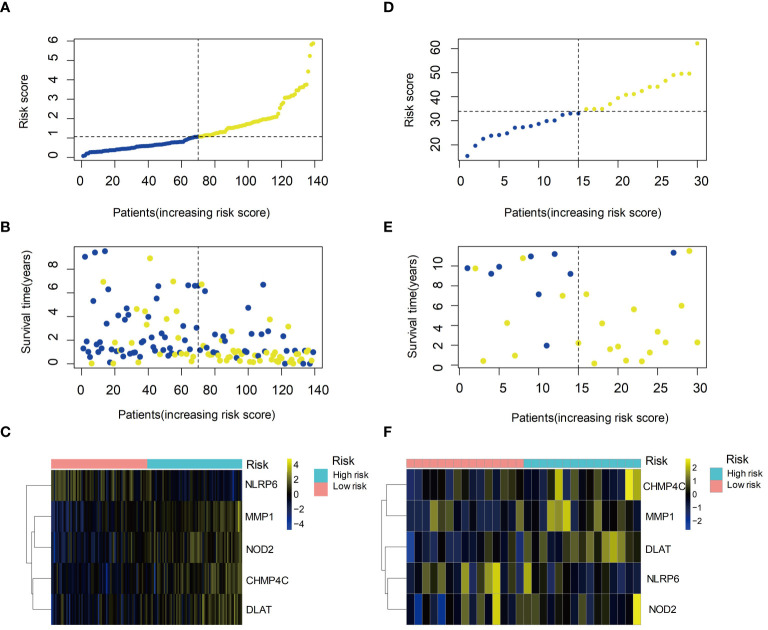
Prognostic value of the risk score and expression of hub genes in HBV-HCC. **(A)** Kaplan-Meier survival curves of the high- and low-risk groups based on the risk score. **(B)** Time-dependent ROC curves of the risk score for predicting survival outcomes. **(C)** Heatmap showing the expression levels of the five hub genes in the high- and low-risk groups. **(D)** Kaplan-Meier survival curves of the high- and low-risk groups in the validation set. **(E)** Time-dependent ROC curves of the risk score in the validation set. **(F)** Heatmap showing the expression levels of the five hub genes in the high- and low-risk groups in the validation set. ROC, Receiver operating characteristic curve.

We ranked the patients according to the risk score of the training set, and found that patients with higher risk scores had significantly worse survival outcomes, indicating that the risk score was a reliable prognostic indicator (**Figures 6A, B**). Compared the expression levels of the five selected genes between the high-risk and low-risk groups, we found NLRP6 exhibited higher expression levels in the low-risk group while the other four genes expressed at higher levels in the high-risk group (**Figure 6C**). The same results were observed in the validation set (**Figures 6D-F**). These findings confirmed the prognostic value of the risk score and the potential clinical significance of the selected genes in HBV-HCC.

### 3.7 Immune cell infiltration and microenvironment in HBV-HCC

**Figure 7:**
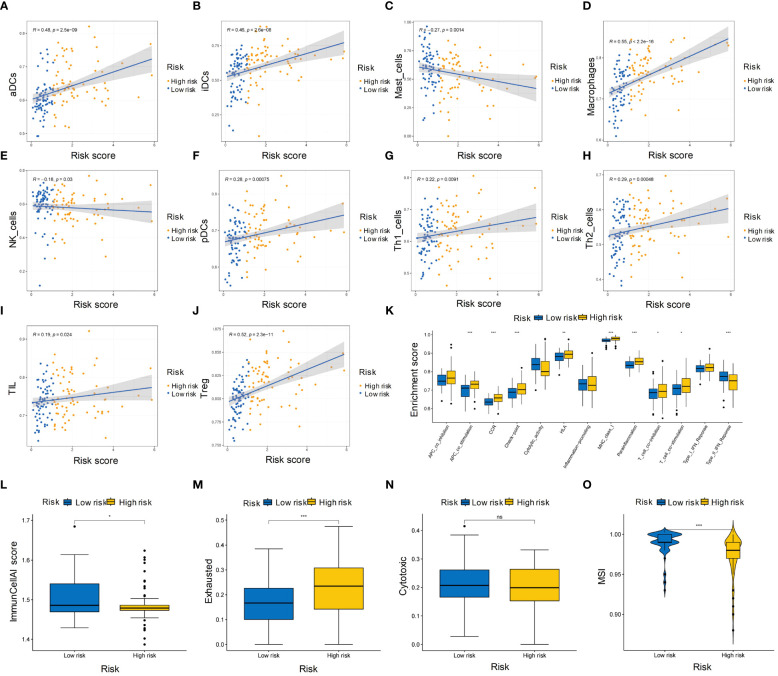
Immune cell infiltration and microenvironment in high- and low-risk groups of HBV-HCC patients. **(A-J)** Heatmap showing the ssGSEA scores of immune cell types in the high- and low-risk groups. **(K)** Boxplot showing the distribution of ssGSEA scores of immune processes in the high-risk and low-risk groups. **(L-N)** ImmunCellAI scores of the high-risk and low-risk groups for immunotherapy sensitivity, immunotherapy exclusion, and cytotoxic activity, respectively. **(O)** Boxplot showing the MSI scores of the high-risk and low-risk groups. ssGSEA, single sample gene set enrichment analysis; MSI, Microsatellite instability. *p≤0.05, **p≤0.01, ***p≤0.001. ns means p>0.05.

We evaluated the immune infiltration with ssGSEA analysis and discovered eight immune cell types were positively correlated with risk score, while two immune cell types were negatively correlated with the risk score (**Figures 7A-J**). In the high-risk group, most immune processes were significantly activated (**Figure 7K**), indicating a higher abundance of immune cells in this group. While low-risk group showed higher ImmunCellAI score, higher MSI score and lower exclusion score (**Figures 7L-O**) compared to high-risk group, indicating that the low-risk group was more likely to benefit from immunotherapy.

### 3.8 Drug sensitivity analysis reveals potential therapeutic options for high-risk and low-risk HCC patients

The IC50 values of 12 drugs were collected from GDSC. Among them, the IC50 of four drugs in the low-risk group was significantly lower than that in the high-risk group, indicating that these drugs may be more suitable for low-risk patients. On the other hand, the IC50 of 8 drugs was lower in the high-risk group, making them more suitable for high-risk patients (**Supplementary Figure S1**).

### 3.9 Development of a clinical prediction model based on T stage and risk score for HBV-HCC patients

**Figure 8:**
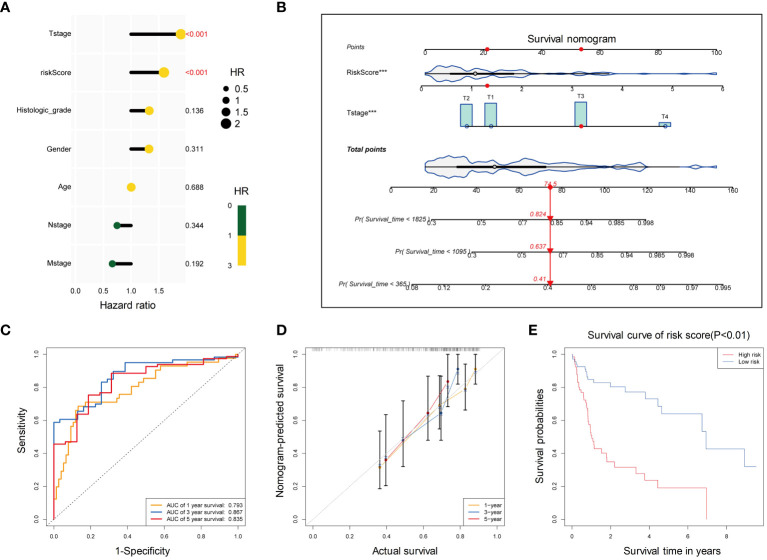
Development of a clinical prediction model based on T stage and risk score for HBV-HCC patients. **(A)** Univariate cox regression analysis of T stage and risk score for OS. **(B)** The distribution of risk scores in the training set. The dotted line represents the cut-off point for dividing patients into high- and low-risk groups. **(C)** The ROC curve of the multivariate Cox regression model based on T stage and risk score. **(D)** Calibration curves for 1-year, 3-year, and 5-year OS of HBV-HCC patients in the training cohort for the multivariate Cox regression model. **(E)** Kaplan-Meier curves for OS of patients in the high-risk and low-risk groups. OS, Overall survival; ROC, Receiver operating characteristic curve.

Combined with the risk score and clinical information (age, sex, T stage, N stage, M stage, and histological stage), univariate Cox regression analysis was performed yielding two factors T stage and risk score (p<0.05). These factors were closely associated with poor survival (**Figure 8A**). They were further used to construct a multivariate Cox regression model, visualized with a survival nomogram (**Figure 8B**). The ROC curve was drawn to assess the discrimination ability of the model (**Figure 8C**), with a larger AUC indicating better discrimination. The calibration curve was plotted to evaluate the calibration of the model, and the deviation between the actual curve and the ideal curve was small (**Figure 8D**). The clinical prediction model was further divided into high-risk and low-risk groups based on the model and significant survival differences were observed between the two groups (**Figure 8E**). Based on the risk score calculated by the 5 PCD-related genes and T stage of HBV-HCC tumors, we constructed a clinical model with good discrimination ability, calibration, and survival prediction.

### 3.9 Upregulation of *DLAT* in tumor tissues of HBV-HCC patients

**Figure 9:**
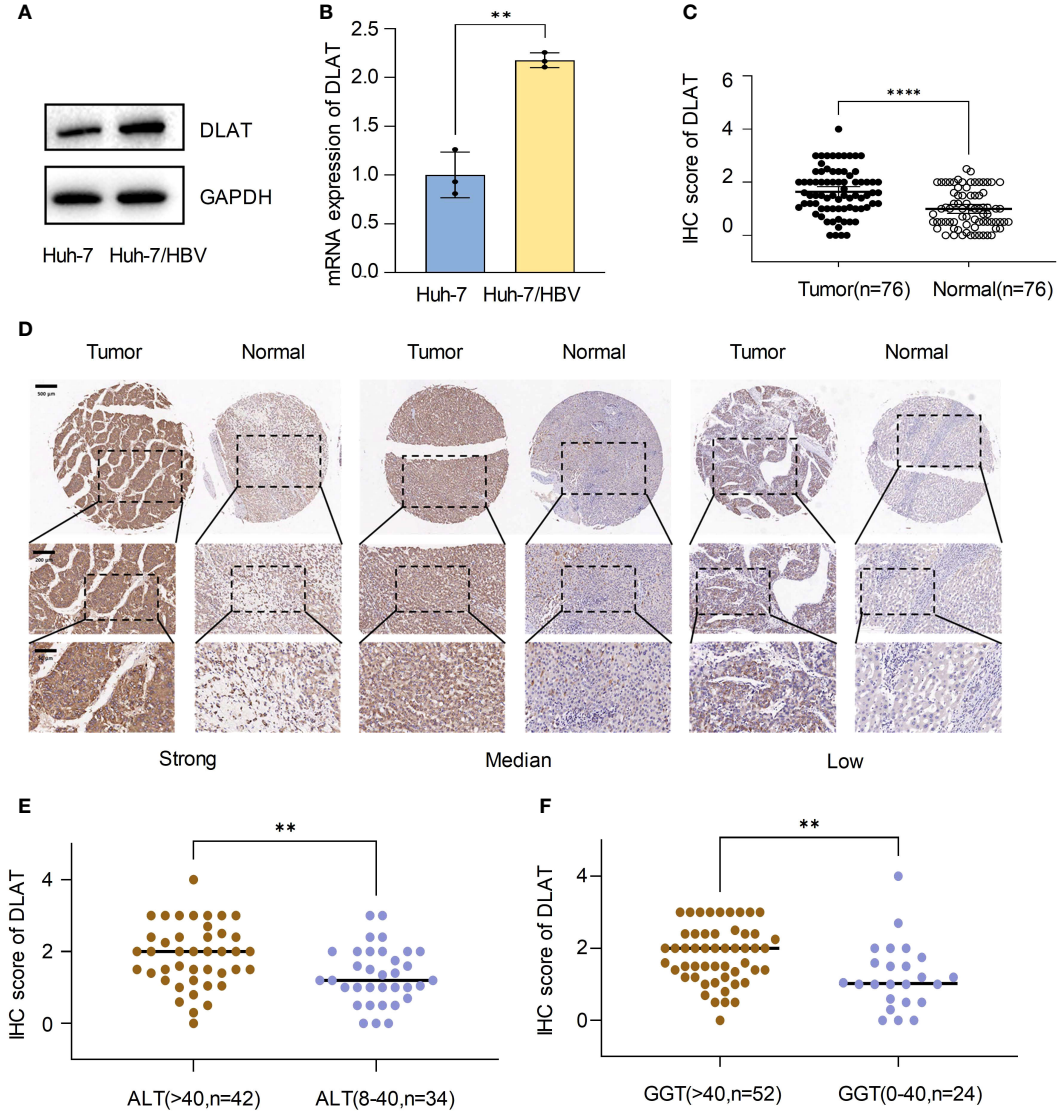
Upregulation of *DLAT* in tumor tissues of HBV-HCC patients. **(A, B)** Verify the expression of *DLAT* protein and mRNA levels in cells through *in vitro* experiments. **(C, D)** IHC staining of a tissue microarray was used to verify the expression of *DLAT* in HBV-HCC patients. **(E, F)** The relationship between *DLAT* IHC scores and levels of ALT and GGT. ** means p≤0.01, *** means p≤0.001, they all indicate significant differences.

We compared the expression of *DLAT* at both the mRNA and protein levels in Huh-7, and Huh-7 cells transfected with a plasmid containing the whole HBV genome (Huh-7/HBV). The results showed that *DLAT* levels increased after HBV transfection, both in terms of RNA and protein levels. (**Figures 9A, B**). Immunohistochemical staining was conducted on a tissue microarray of 76 HBV-HCC patients’ tumors and adjacent tissues. Three fields of view with high, medium, and low staining were chosen, revealing that the tumor exhibited stronger staining compared to the adjacent tissues. The immunohistochemical staining score of *DLAT* in cancer tissue was also significantly higher than in the adjacent tissues (p<0.001) (**Figures 9C, D**). Further analysis of the relationship between *DLAT* and the clinical characteristics of patients revealed that *DLAT* was associated with abnormal ALT and GGT levels (**Figures 9E, F**). It was speculated that *DLAT* is a gene associated with adverse effects in patients with HBV-HCC.

## 4 Discussion

HBV-related HCC is a complex and heterogeneous disease with pessimistic clinical outcomes (10–13). In this study, we focused on the role of three types of PCDs (cuproptosis, netotic cell death, and pyroptosis) and investigated their values in the progression and prognosis of HBV-HCC.

Several previous studies have identified subtypes in HCC (14, 15), and classified HCC patients with distinct clinical outcomes. Our study differed from those previous ones in the methods used to identify subtypes, and found specific clinical characteristics and immune features of each subtype. Through unsupervised clustering analysis, we firstly discovered three distinct subgroups of HBV-HCC patients with different clinical characteristics and survival outcomes. Cluster 2 was associated with the worst OS, and it had the highest abundance of immune cells, suggesting a more active microenvironment. However, TIDE analysis showed that Cluster 2 had significantly higher exclusion scores, indicating an immunosuppressive state and an inability for immune cells to infiltrate into the tumors, which may be related to its poor survival outcomes. MSI analysis also indicated that Cluster 2 was the least likely to benefit from immune checkpoint blockade therapy, while both Cluster 1 and Cluster 3 had higher MSI scores, suggesting that these two subgroups may be more sensitive to immunotherapy.

Previous studies have reported that dysregulated metabolism is a hallmark of cancer, especially in HCC (16–18), for example, glycolytic pathway (9) and lipid metabolism pathway (19) were found to be upregulated in HCC, and targeting these pathways may have therapeutic potential. In our study, we performed more detailed research and found that metabolic pathways were activated or inhibited in different immune subtypes of HBV-HCC. Small molecules, carboxylic acid, organic acid and other catabolic process related pathways were upregulated in Cluster 1 and downregulated in Cluster 2. The subgroups with distinct characteristics and activated pathways found in our study may bring implications for the development of personalized therapies for HBV-HCC patients.

Using a combination of univariate Cox regression analysis, LASSO regression, and random forest analysis, we identified five genes (*CHMP4C*, *DLAT*, *MMP1*, *NLRP6*, and *NOD2*) associated with OS. Among them, *MMP1* is a biomarker related to netotic cell death, *NOD2* and *NLRP6* associates with both autophagy and pyroptosis. Furthermore, *CHMP4C* and *DLAT* are related to pyroptosis and cuproptosis, respectively. We then developed a risk score formula based on their expression levels. The risk score had good predictive accuracy in differentiating high-risk and low-risk patients, and patients in the high-risk group had significantly poorer OS than those in the low-risk group. Furthermore, we evaluated the potential for drug sensitivity analysis based on the risk score. We found that four drugs had significantly lower IC50 values in the low-risk group, indicating that these drugs may be more effective in low-risk patients, while eight drugs had significantly lower IC50 values in the high-risk group. These genes may serve as potential prognostic biomarkers and therapeutic targets for HBV-HCC. As part of the pyruvate dehydrogenase complex, *DLAT* plays an important role in glucose metabolism and the TCA cycle. However, the relevance and function of *DLAT* in cancers such as HCC, are unclear (20, 21). It has been found that *DLAT* is a gene related to cuproptosis and glucose metabolism (22, 23). Therefore, *DLAT* was selected for further research.

Our study has several limitations. First, the sample size is relatively small, and external validation with a larger sample size is needed to confirm our findings. Second, the molecular mechanisms underlying the identified pathways and PCD-related genes need further investigation. Genes from different types of PCD that influence HBV-HCC progression independently or synergistically remains to be explored. Third, our study is based on transcriptomic data of HBV-HCC liver tissues, and further validation of the prediction model using other omics data from different HBV-HCC samples (such as blood samples, urine specimen and stool samples with better access) is warranted. Fourth, we selected *DLAT in vitro* experiments to verify its correlation with the poor prognosis of HBV-HCC. Subsequent functional experiments are needed to further explore how upstream HBV regulates *DLAT* and the effect of the increase in downstream *DLAT* on cuproptosis and metabolism.

In conclusion, our study identified distinct subgroups of HBV-HCC patients with different clinical characteristics, survival outcomes, and metabolic states, providing new insights into the heterogeneity of HBV-HCC. A prognostic model based on five PCD-related genes (specifically *DLAT*) and tumor stage that may serve as potential biomarkers for patient stratification and personalized therapy. Finally, our study highlights the potential for drug sensitivity analysis based on the risk score, which may facilitate the development of targeted therapies for HBV-HCC.

## Data availability statement

The datasets presented in this study can be found in online repositories. The names of the repository/repositories and accession number(s) can be found in the article/**Supplementary Material**.

## Author contributions

JT: Data curation, Writing – original draft. JM: Data curation, Methodology, Writing – original draft. ZY: Formal analysis, Methodology, Writing – original draft. LS: Formal analysis, Investigation, Writing – original draft. XJ: Project administration, Software, Supervision, Writing – original draft, Writing – review & editing. JZ: Conceptualization, Project administration, Supervision, Writing – review & editing.
