# Supplementary figures and images for: Hepatitis B-related hepatocellular carcinoma: classification and prognostic model based on programmed cell death genes (part 1 of 2)
Source: Front Immunol. 2024 May 10;15:1411161. doi: 10.3389/fimmu.2024.1411161 (PMC11116790; doi:10.3389/fimmu.2024.1411161)

# NMF rank survey

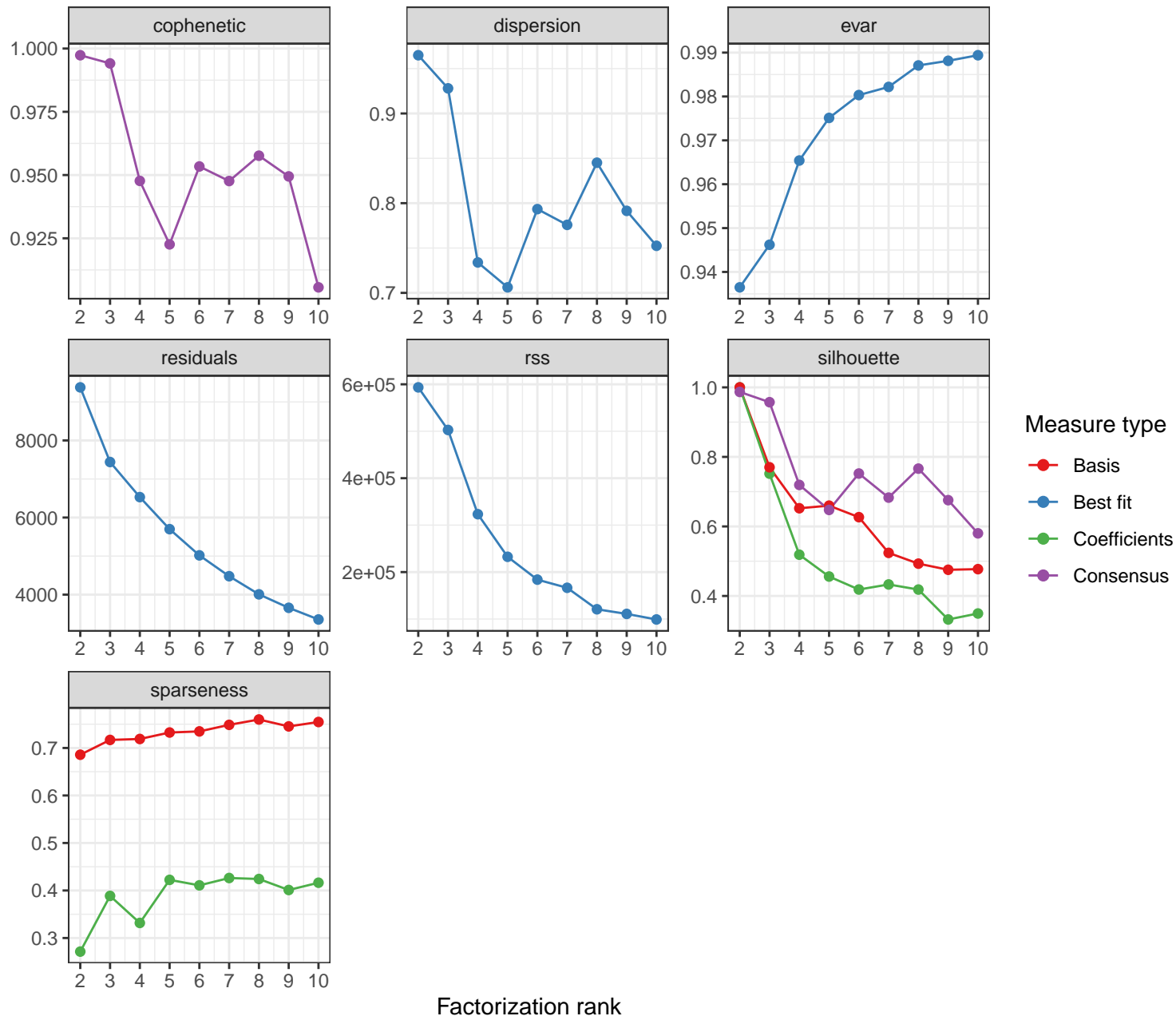

Supplement: Supplementary file 1 [file DataSheet_1.zip › original data 1-3/1-cluster/cophenetic.pdf]

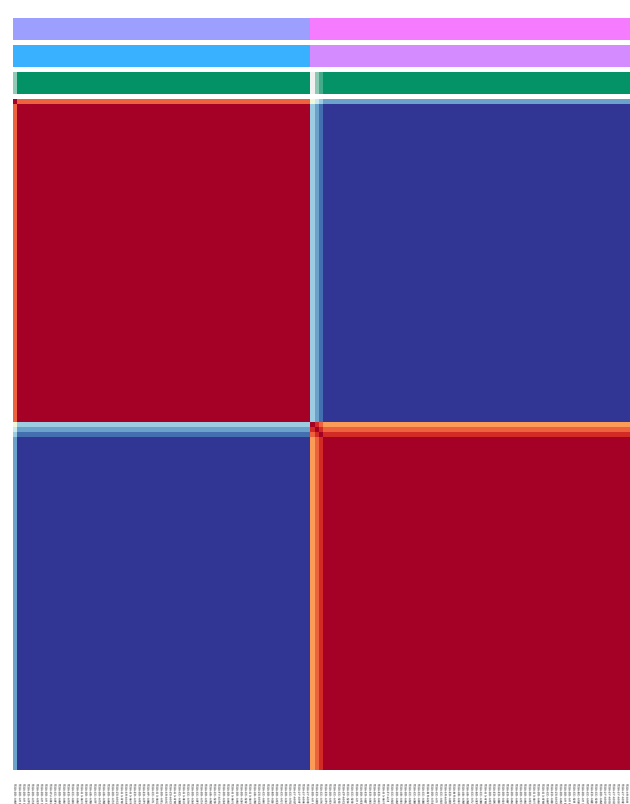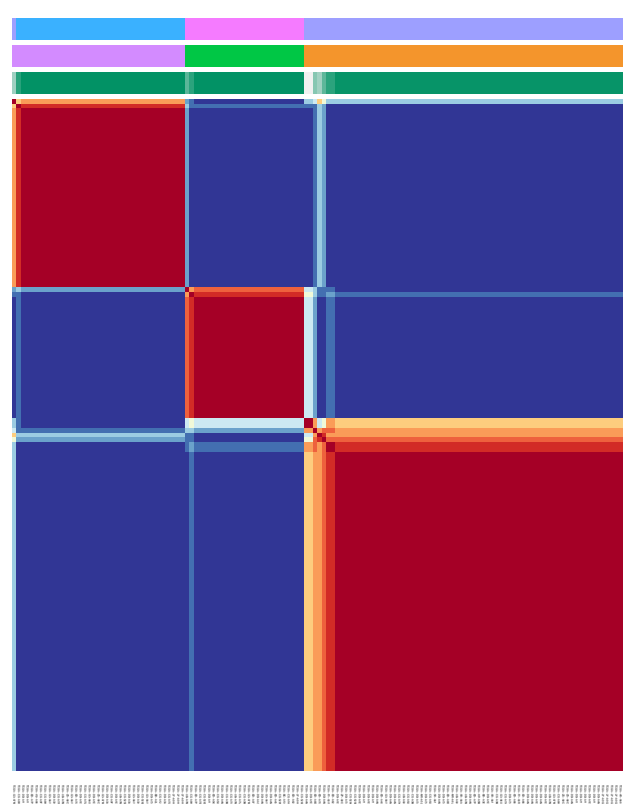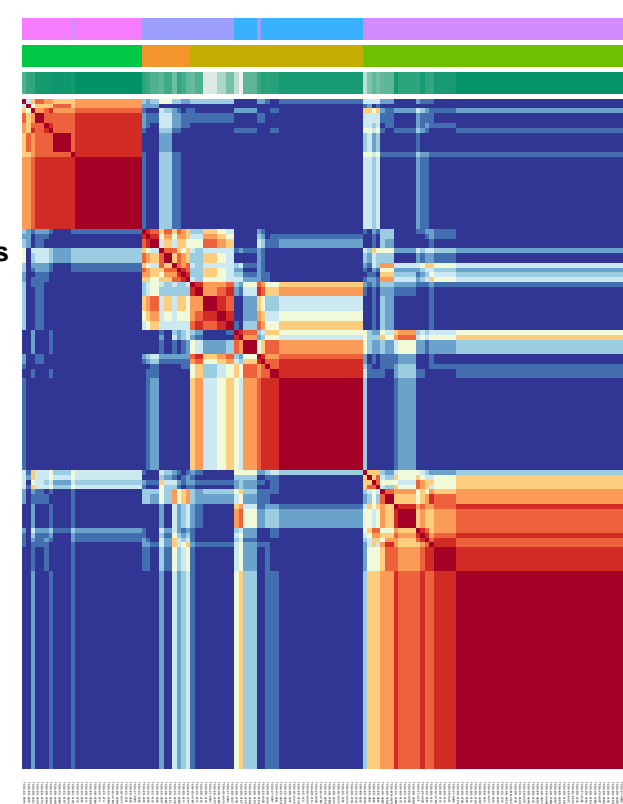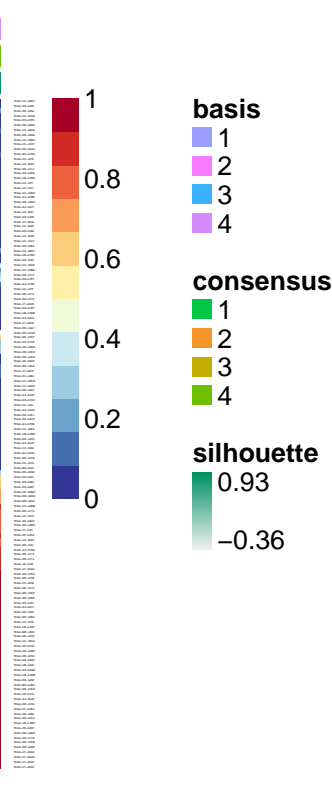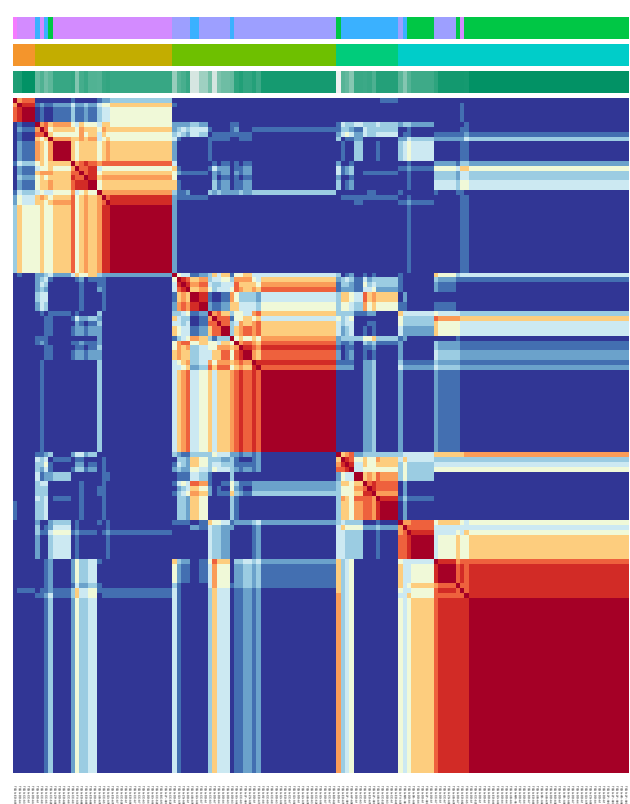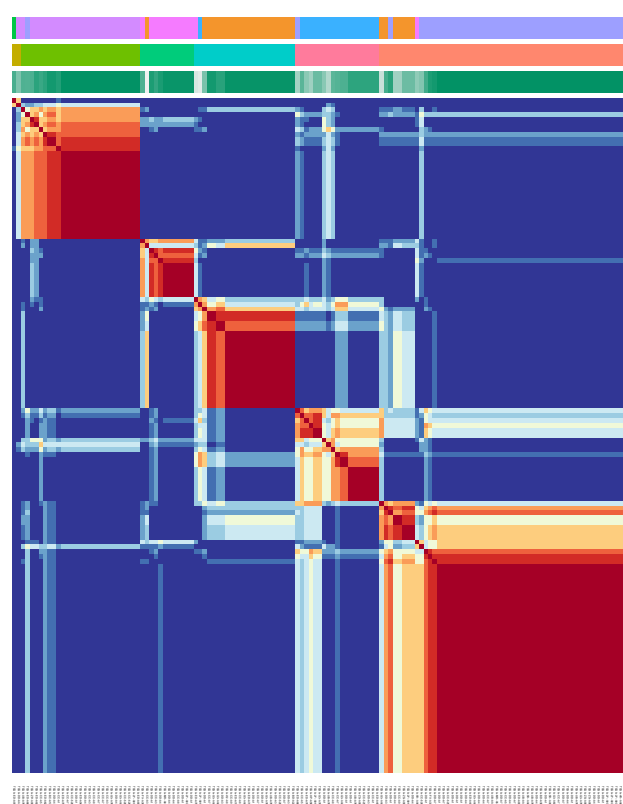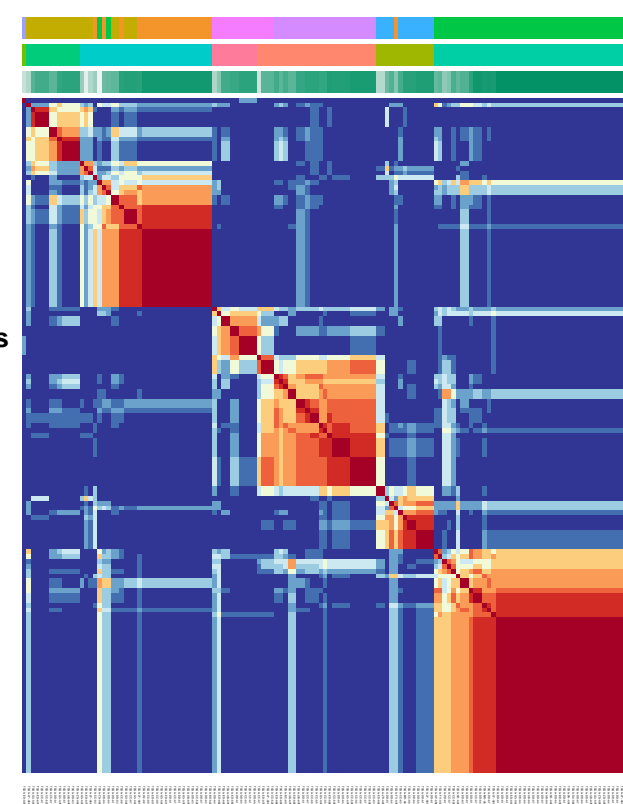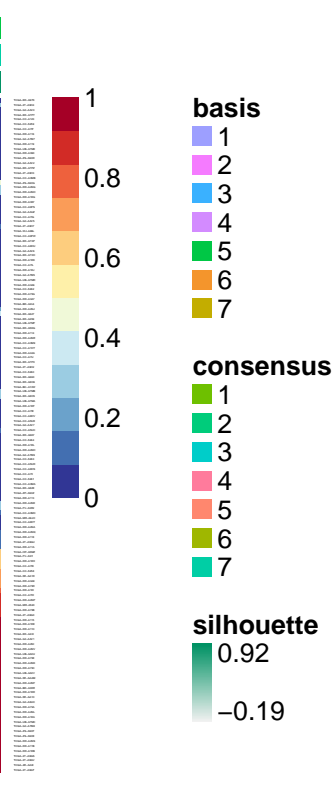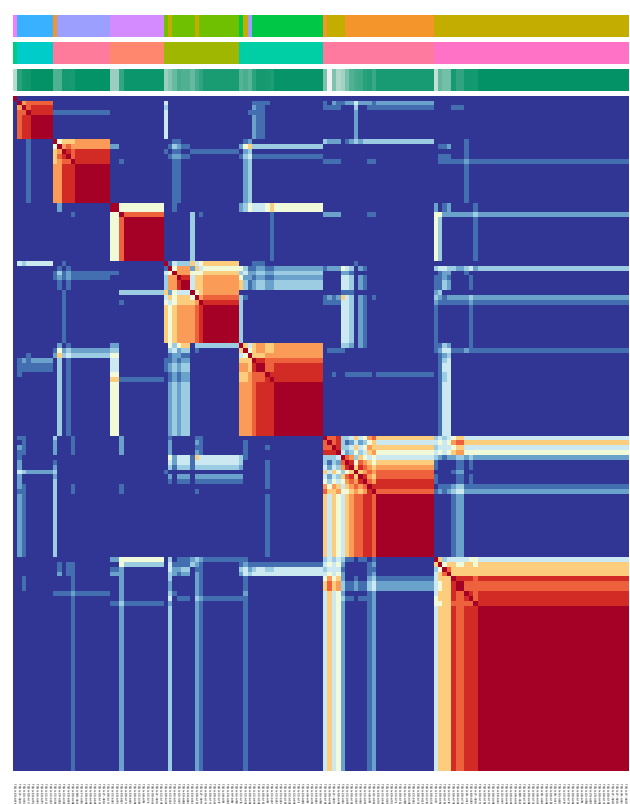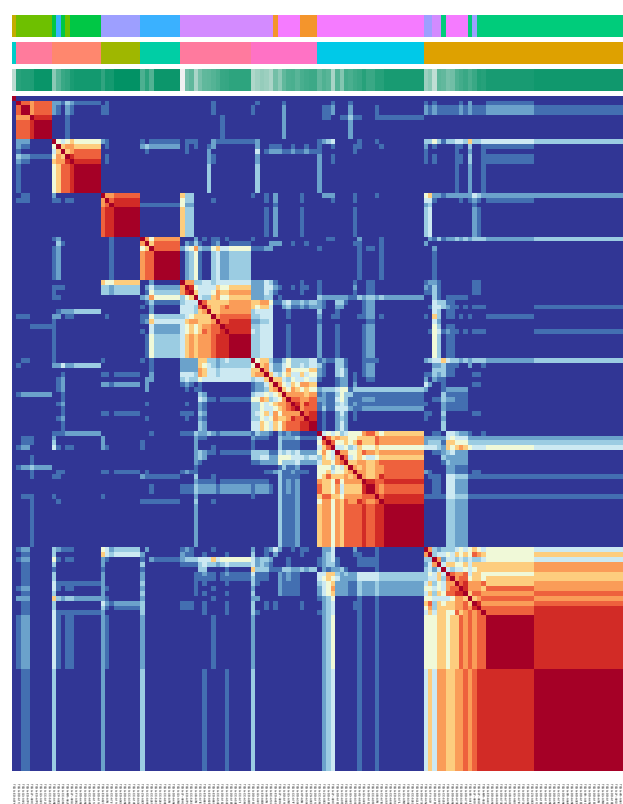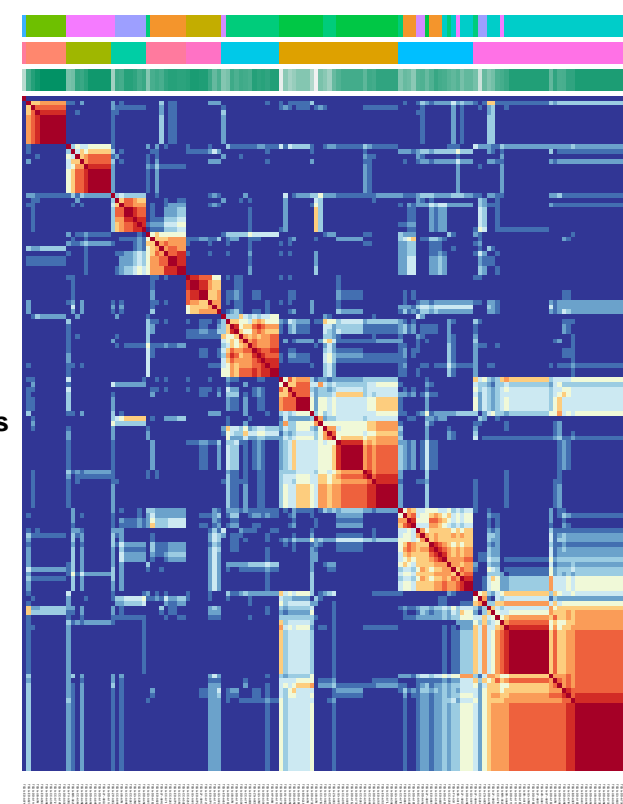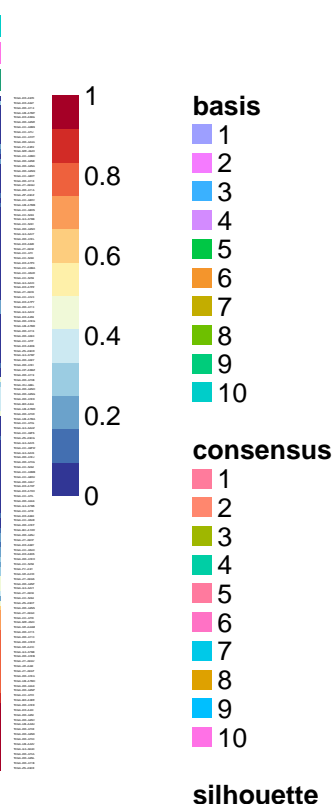

Supplement: Supplementary file 1 [file DataSheet_1.zip › original data 1-3/1-cluster/heatmap.all.pdf]

# Consensus matrix

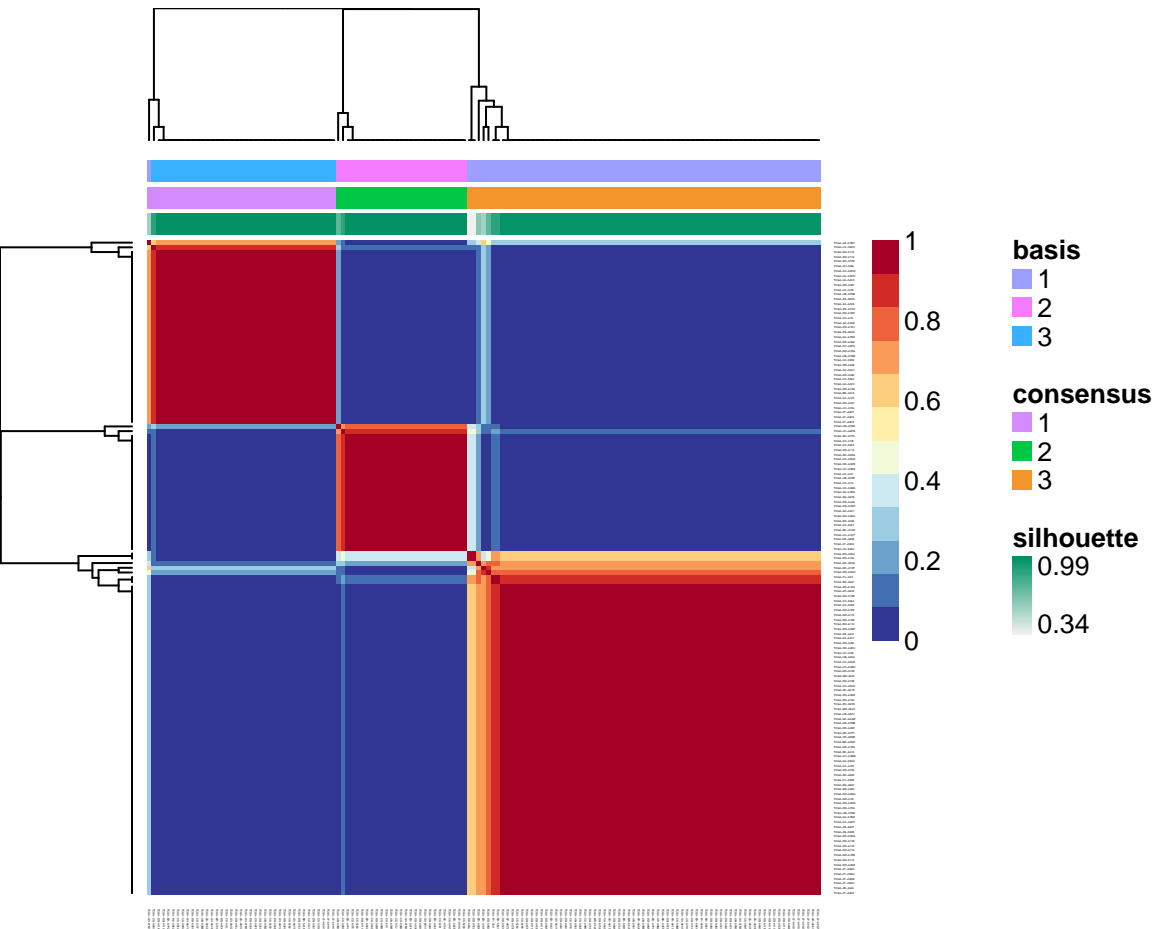

Supplement: Supplementary file 1 [file DataSheet_1.zip › original data 1-3/1-cluster/heatmap-3.pdf]

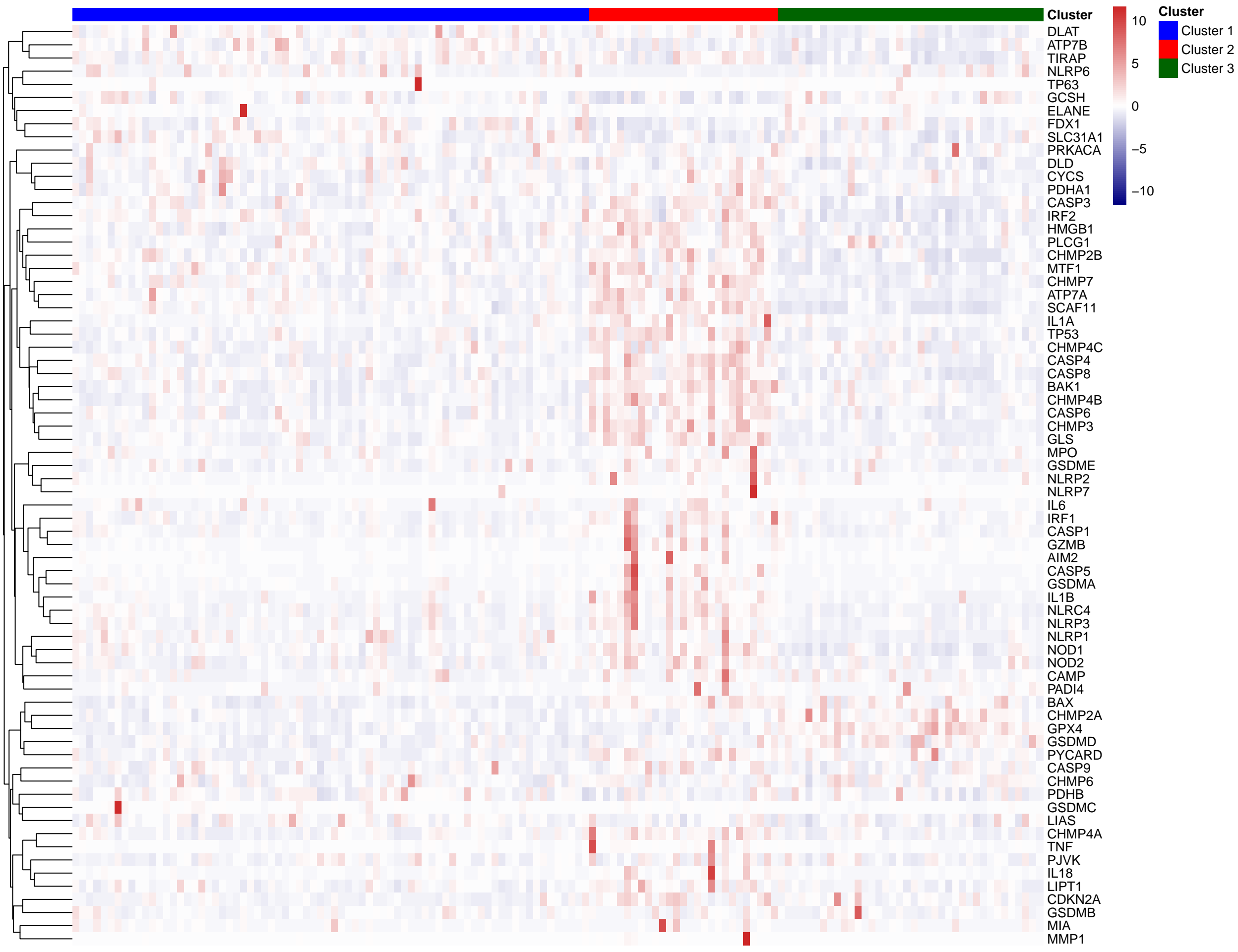

Supplement: Supplementary file 1 [file DataSheet_1.zip › original data 1-3/1-cluster/Heatmap-gene.pdf]

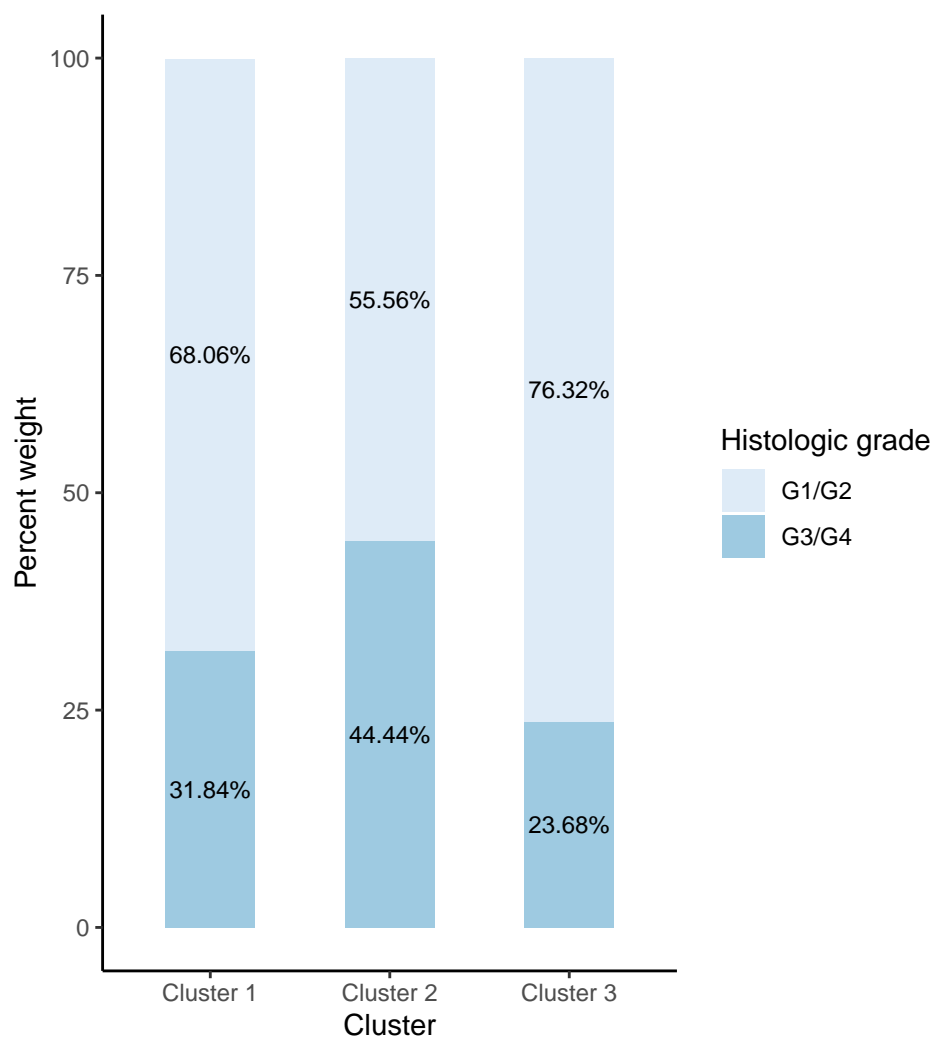

Supplement: Supplementary file 1 [file DataSheet_1.zip › original data 1-3/1-cluster/Histologic_grade-barplot.pdf]

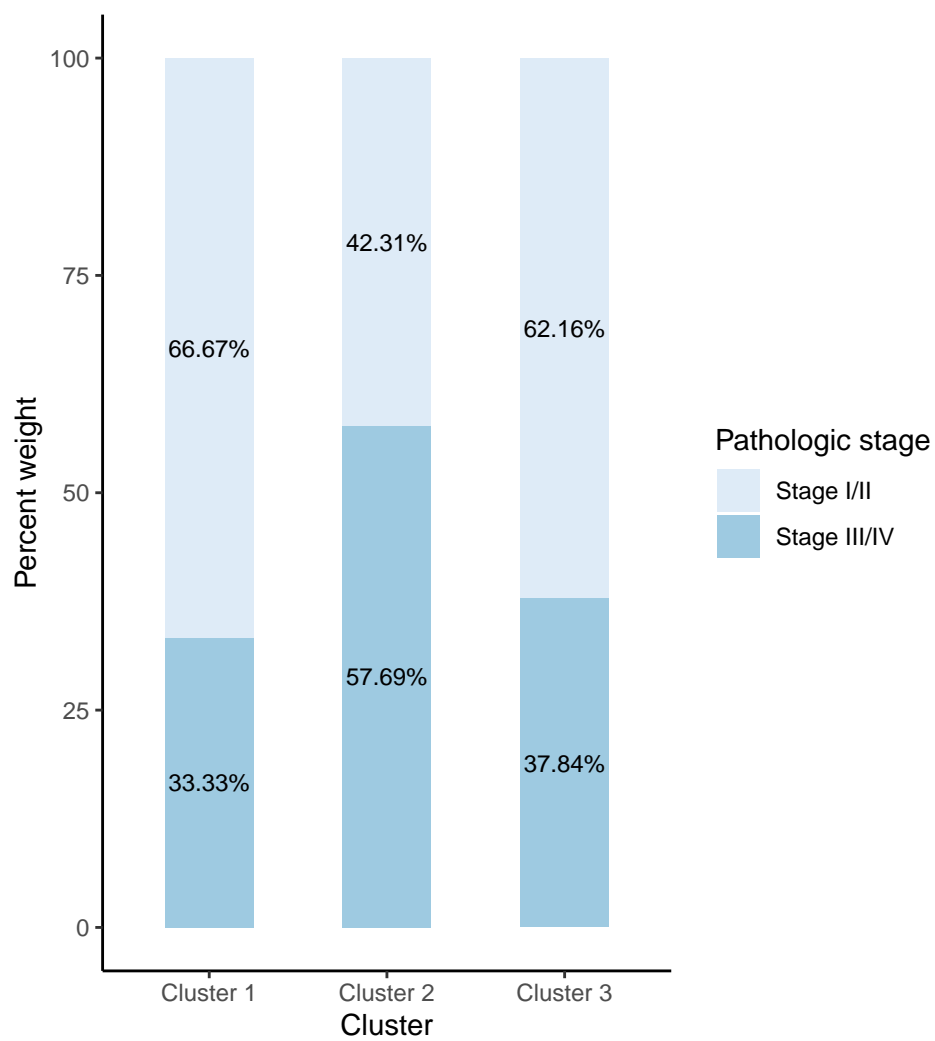

Supplement: Supplementary file 1 [file DataSheet_1.zip › original data 1-3/1-cluster/Pathologic_stage-barplot.pdf]

Survival probability

Cluster 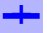 Cluster 1 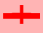 Cluster 2 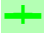 Cluster 3

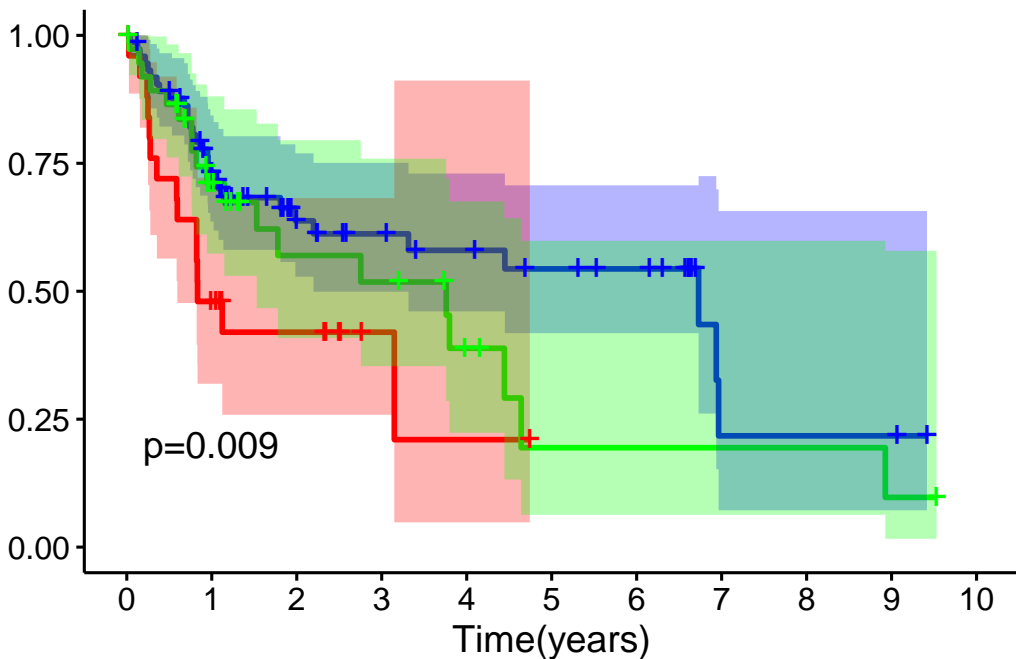

Cluster

Cluster 1  
Cluster 2  
Cluster 3

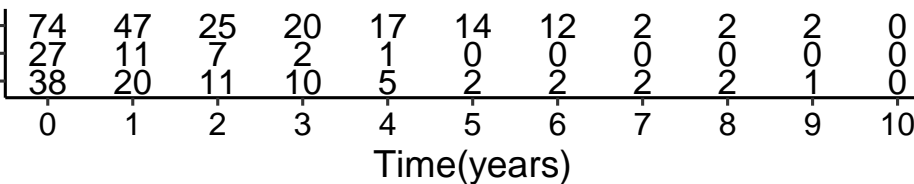

Supplement: Supplementary file 1 [file DataSheet_1.zip › original data 1-3/1-cluster/Survival.pdf]

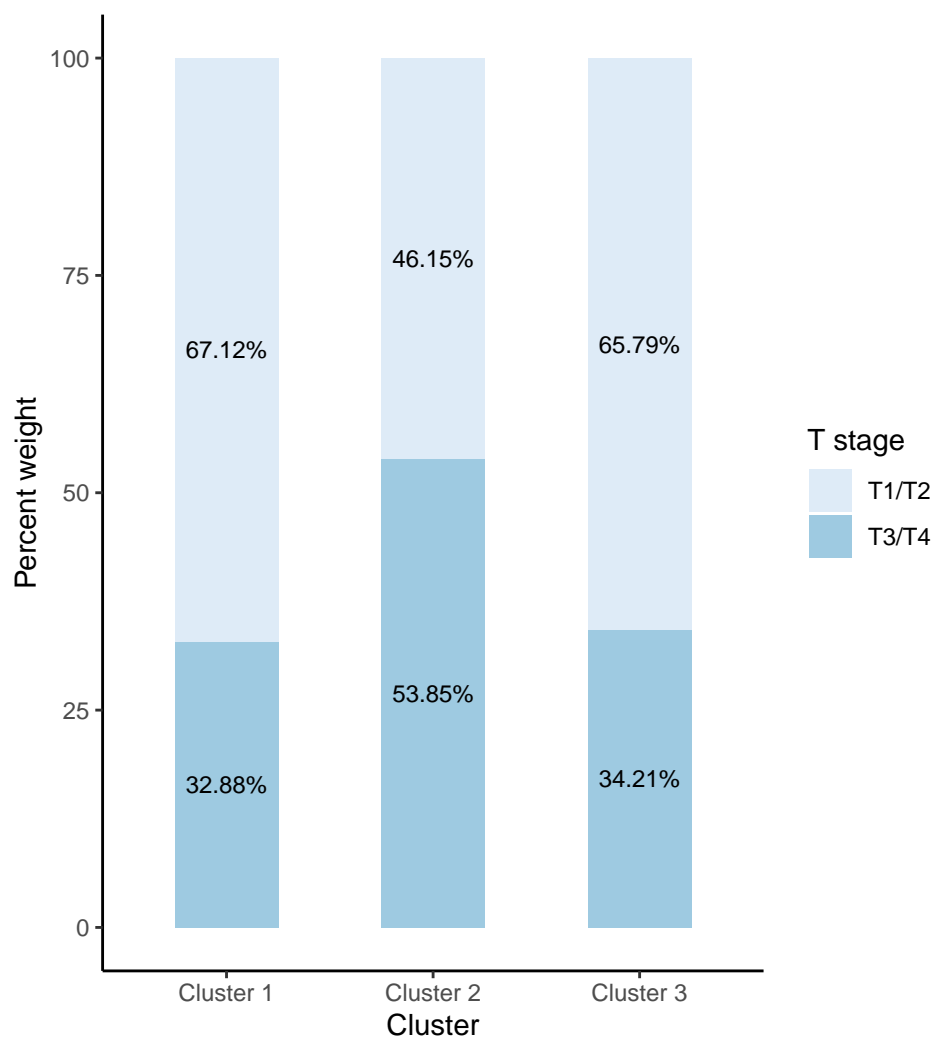

Supplement: Supplementary file 1 [file DataSheet_1.zip › original data 1-3/1-cluster/Tstage-barplot.pdf]

Cluster 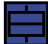 Cluster 1 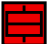 Cluster 2 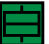 Cluster 3

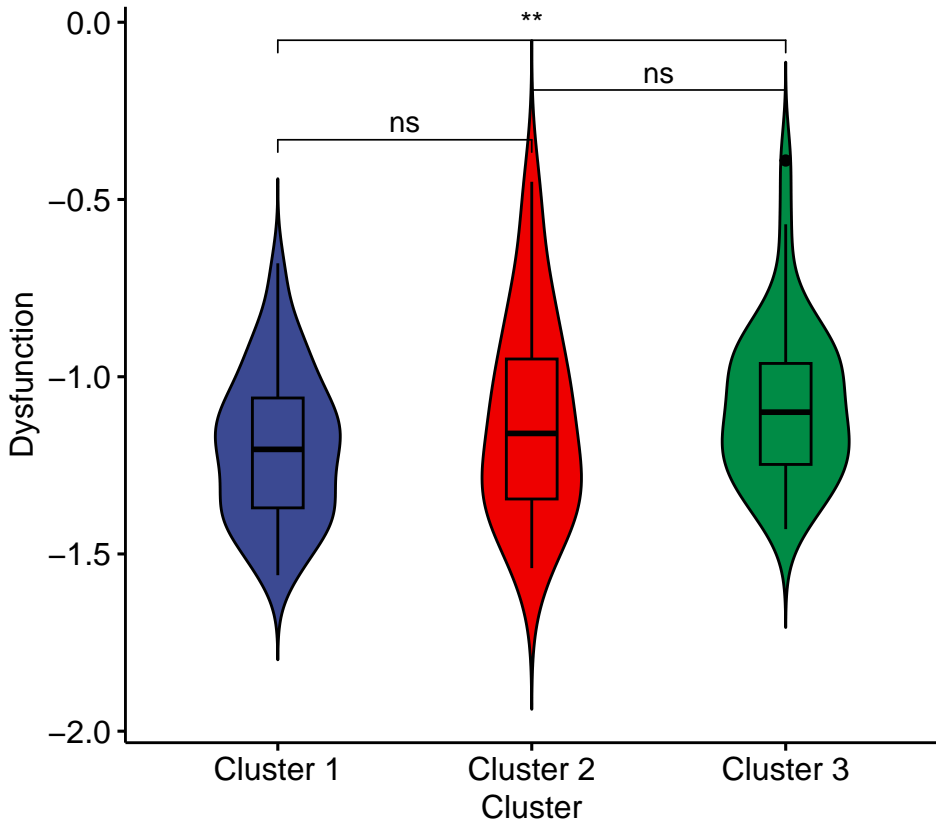

Supplement: Supplementary file 1 [file DataSheet_1.zip › original data 1-3/2-cluster immune/Dysfunction.pdf]

Cluster Cluster 1 Cluster 2 Cluster 3

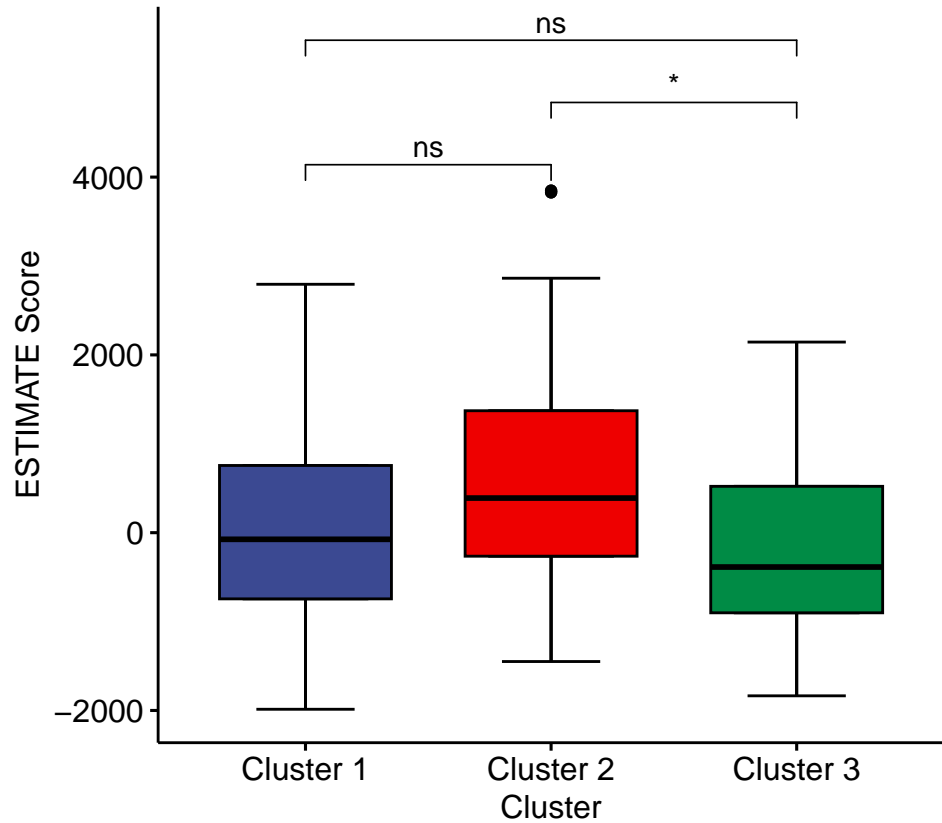

Supplement: Supplementary file 1 [file DataSheet_1.zip › original data 1-3/2-cluster immune/ESTIMATEScore.pdf]

Cluster 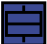 Cluster 1 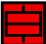 Cluster 2 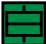 Cluster 3

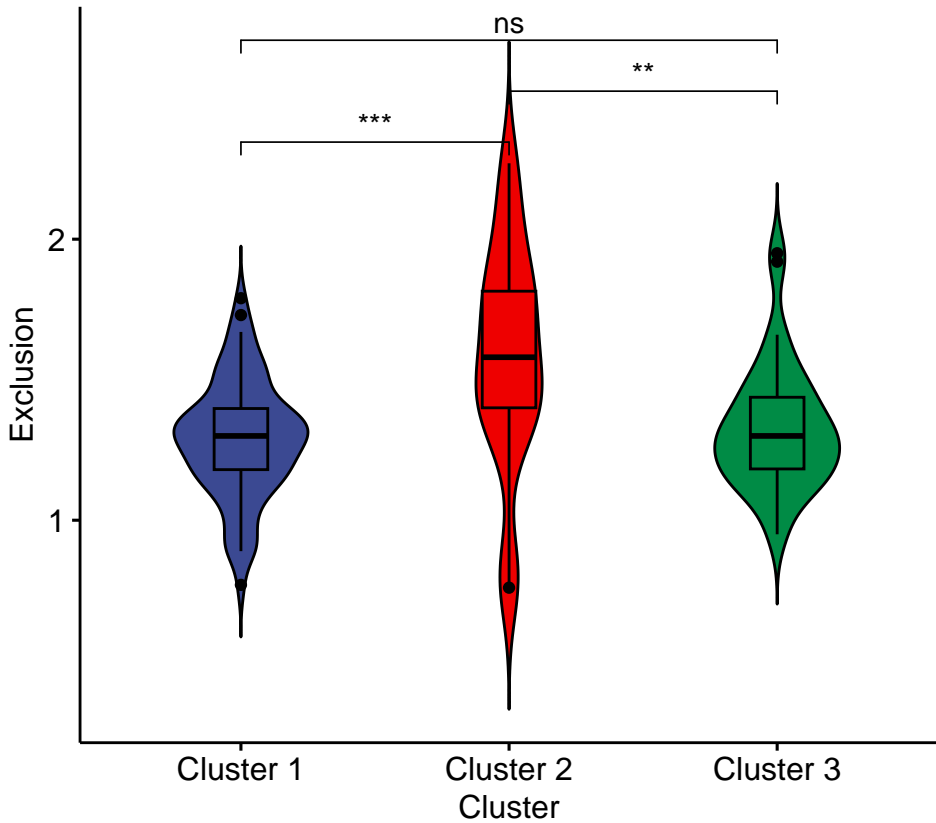

Supplement: Supplementary file 1 [file DataSheet_1.zip › original data 1-3/2-cluster immune/Exclusion.pdf]

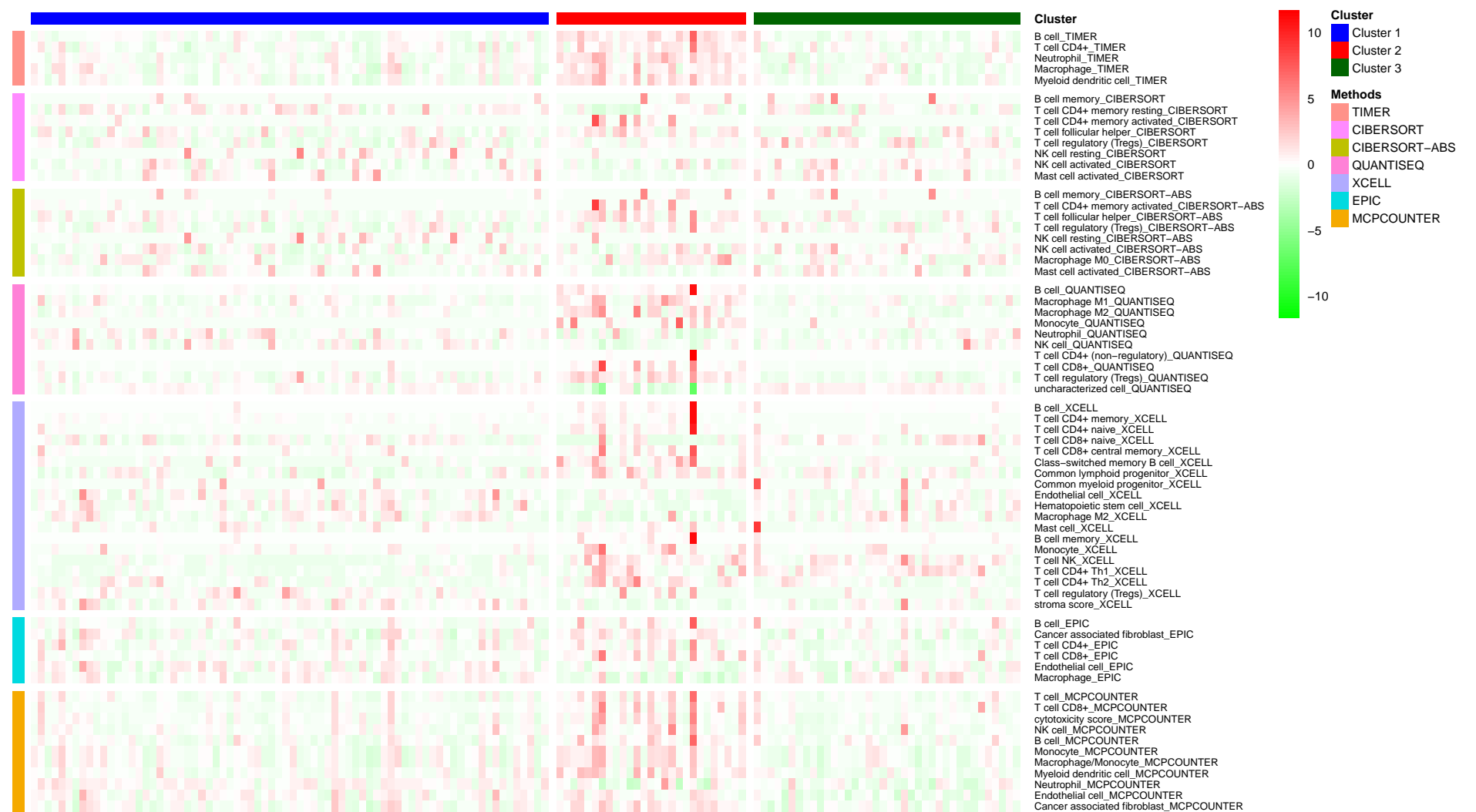

Supplement: Supplementary file 1 [file DataSheet_1.zip › original data 1-3/2-cluster immune/immHeatmap.pdf]

Cluster Cluster 1 Cluster 2 Cluster 3

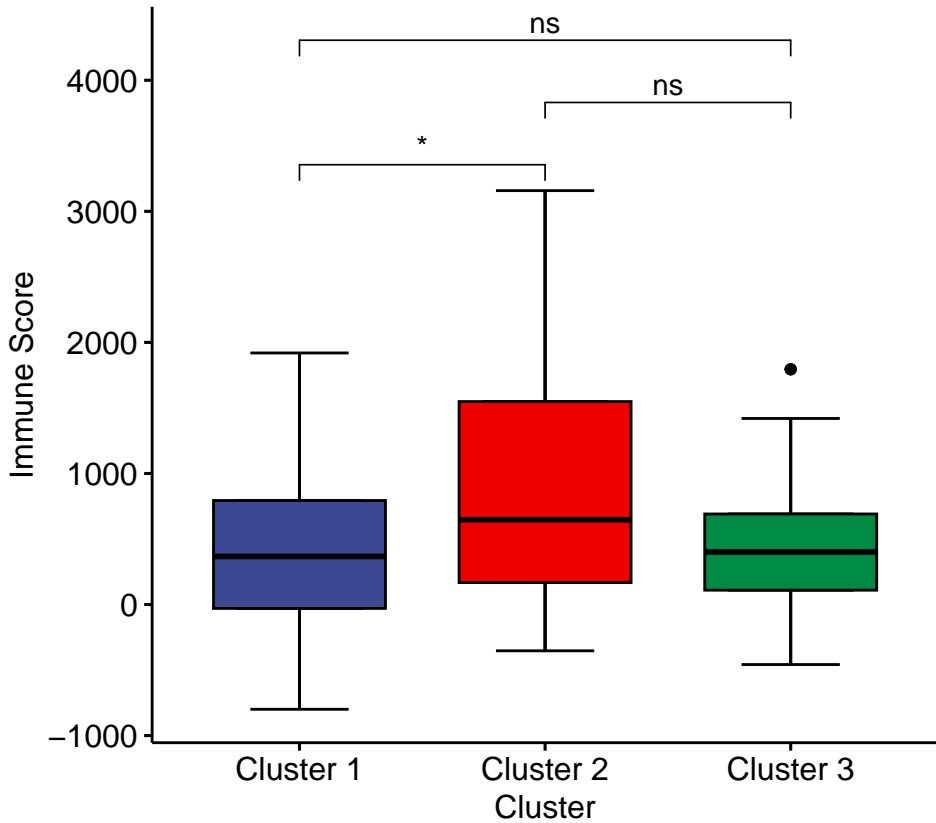

Supplement: Supplementary file 1 [file DataSheet_1.zip › original data 1-3/2-cluster immune/Immune Score.pdf]

Cluster 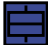 Cluster 1 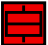 Cluster 2 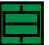 Cluster 3

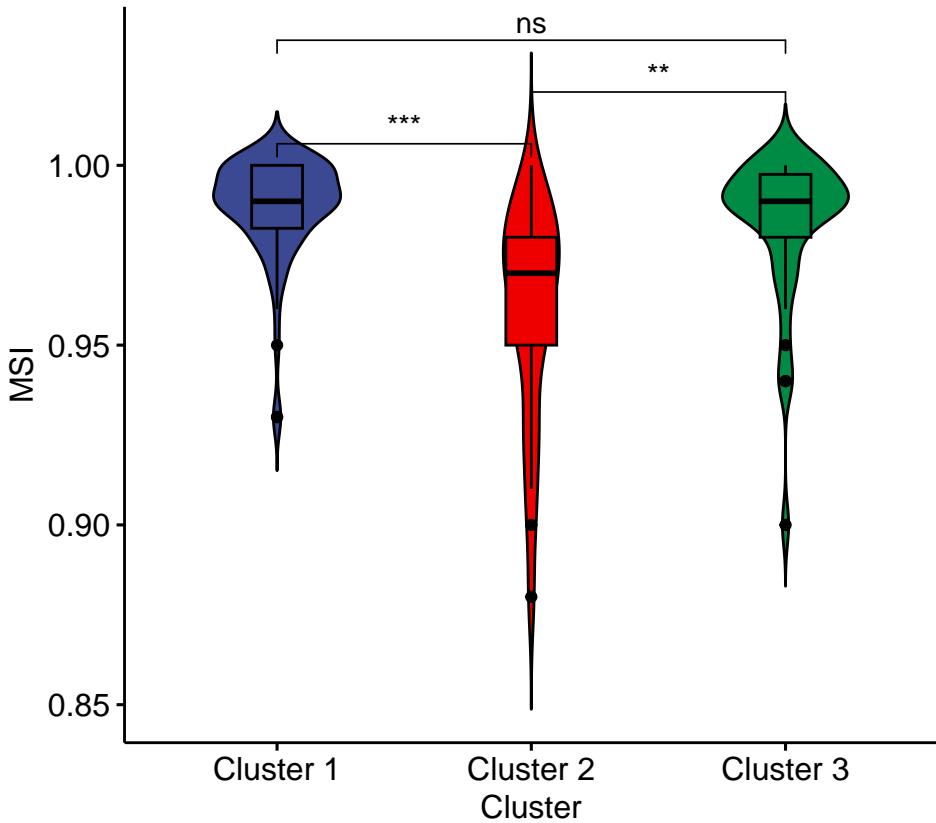

Supplement: Supplementary file 1 [file DataSheet_1.zip › original data 1-3/2-cluster immune/MSI.pdf]

Cluster Cluster 1 Cluster 2 Cluster 3

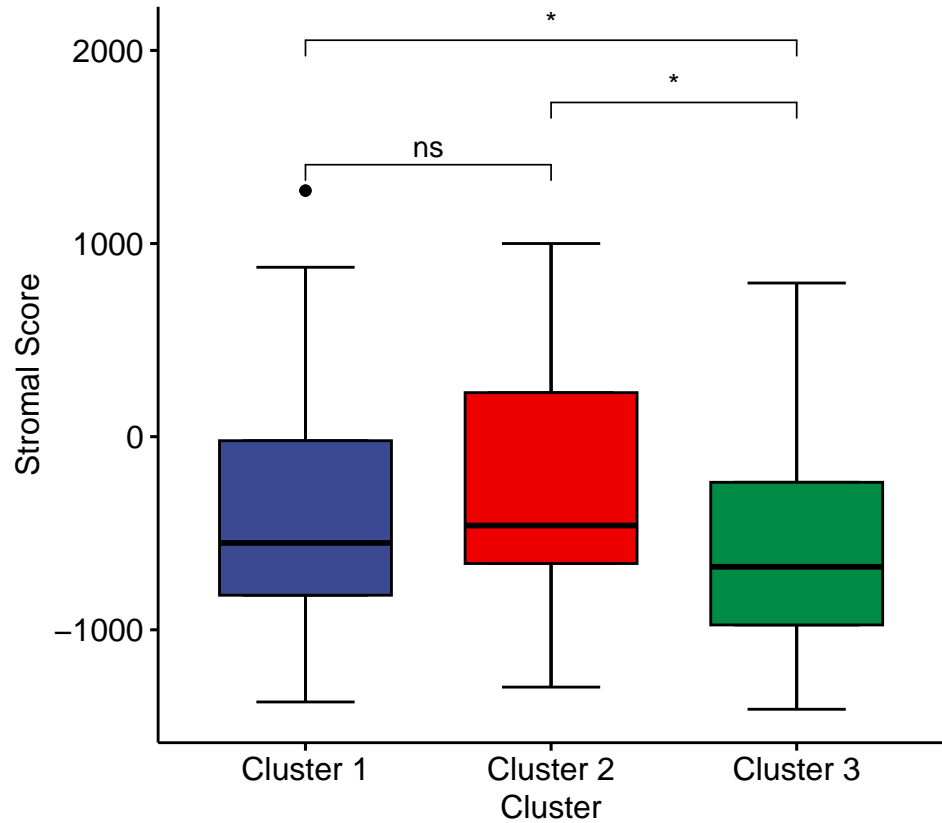

Supplement: Supplementary file 1 [file DataSheet_1.zip › original data 1-3/2-cluster immune/Stromal Score.pdf]

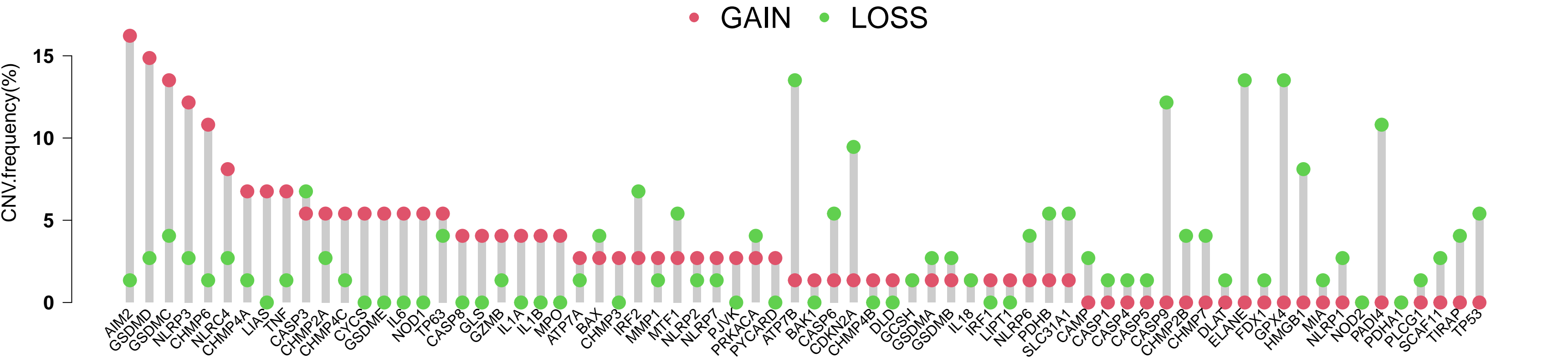

Supplement: Supplementary file 1 [file DataSheet_1.zip › original data 1-3/3-cluster pathway/CNVfreq-cluster1.pdf]

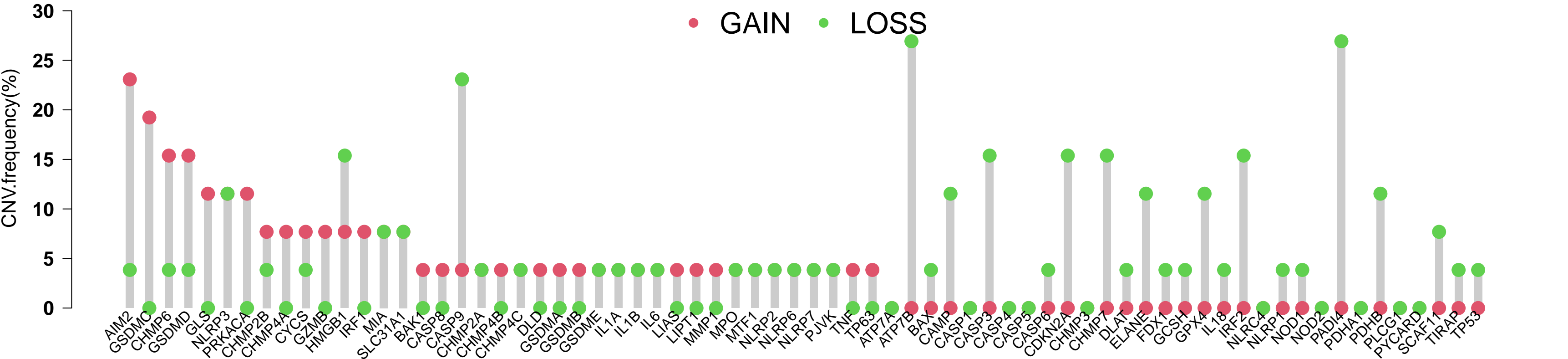

Supplement: Supplementary file 1 [file DataSheet_1.zip › original data 1-3/3-cluster pathway/CNVfreq-cluster2.pdf]

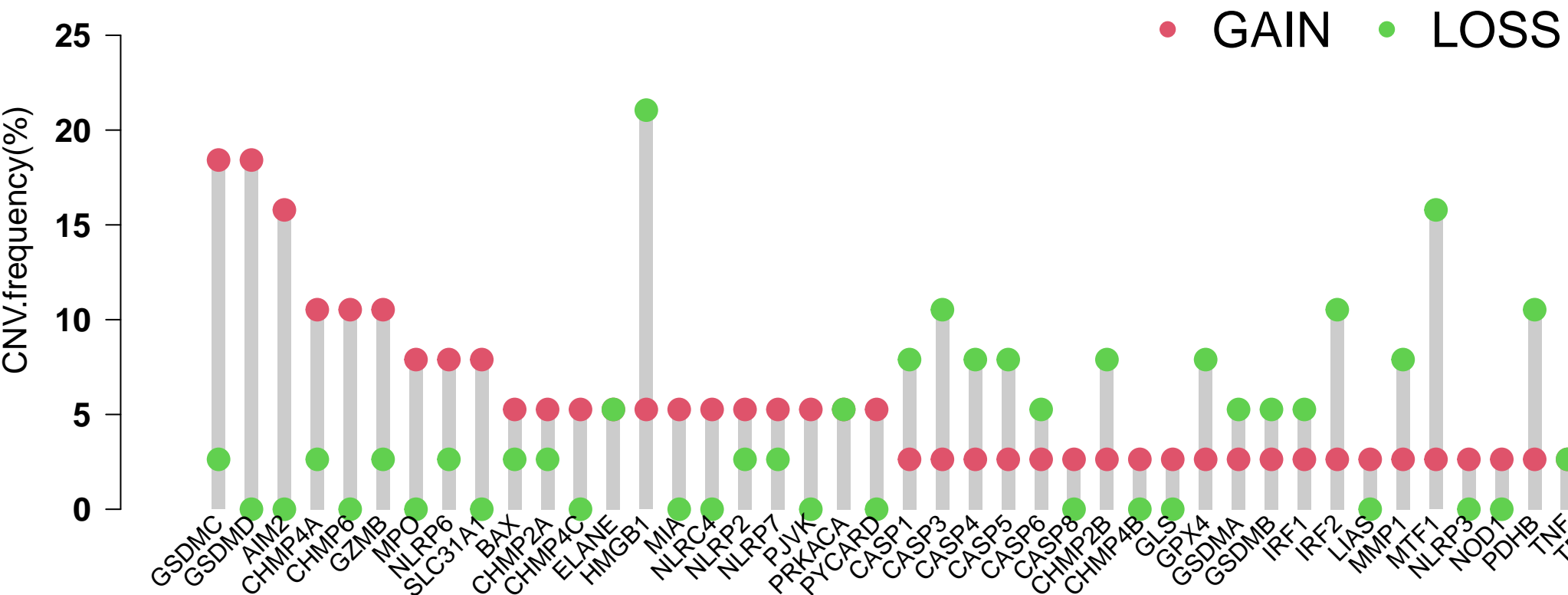

Supplement: Supplementary file 1 [file DataSheet_1.zip › original data 1-3/3-cluster pathway/CNVfreq-cluster3.pdf]

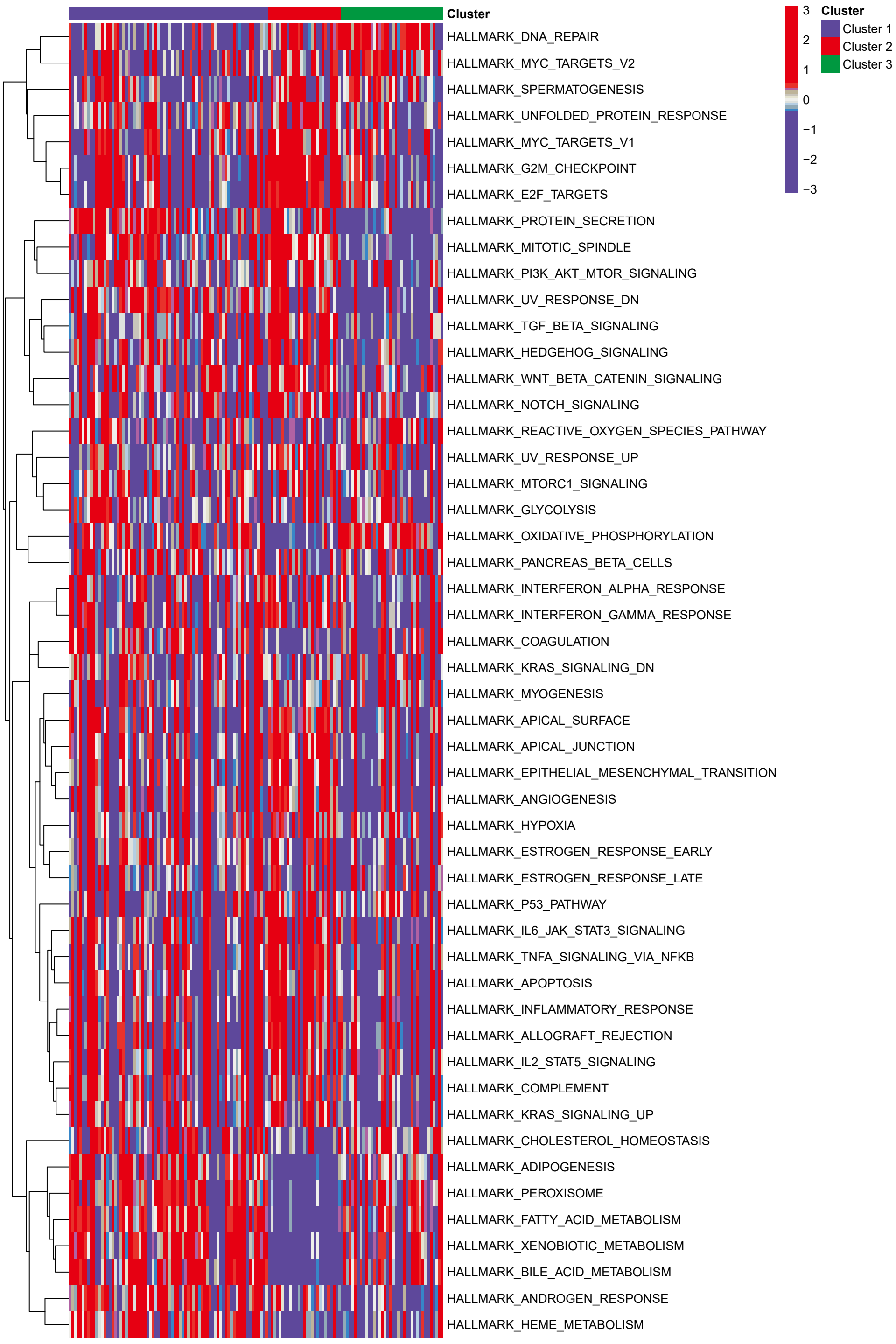

Supplement: Supplementary file 1 [file DataSheet_1.zip › original data 1-3/3-cluster pathway/immHeatmap-HALLMARK.pdf]

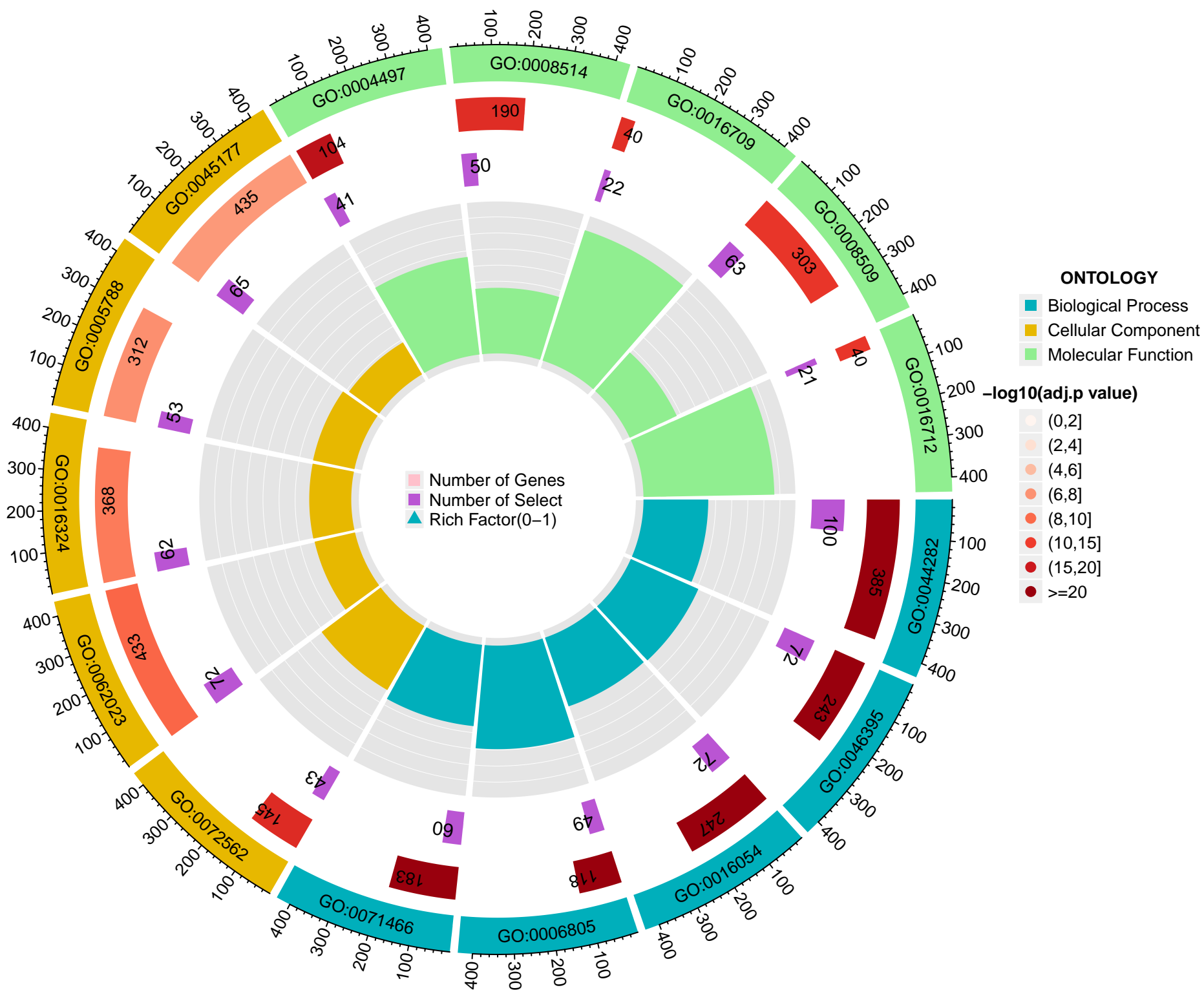

Supplement: Supplementary file 2 [file DataSheet_2.zip › original data 4-6/4-cluster diff/cluster1-GO.circlize.pdf]

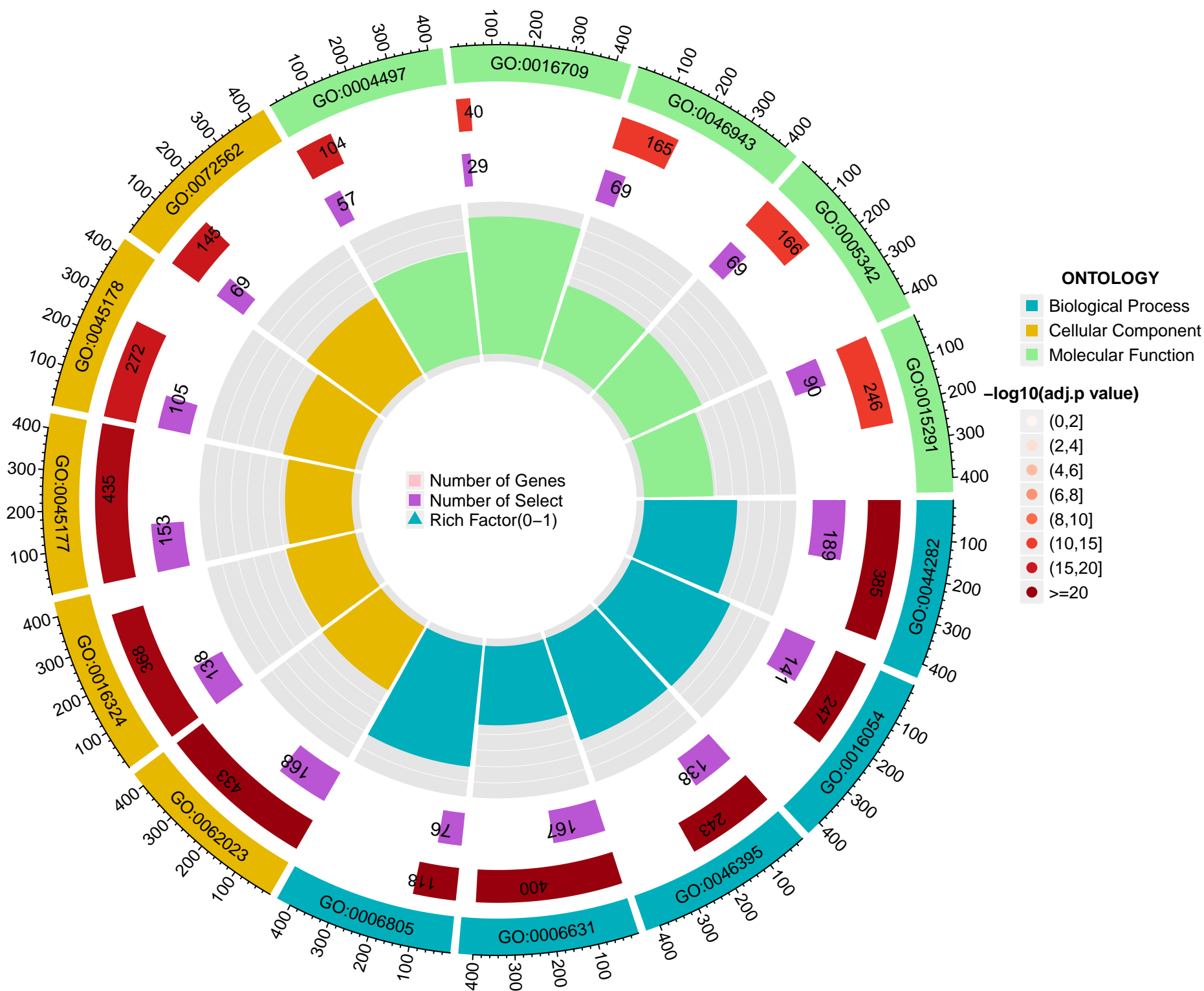

Supplement: Supplementary file 2 [file DataSheet_2.zip › original data 4-6/4-cluster diff/cluster2-GO.circlize.pdf]

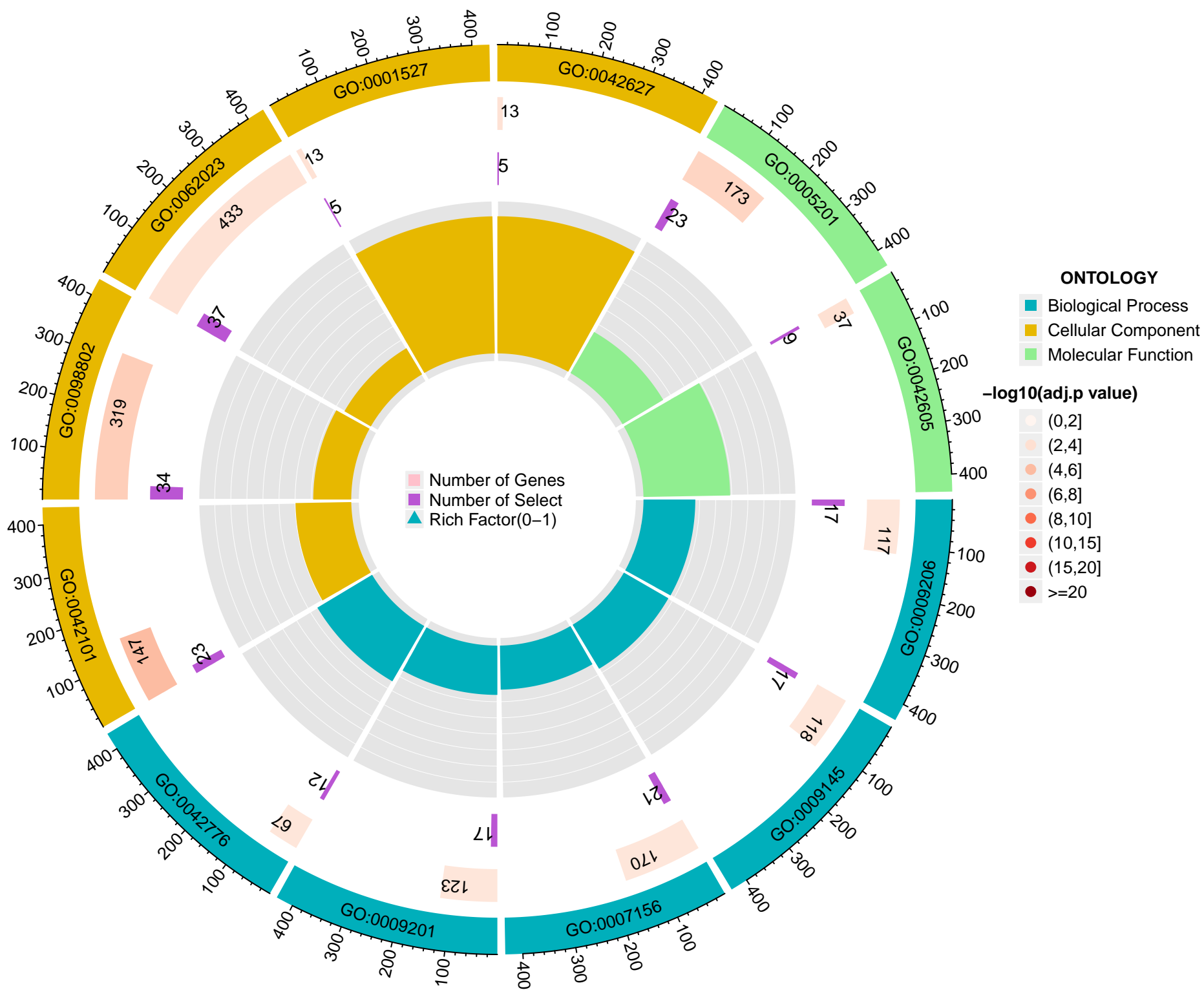

Supplement: Supplementary file 2 [file DataSheet_2.zip › original data 4-6/4-cluster diff/cluster3-GO.circlize.pdf]

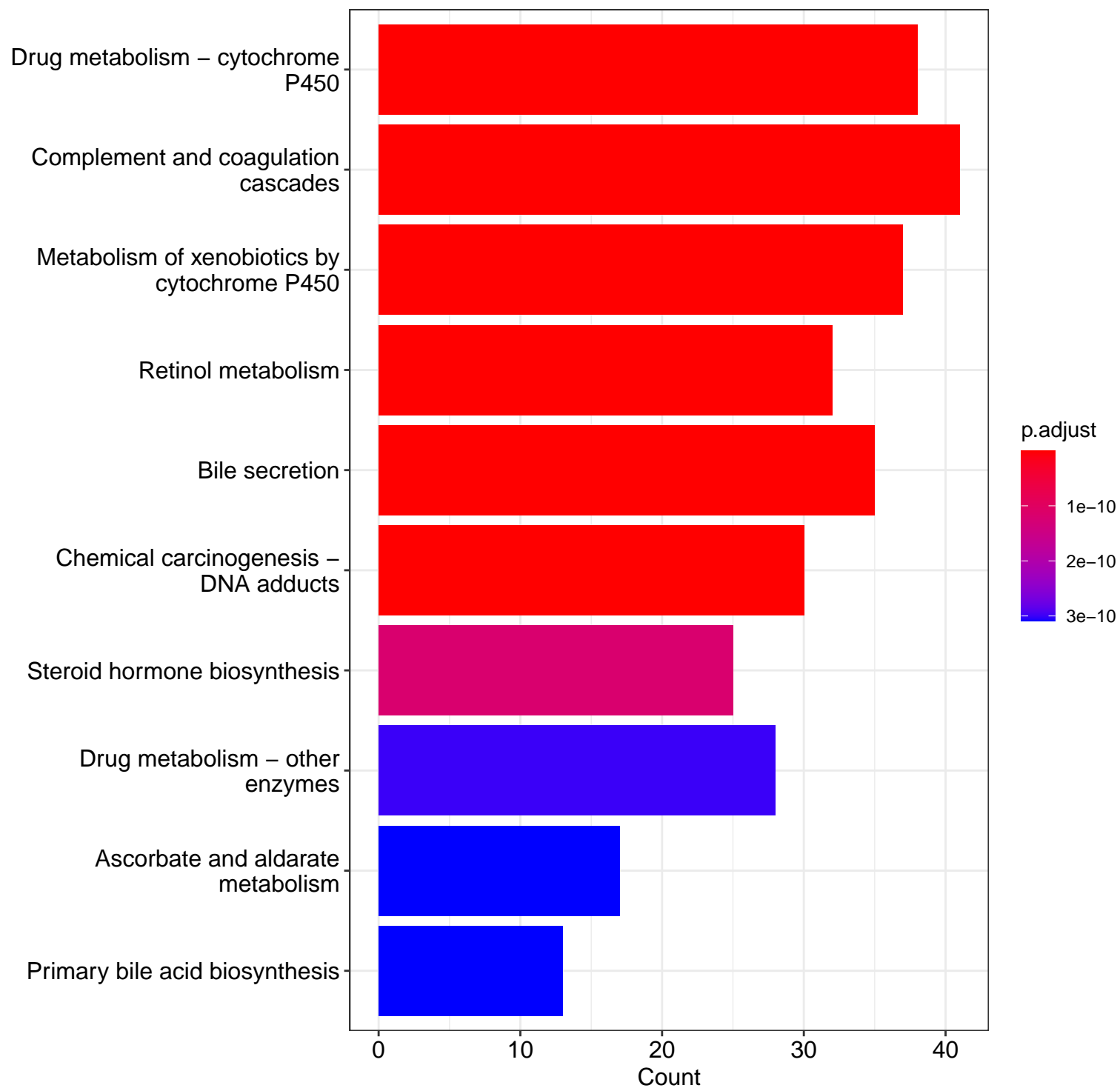

Supplement: Supplementary file 2 [file DataSheet_2.zip › original data 4-6/4-cluster diff/KEGG.barplot-cluster1.pdf]

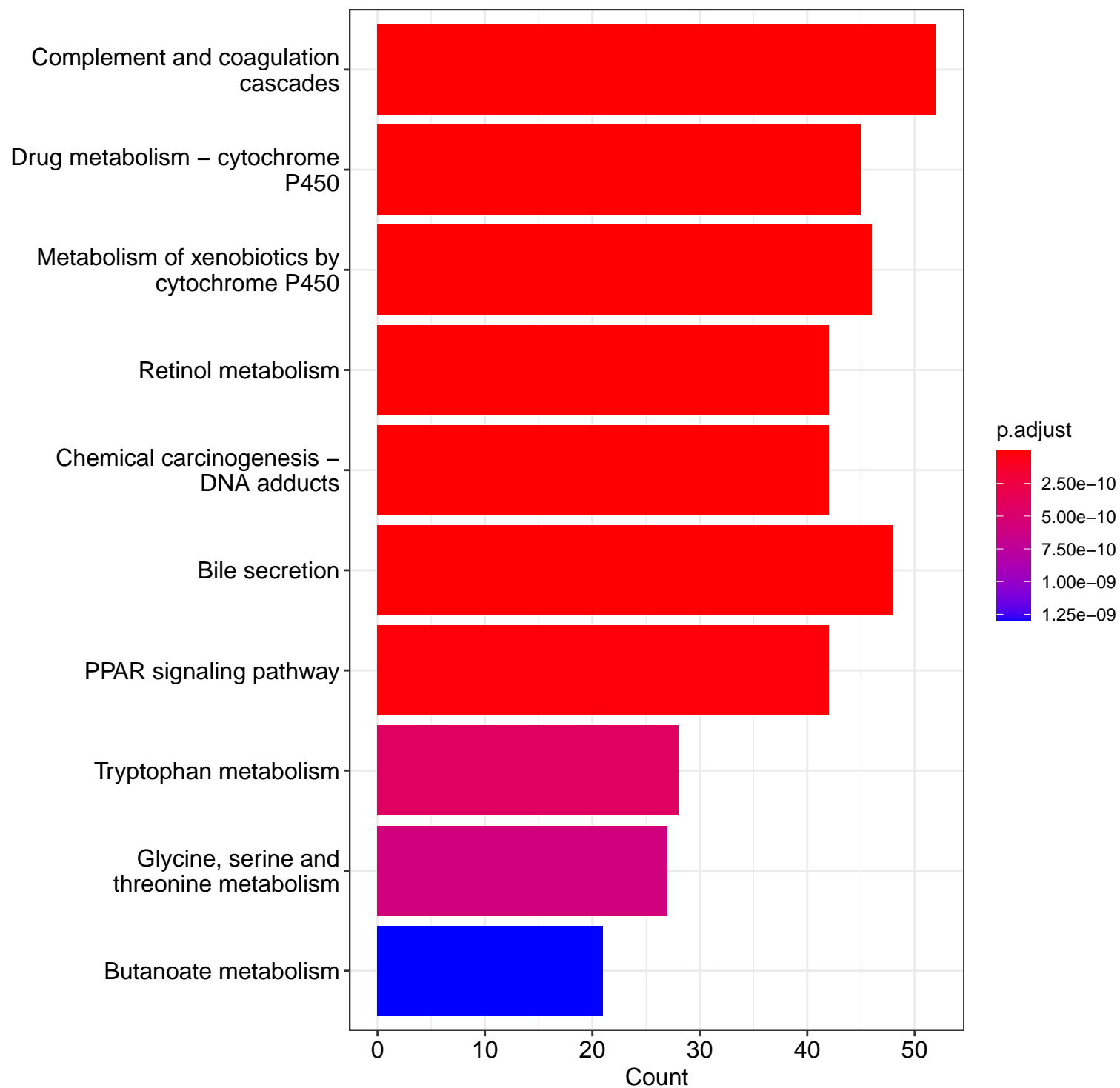

Supplement: Supplementary file 2 [file DataSheet_2.zip › original data 4-6/4-cluster diff/KEGG.barplot-cluster2.pdf]

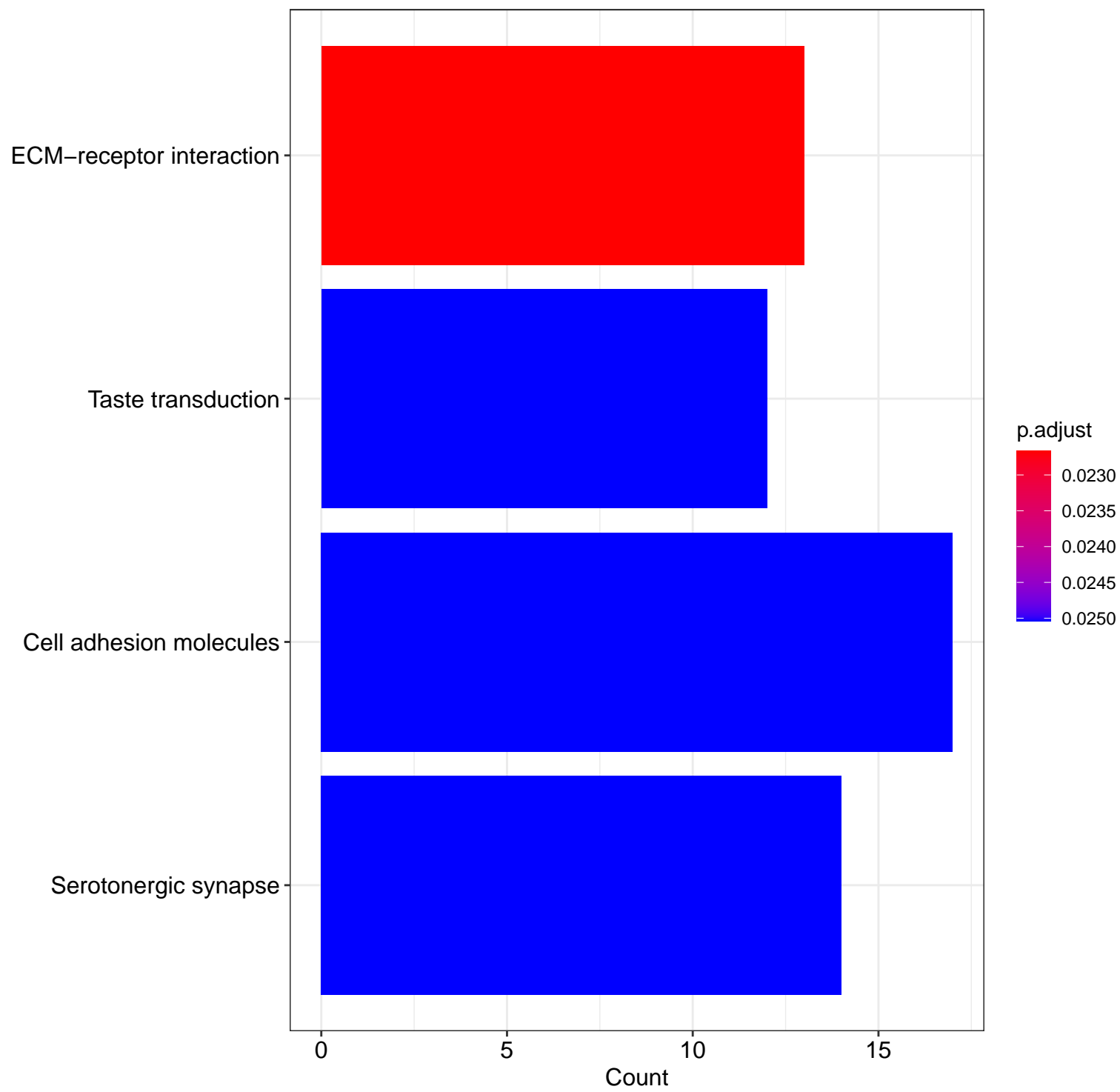

Supplement: Supplementary file 2 [file DataSheet_2.zip › original data 4-6/4-cluster diff/KEGG.barplot-cluster3.pdf]

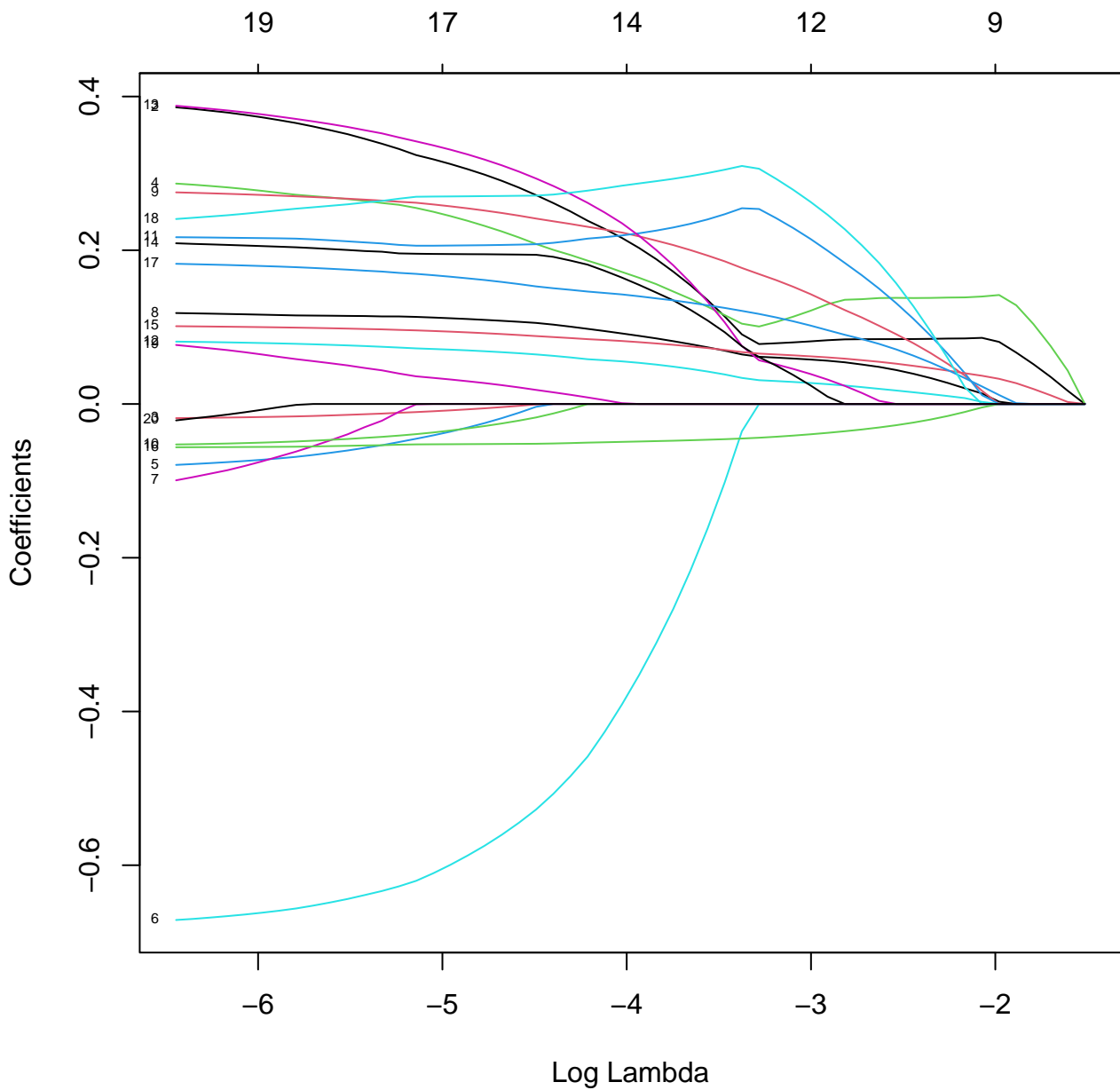

Supplement: Supplementary file 2 [file DataSheet_2.zip › original data 4-6/5-model/lambda.pdf]

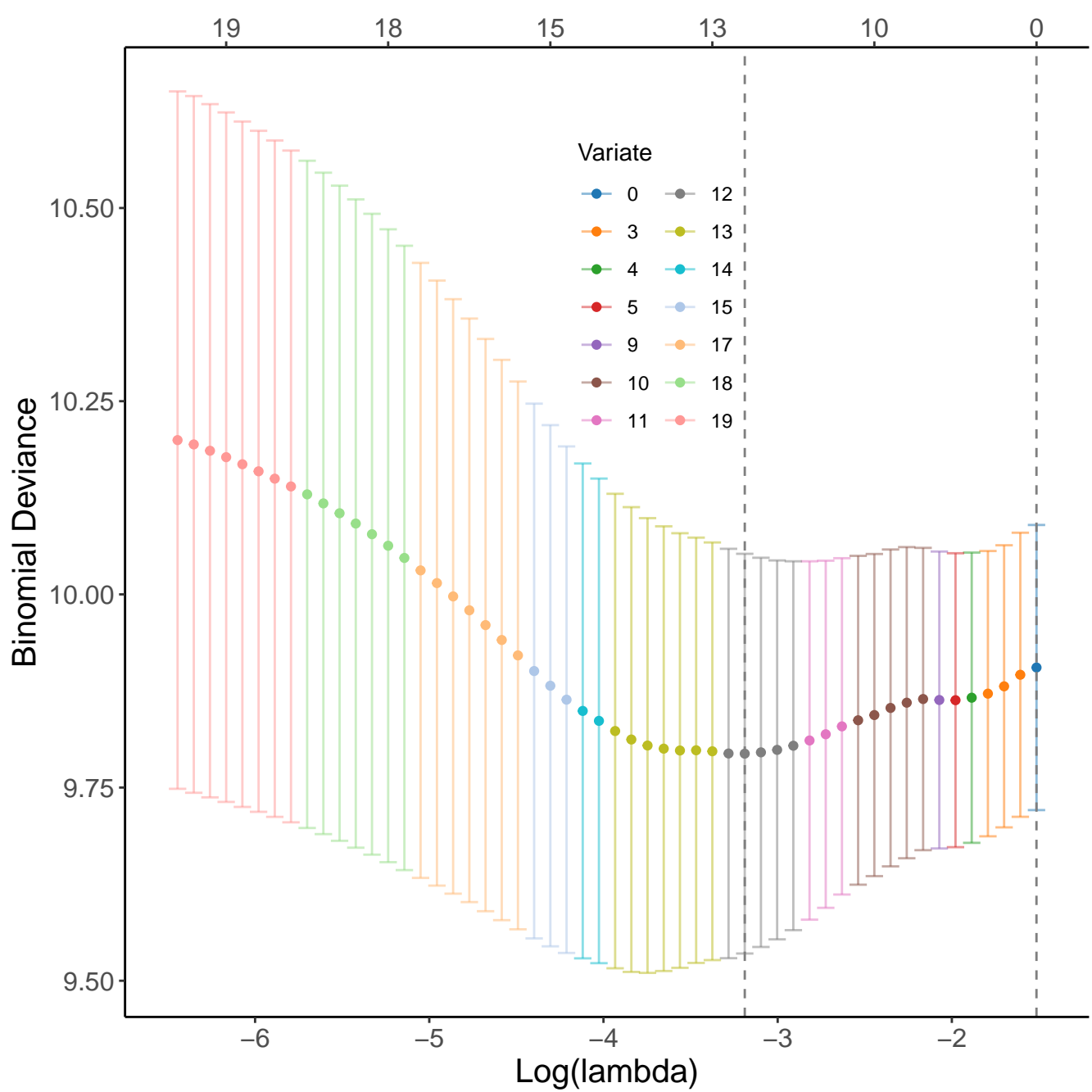

Supplement: Supplementary file 2 [file DataSheet_2.zip › original data 4-6/5-model/min.pdf]

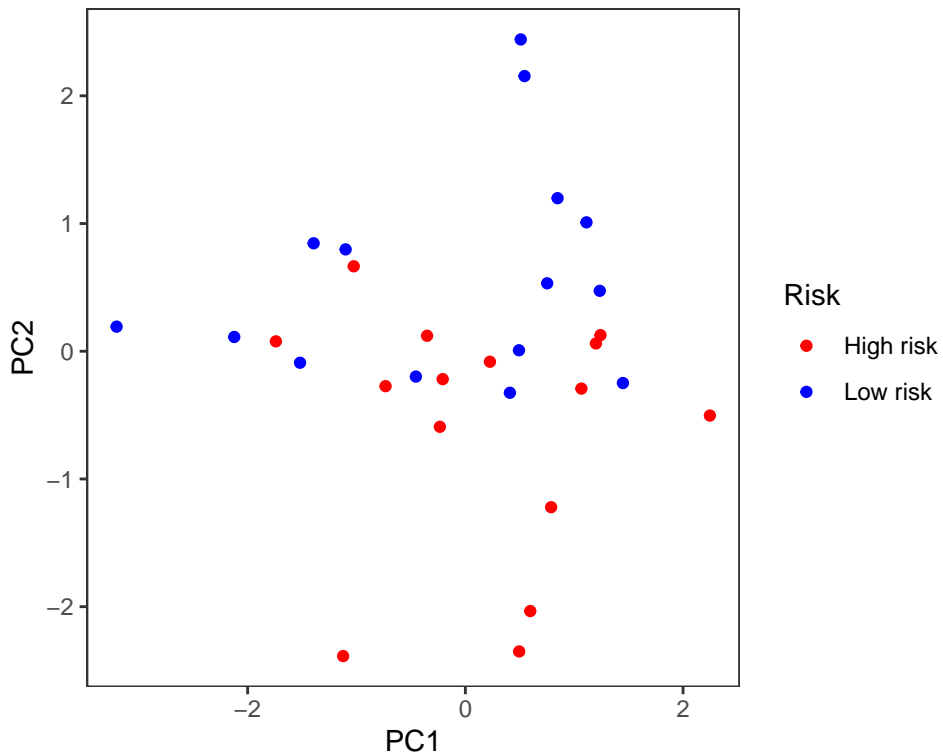

Supplement: Supplementary file 2 [file DataSheet_2.zip › original data 4-6/5-model/risk-PCA-test.pdf]

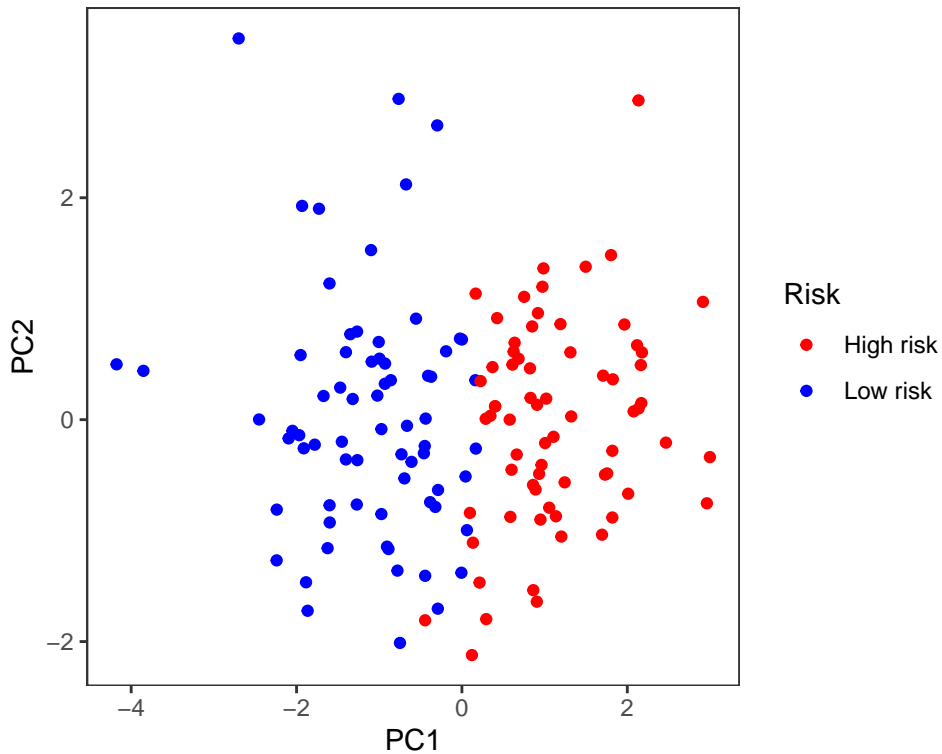

Supplement: Supplementary file 2 [file DataSheet_2.zip › original data 4-6/5-model/risk-PCA-train.pdf]

riskScore High risk Low risk

Survival probability

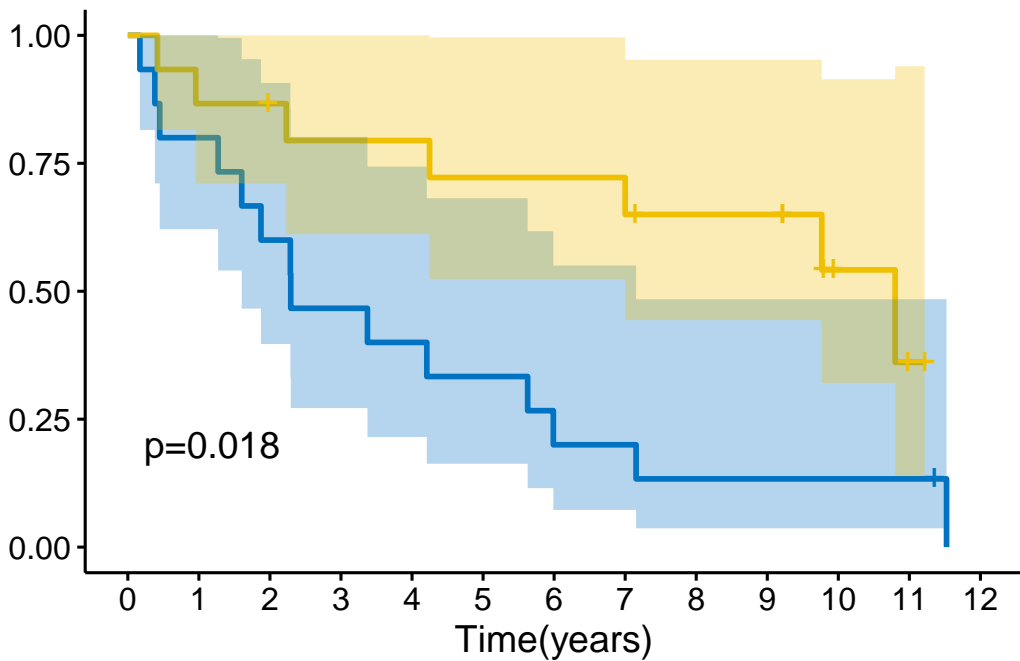

p=0.018

riskScore

High risk  
Low risk

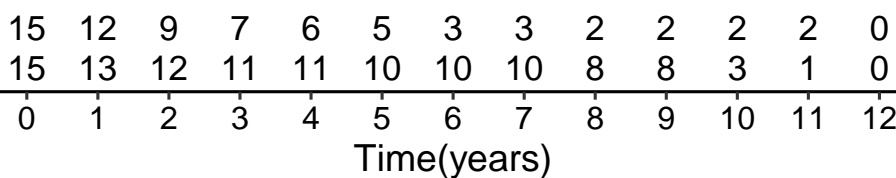

Supplement: Supplementary file 2 [file DataSheet_2.zip › original data 4-6/5-model/riskScore.survivaltestGSE141198.pdf]

Survival probability

riskScore High risk Low risk

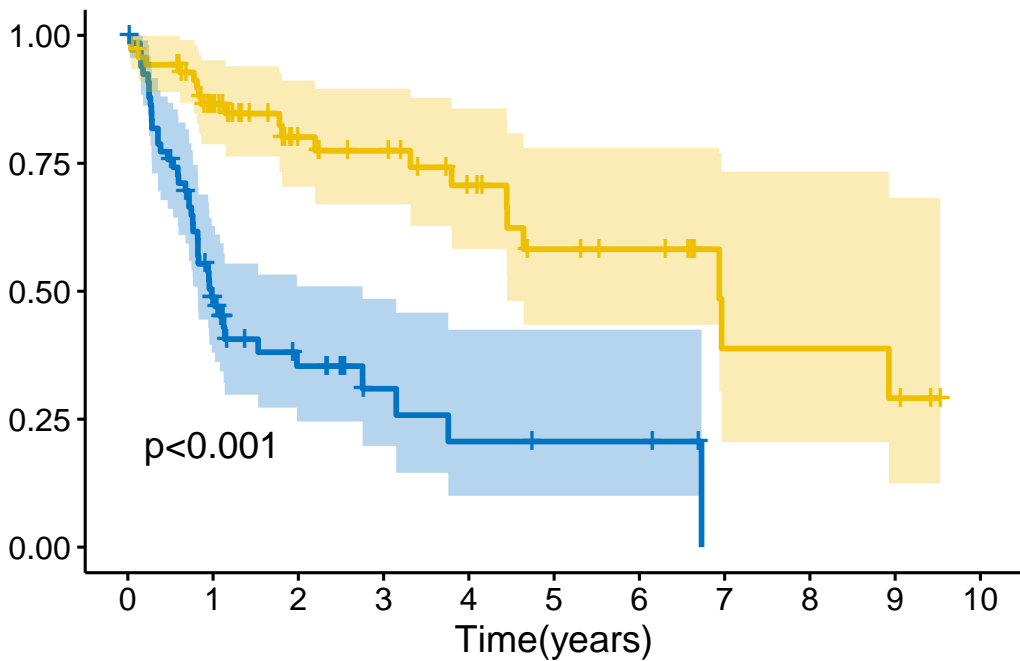

riskScore

High risk

Low risk

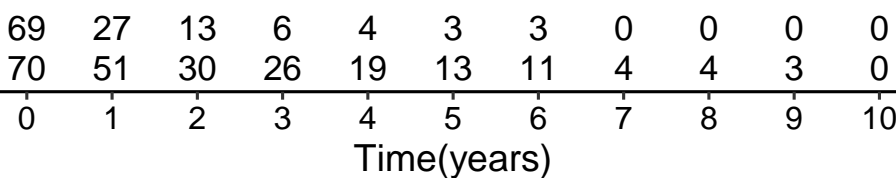

Supplement: Supplementary file 2 [file DataSheet_2.zip › original data 4-6/5-model/riskScore.survivaltrain.pdf]

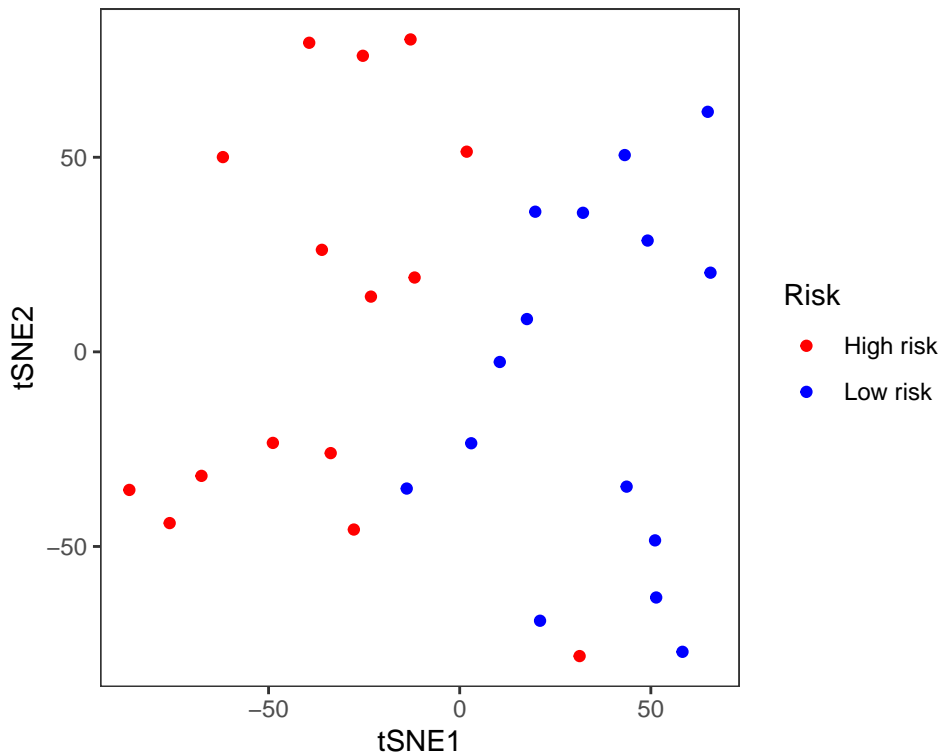

Supplement: Supplementary file 2 [file DataSheet_2.zip › original data 4-6/5-model/risk-tSNE-test.pdf]

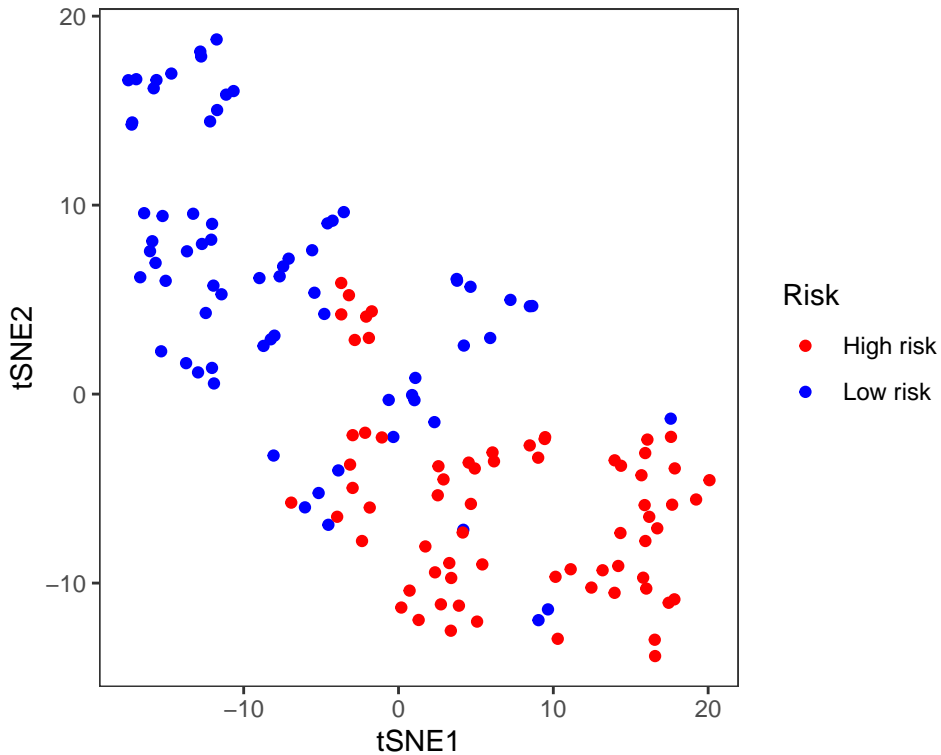

Supplement: Supplementary file 2 [file DataSheet_2.zip › original data 4-6/5-model/risk-tSNE-train.pdf]

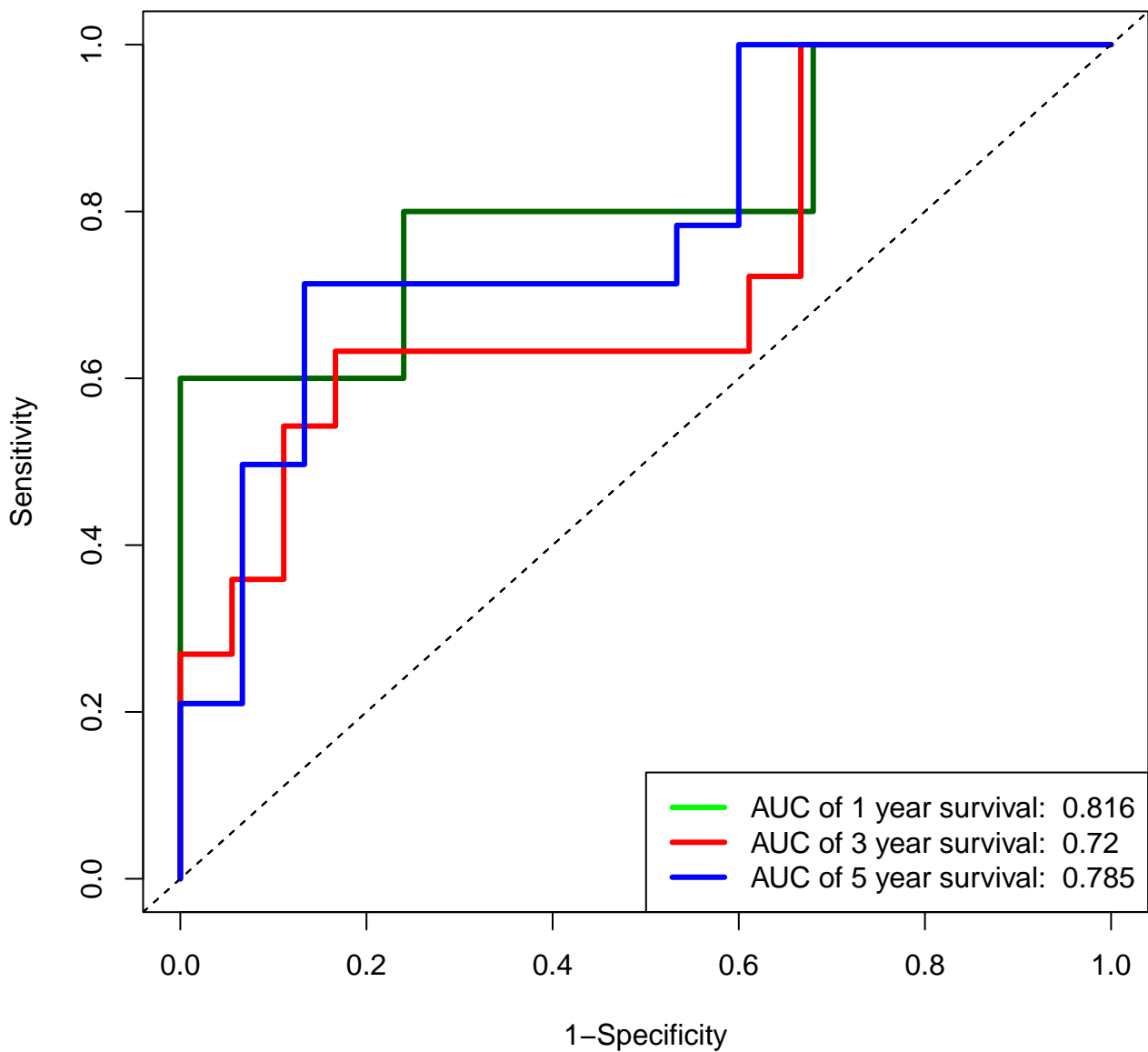

Supplement: Supplementary file 2 [file DataSheet_2.zip › original data 4-6/5-model/ROCtest.pdf]

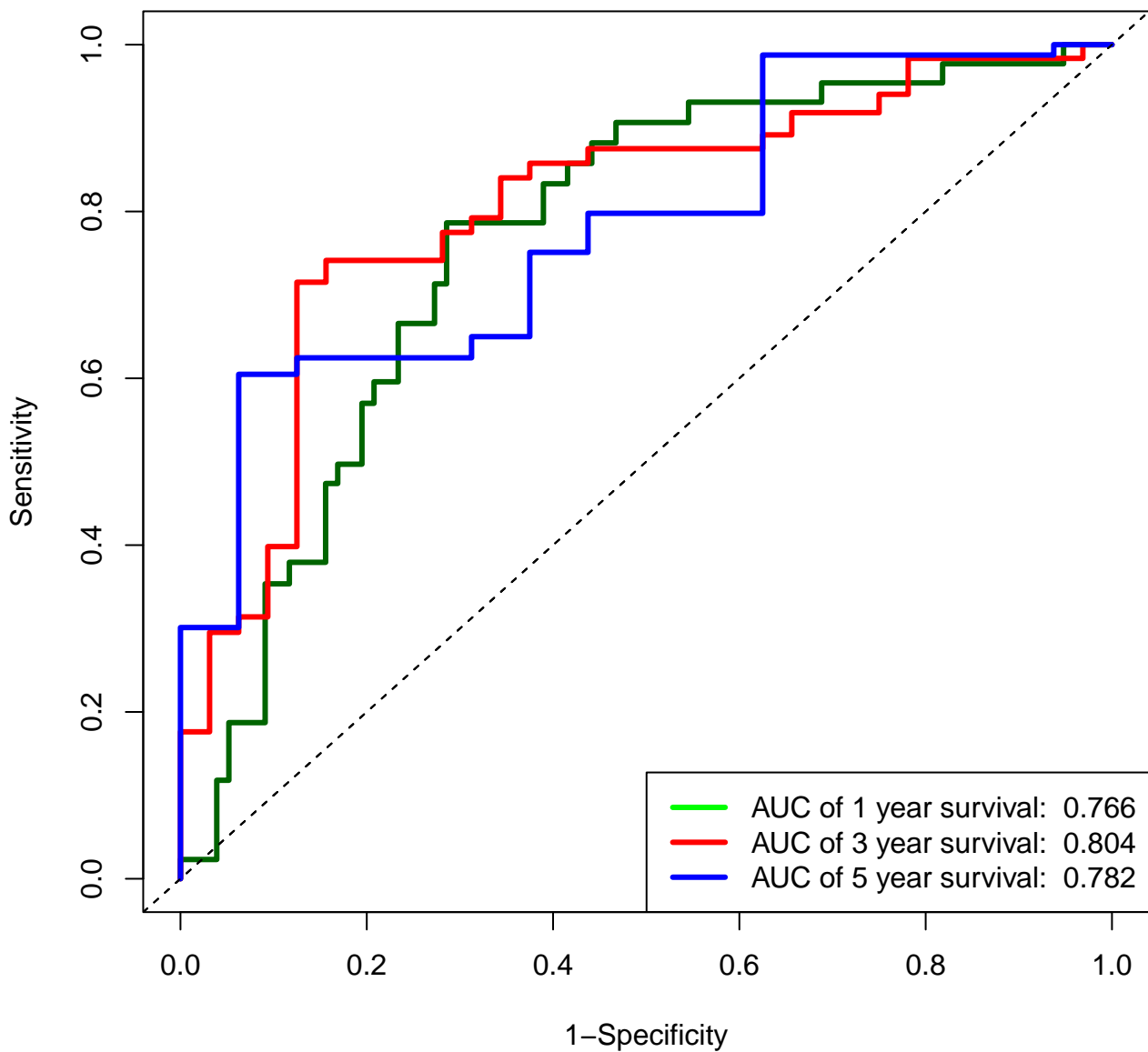

Supplement: Supplementary file 2 [file DataSheet_2.zip › original data 4-6/5-model/ROCtrain.pdf]

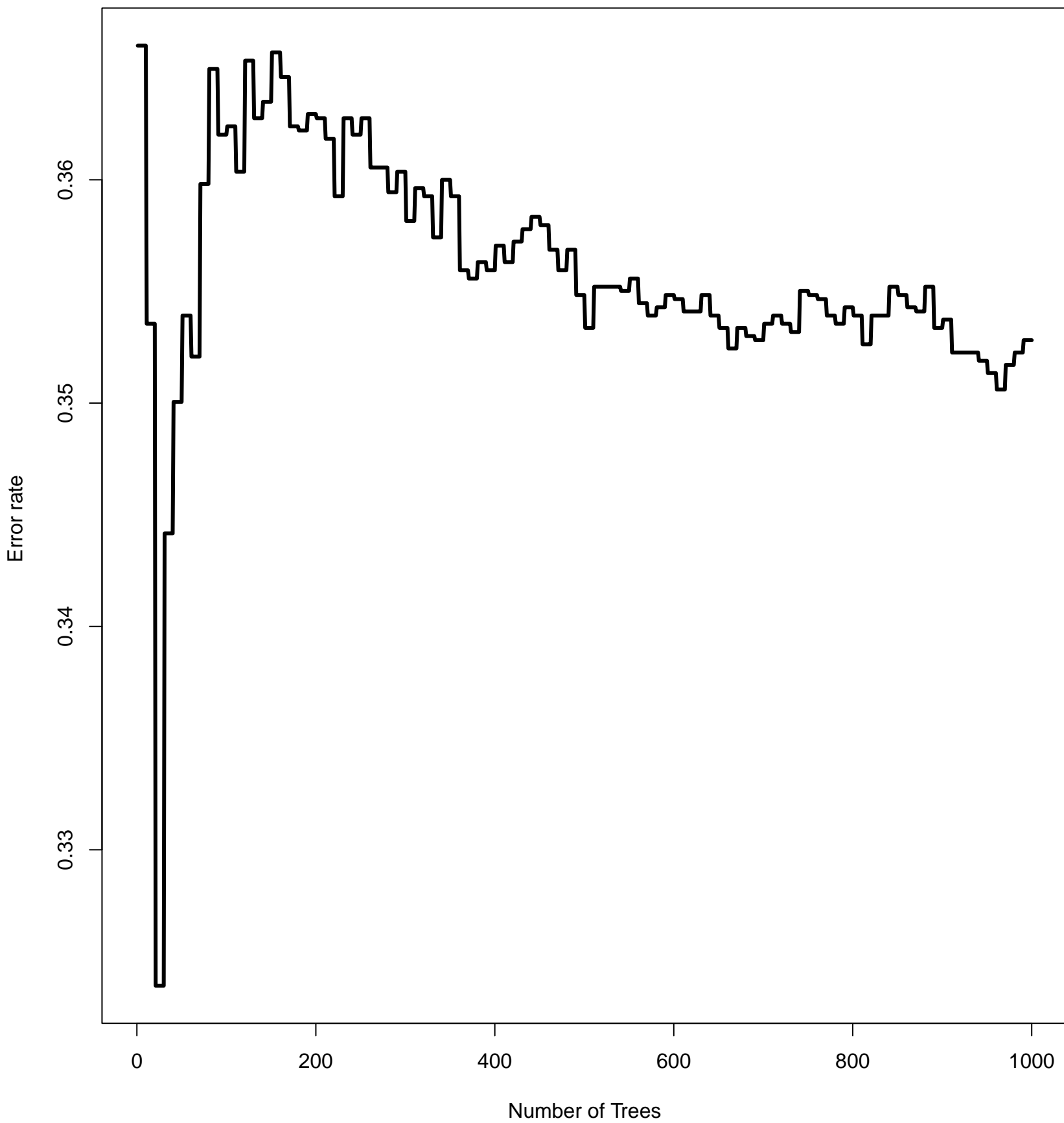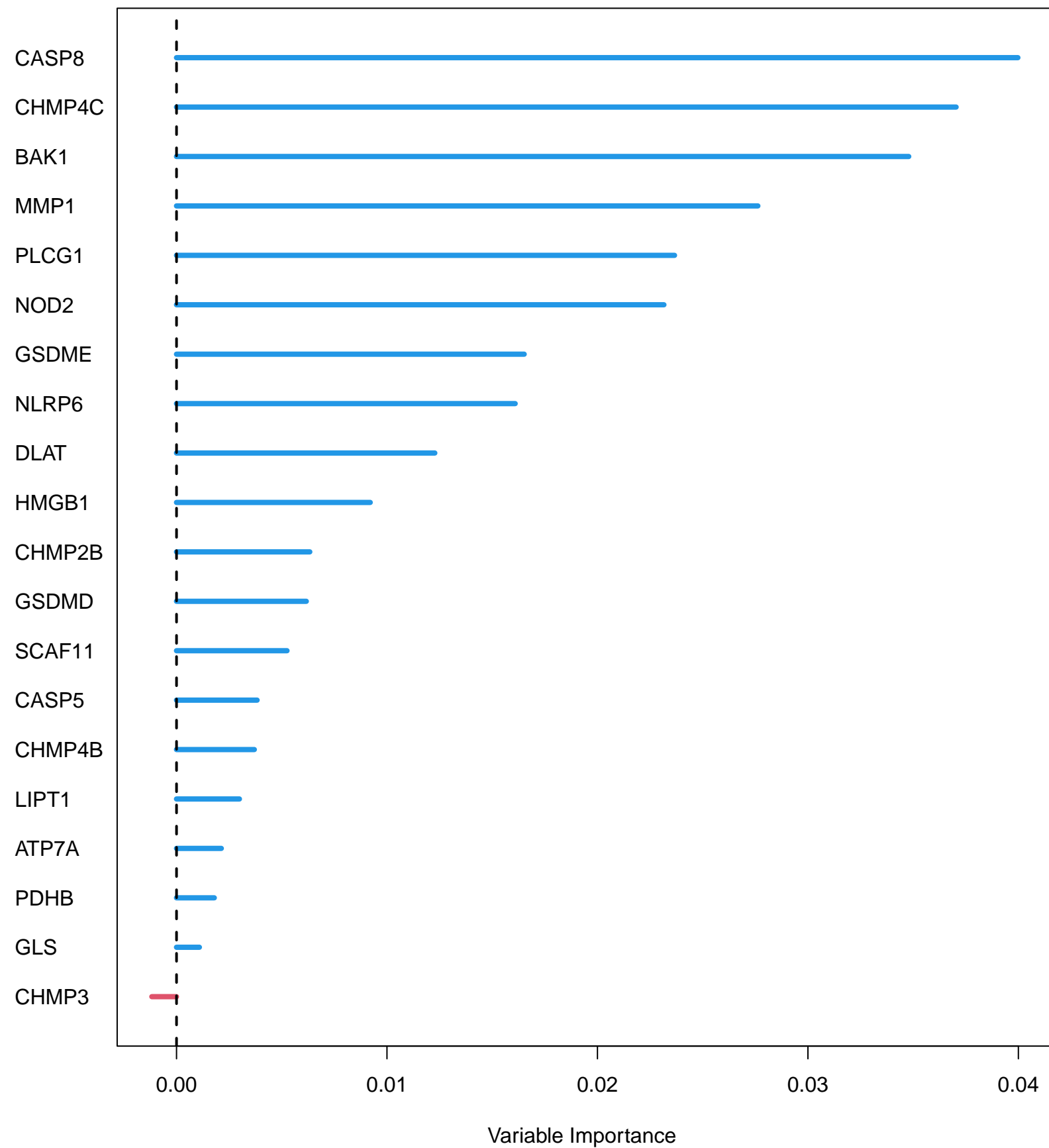

Supplement: Supplementary file 2 [file DataSheet_2.zip › original data 4-6/5-model/rsf.pdf]

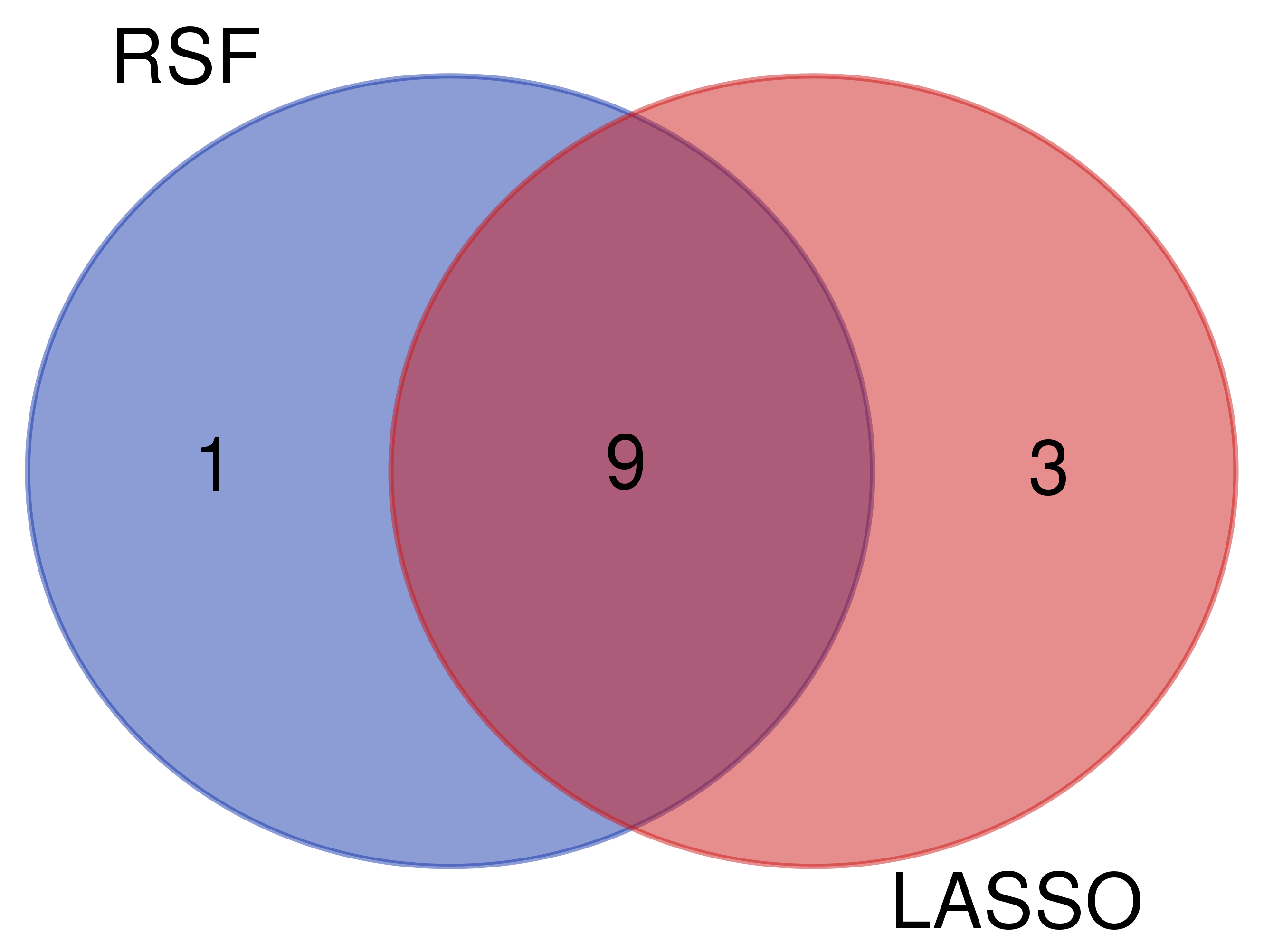

Supplement: Supplementary file 2 [file DataSheet_2.zip › original data 4-6/5-model/venn_result17961.png]

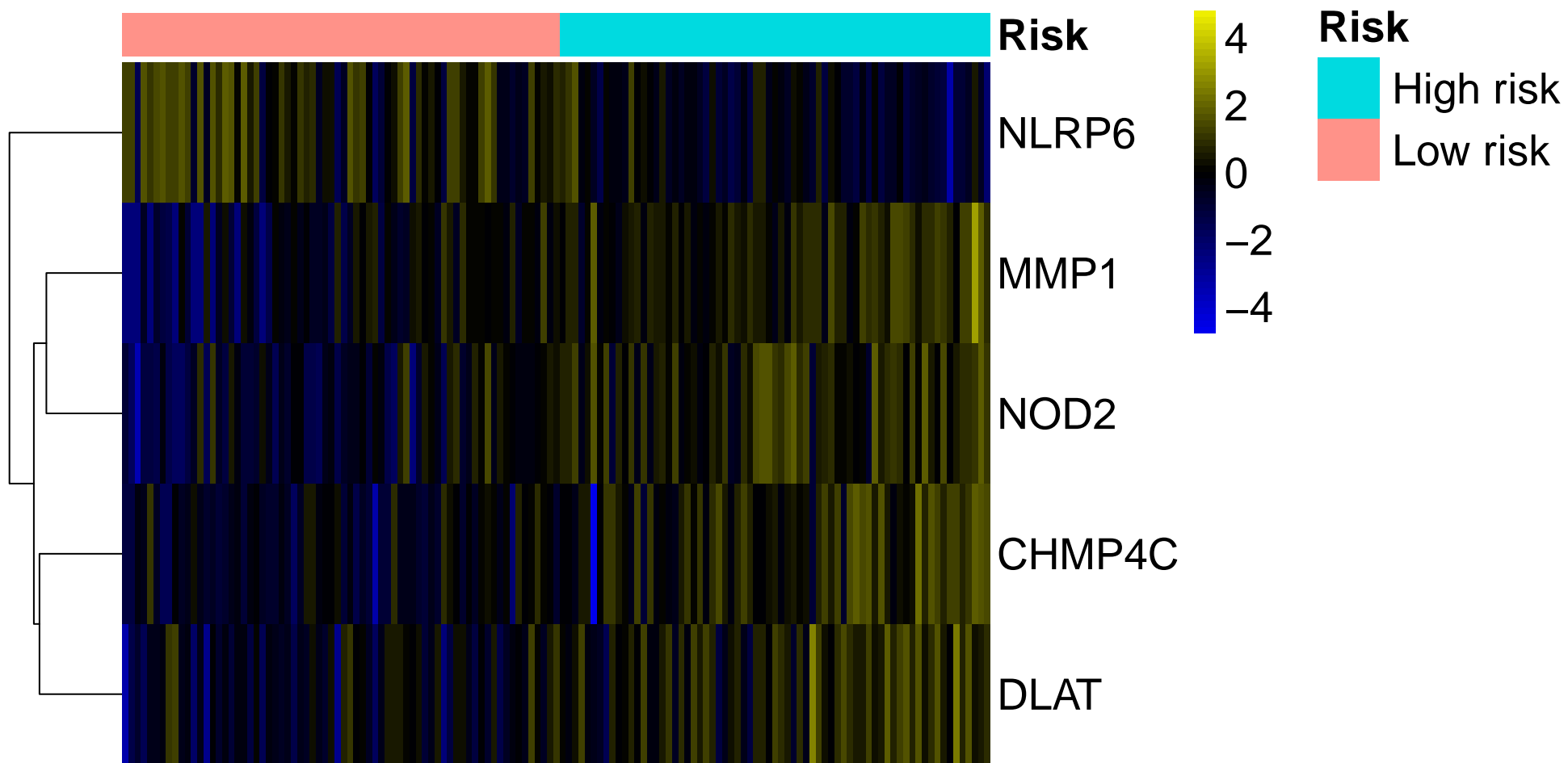

Supplement: Supplementary file 2 [file DataSheet_2.zip › original data 4-6/6-risk/risk-heatmap.pdf]

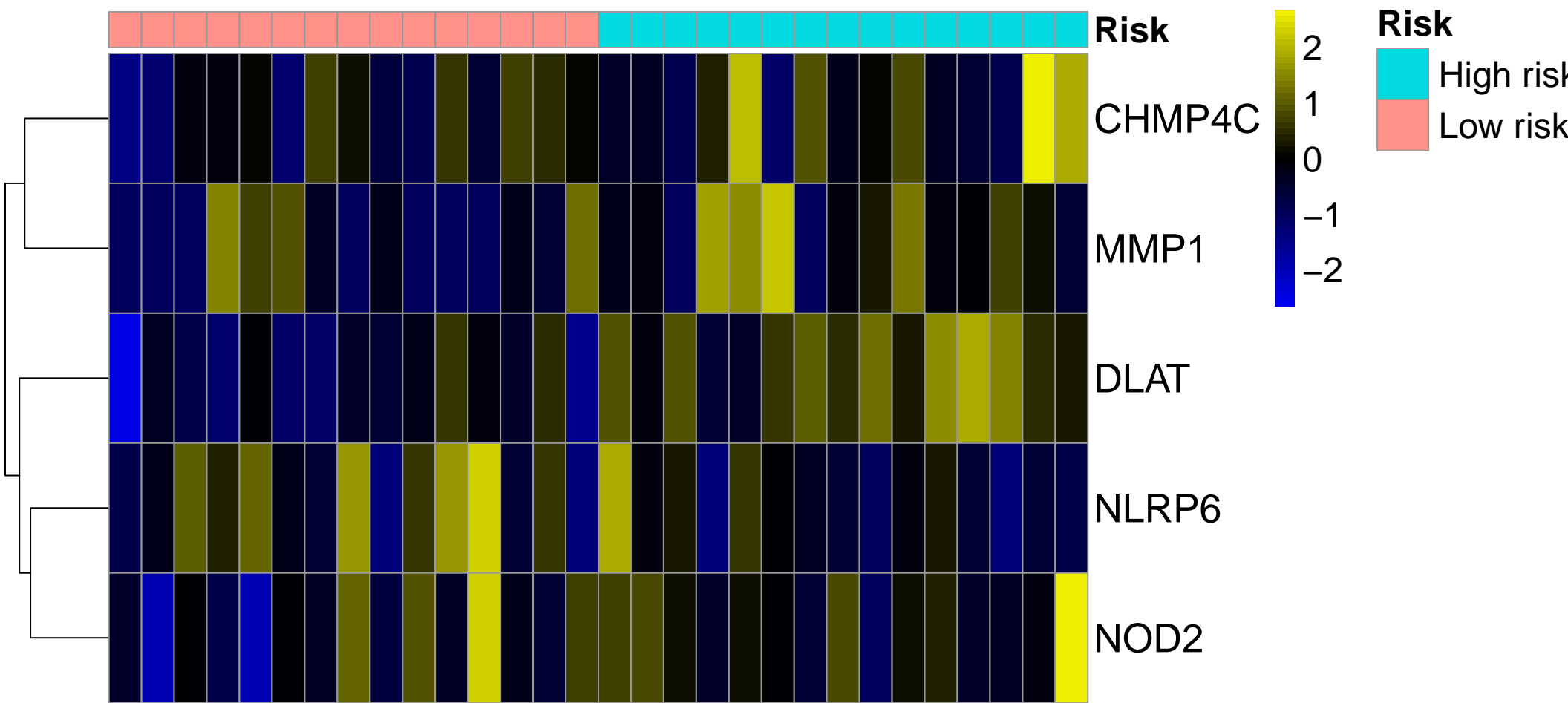

Supplement: Supplementary file 2 [file DataSheet_2.zip › original data 4-6/6-risk/risk-heatmap-test.pdf]

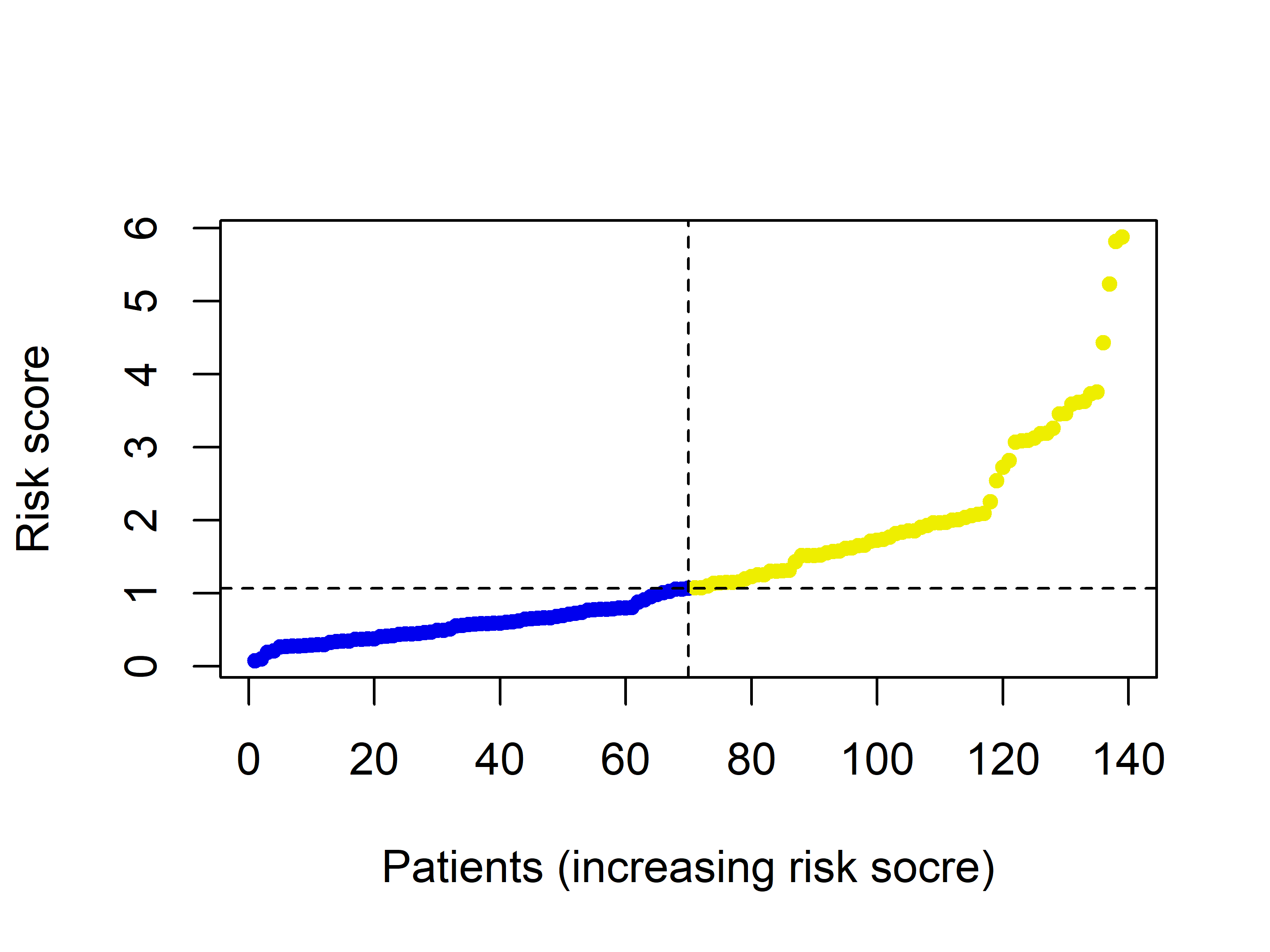

Supplement: Supplementary file 2 [file DataSheet_2.zip › original data 4-6/6-risk/riskScore.tiff]

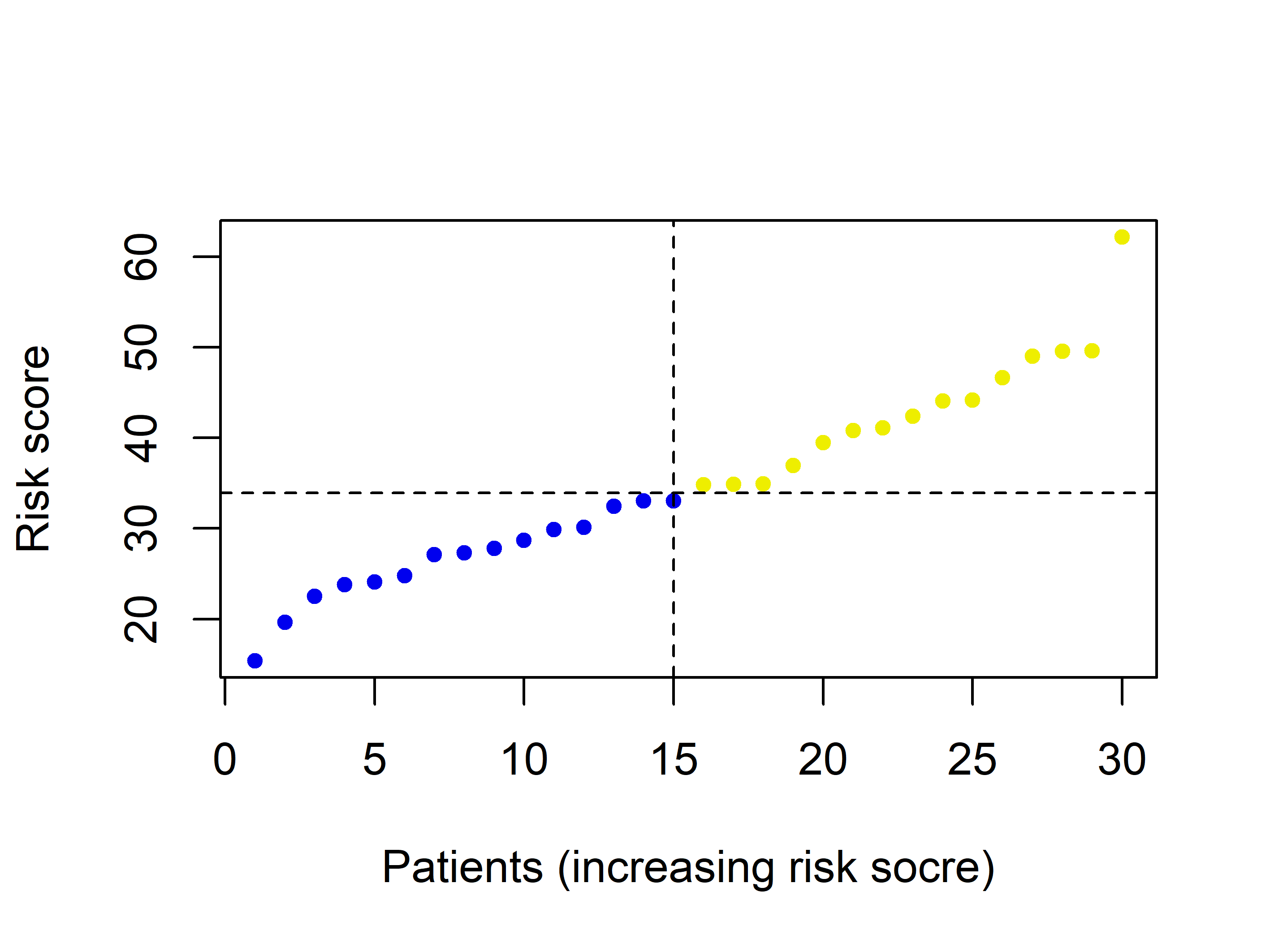

Supplement: Supplementary file 2 [file DataSheet_2.zip › original data 4-6/6-risk/riskScore-test.tiff]

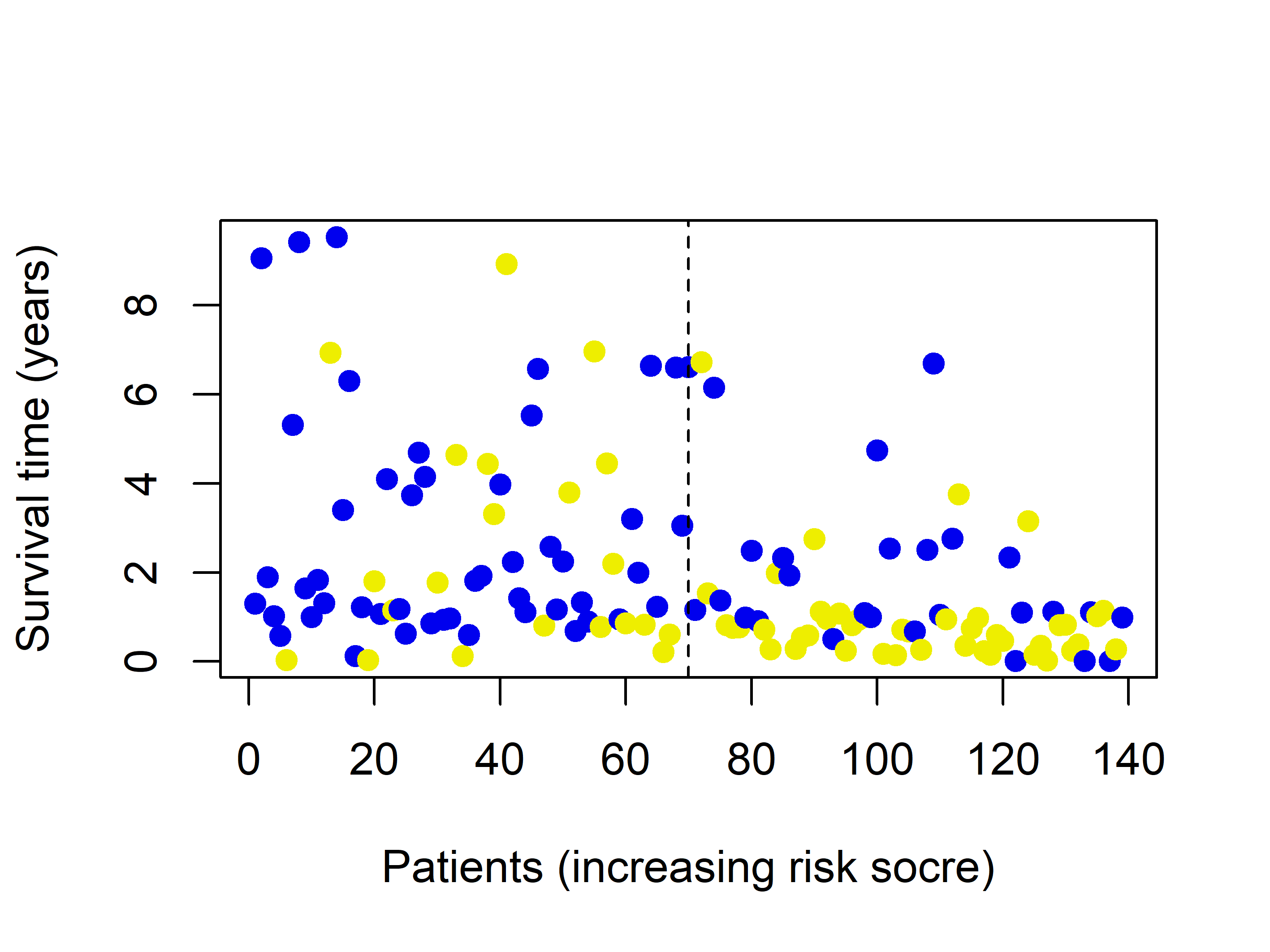

Supplement: Supplementary file 2 [file DataSheet_2.zip › original data 4-6/6-risk/survStat.tiff]

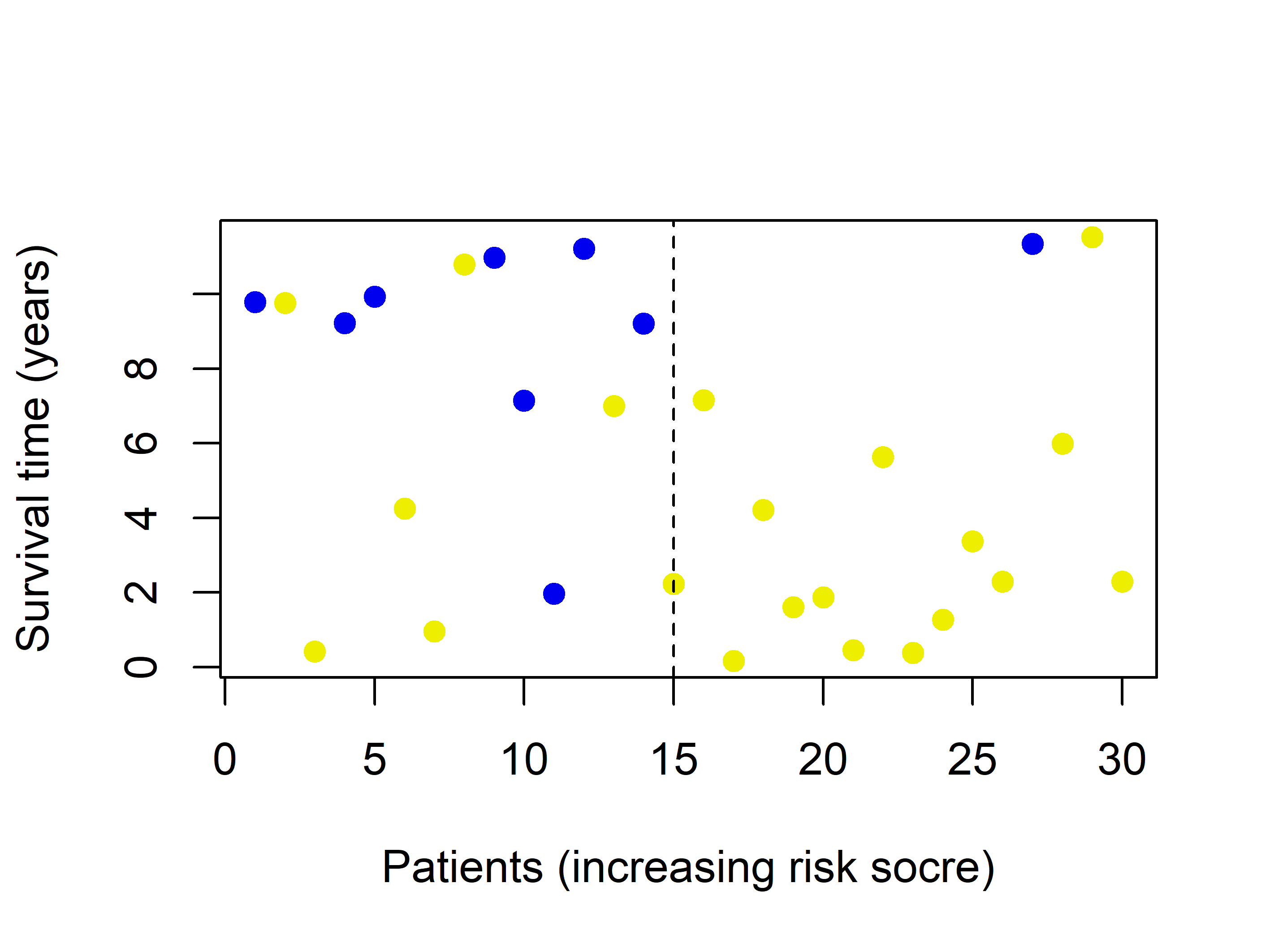

Supplement: Supplementary file 2 [file DataSheet_2.zip › original data 4-6/6-risk/survStat-test.tiff]

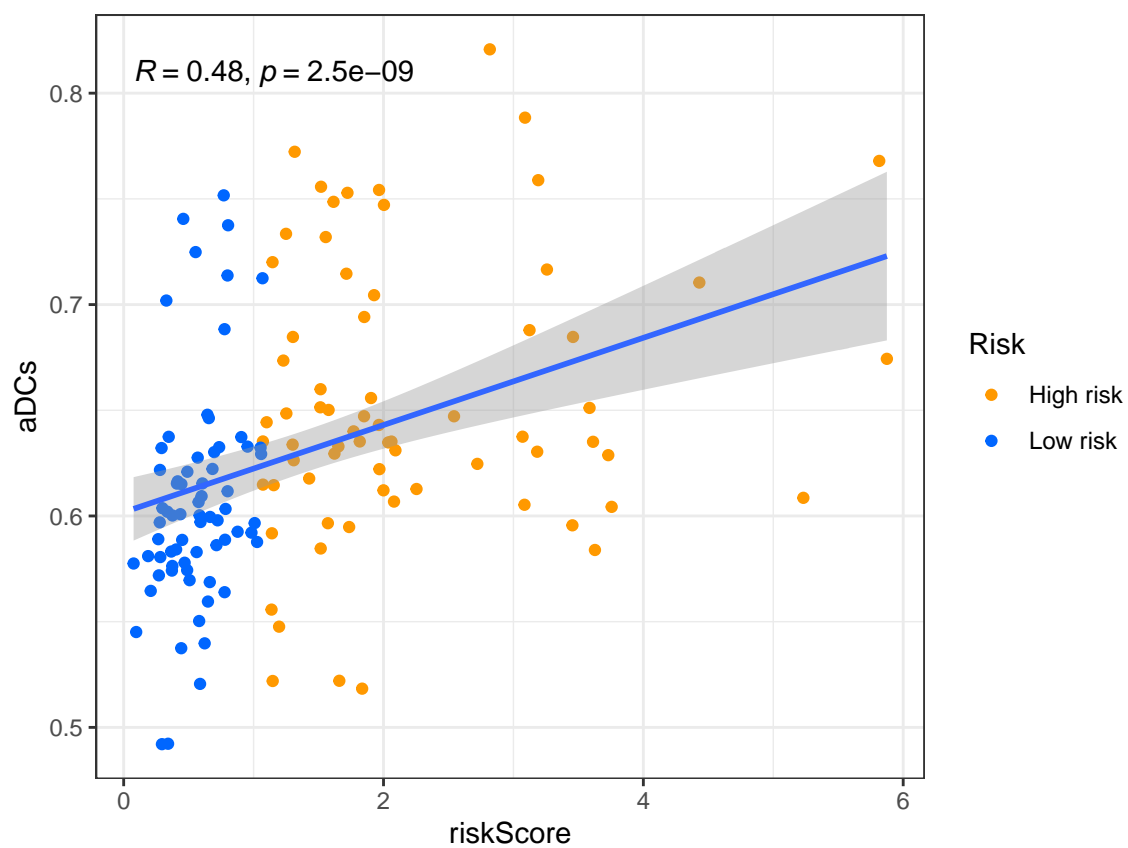

Supplement: Supplementary file 3 [file DataSheet_3.zip › original data 7-9/7-risk immune response/cor-aDCs-sig.pdf]

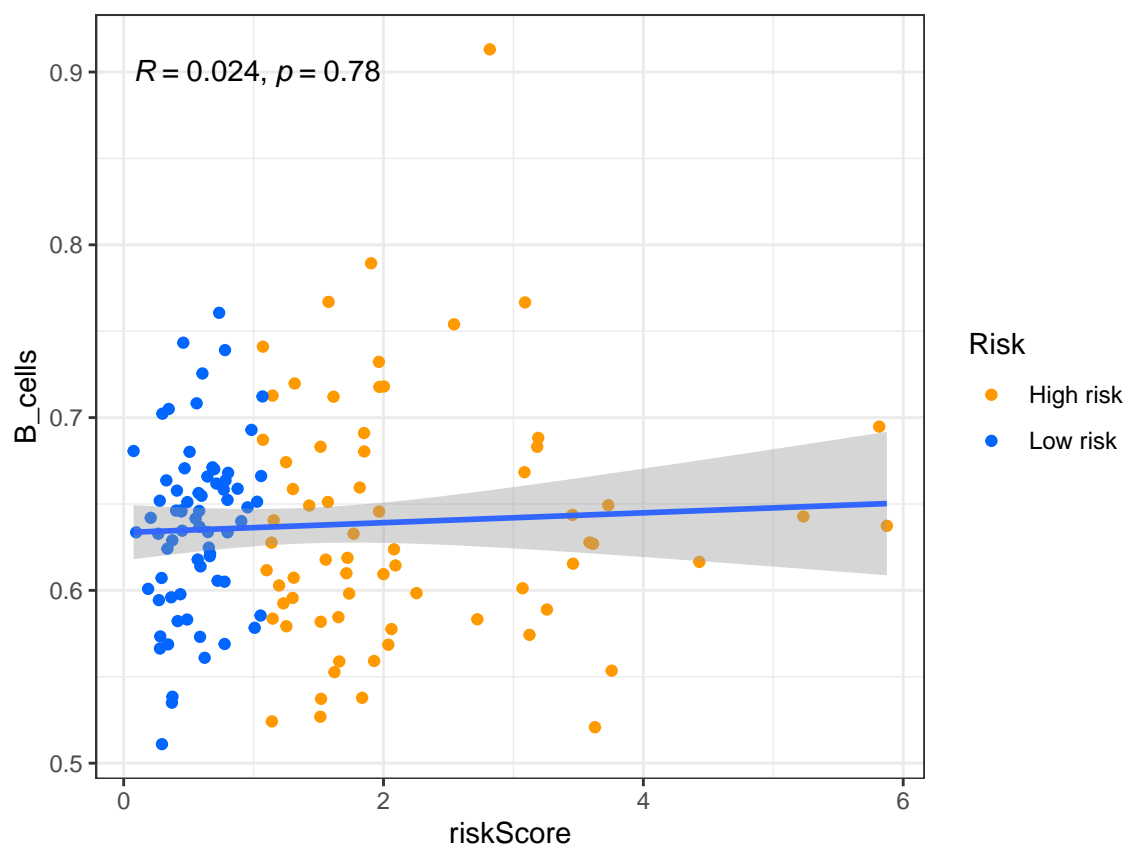

Supplement: Supplementary file 3 [file DataSheet_3.zip › original data 7-9/7-risk immune response/cor-B_cells.pdf]

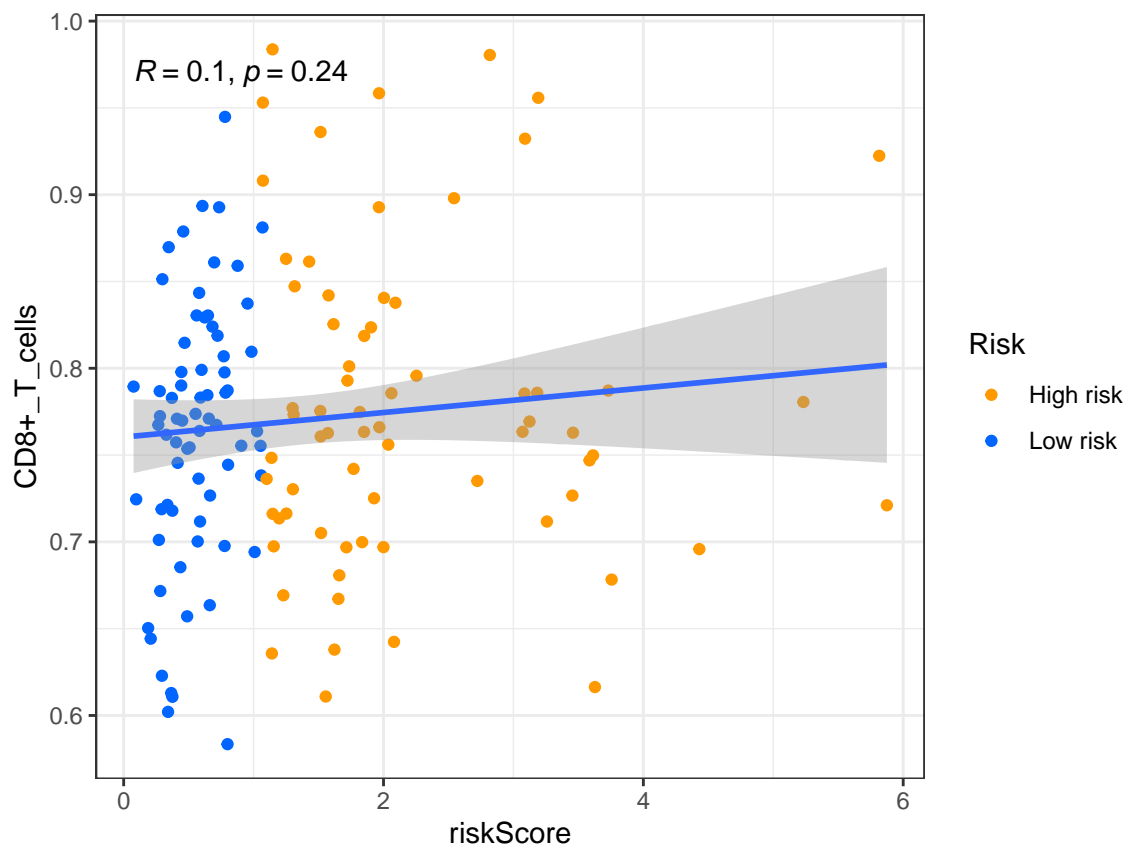

Supplement: Supplementary file 3 [file DataSheet_3.zip › original data 7-9/7-risk immune response/cor-CD8+_T_cells.pdf]

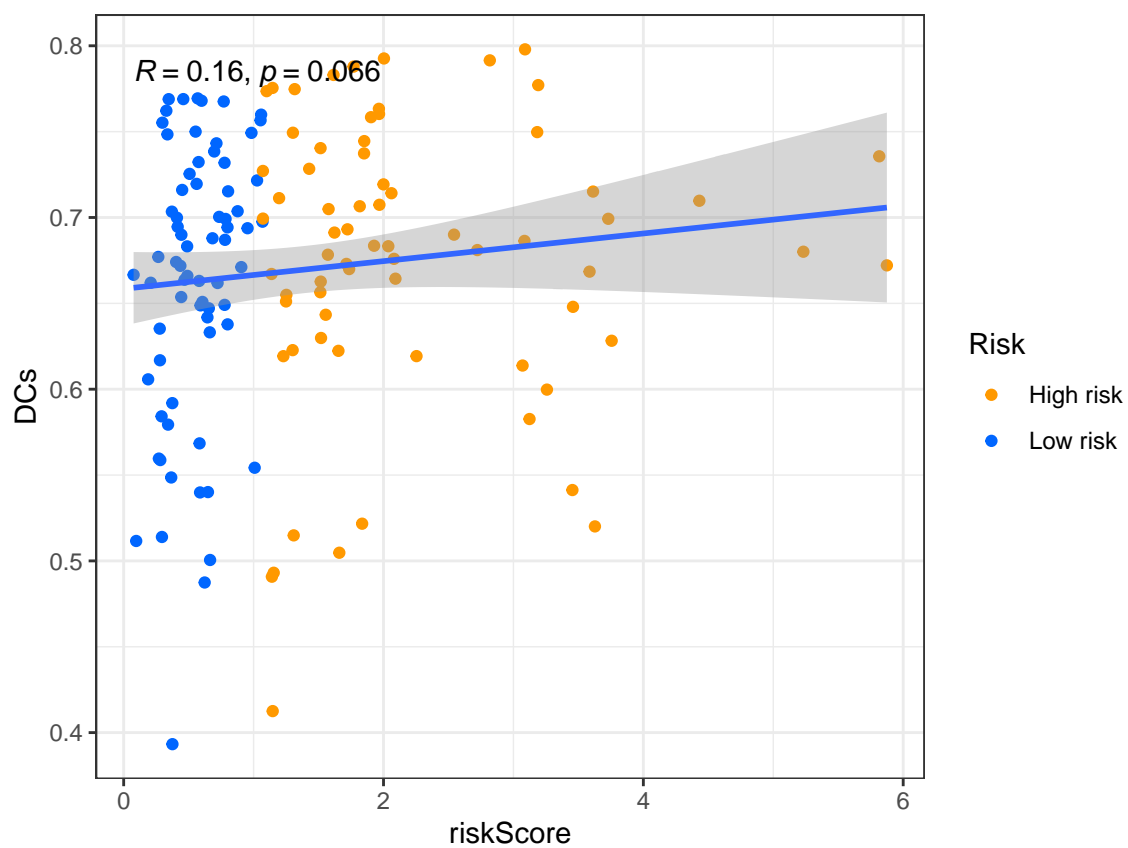

Supplement: Supplementary file 3 [file DataSheet_3.zip › original data 7-9/7-risk immune response/cor-DCs.pdf]

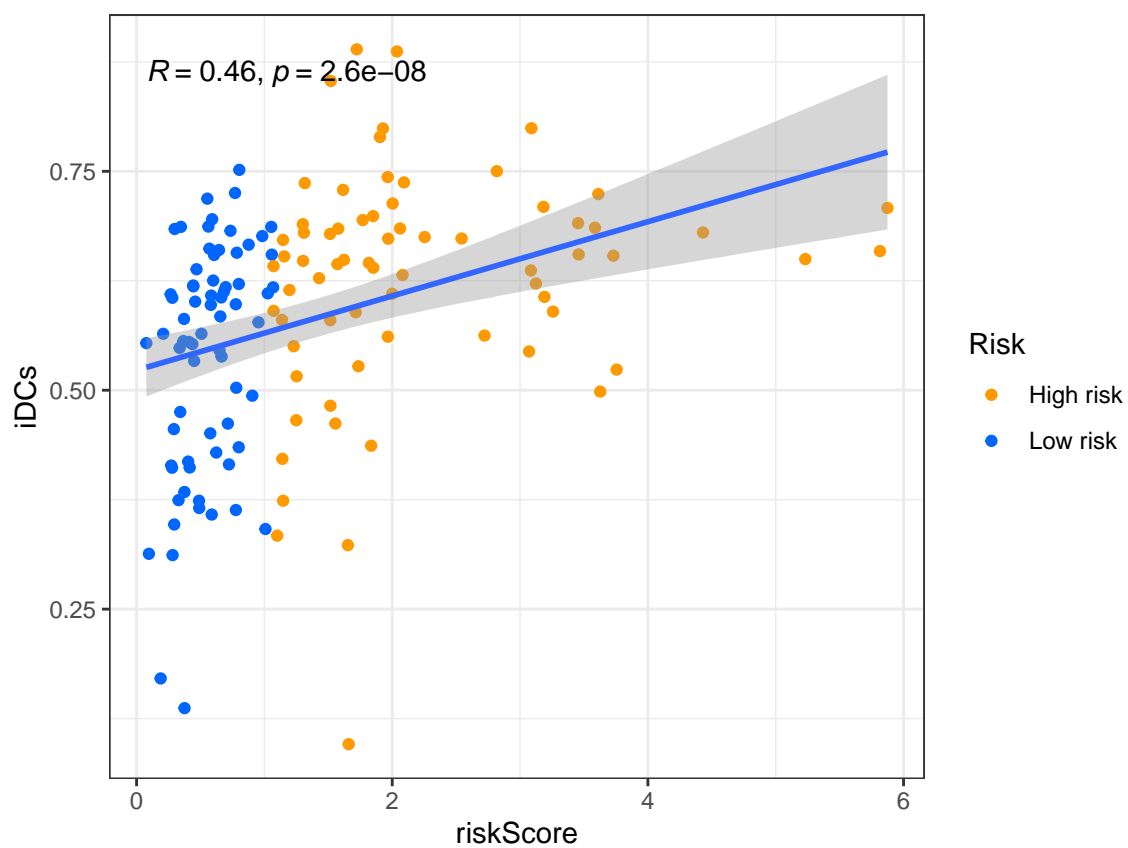

Supplement: Supplementary file 3 [file DataSheet_3.zip › original data 7-9/7-risk immune response/cor-iDCs-sig.pdf]

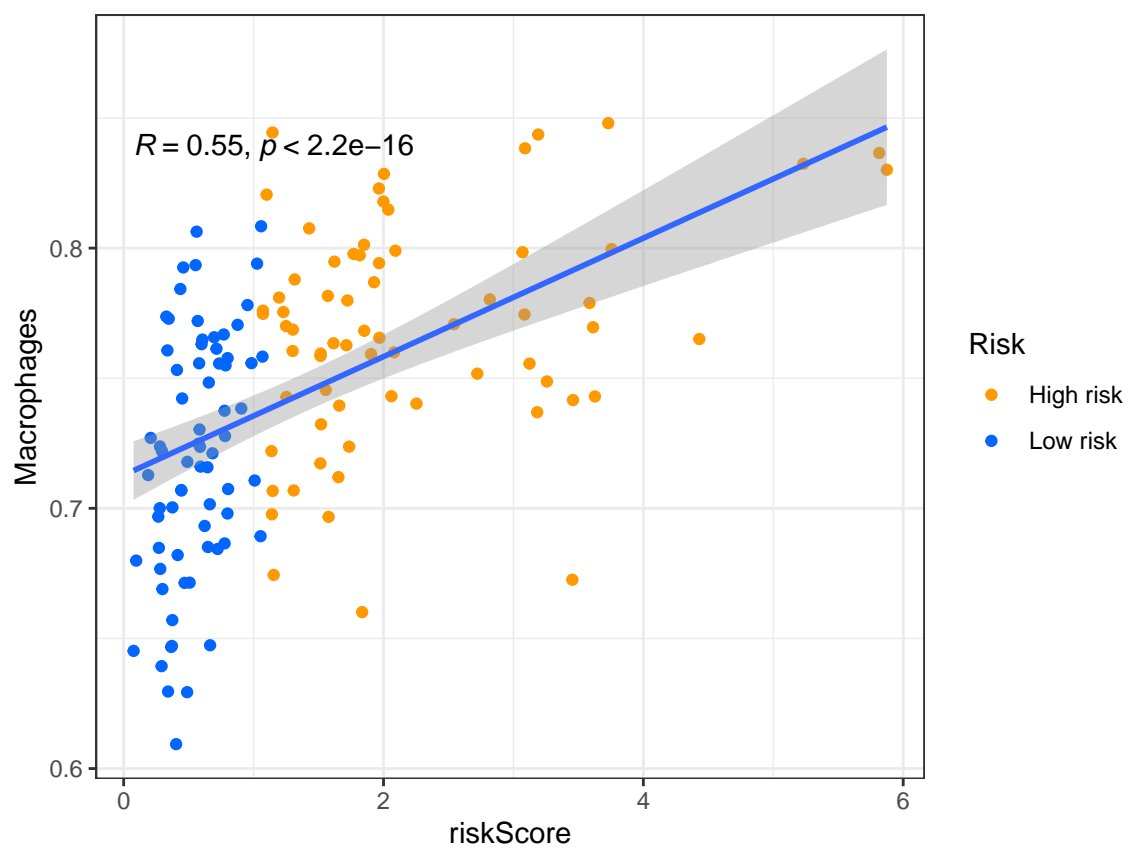

Supplement: Supplementary file 3 [file DataSheet_3.zip › original data 7-9/7-risk immune response/cor-Macrophages-sig.pdf]

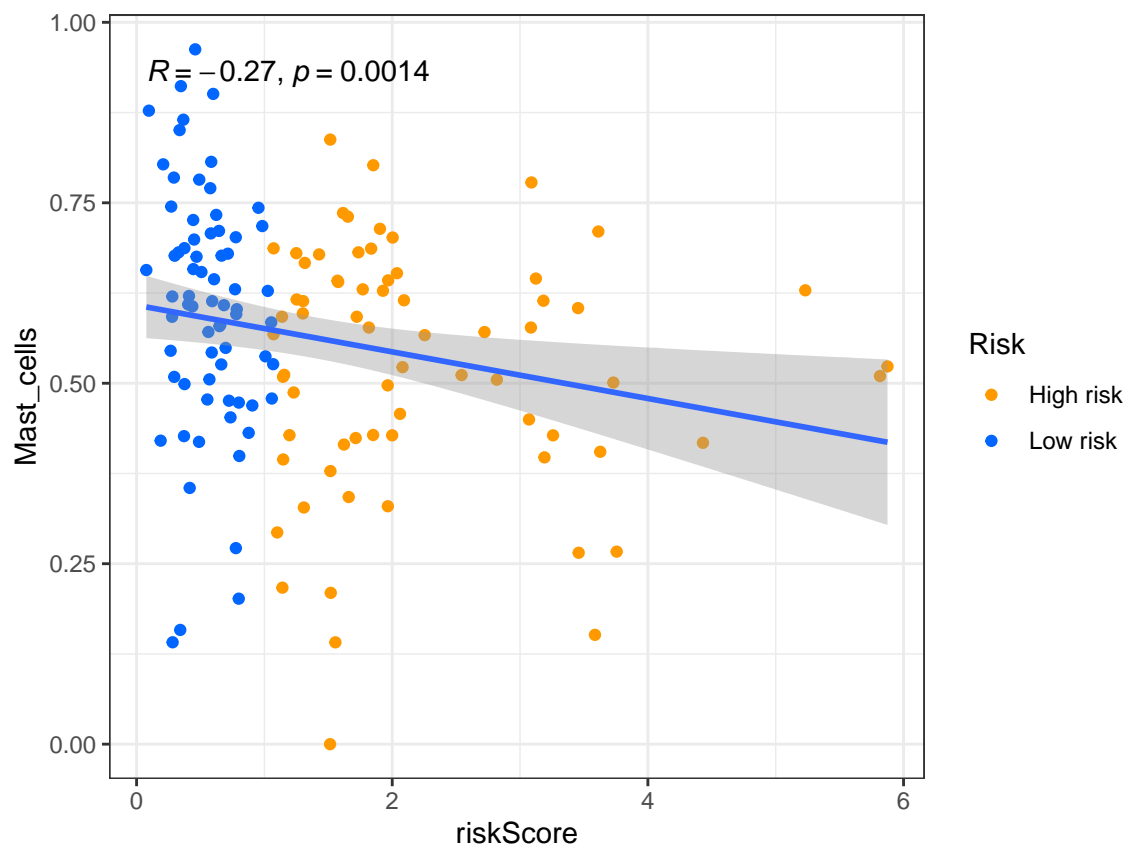

Supplement: Supplementary file 3 [file DataSheet_3.zip › original data 7-9/7-risk immune response/cor-Mast_cells-sig.pdf]

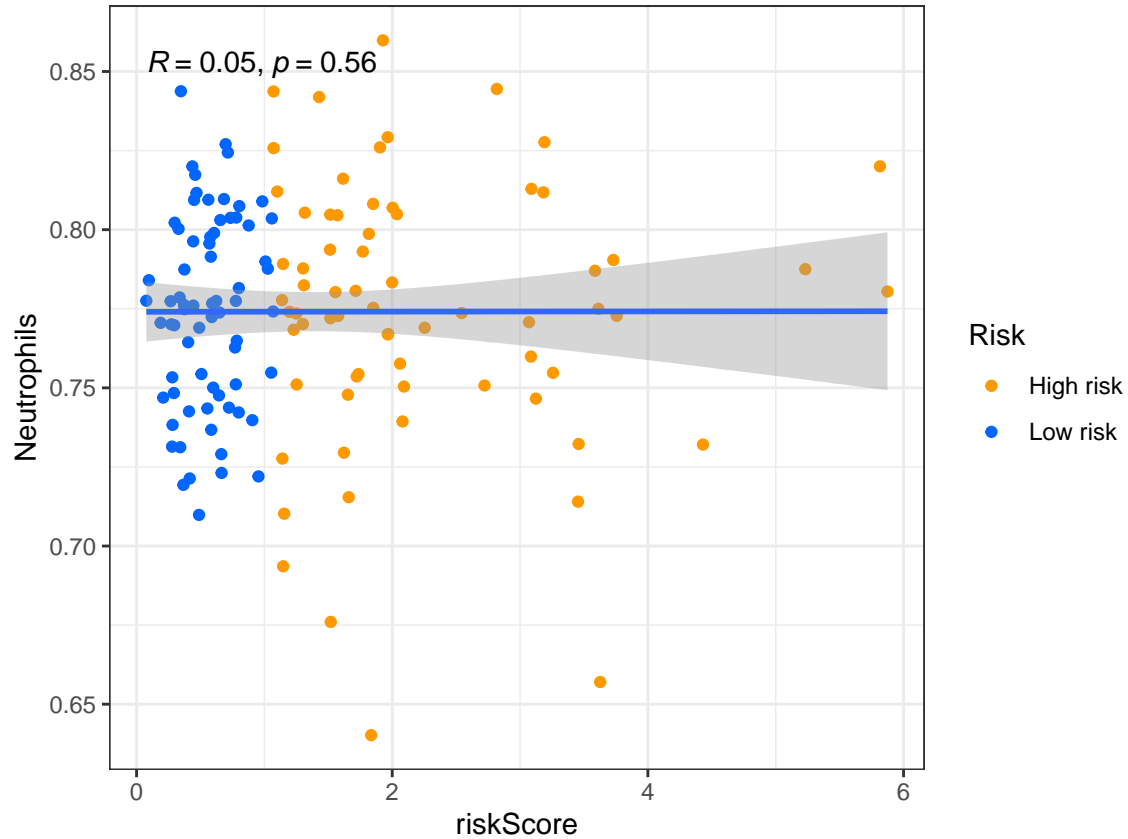

Supplement: Supplementary file 3 [file DataSheet_3.zip › original data 7-9/7-risk immune response/cor-Neutrophils.pdf]

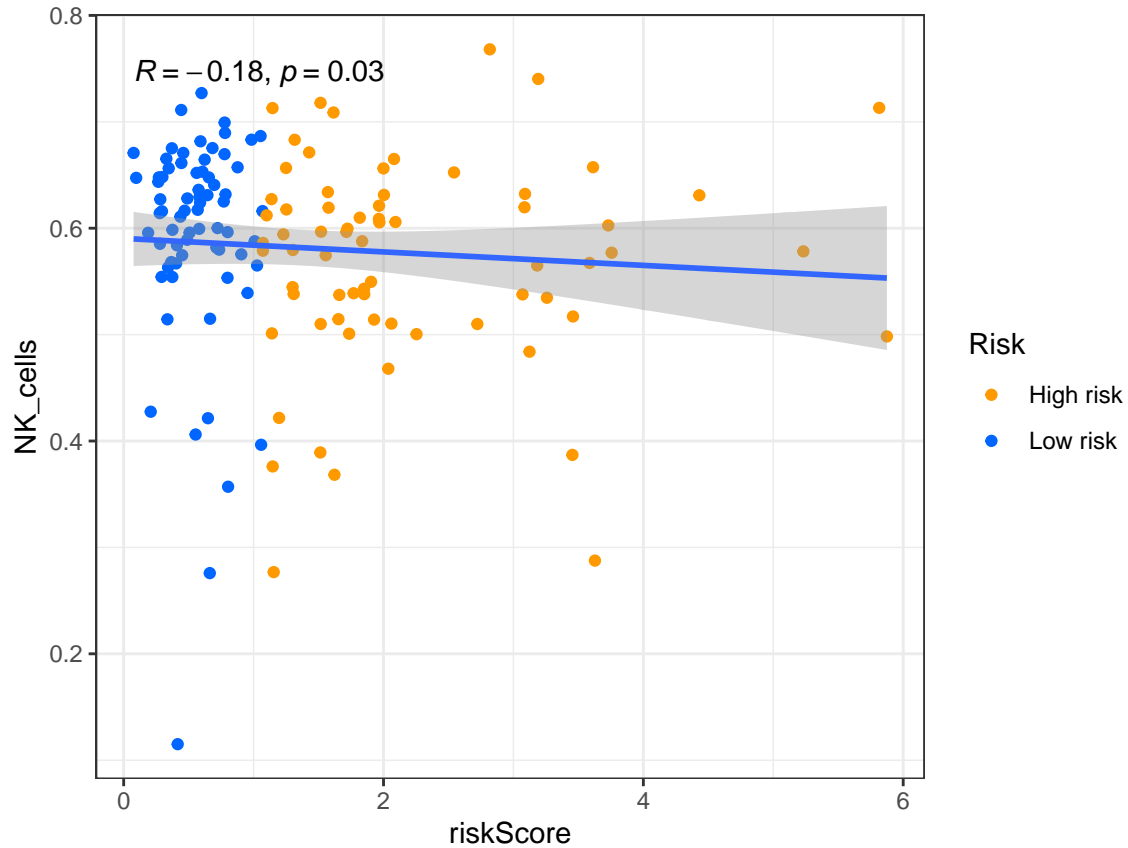

Supplement: Supplementary file 3 [file DataSheet_3.zip › original data 7-9/7-risk immune response/cor-NK_cells-sig.pdf]

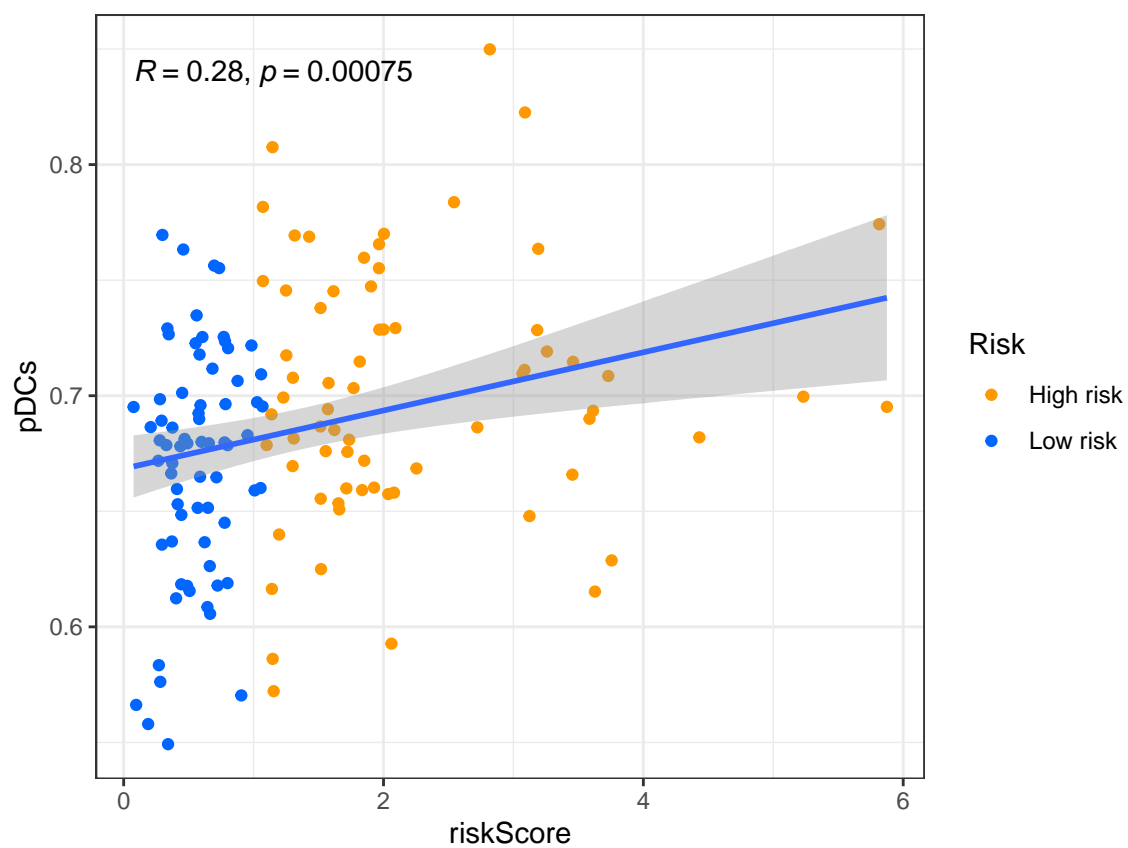

Supplement: Supplementary file 3 [file DataSheet_3.zip › original data 7-9/7-risk immune response/cor-pDCs-sig.pdf]

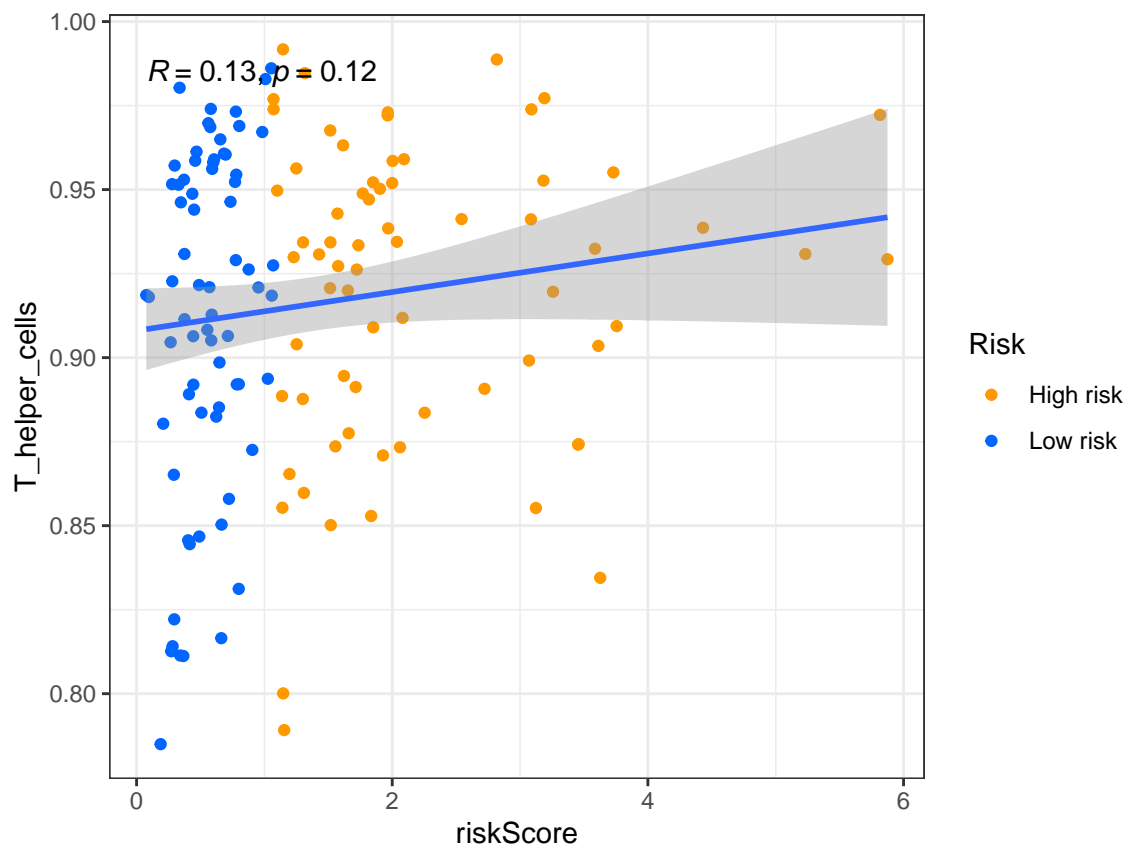

Supplement: Supplementary file 3 [file DataSheet_3.zip › original data 7-9/7-risk immune response/cor-T_helper_cells.pdf]

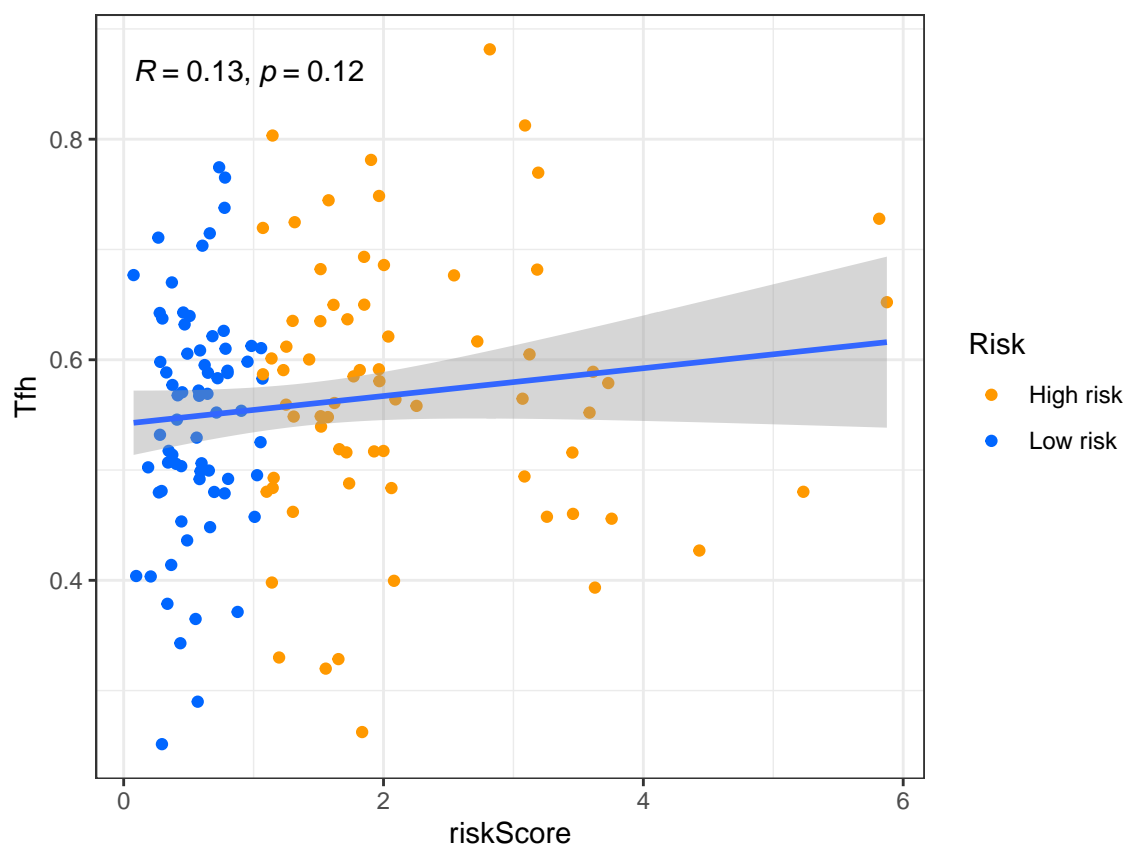

Supplement: Supplementary file 3 [file DataSheet_3.zip › original data 7-9/7-risk immune response/cor-Tfh.pdf]

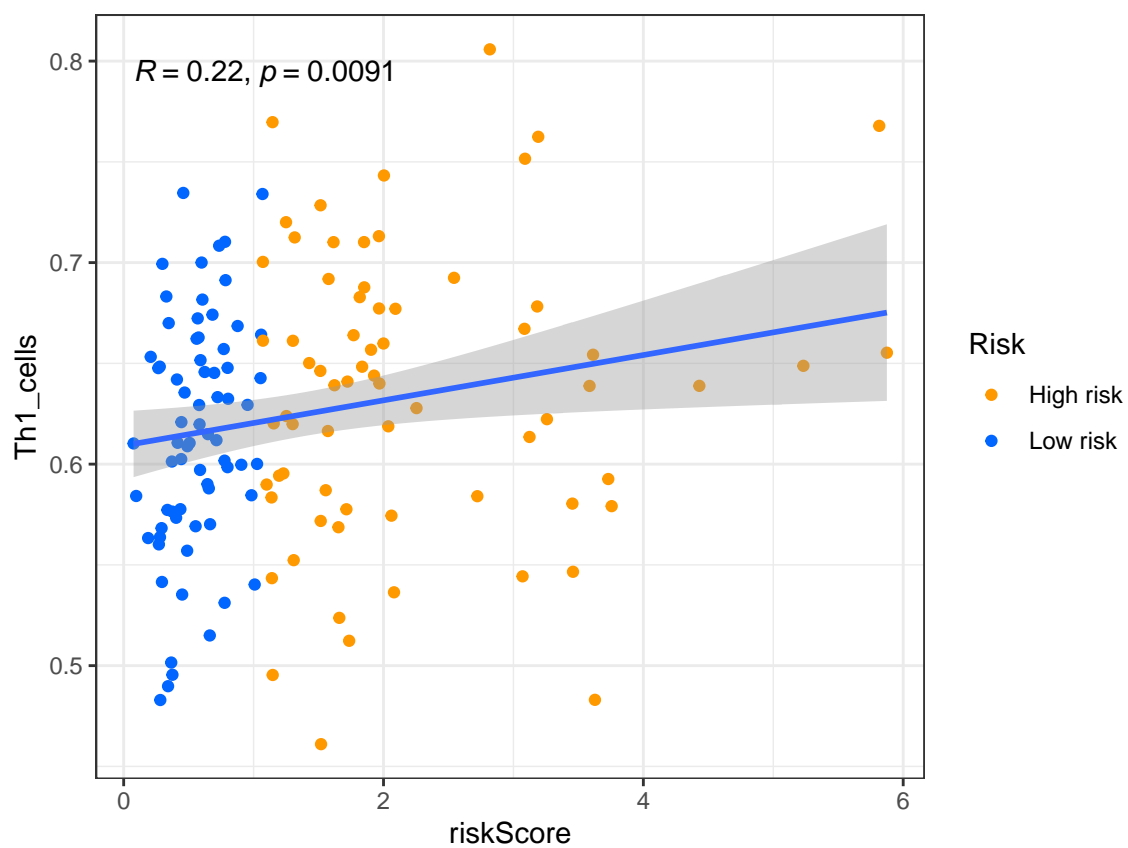

Supplement: Supplementary file 3 [file DataSheet_3.zip › original data 7-9/7-risk immune response/cor-Th1_cells-sig.pdf]

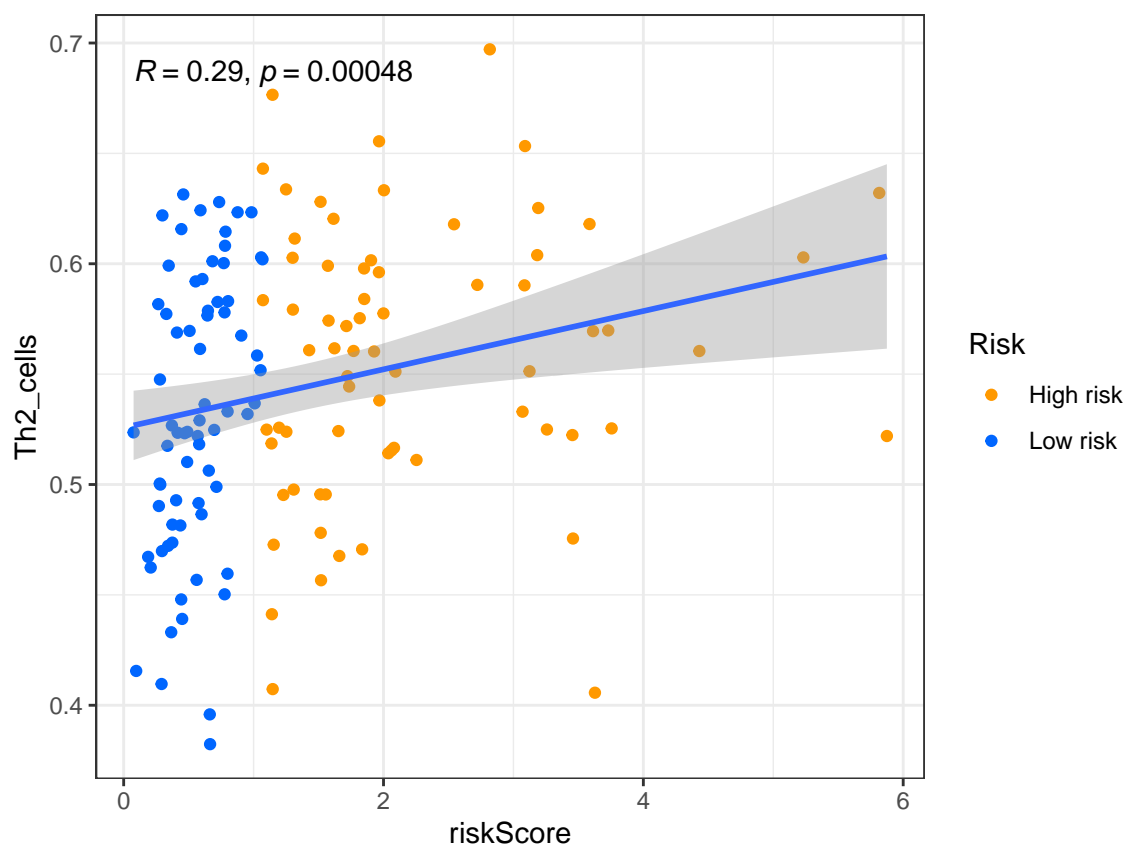

Supplement: Supplementary file 3 [file DataSheet_3.zip › original data 7-9/7-risk immune response/cor-Th2_cells-sig.pdf]

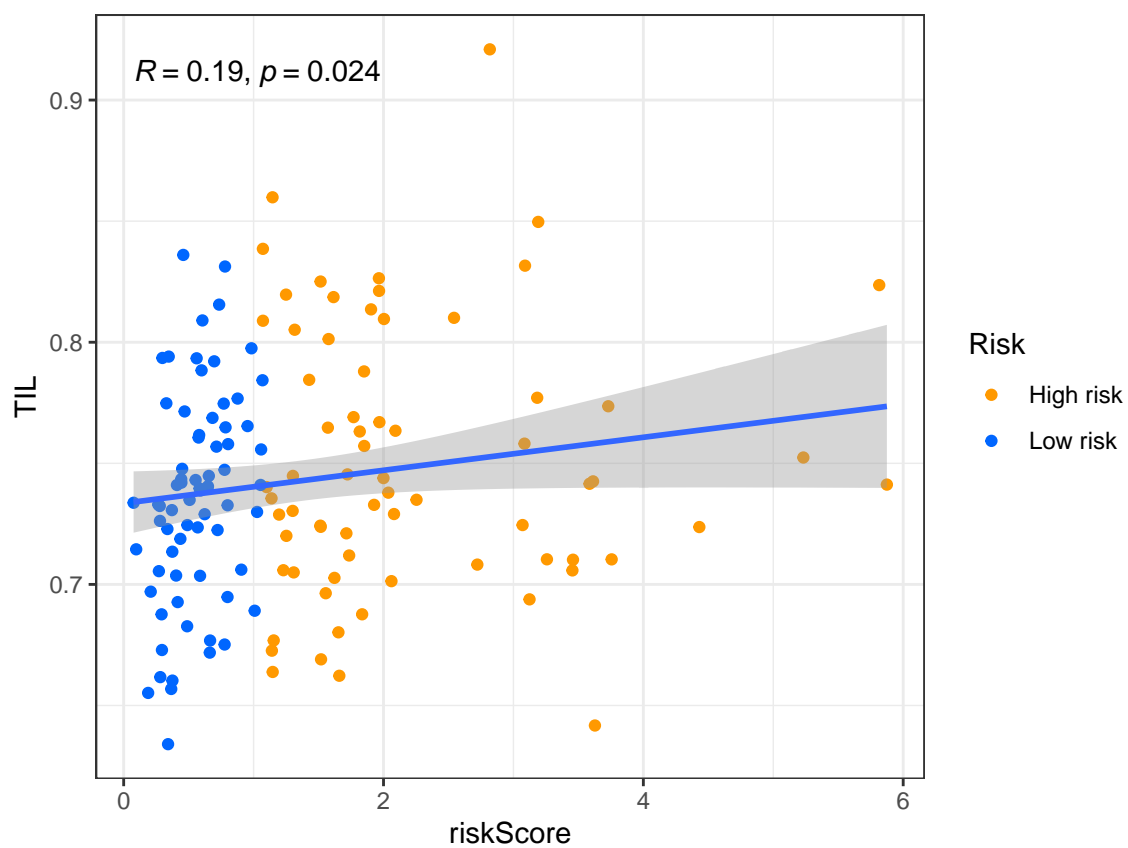

Supplement: Supplementary file 3 [file DataSheet_3.zip › original data 7-9/7-risk immune response/cor-TIL-sig.pdf]

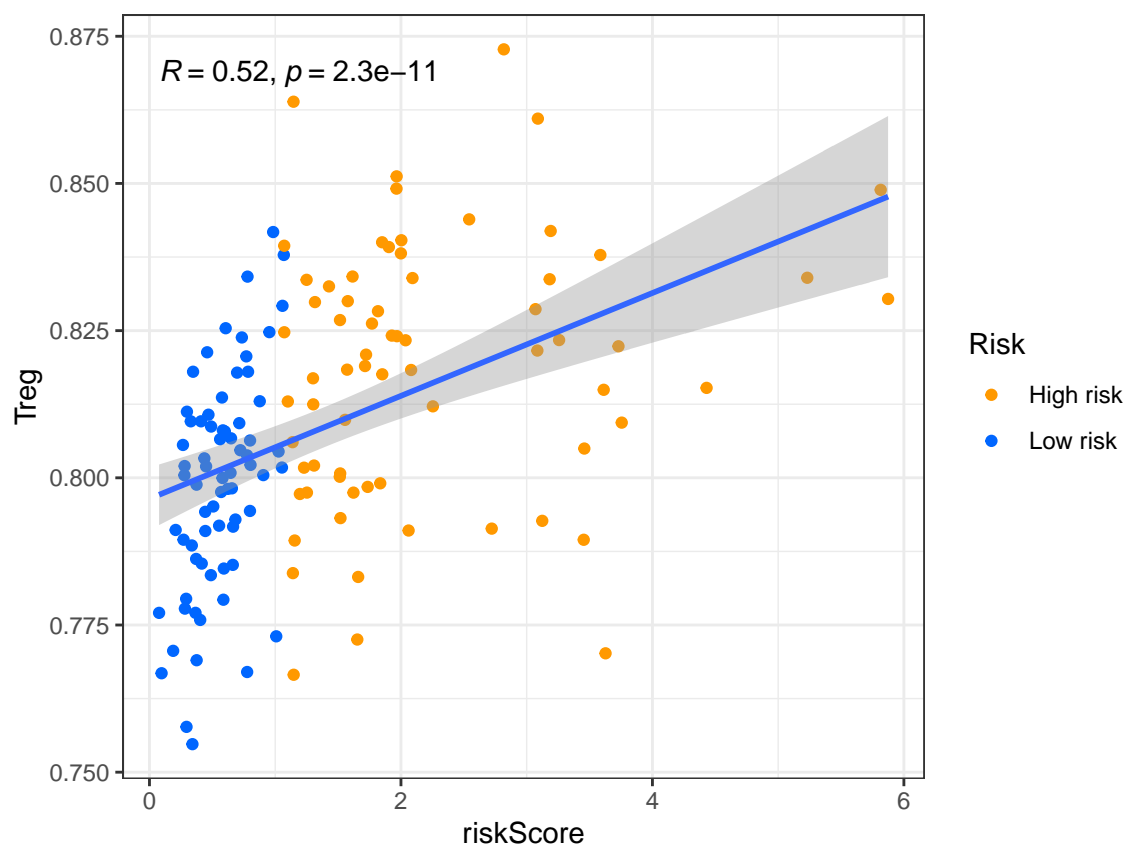

Supplement: Supplementary file 3 [file DataSheet_3.zip › original data 7-9/7-risk immune response/cor-Treg-sig.pdf]

Risk   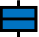 Low risk   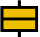 High risk

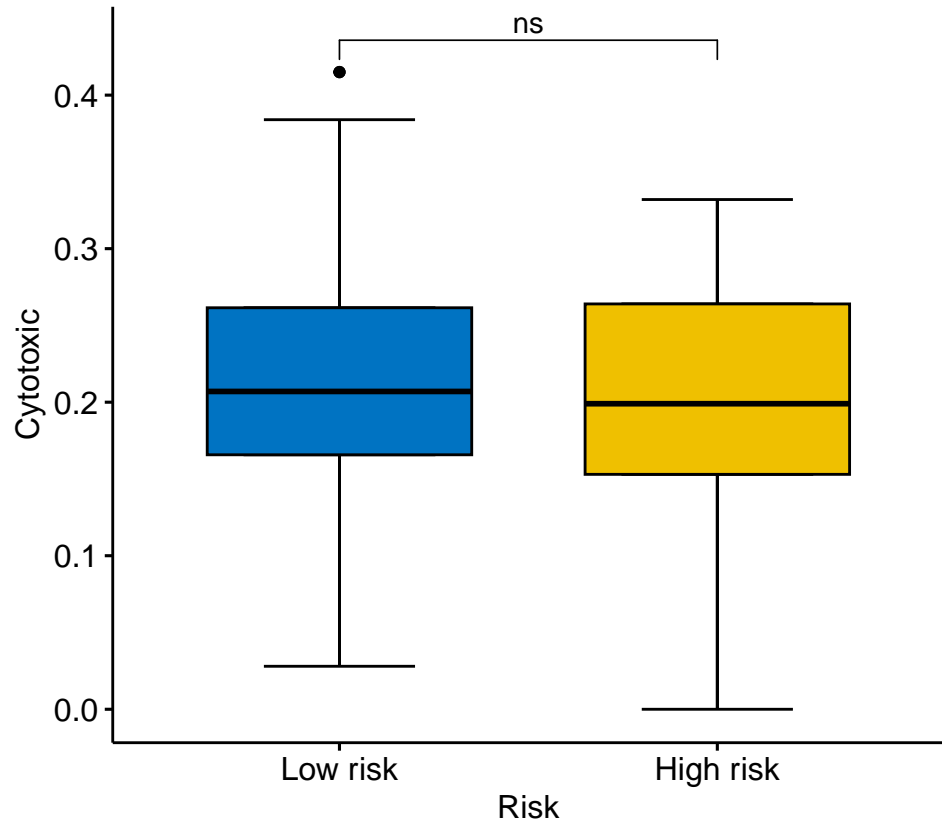

Supplement: Supplementary file 3 [file DataSheet_3.zip › original data 7-9/7-risk immune response/Cytotoxic.pdf]

Risk 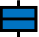 Low risk 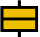 High risk

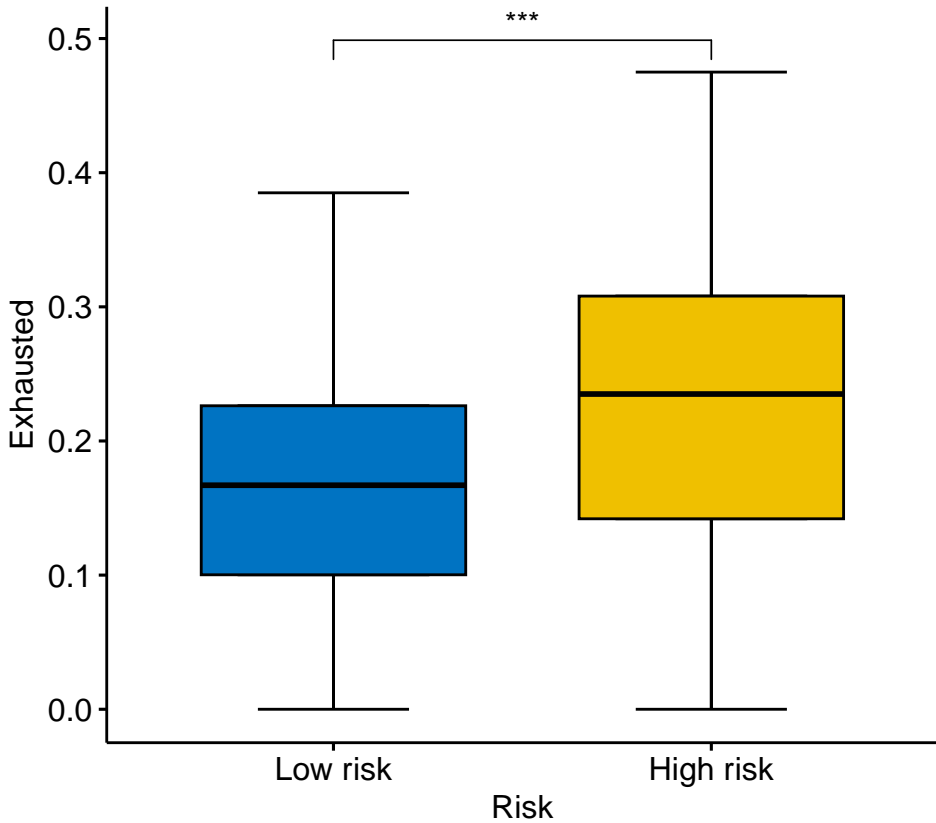

Supplement: Supplementary file 3 [file DataSheet_3.zip › original data 7-9/7-risk immune response/Exhausted.pdf]

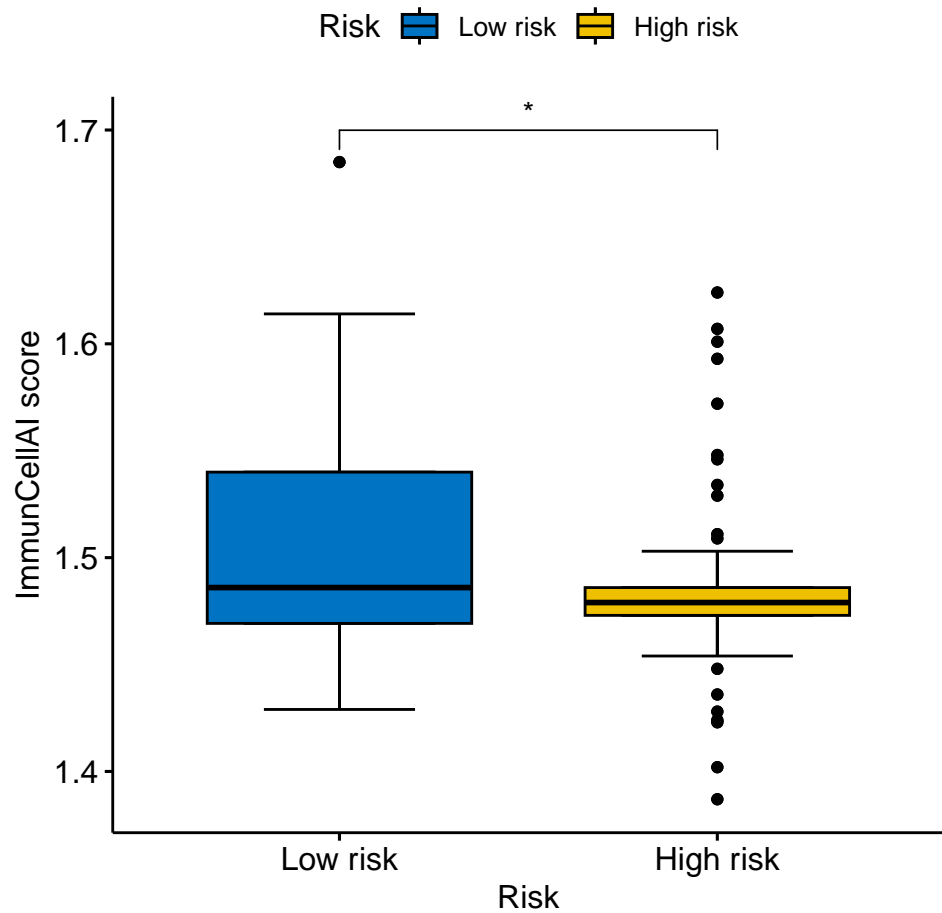

Supplement: Supplementary file 3 [file DataSheet_3.zip › original data 7-9/7-risk immune response/ImmunCellAI score.pdf]

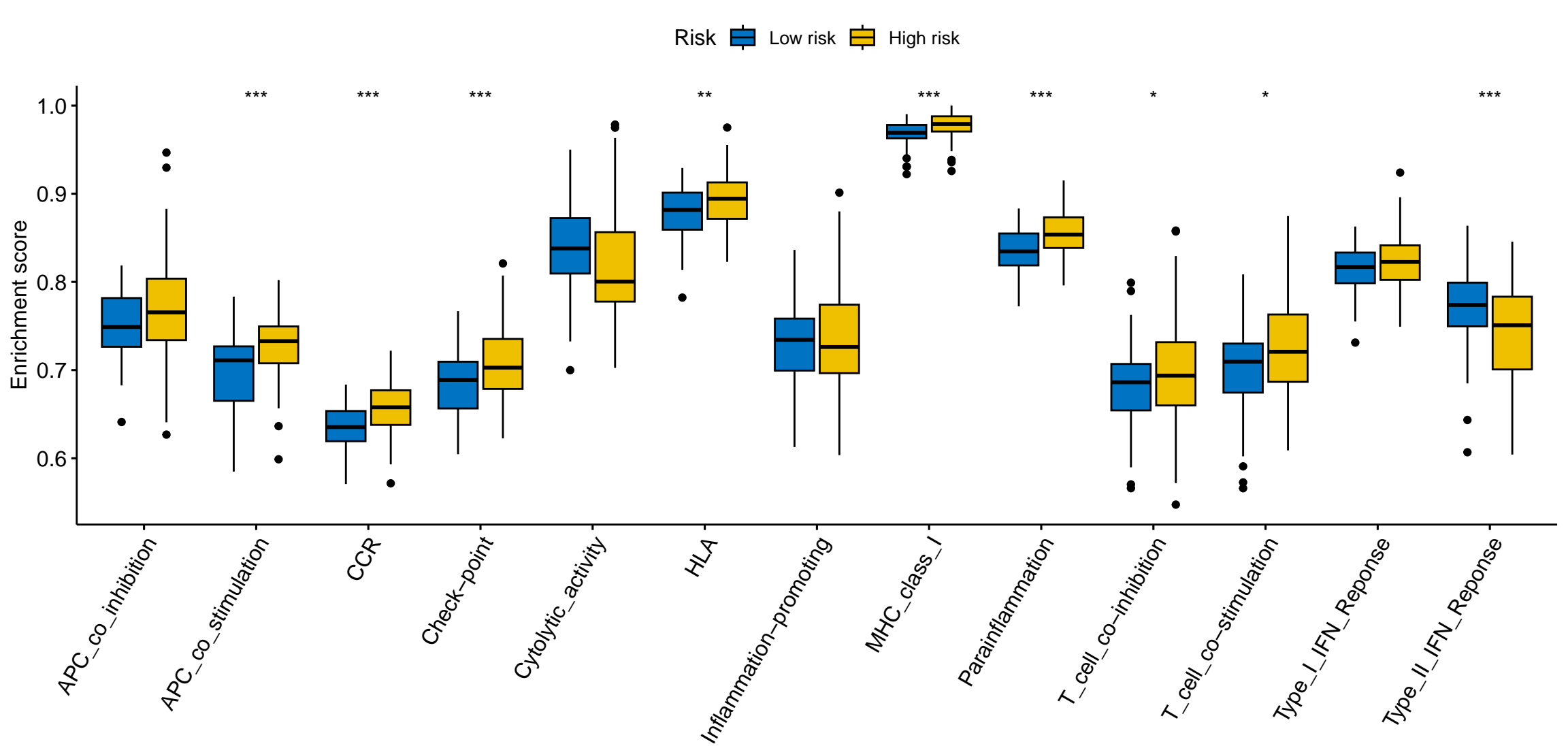

Supplement: Supplementary file 3 [file DataSheet_3.zip › original data 7-9/7-risk immune response/immune process.pdf]

Risk 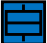 Low risk 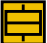 High risk

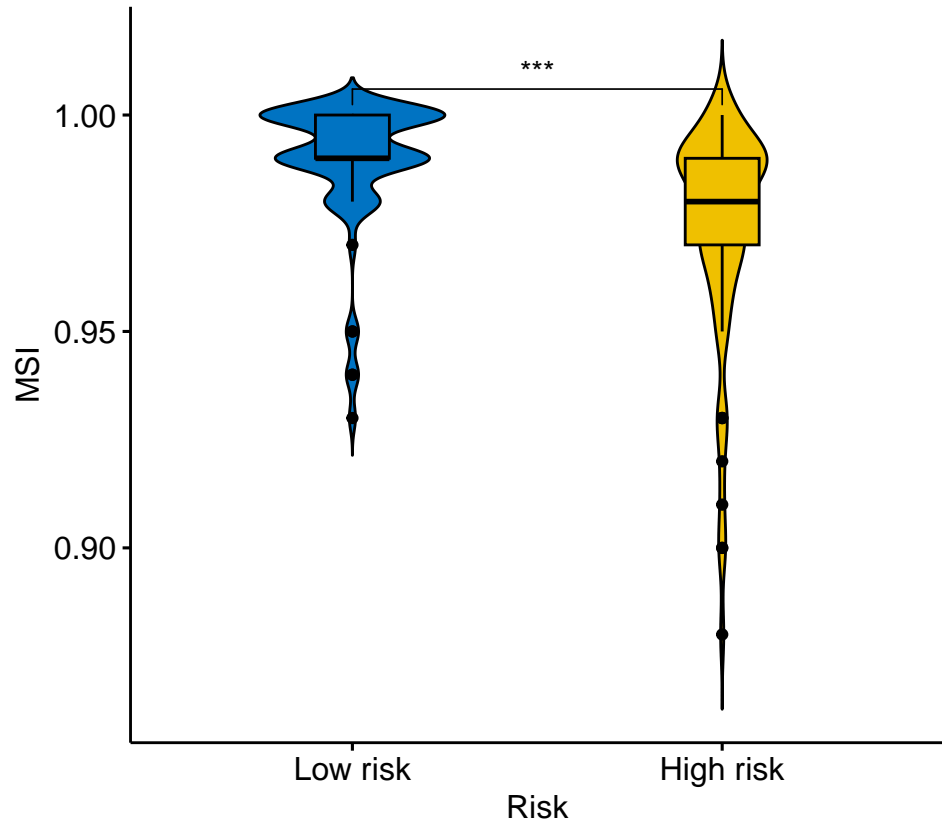

Supplement: Supplementary file 3 [file DataSheet_3.zip › original data 7-9/7-risk immune response/MSI.pdf]

Risk   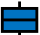 Low risk   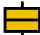 High risk

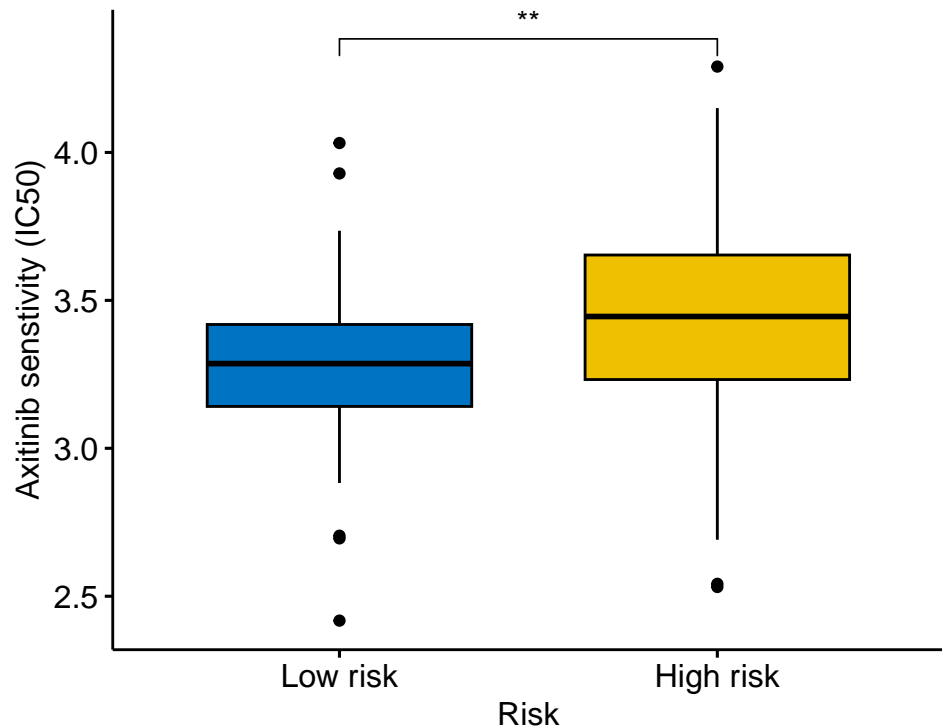

Supplement: Supplementary file 3 [file DataSheet_3.zip › original data 7-9/8-drug sensitivity/durgSenstivity.Axitinib.pdf]

Risk 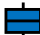 Low risk 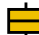 High risk

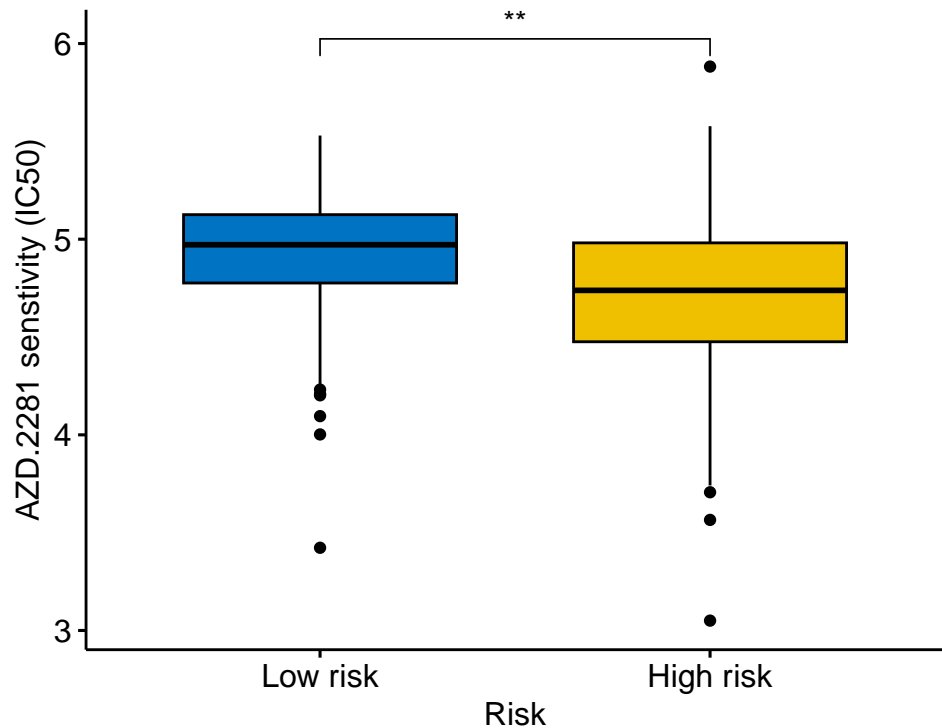

Supplement: Supplementary file 3 [file DataSheet_3.zip › original data 7-9/8-drug sensitivity/durgSenstivity.AZD.2281.pdf]

Risk Low risk High risk

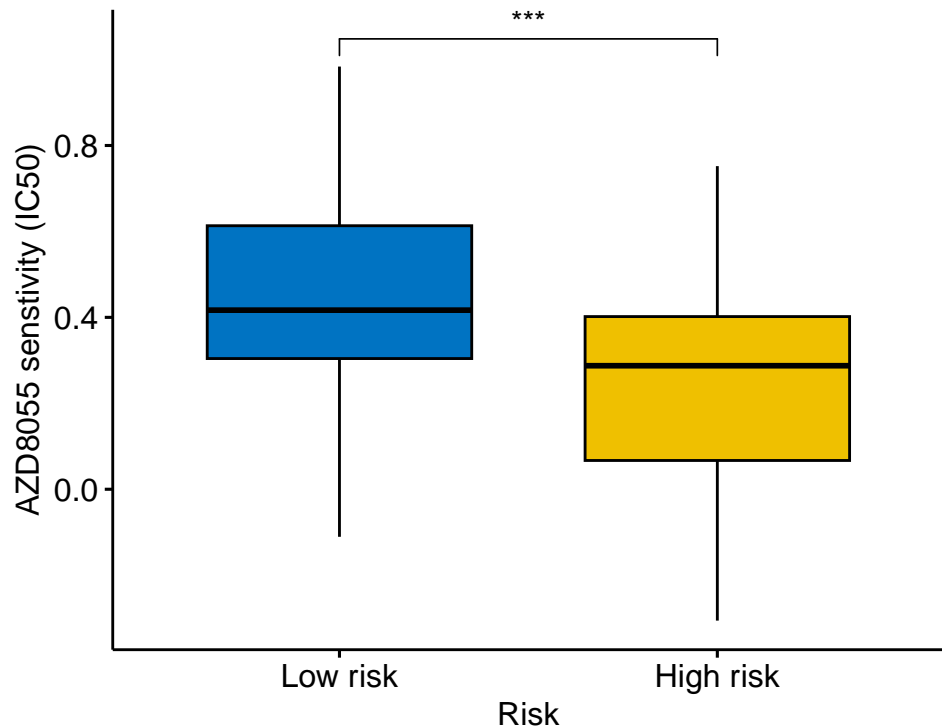

Supplement: Supplementary file 3 [file DataSheet_3.zip › original data 7-9/8-drug sensitivity/durgSenstivity.AZD8055.pdf]

Risk 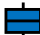 Low risk 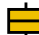 High risk

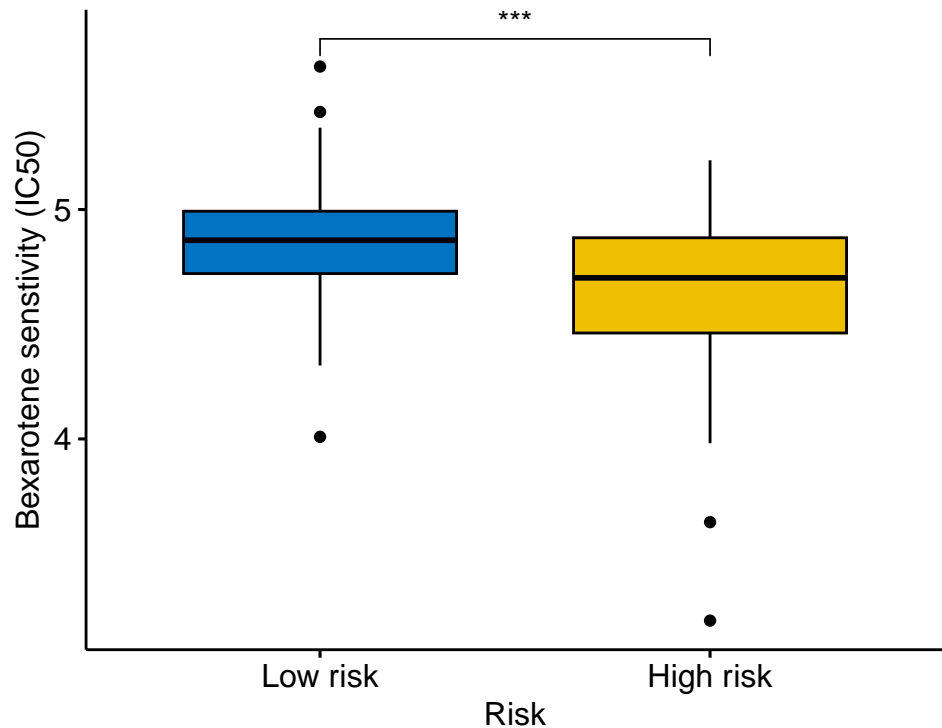

Supplement: Supplementary file 3 [file DataSheet_3.zip › original data 7-9/8-drug sensitivity/durgSenstivity.Bexarotene.pdf]

Risk 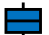 Low risk 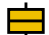 High risk

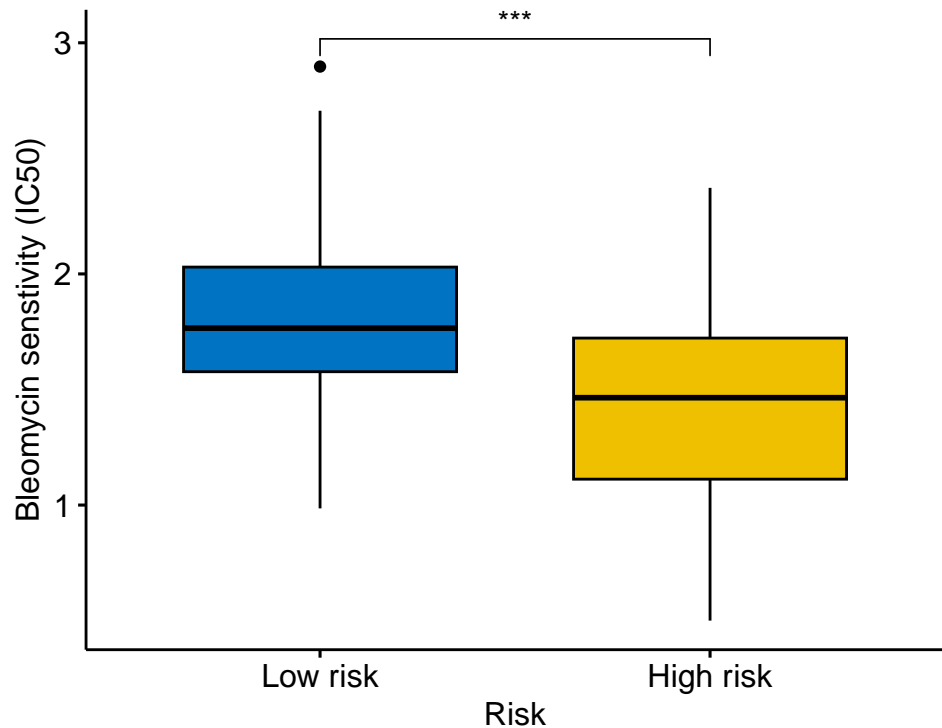

Supplement: Supplementary file 3 [file DataSheet_3.zip › original data 7-9/8-drug sensitivity/durgSenstivity.Bleomycin.pdf]

Risk   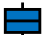 Low risk   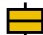 High risk

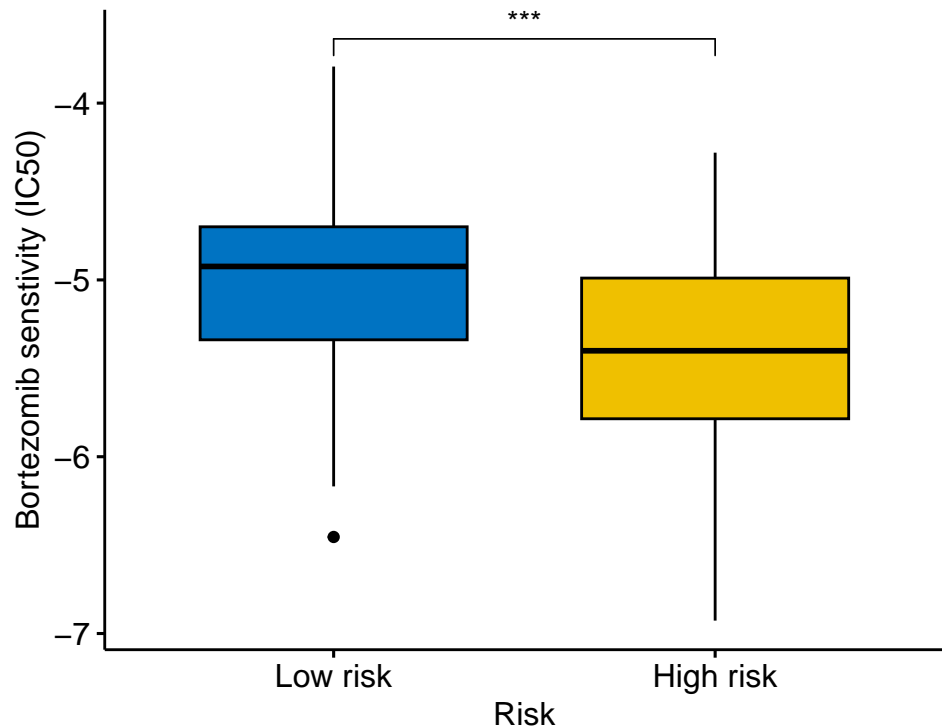

Supplement: Supplementary file 3 [file DataSheet_3.zip › original data 7-9/8-drug sensitivity/durgSenstivity.Bortezomib.pdf]

Risk Low risk High risk

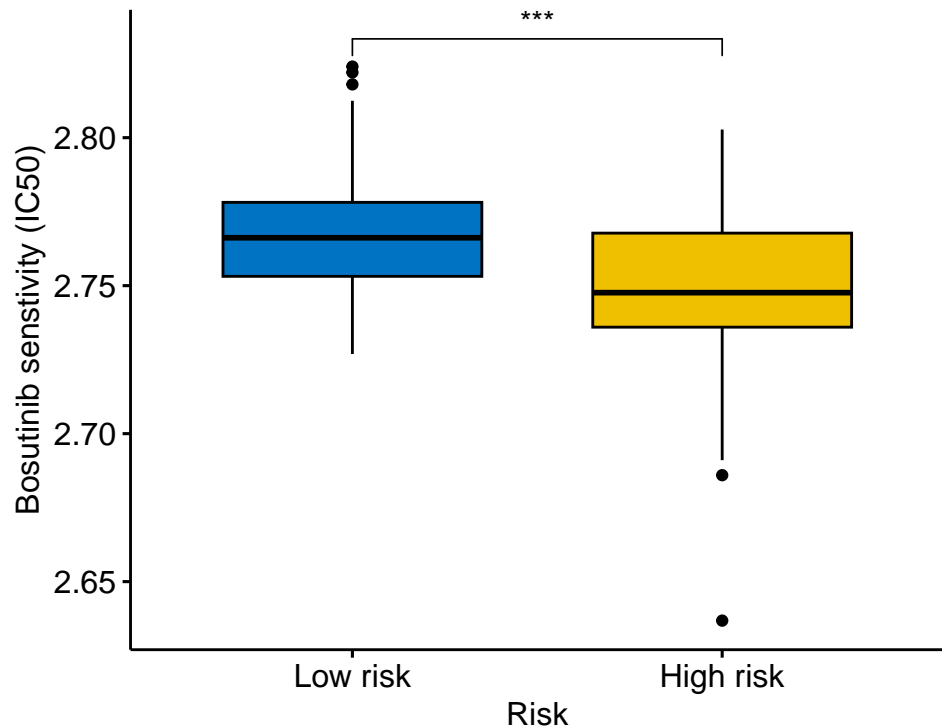

Supplement: Supplementary file 3 [file DataSheet_3.zip › original data 7-9/8-drug sensitivity/durgSenstivity.Bosutinib.pdf]

Risk Low risk High risk

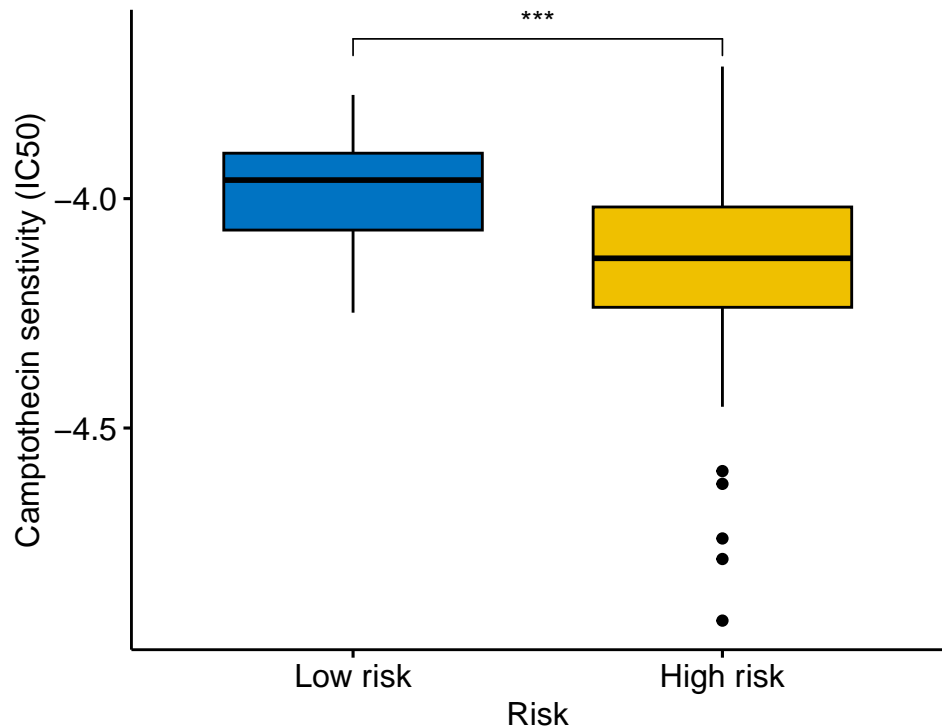

Supplement: Supplementary file 3 [file DataSheet_3.zip › original data 7-9/8-drug sensitivity/durgSenstivity.Camptothecin.pdf]

Risk 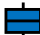 Low risk 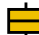 High risk

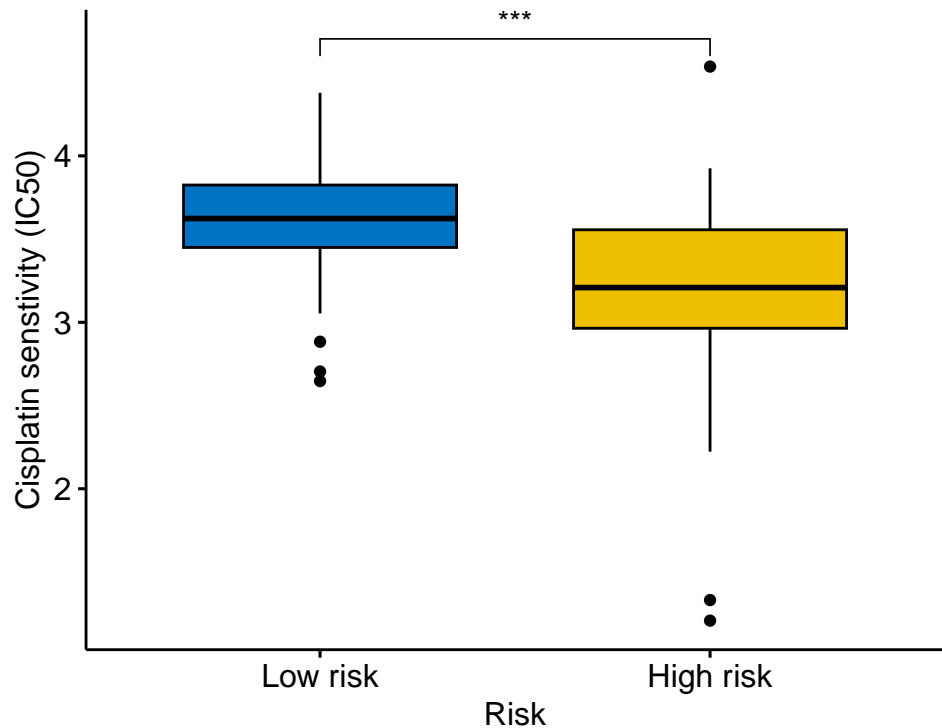

Supplement: Supplementary file 3 [file DataSheet_3.zip › original data 7-9/8-drug sensitivity/durgSenstivity.Cisplatin.pdf]

Risk   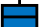 Low risk   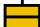 High risk

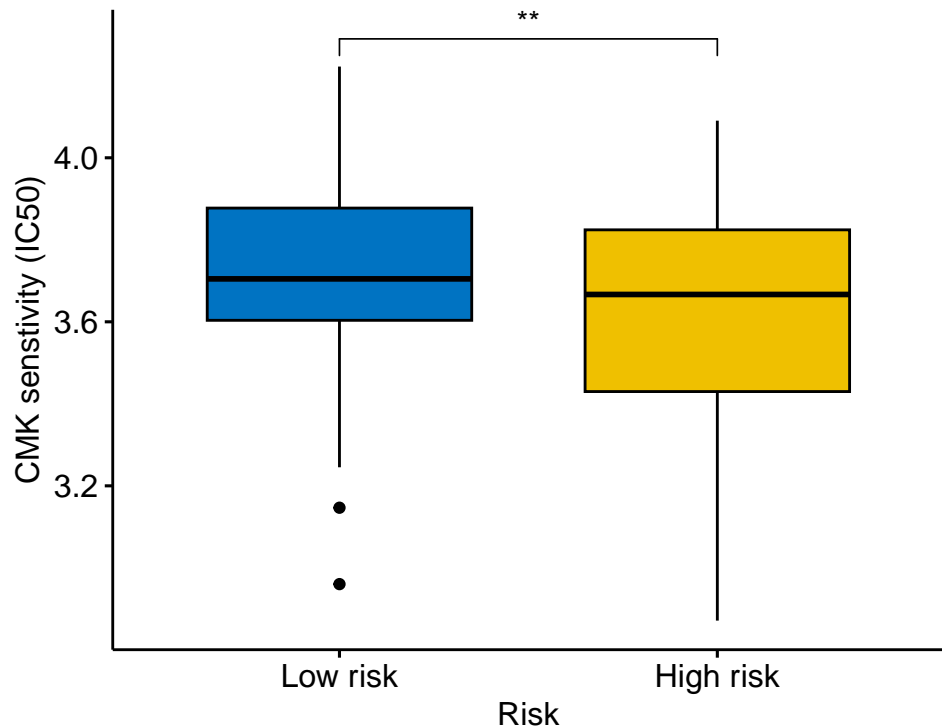

Supplement: Supplementary file 3 [file DataSheet_3.zip › original data 7-9/8-drug sensitivity/durgSenstivity.CMK.pdf]

Risk   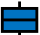 Low risk   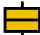 High risk

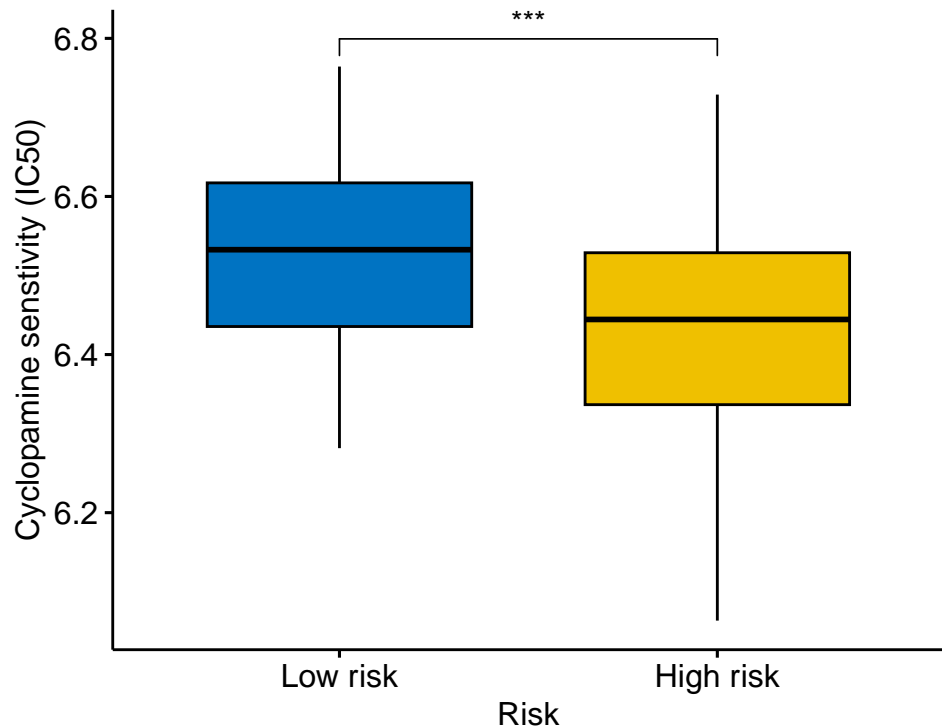

Supplement: Supplementary file 3 [file DataSheet_3.zip › original data 7-9/8-drug sensitivity/durgSenstivity.Cyclopamine.pdf]

Risk   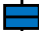 Low risk   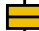 High risk

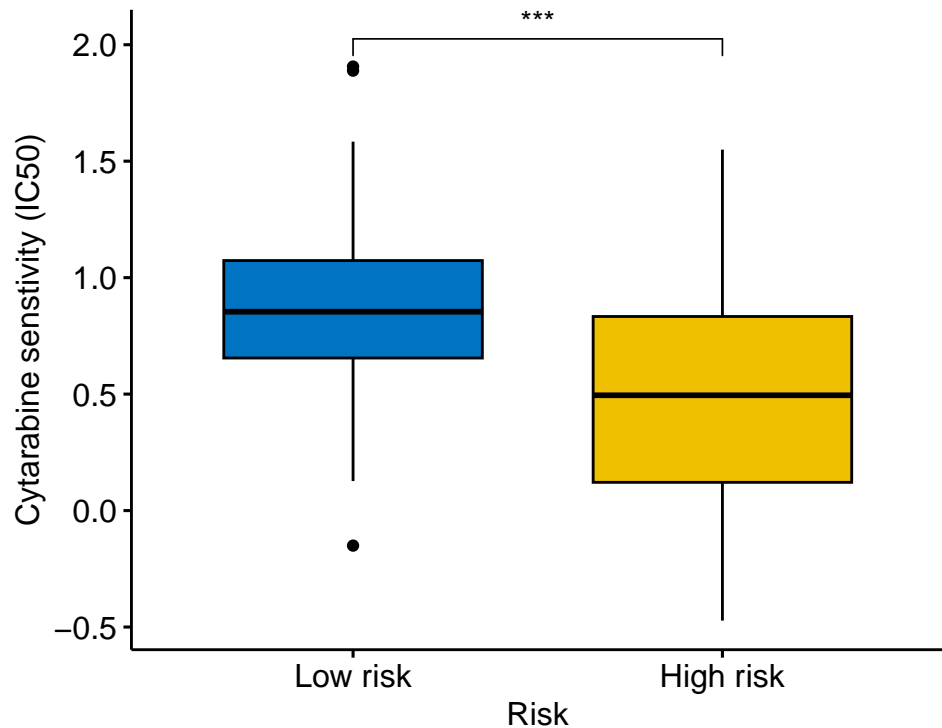

Supplement: Supplementary file 3 [file DataSheet_3.zip › original data 7-9/8-drug sensitivity/durgSenstivity.Cytarabine.pdf]

Risk 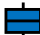 Low risk 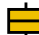 High risk

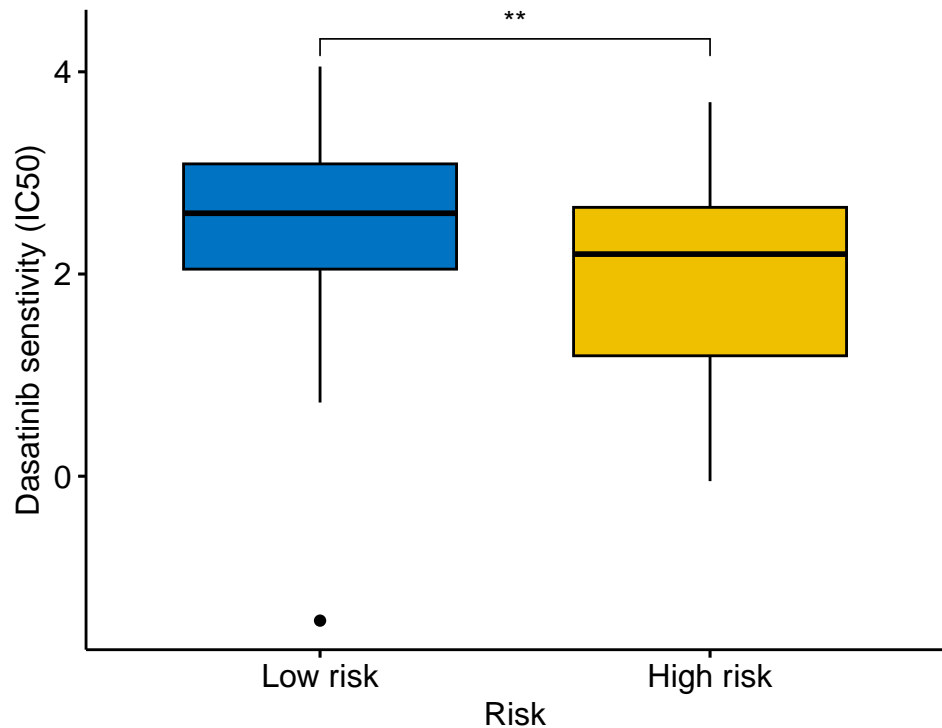

Supplement: Supplementary file 3 [file DataSheet_3.zip › original data 7-9/8-drug sensitivity/durgSenstivity.Dasatinib.pdf]

Risk    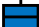 Low risk    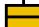 High risk

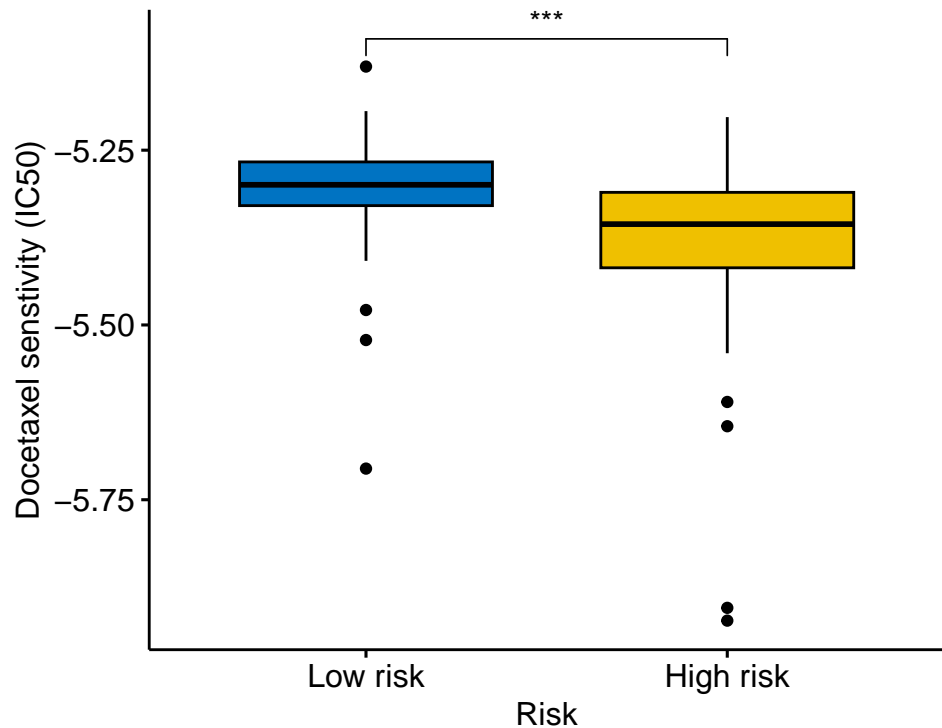

Supplement: Supplementary file 3 [file DataSheet_3.zip › original data 7-9/8-drug sensitivity/durgSenstivity.Docetaxel.pdf]

Risk Low risk High risk

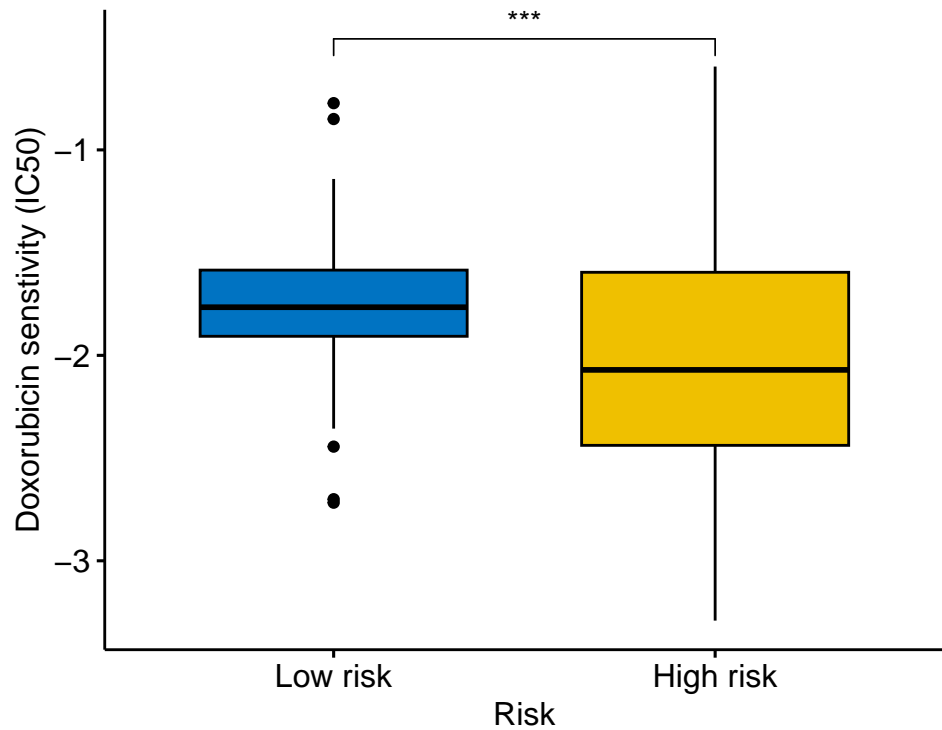

Supplement: Supplementary file 3 [file DataSheet_3.zip › original data 7-9/8-drug sensitivity/durgSenstivity.Doxorubicin.pdf]

Risk 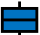 Low risk 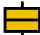 High risk

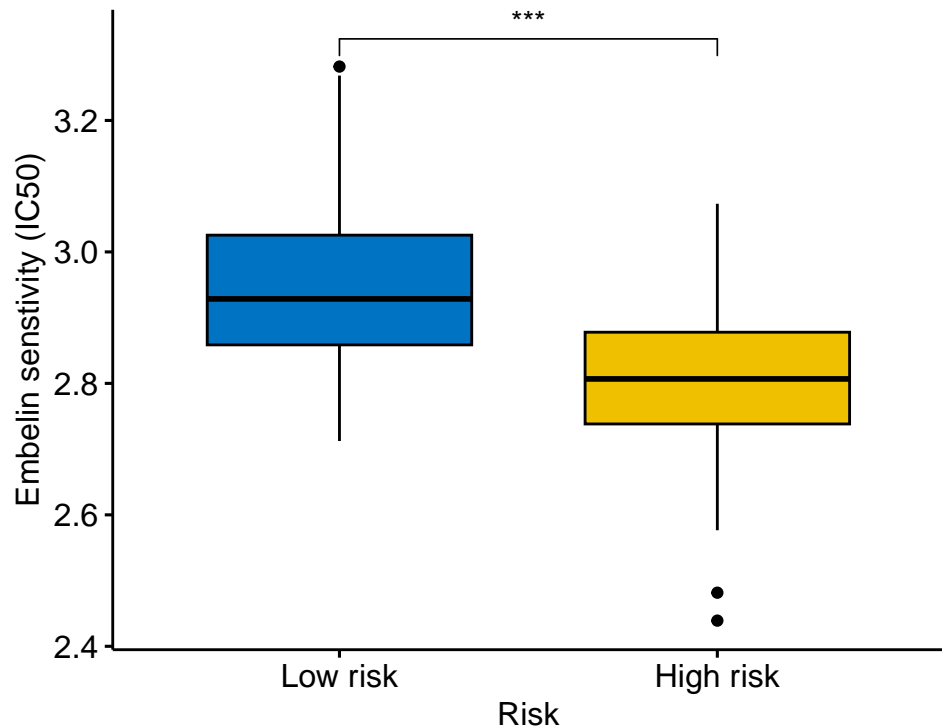

Supplement: Supplementary file 3 [file DataSheet_3.zip › original data 7-9/8-drug sensitivity/durgSenstivity.Embelin.pdf]

Risk Low risk High risk

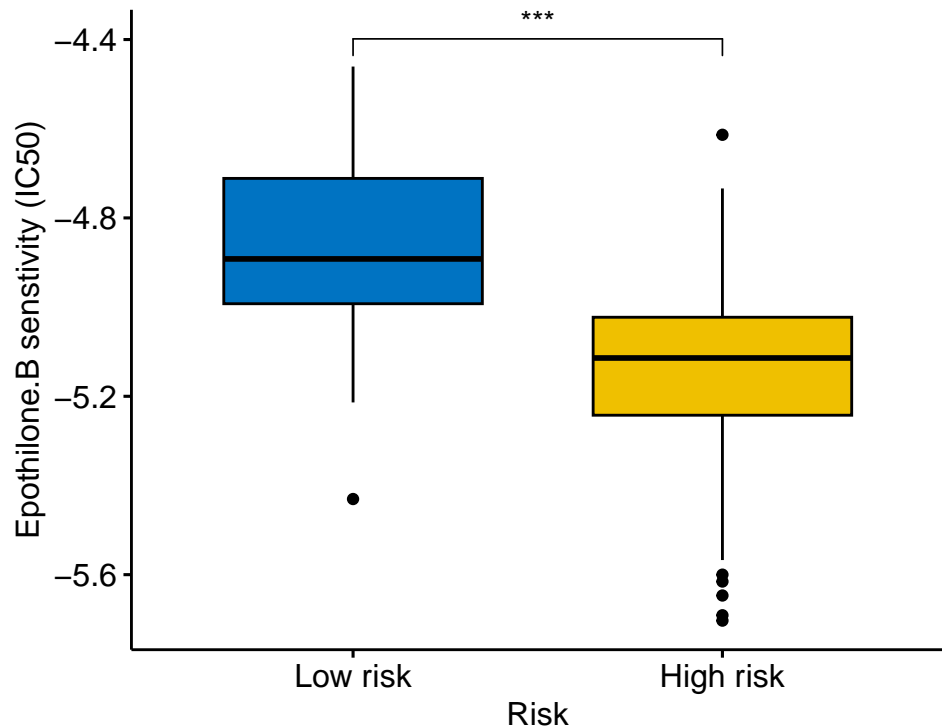

Supplement: Supplementary file 3 [file DataSheet_3.zip › original data 7-9/8-drug sensitivity/durgSenstivity.Epothilone.B.pdf]

Risk Low risk High risk

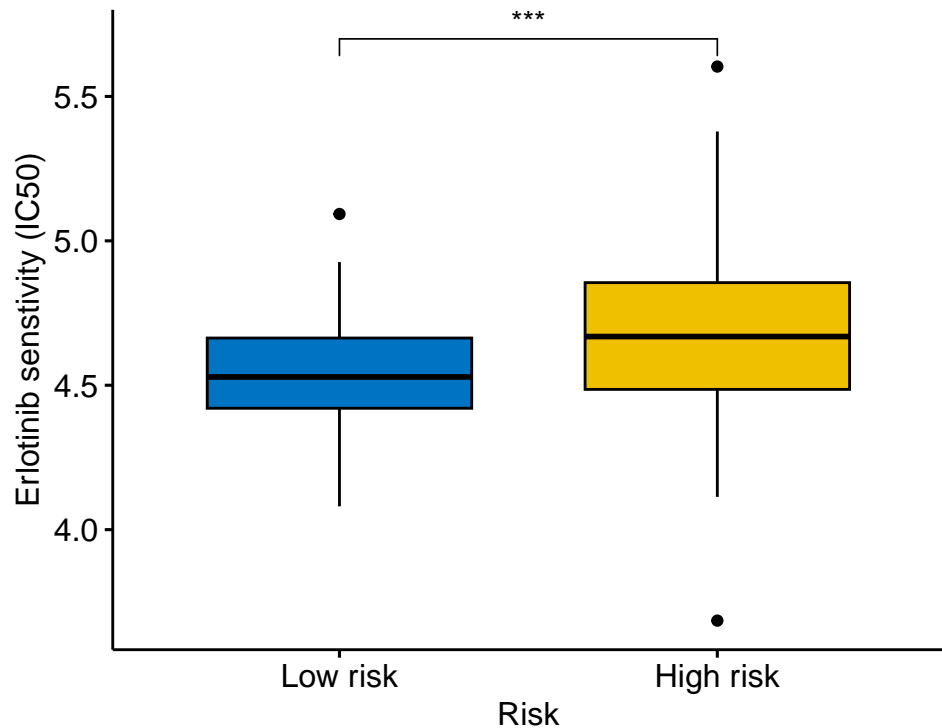

Supplement: Supplementary file 3 [file DataSheet_3.zip › original data 7-9/8-drug sensitivity/durgSenstivity.Erlotinib.pdf]

Risk 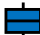 Low risk 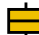 High risk

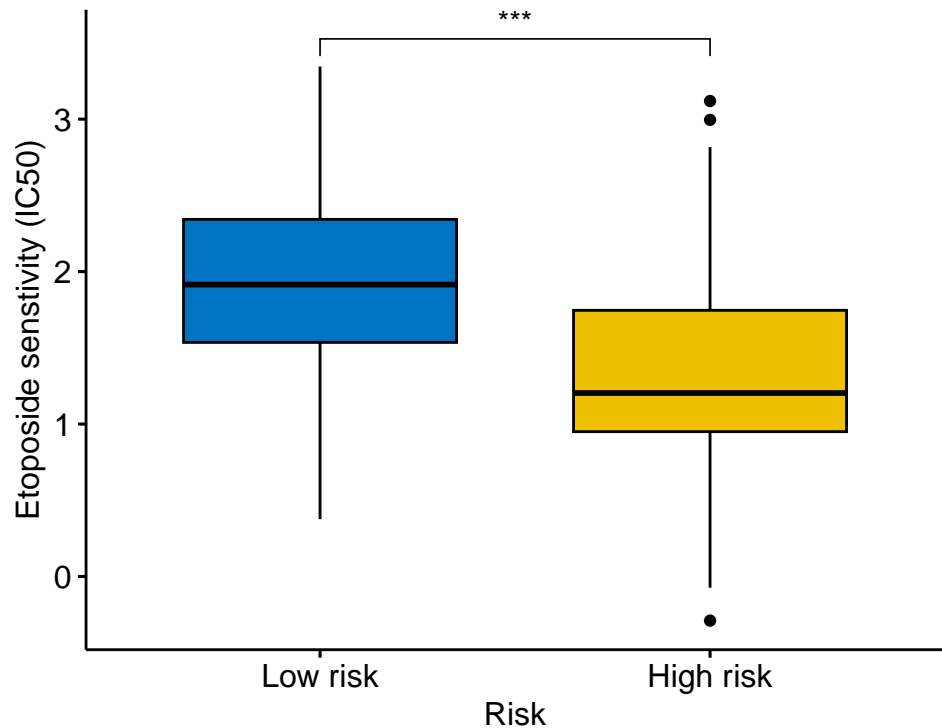

Supplement: Supplementary file 3 [file DataSheet_3.zip › original data 7-9/8-drug sensitivity/durgSenstivity.Etoposide.pdf]

Risk 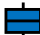 Low risk 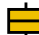 High risk

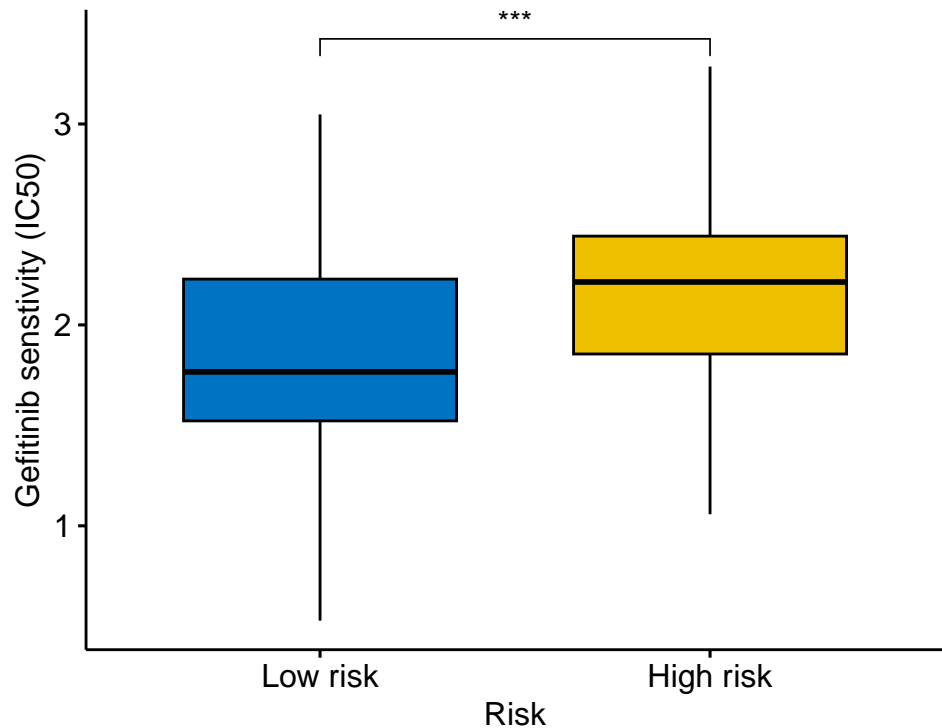

Supplement: Supplementary file 3 [file DataSheet_3.zip › original data 7-9/8-drug sensitivity/durgSenstivity.Gefitinib.pdf]

Risk Low risk High risk

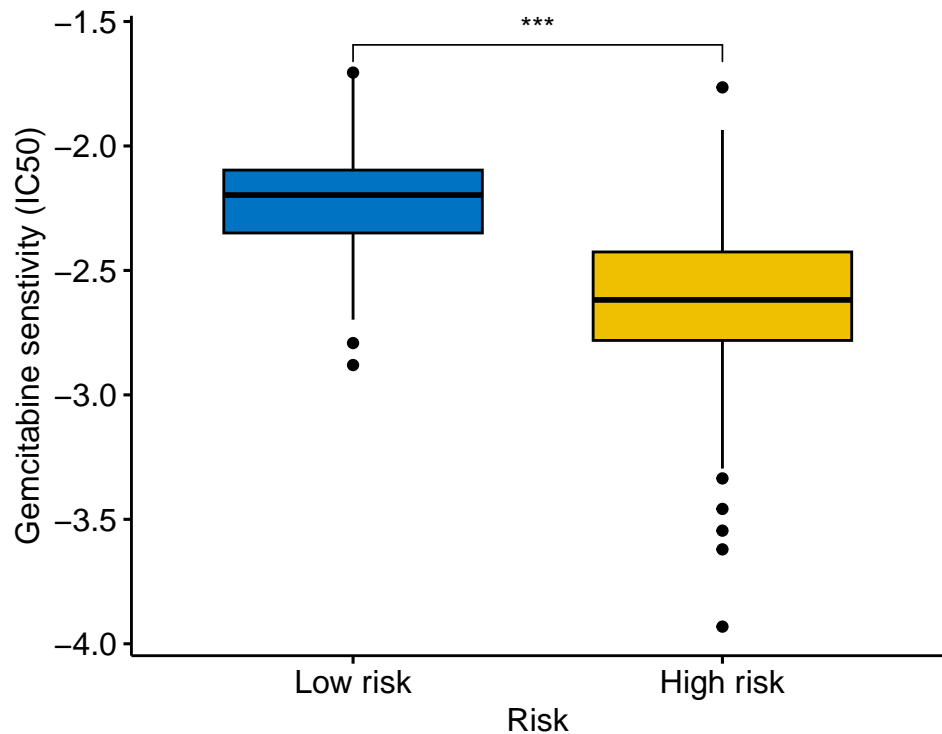

Supplement: Supplementary file 3 [file DataSheet_3.zip › original data 7-9/8-drug sensitivity/durgSenstivity.Gemcitabine.pdf]

Risk Low risk High risk

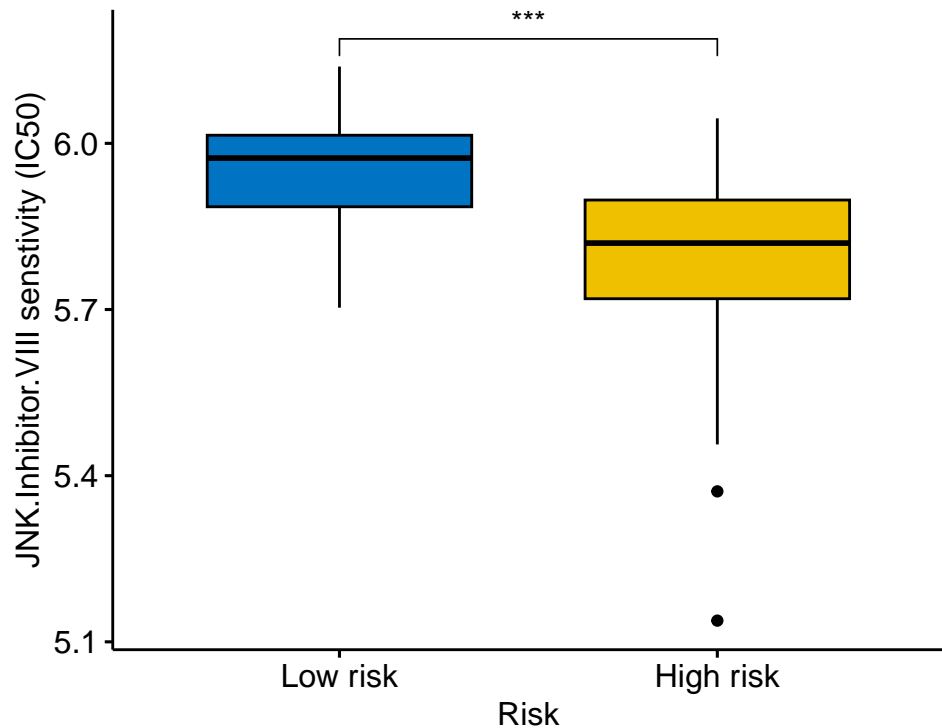

Supplement: Supplementary file 3 [file DataSheet_3.zip › original data 7-9/8-drug sensitivity/durgSenstivity.JNK.Inhibitor.VIII.pdf]

Risk   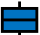 Low risk   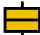 High risk

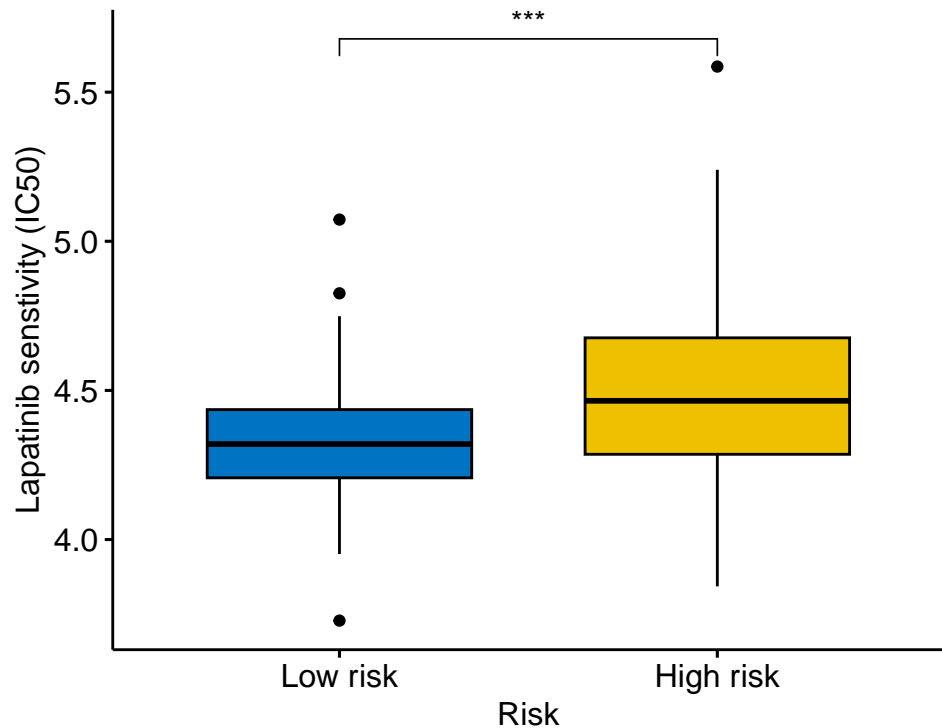

Supplement: Supplementary file 3 [file DataSheet_3.zip › original data 7-9/8-drug sensitivity/durgSenstivity.Lapatinib.pdf]

Risk   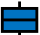 Low risk   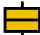 High risk

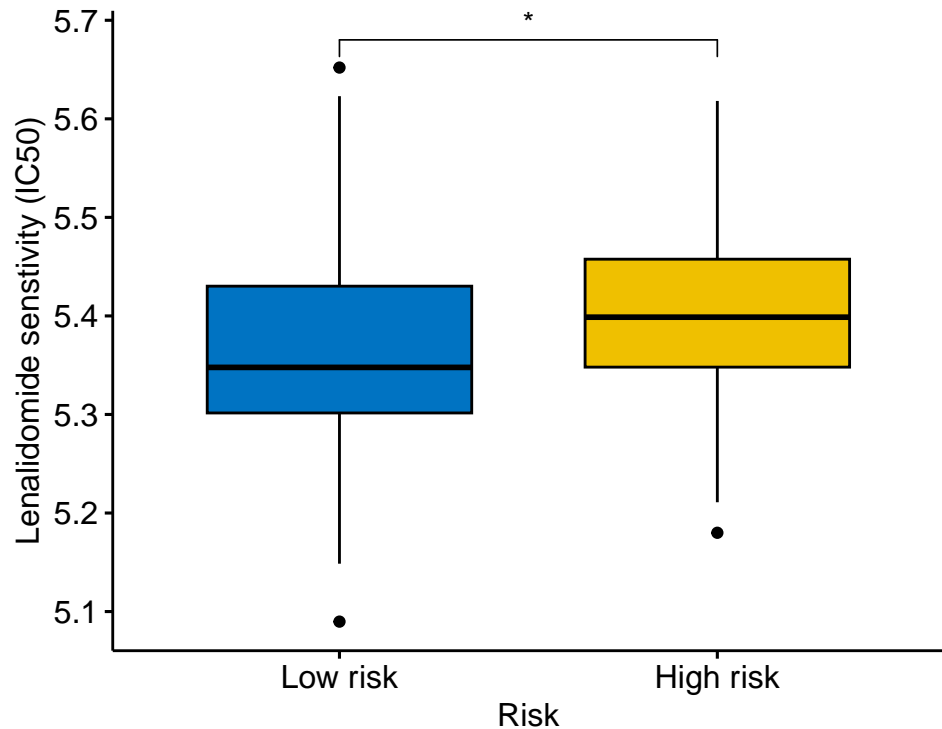

Supplement: Supplementary file 3 [file DataSheet_3.zip › original data 7-9/8-drug sensitivity/durgSenstivity.Lenalidomide.pdf]

Risk   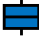 Low risk   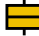 High risk

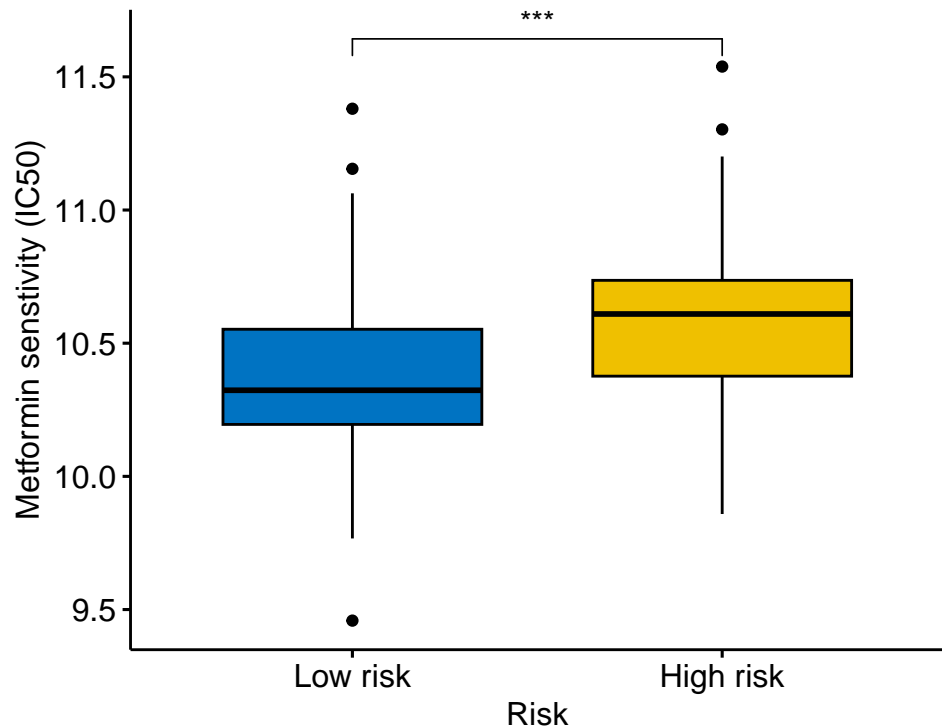

Supplement: Supplementary file 3 [file DataSheet_3.zip › original data 7-9/8-drug sensitivity/durgSenstivity.Metformin.pdf]

Risk    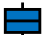 Low risk    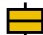 High risk

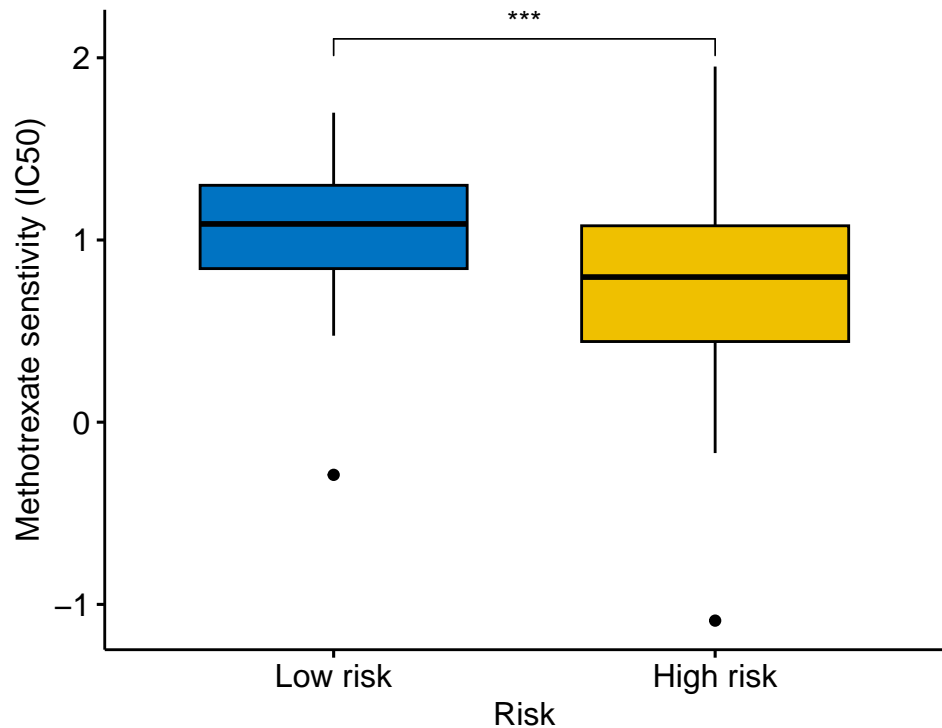

Supplement: Supplementary file 3 [file DataSheet_3.zip › original data 7-9/8-drug sensitivity/durgSenstivity.Methotrexate.pdf]

Risk Low risk High risk

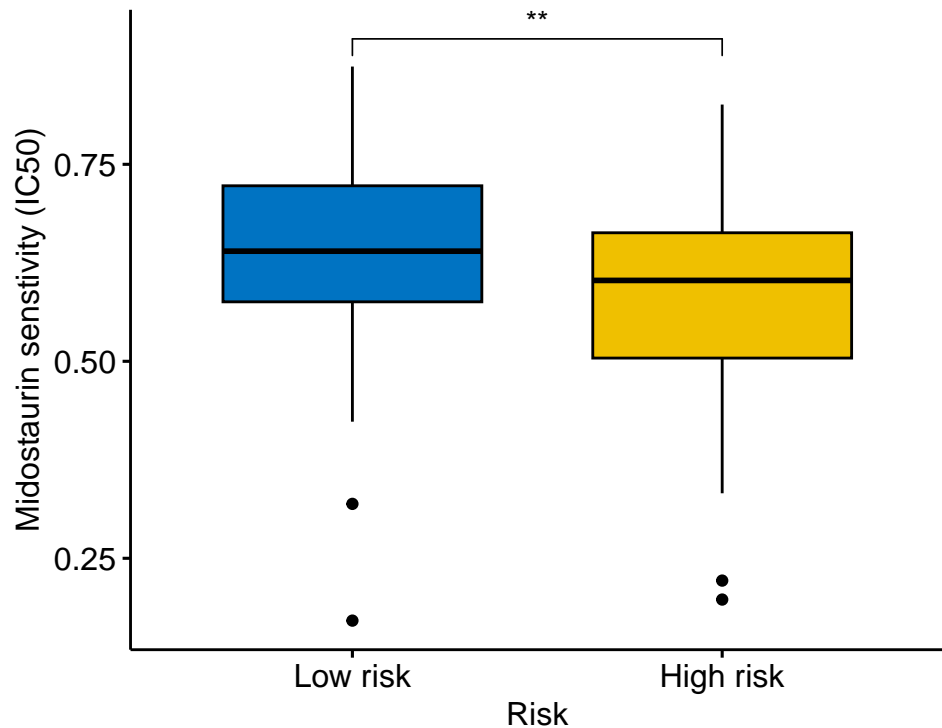

Supplement: Supplementary file 3 [file DataSheet_3.zip › original data 7-9/8-drug sensitivity/durgSenstivity.Midostaurin.pdf]

Risk   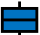 Low risk   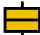 High risk

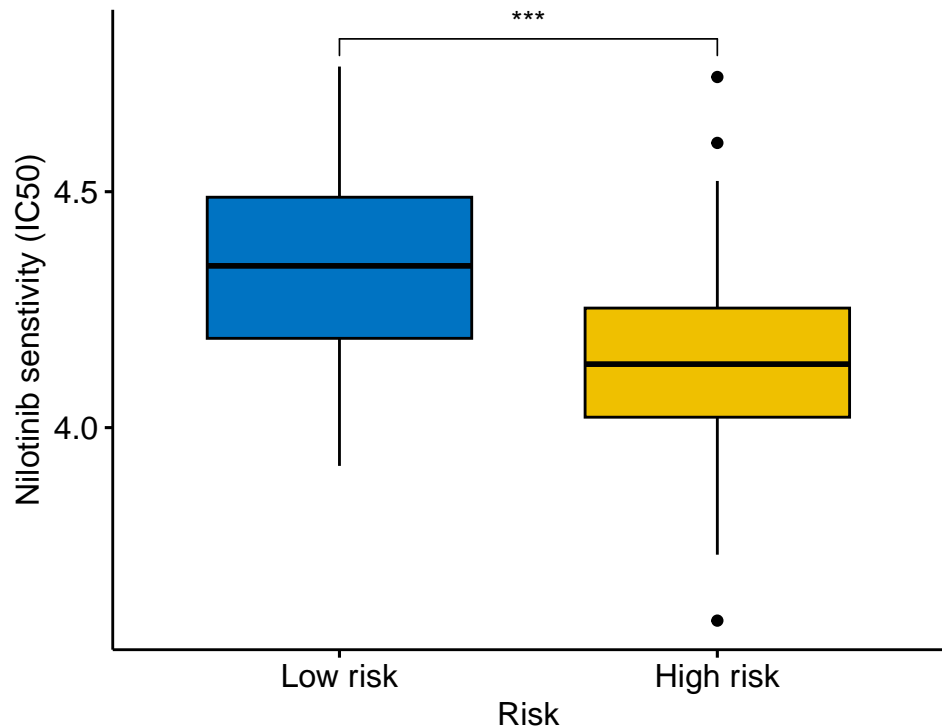

Supplement: Supplementary file 3 [file DataSheet_3.zip › original data 7-9/8-drug sensitivity/durgSenstivity.Nilotinib.pdf]

Risk   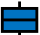 Low risk   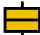 High risk

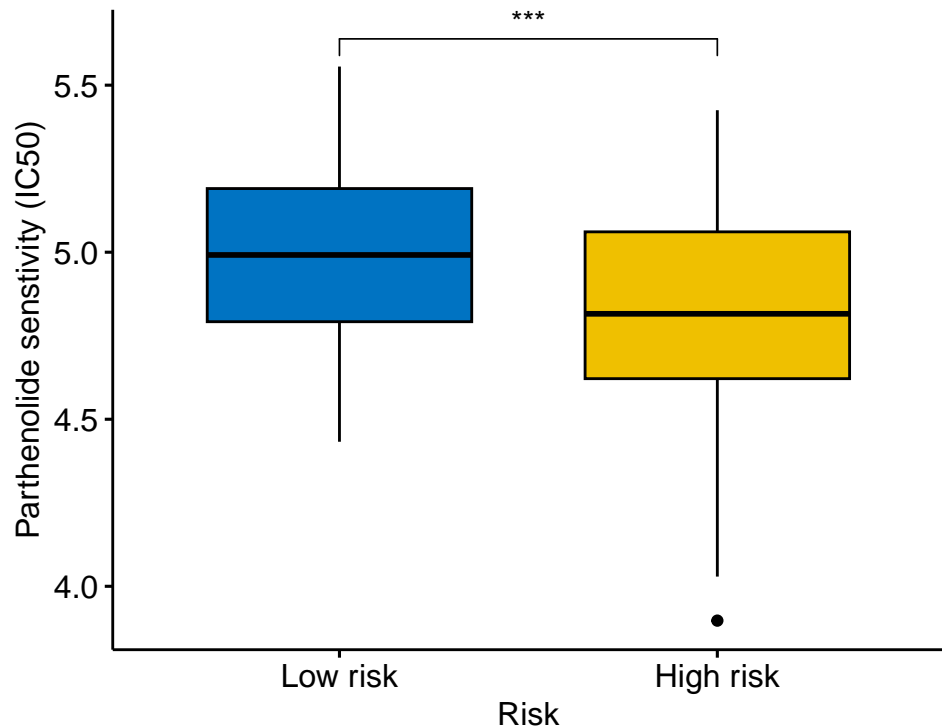

Supplement: Supplementary file 3 [file DataSheet_3.zip › original data 7-9/8-drug sensitivity/durgSenstivity.Parthenolide.pdf]

Risk Low risk High risk

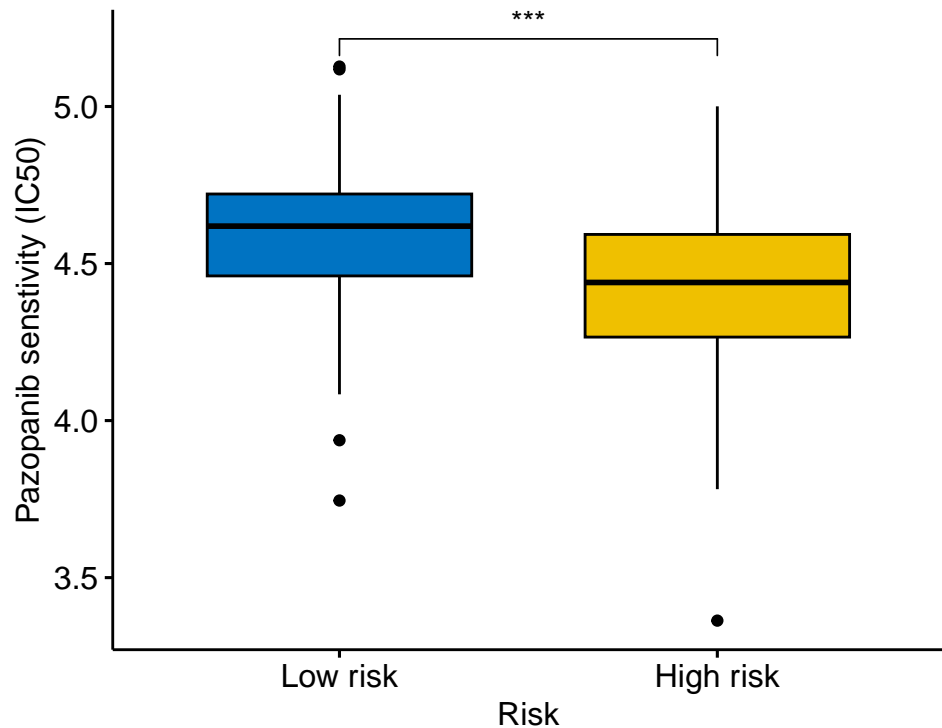

Supplement: Supplementary file 3 [file DataSheet_3.zip › original data 7-9/8-drug sensitivity/durgSenstivity.Pazopanib.pdf]

Risk Low risk High risk

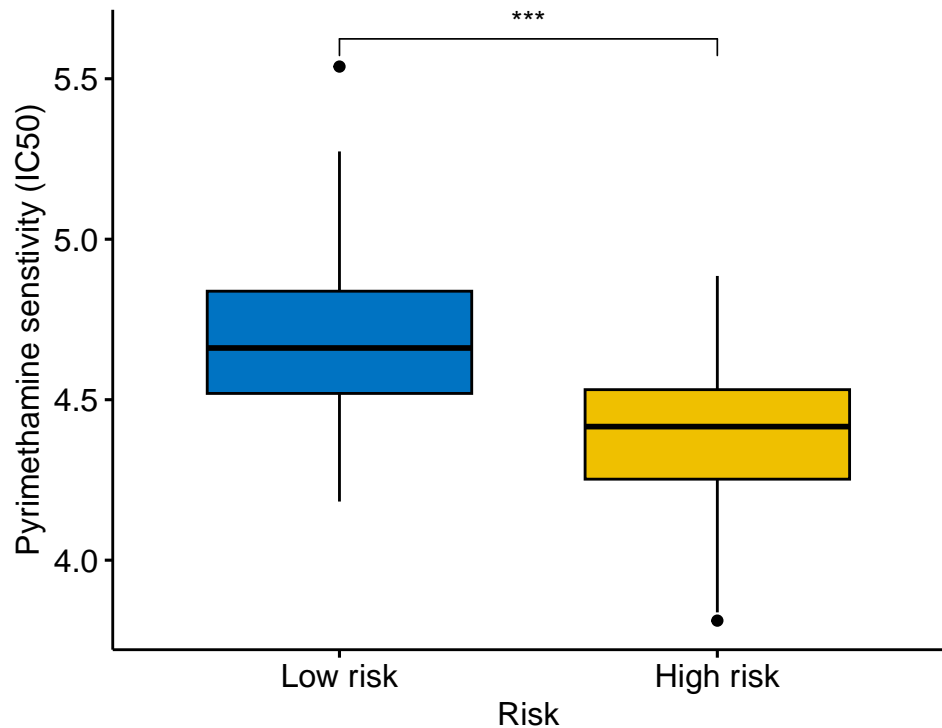

Supplement: Supplementary file 3 [file DataSheet_3.zip › original data 7-9/8-drug sensitivity/durgSenstivity.Pyrimethamine.pdf]

Risk   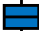 Low risk   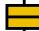 High risk

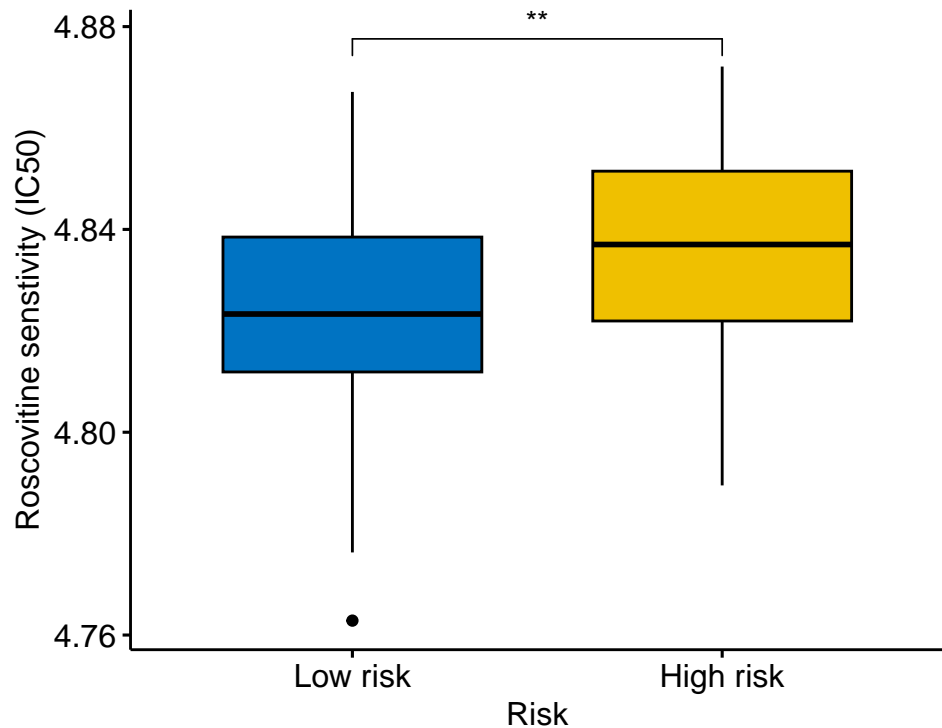

Supplement: Supplementary file 3 [file DataSheet_3.zip › original data 7-9/8-drug sensitivity/durgSenstivity.Roscovitine.pdf]

Risk   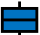 Low risk   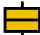 High risk

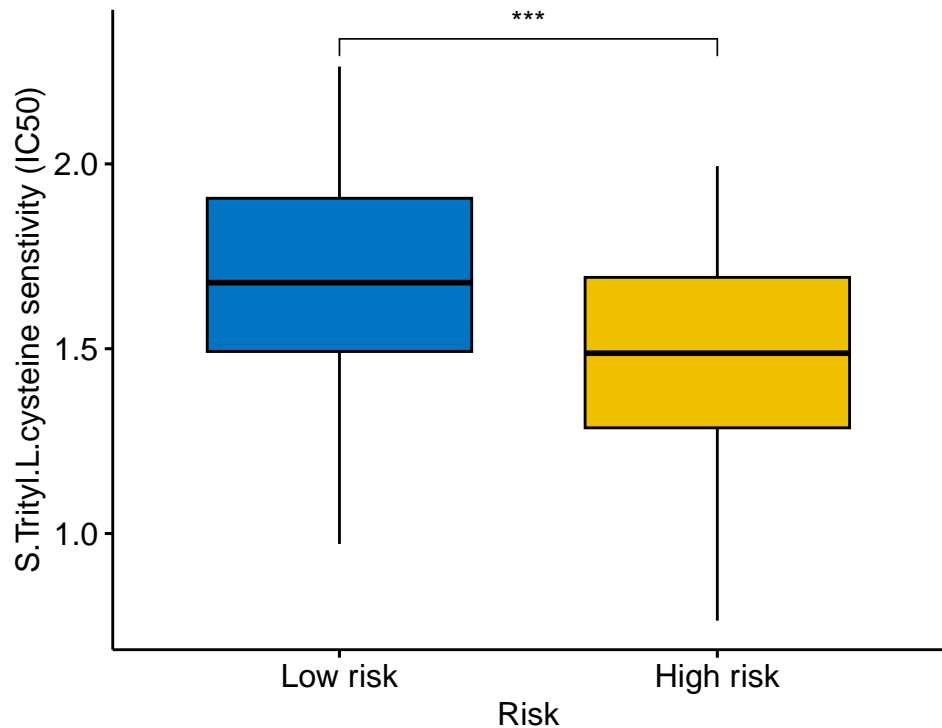

Supplement: Supplementary file 3 [file DataSheet_3.zip › original data 7-9/8-drug sensitivity/durgSenstivity.S.Trityl.L.cysteine.pdf]

Risk   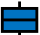 Low risk   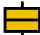 High risk

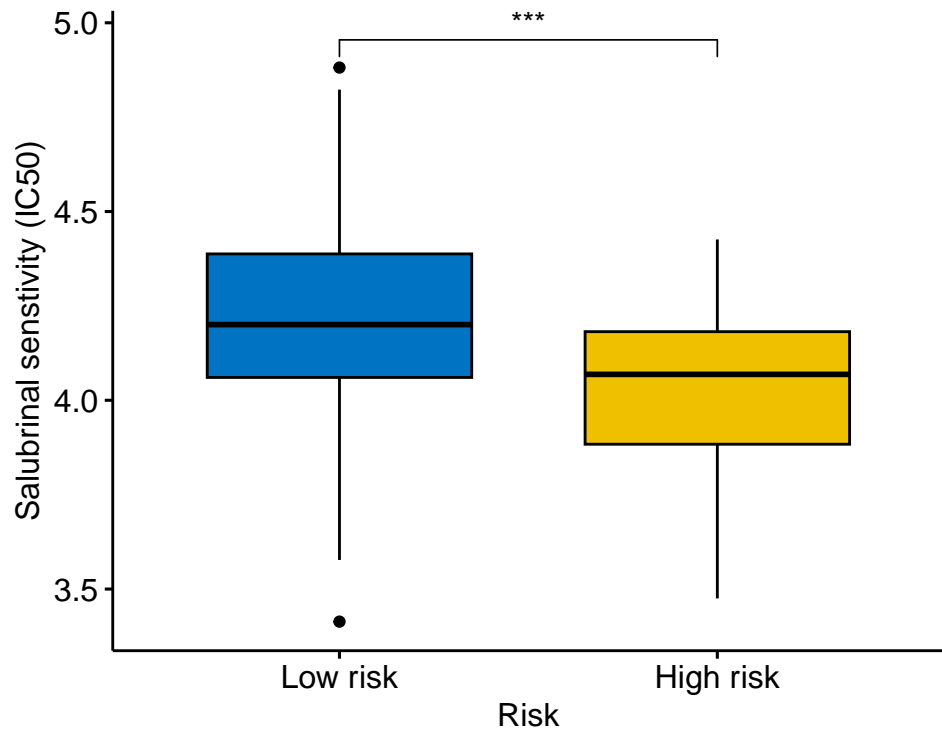

Supplement: Supplementary file 3 [file DataSheet_3.zip › original data 7-9/8-drug sensitivity/durgSenstivity.Salubrinal.pdf]

Risk 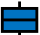 Low risk 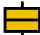 High risk

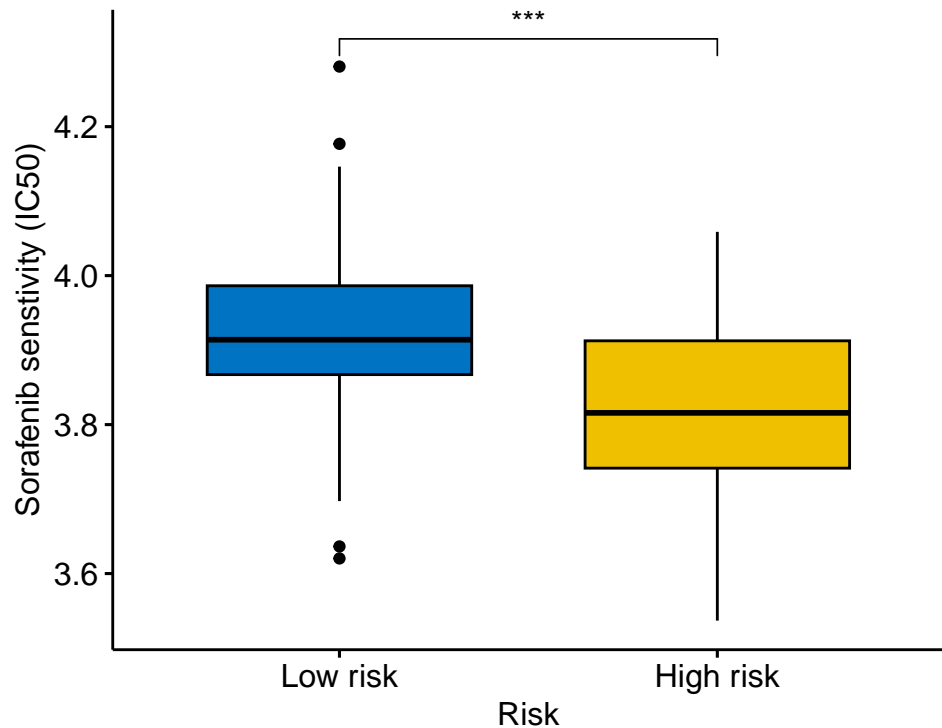

Supplement: Supplementary file 3 [file DataSheet_3.zip › original data 7-9/8-drug sensitivity/durgSenstivity.Sorafenib.pdf]

Risk Low risk High risk

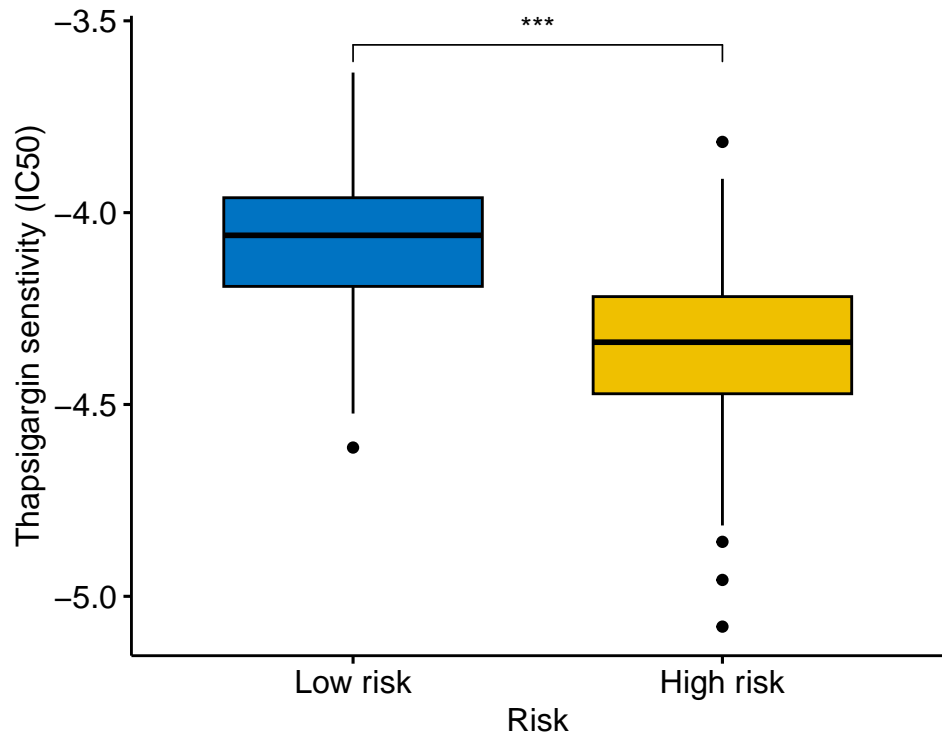

Supplement: Supplementary file 3 [file DataSheet_3.zip › original data 7-9/8-drug sensitivity/durgSenstivity.Thapsigargin.pdf]
