# Supplementary material for: Hepatitis B-related hepatocellular carcinoma: classification and prognostic model based on programmed cell death genes
Source: Front Immunol. 2024 May 10;15:1411161. doi: 10.3389/fimmu.2024.1411161 (PMC11116790; doi:10.3389/fimmu.2024.1411161)
Supplement: Supplementary file 3 [file DataSheet_3.zip › original data 7-9/9-prediction/nomo.pdf]

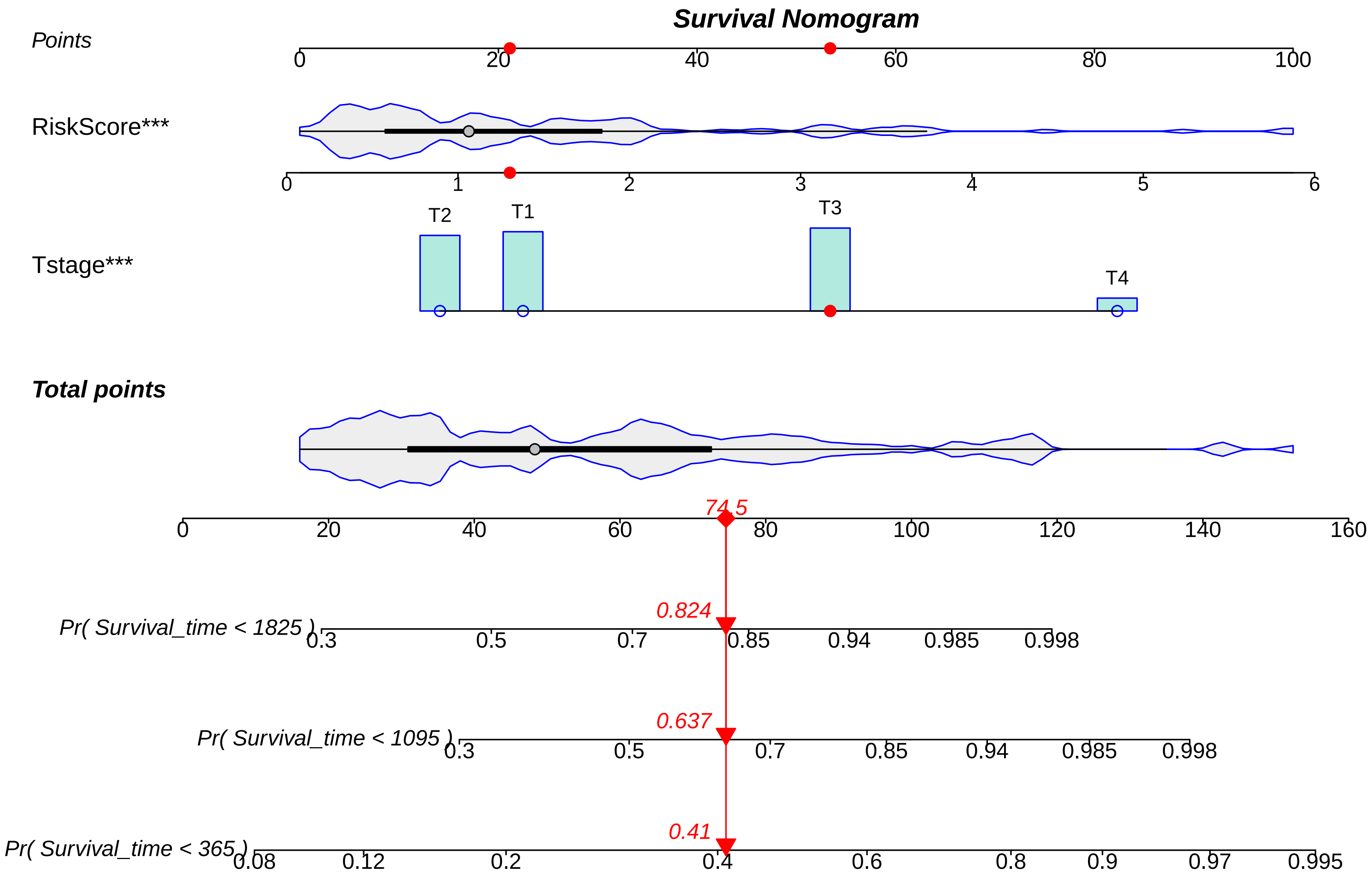

Survival Nomogram
Points
0 20 40 60 80 100
RiskScore***
0 1 2 3 4 5 6
Tstage***
T2 T1 T3 T4
Total points
74.5
0 20 40 60 80 100 120 140 160
Pr(Survival_time < 1825)
0.824
0.3 0.5 0.7 0.85 0.94 0.985 0.998
Pr(Survival_time < 1095)
0.637
0.3 0.5 0.7 0.85 0.94 0.985 0.998
Pr(Survival_time < 365)
0.41
0.08 0.12 0.2 0.4 0.6 0.8 0.9 0.97 0.995
