# Supplementary figures and images for: Hepatitis B-related hepatocellular carcinoma: classification and prognostic model based on programmed cell death genes (part 2 of 2)
Source: Front Immunol. 2024 May 10;15:1411161. doi: 10.3389/fimmu.2024.1411161 (PMC11116790; doi:10.3389/fimmu.2024.1411161)

Risk 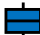 Low risk 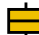 High risk

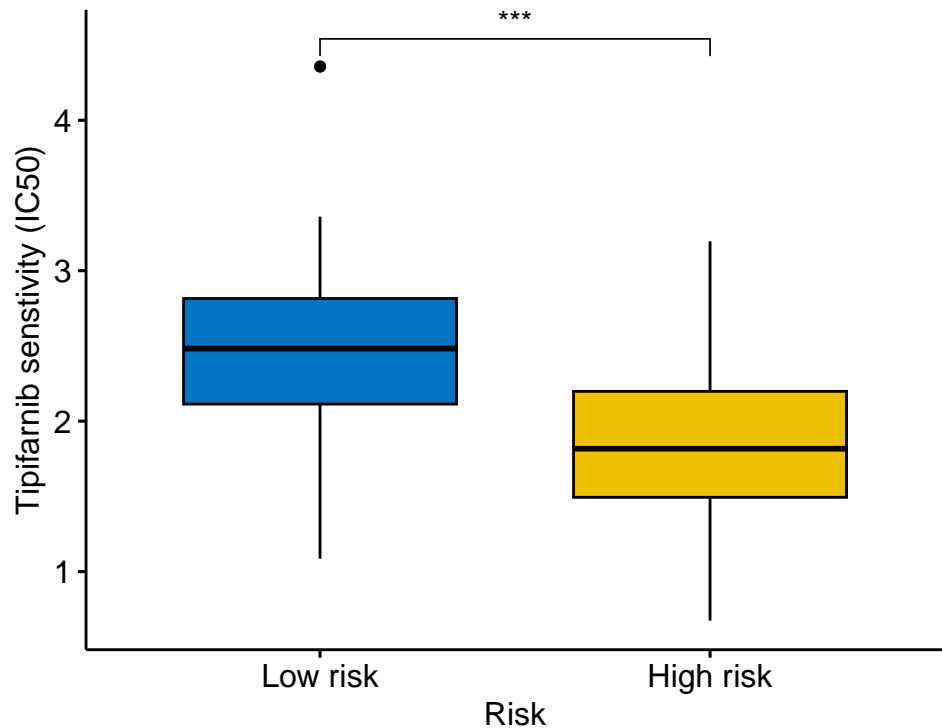

Supplement: Supplementary file 3 [file DataSheet_3.zip › original data 7-9/8-drug sensitivity/durgSenstivity.Tipifarnib.pdf]

Risk    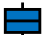 Low risk    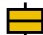 High risk

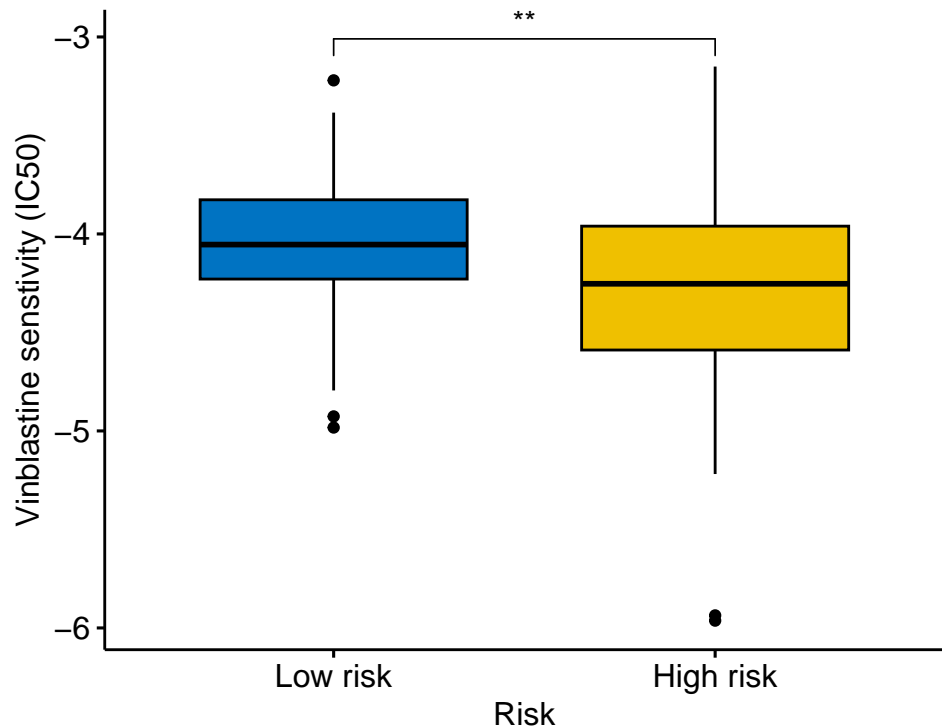

Supplement: Supplementary file 3 [file DataSheet_3.zip › original data 7-9/8-drug sensitivity/durgSenstivity.Vinblastine.pdf]

Risk   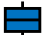 Low risk   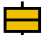 High risk

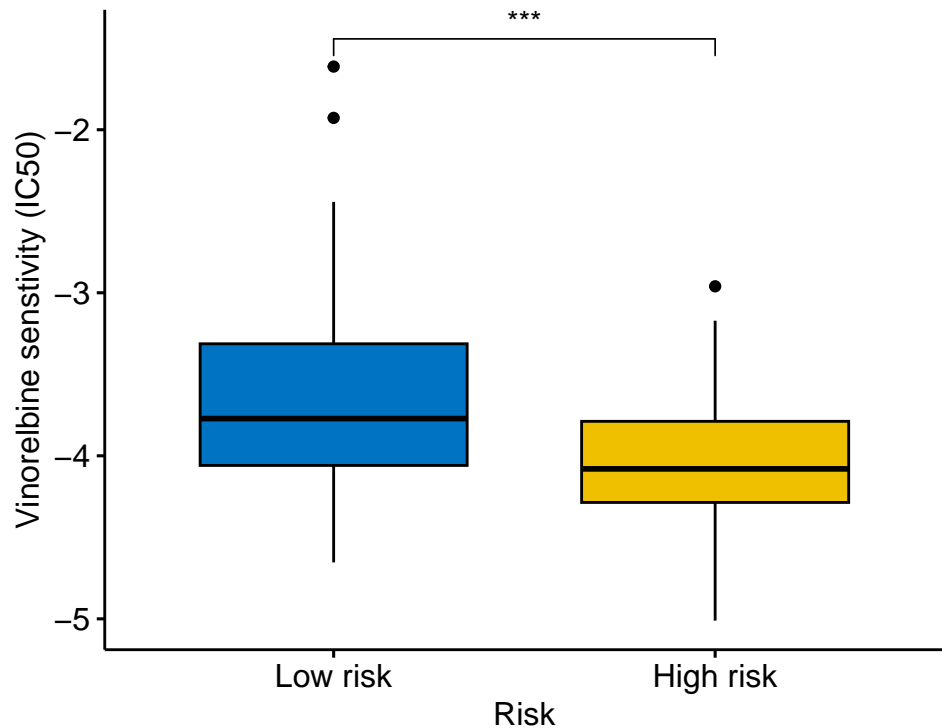

Supplement: Supplementary file 3 [file DataSheet_3.zip › original data 7-9/8-drug sensitivity/durgSenstivity.Vinorelbine.pdf]

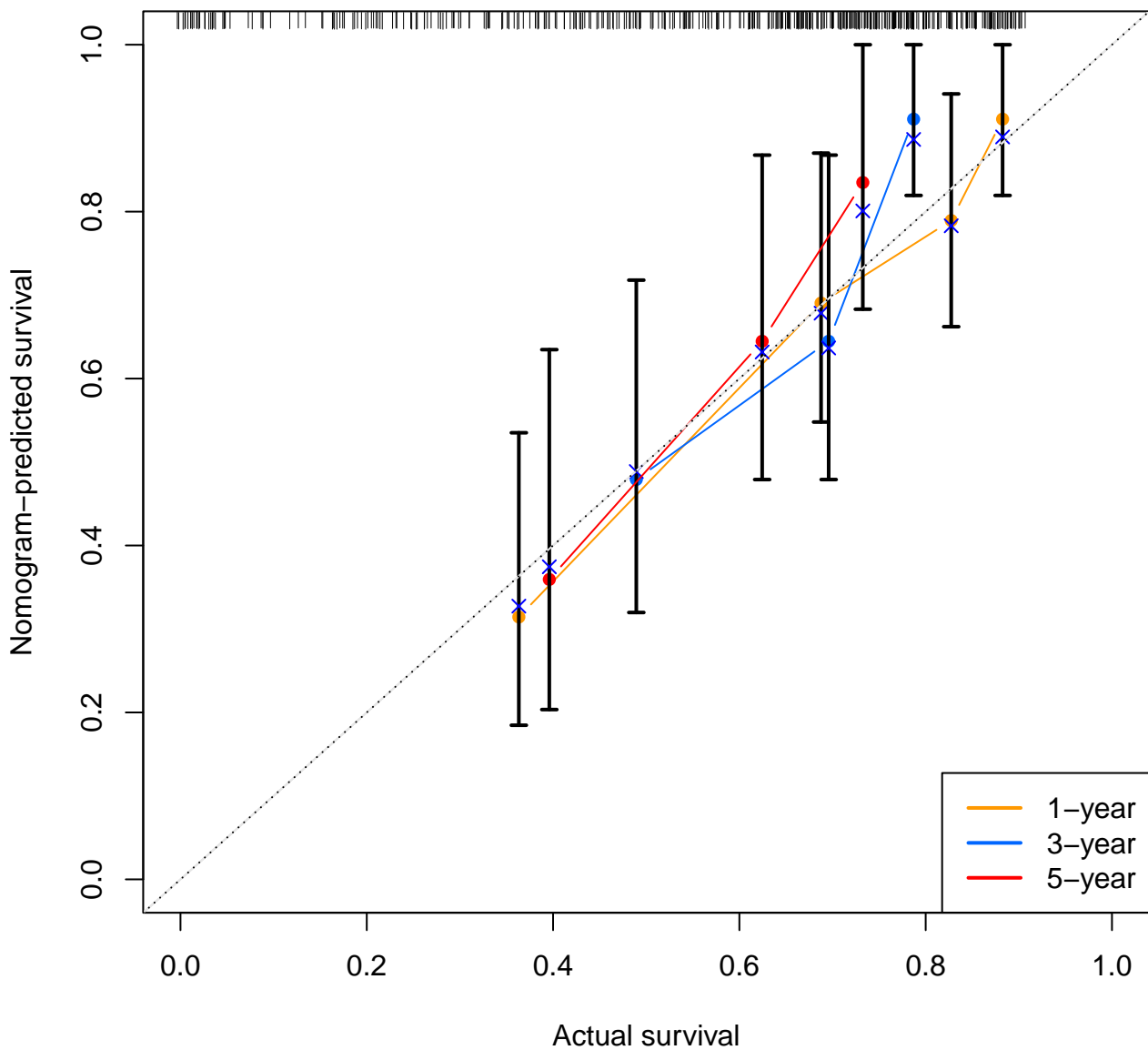

Supplement: Supplementary file 3 [file DataSheet_3.zip › original data 7-9/9-prediction/calibrate.pdf]

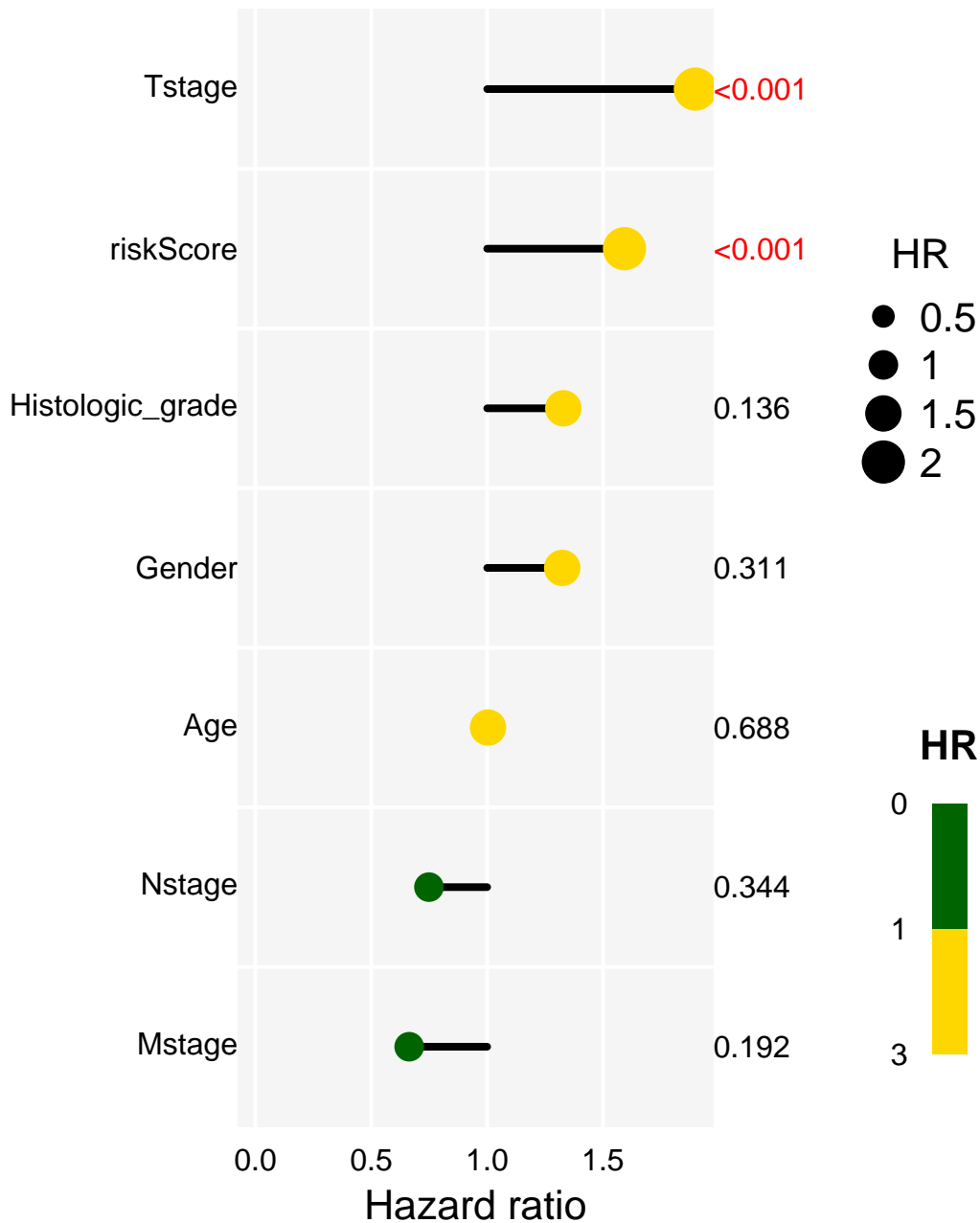

Supplement: Supplementary file 3 [file DataSheet_3.zip › original data 7-9/9-prediction/Forest-unicox.pdf]

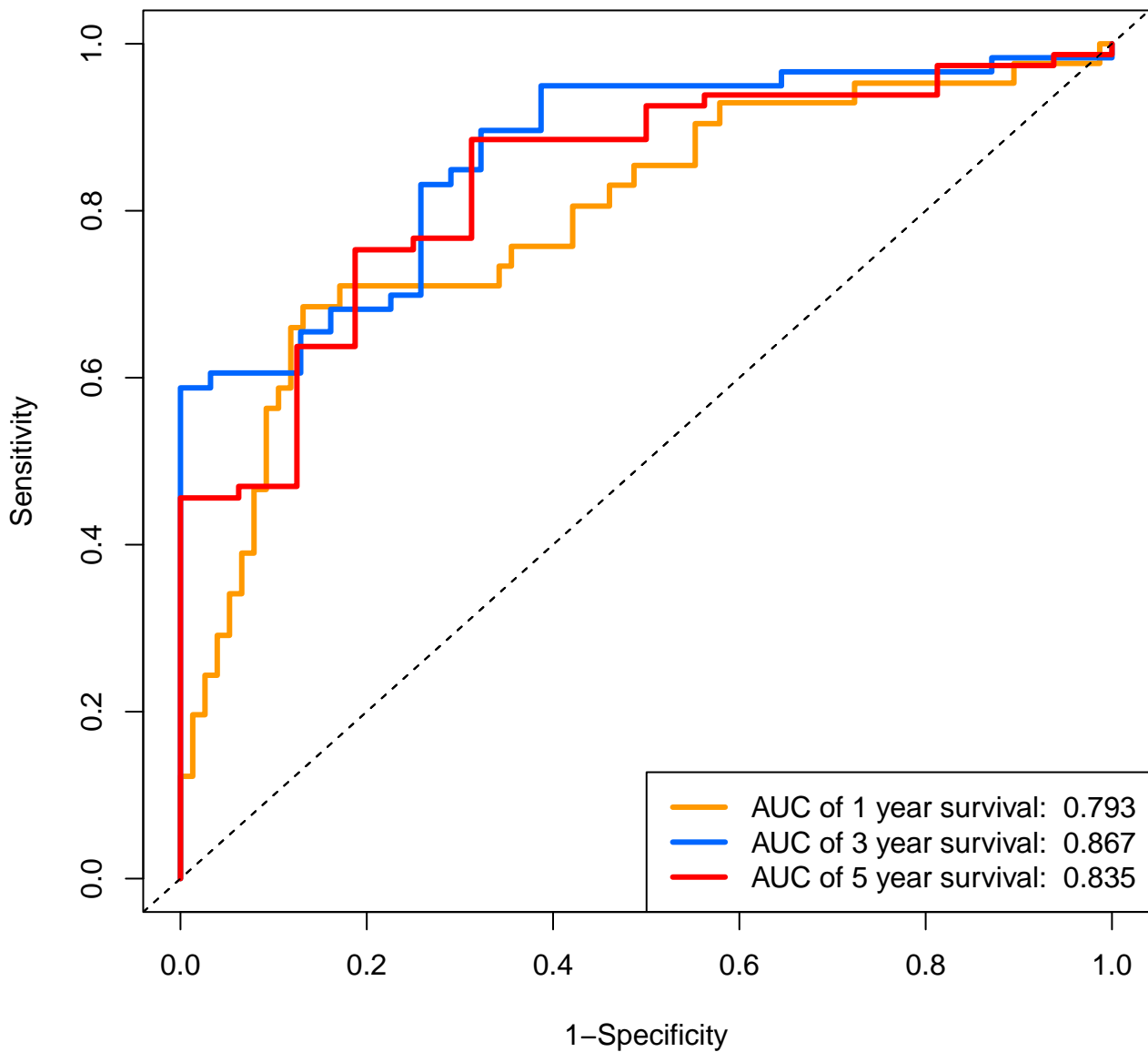

Supplement: Supplementary file 3 [file DataSheet_3.zip › original data 7-9/9-prediction/ROC.pdf]

**Survival curve of risk score( $P<0.001$ )**

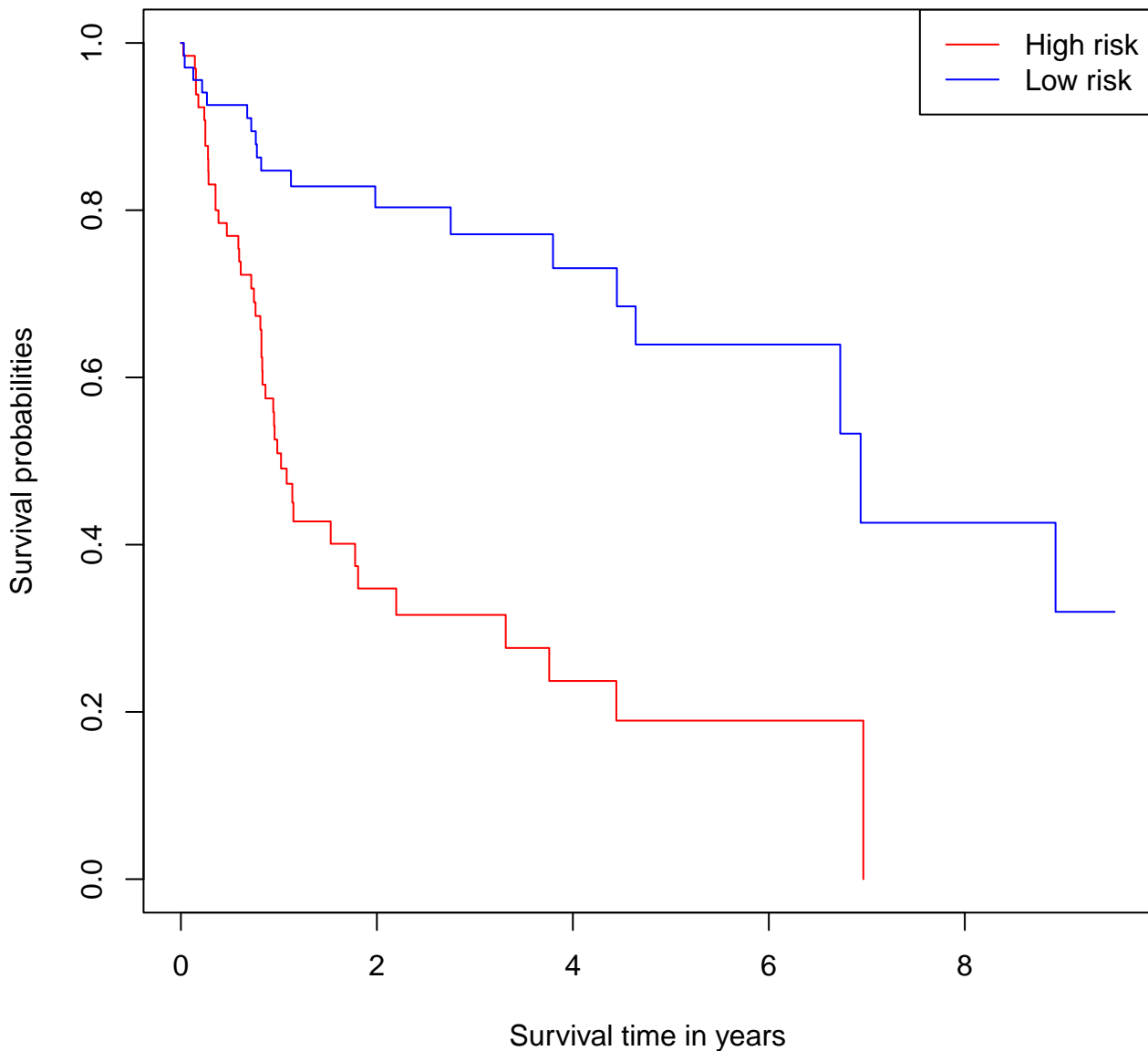

Supplement: Supplementary file 3 [file DataSheet_3.zip › original data 7-9/9-prediction/survival.pdf]

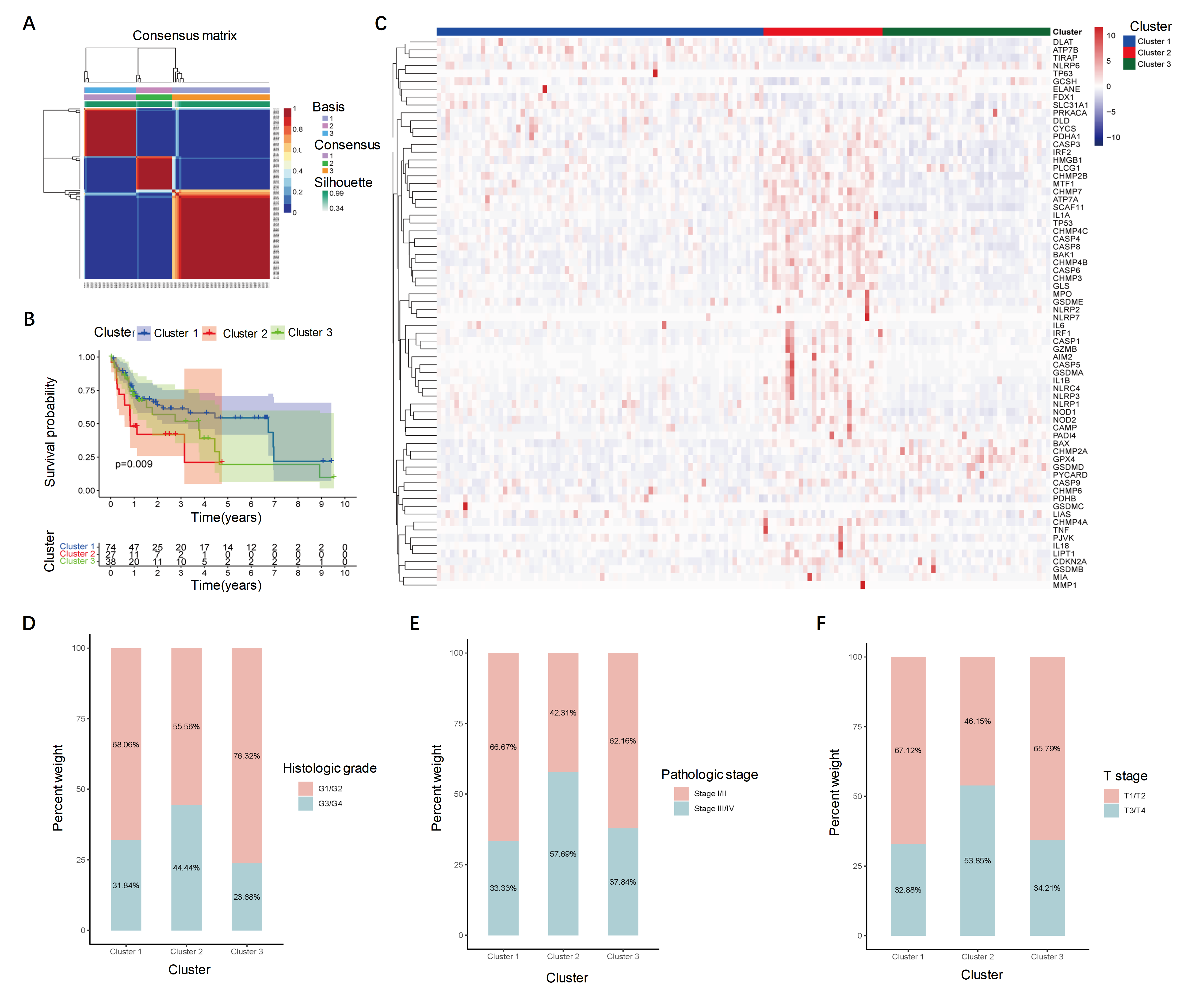

Supplement: Supplementary file 8 [file DataSheet_8.zip › figures/Figure1.tif]

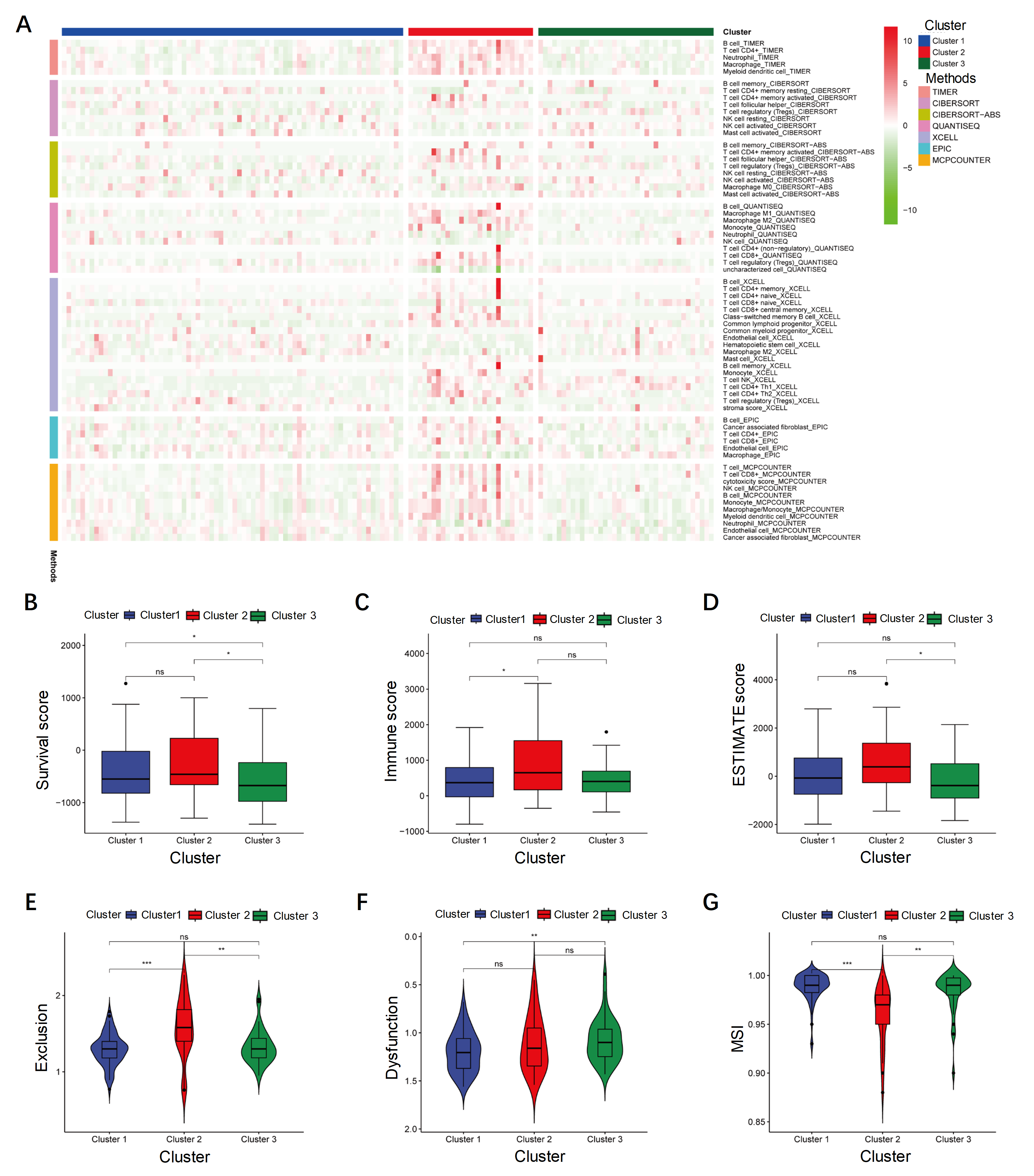

Supplement: Supplementary file 8 [file DataSheet_8.zip › figures/Figure2.tif]

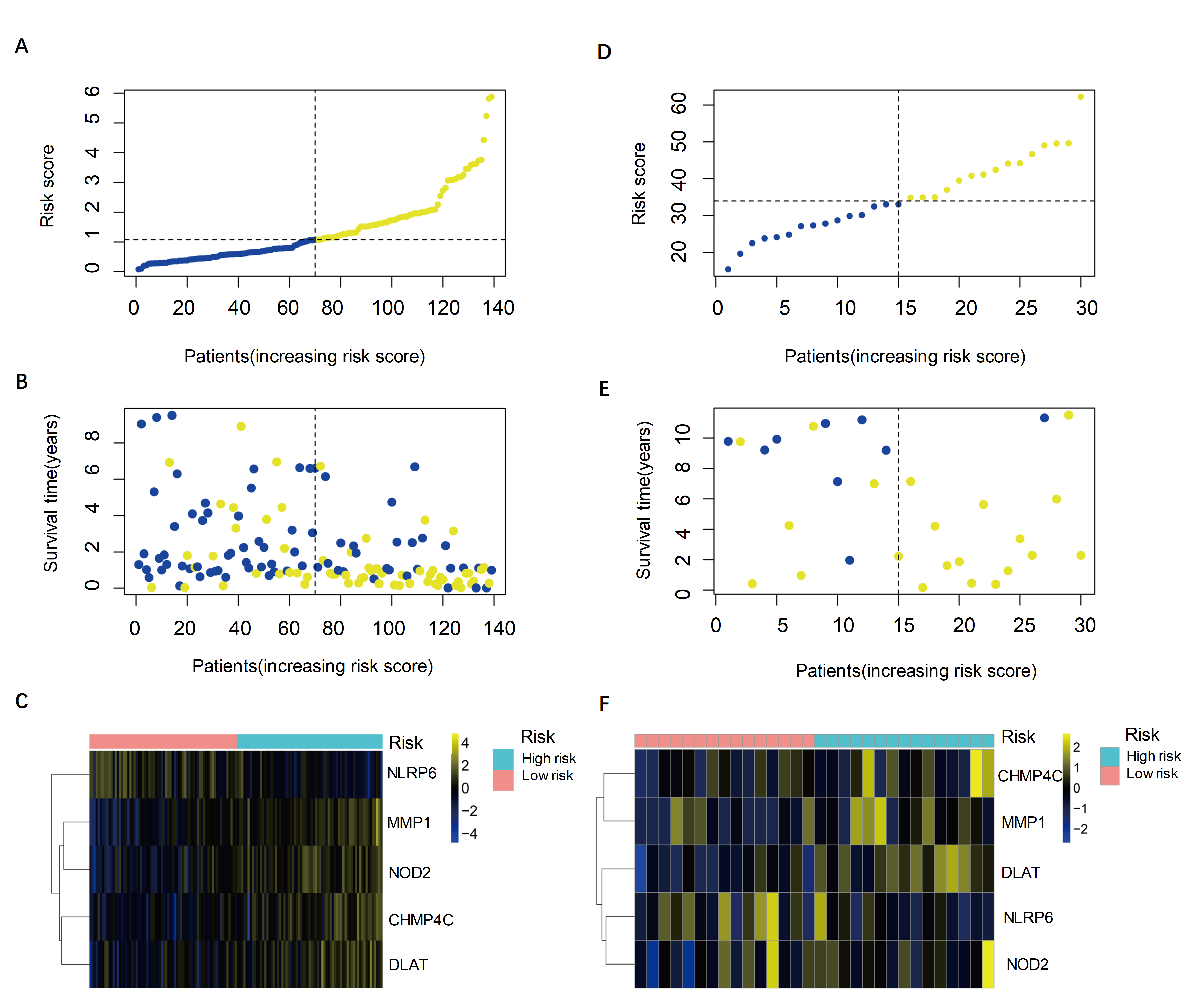

Supplement: Supplementary file 8 [file DataSheet_8.zip › figures/Figure6.tif]

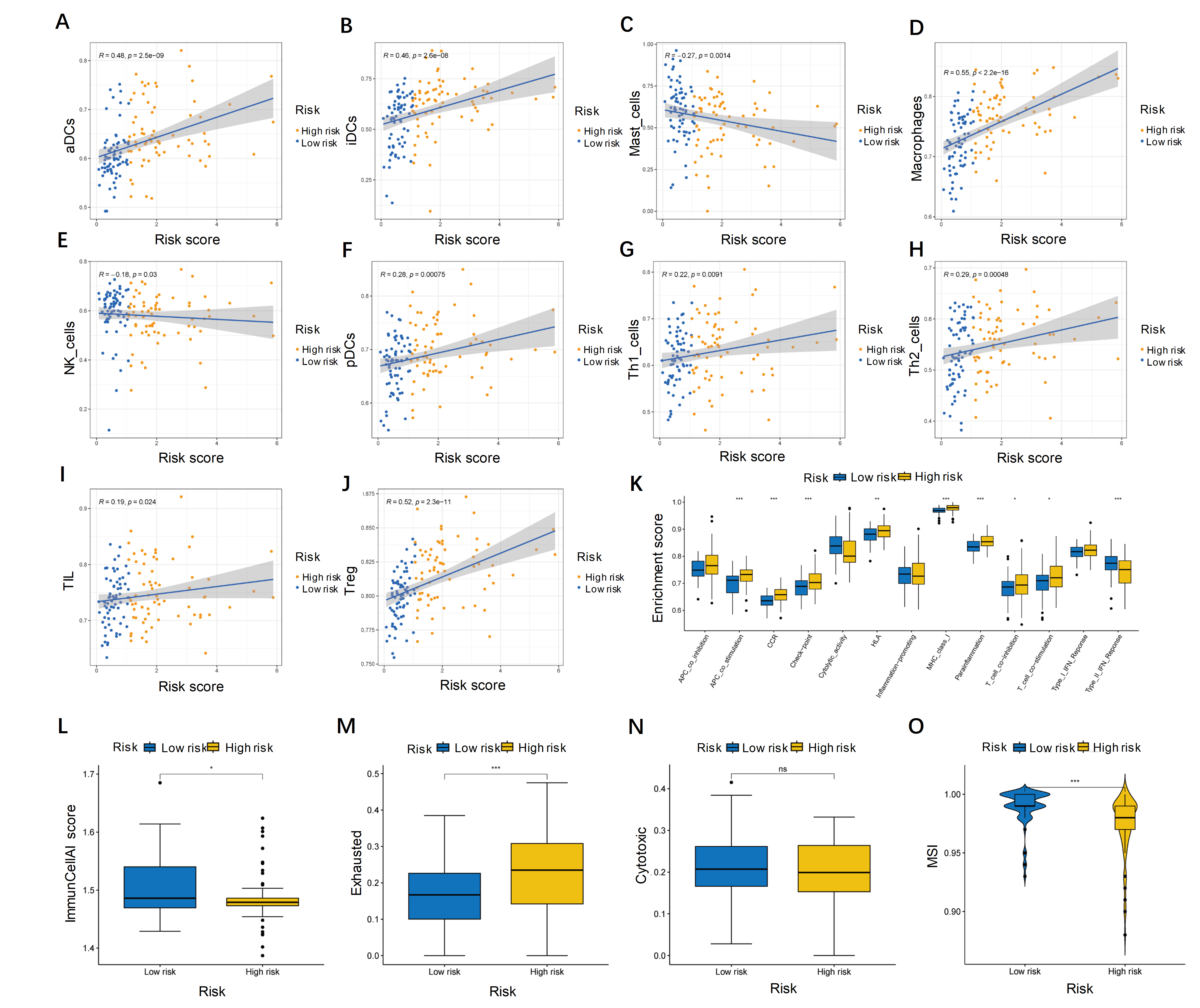

Supplement: Supplementary file 8 [file DataSheet_8.zip › figures/Figure7.tif]

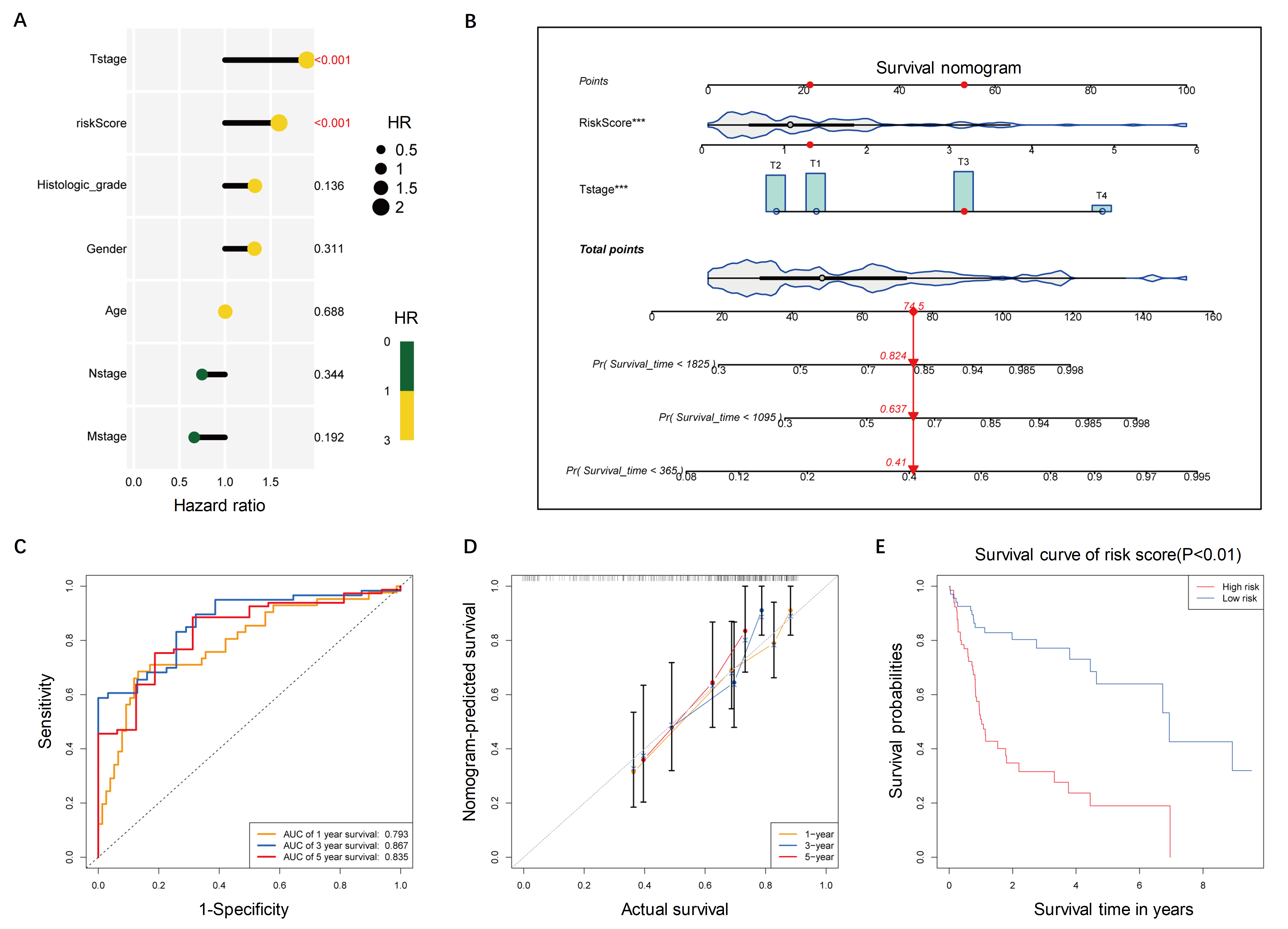

Supplement: Supplementary file 8 [file DataSheet_8.zip › figures/Figure8.tif]

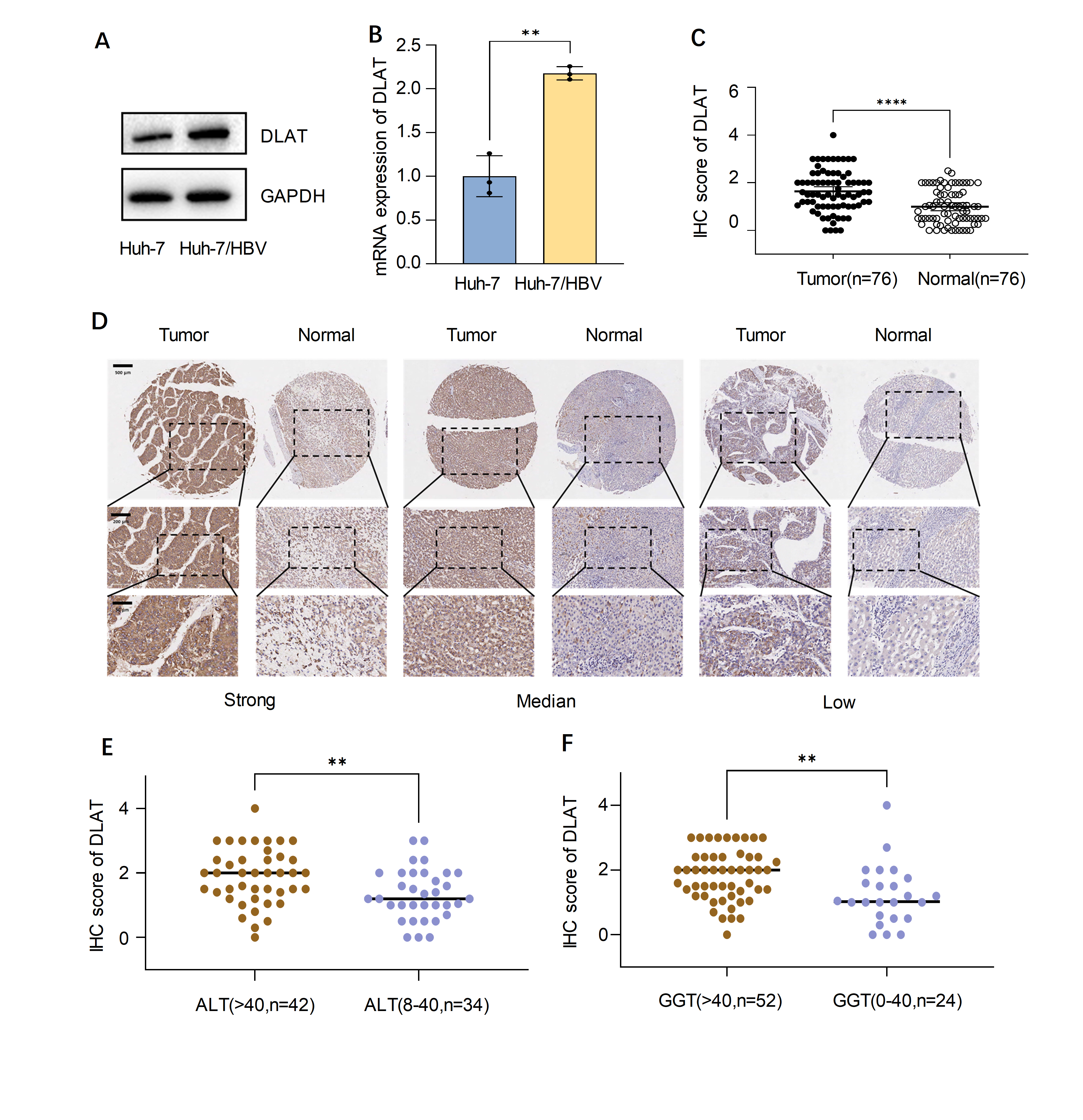

Supplement: Supplementary file 8 [file DataSheet_8.zip › figures/Figure9.tif]

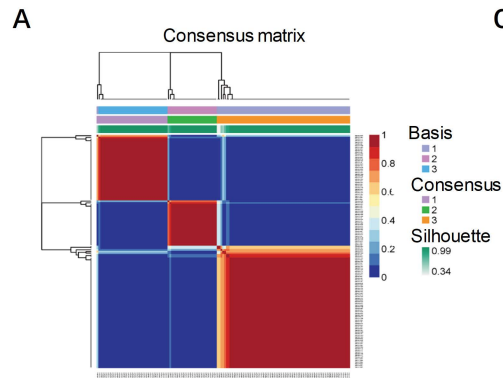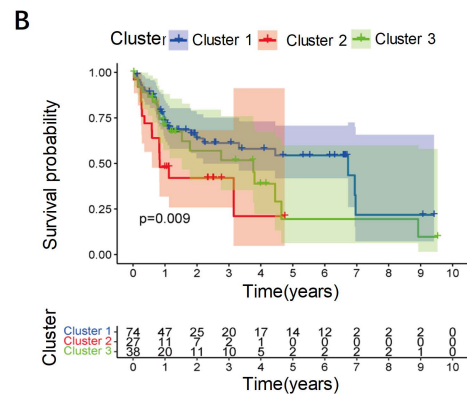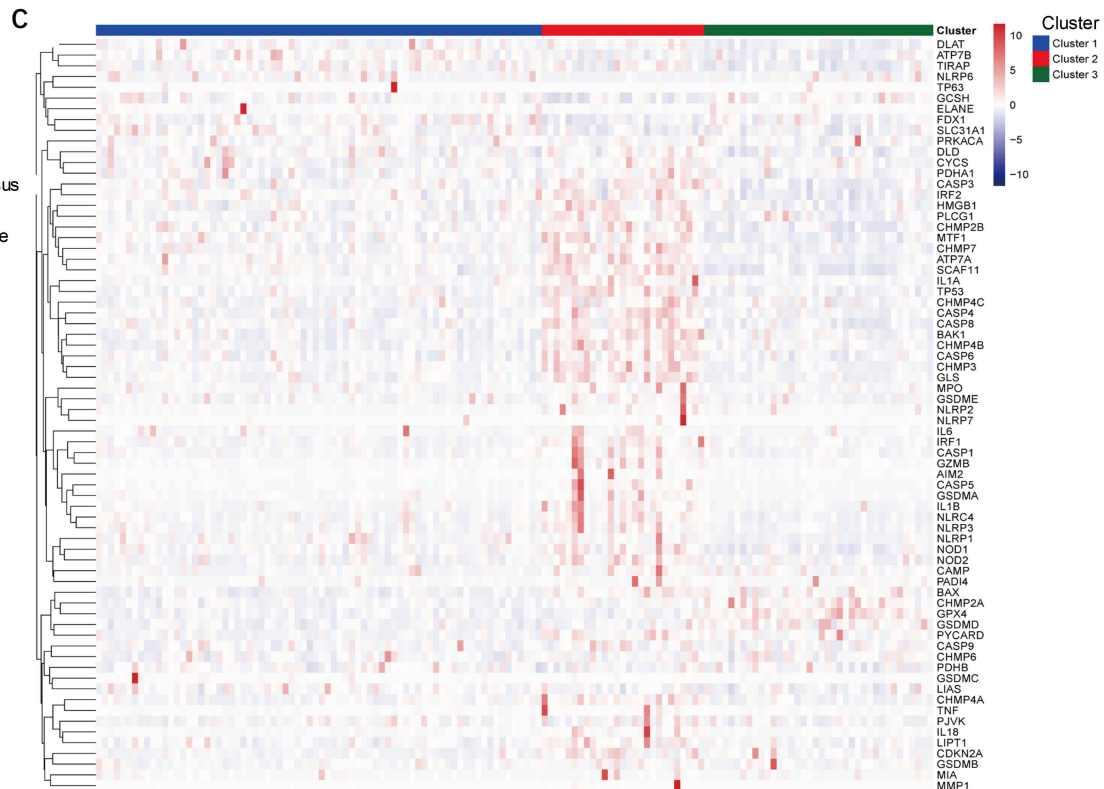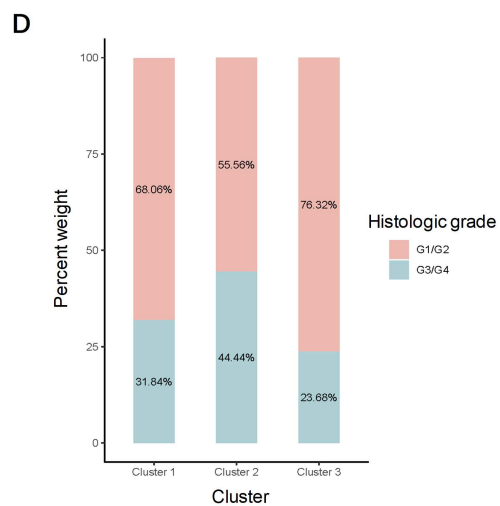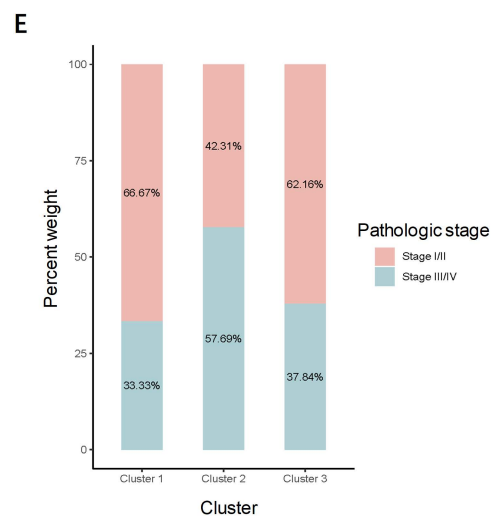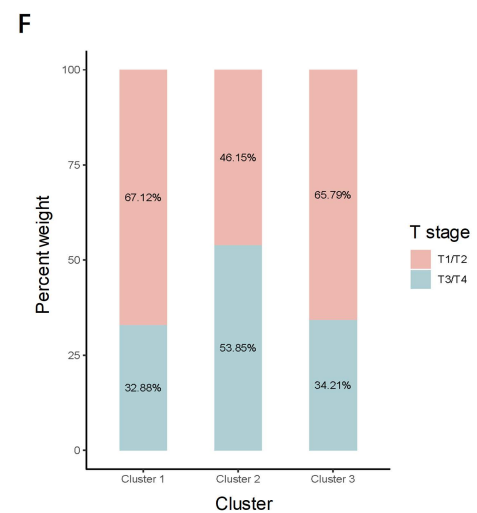

**A**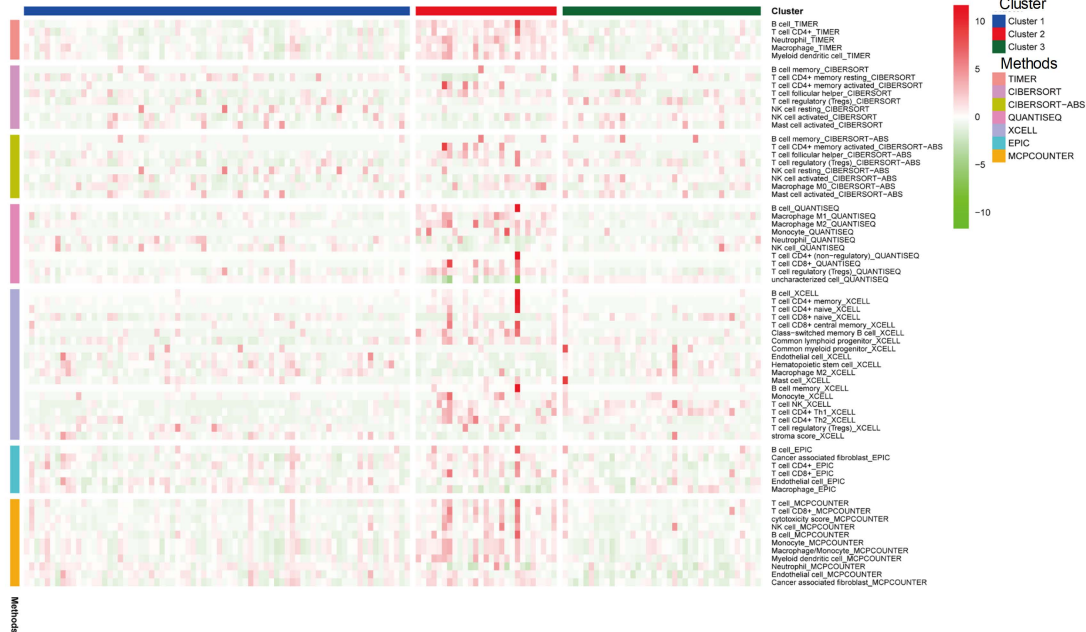**B**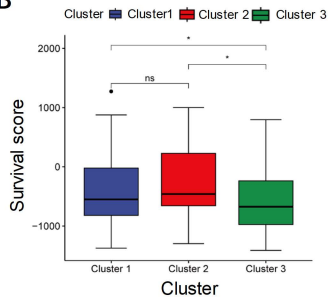**C**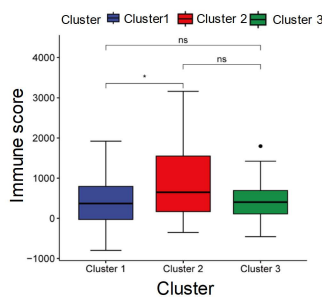**D**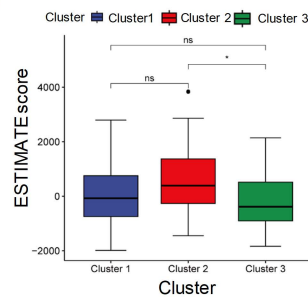**E**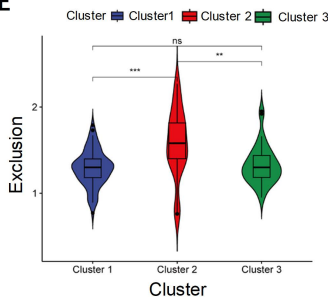**F**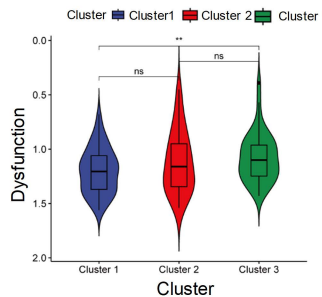**G**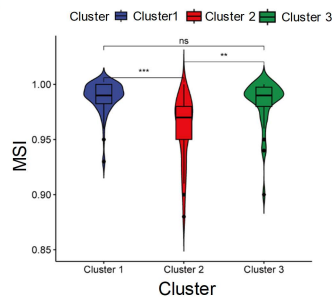

**A**

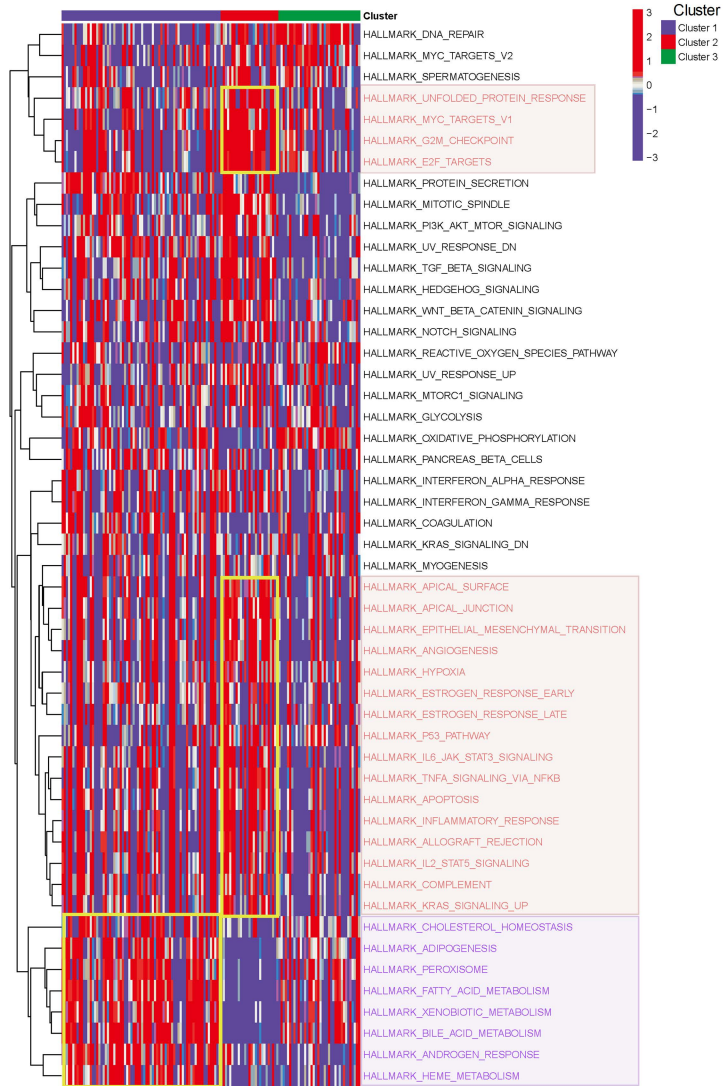

**B**

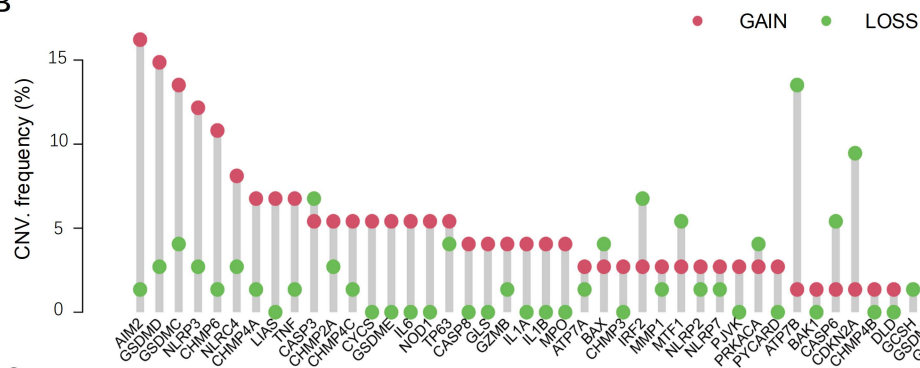

C

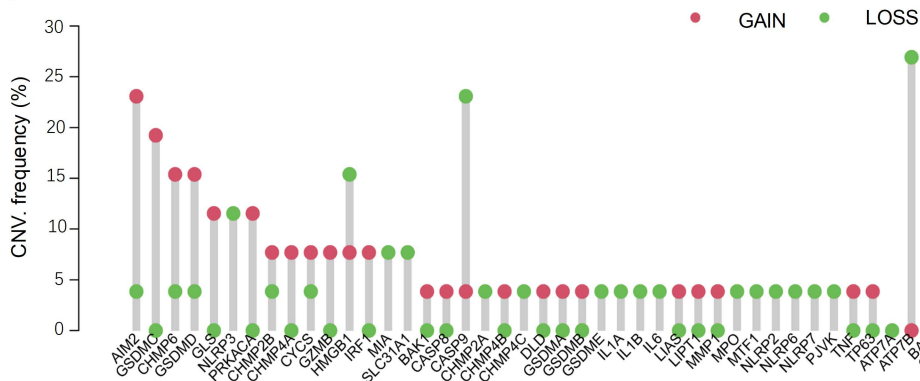

D

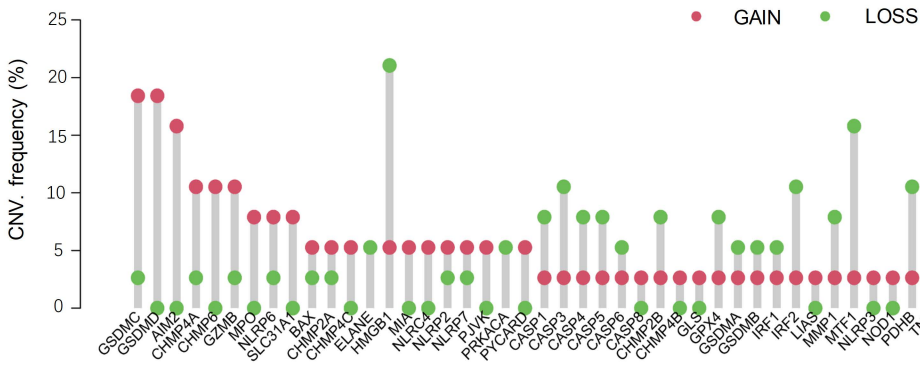

**A**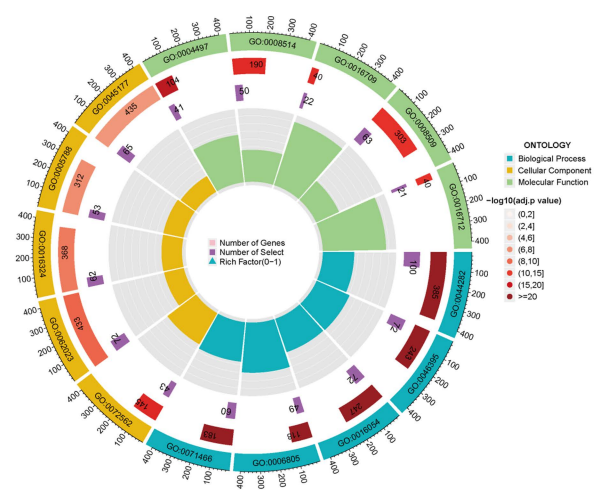**B**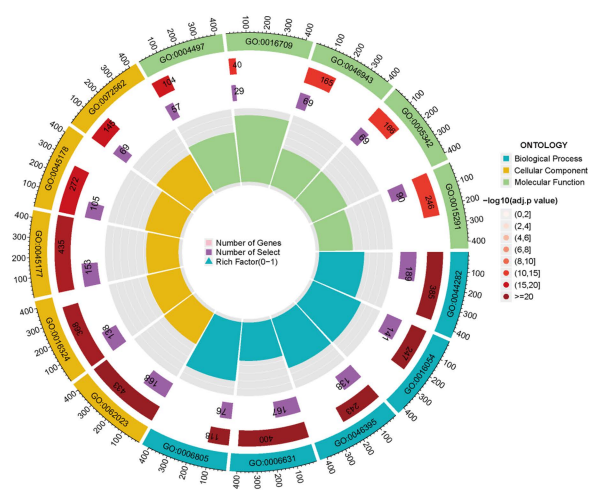**C**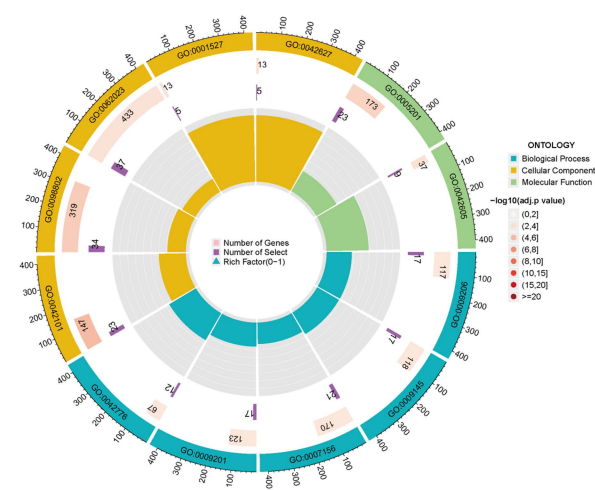**D**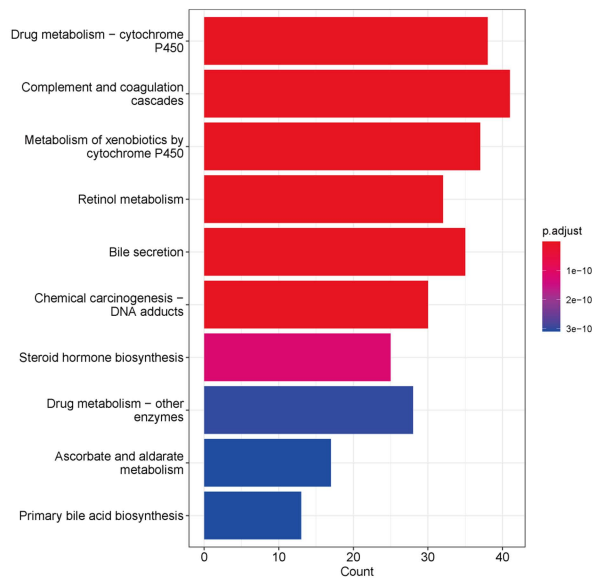**E**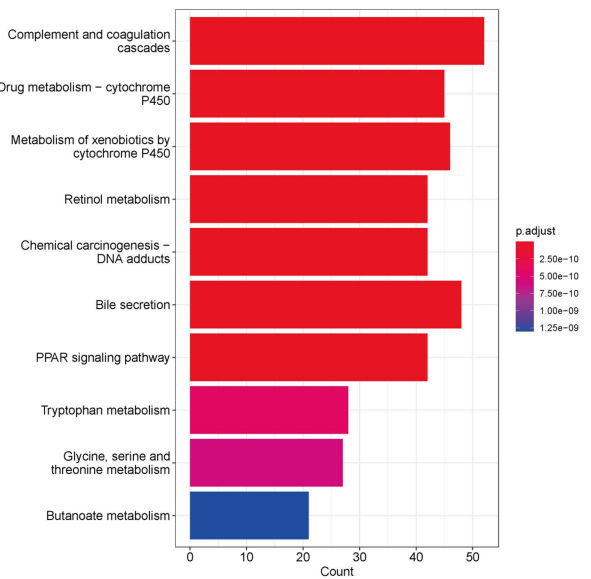**F**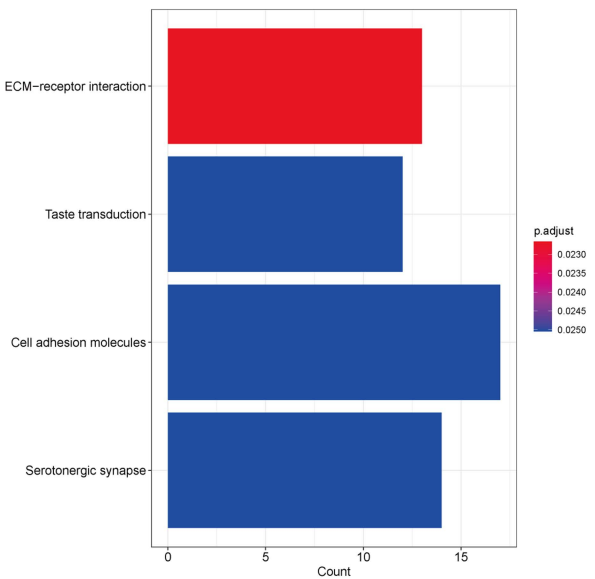

**A**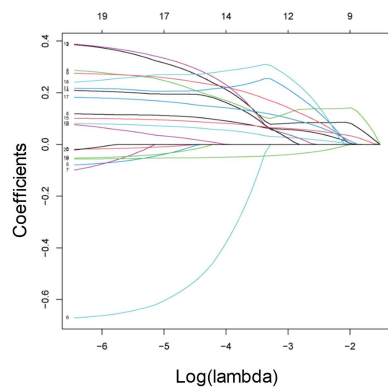**B**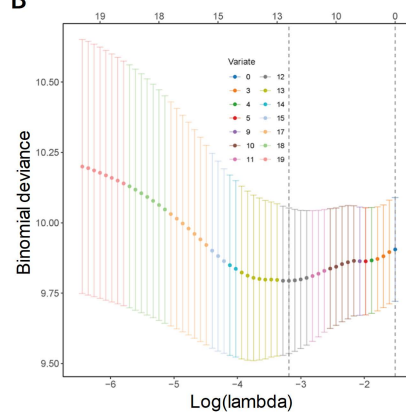**C**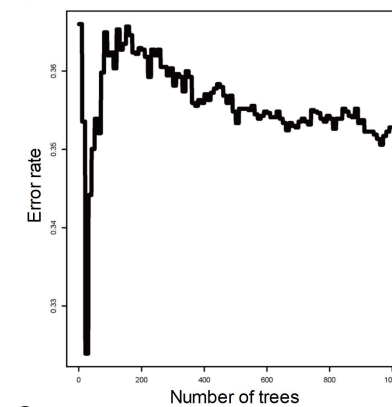**D**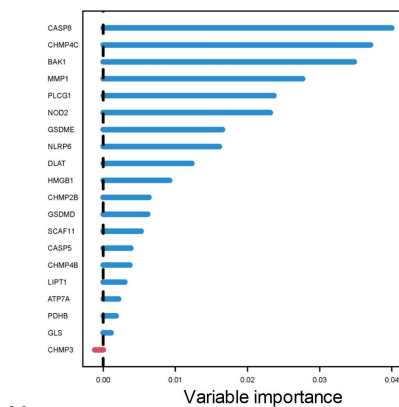**E**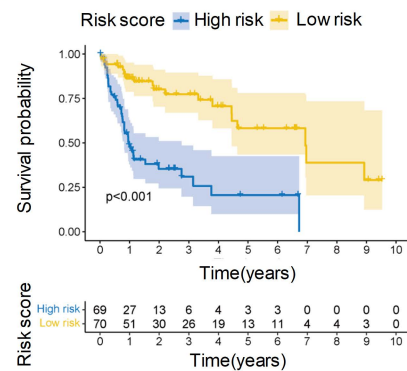**F**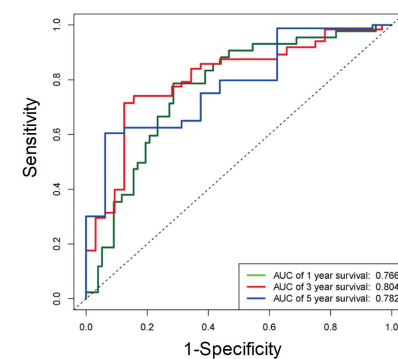**G**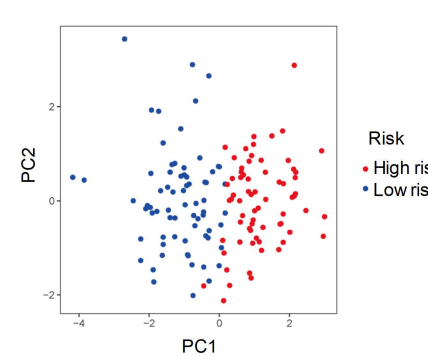**H**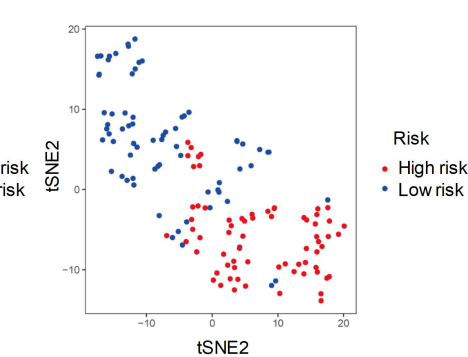**I**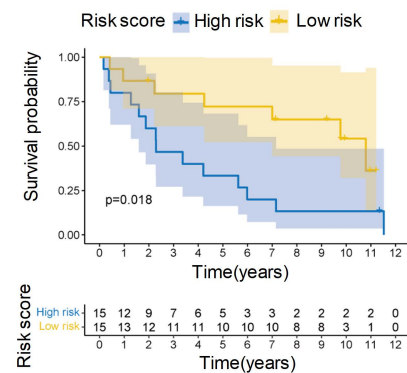**J**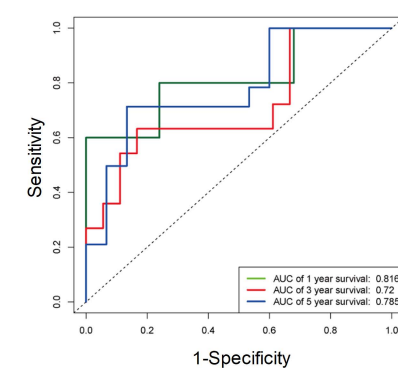**K**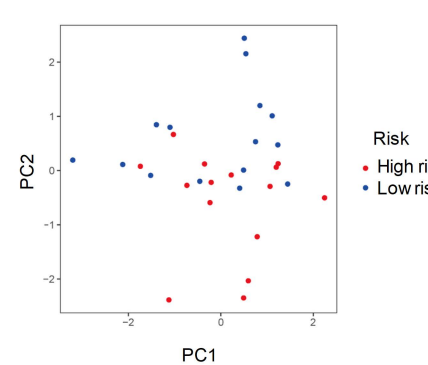**L**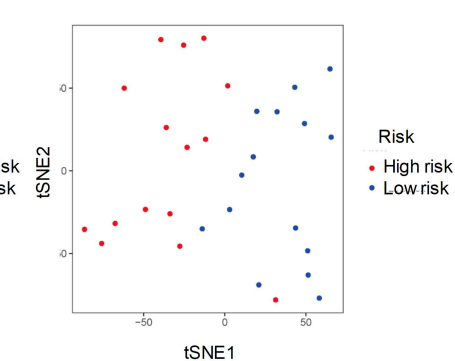

**A**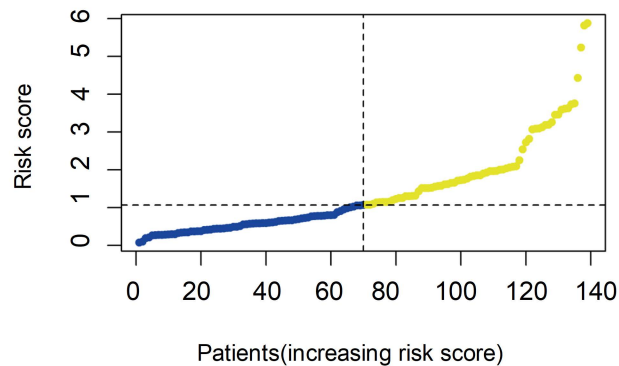**B**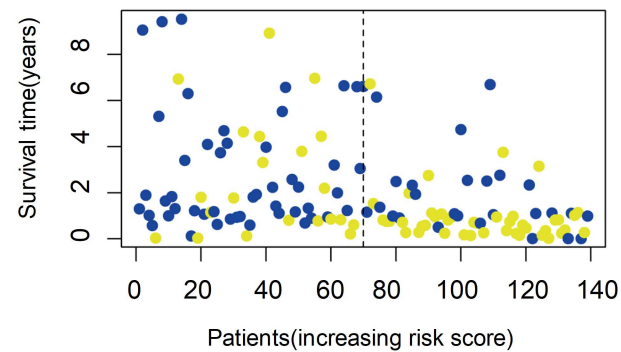**C**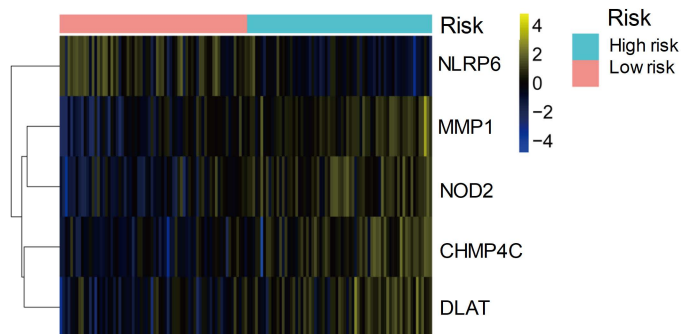**D**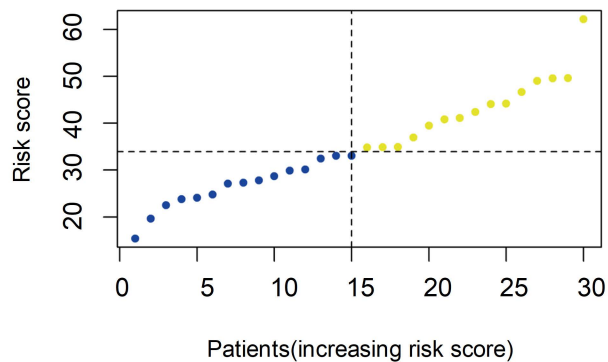**E**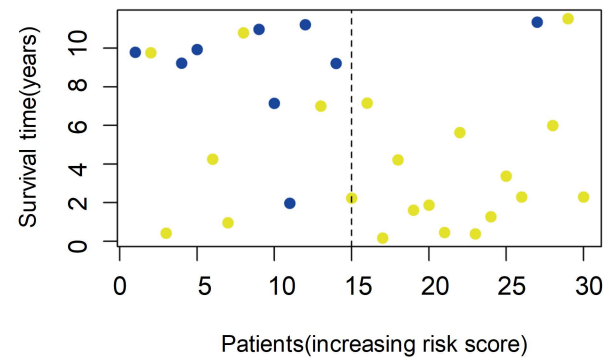**F**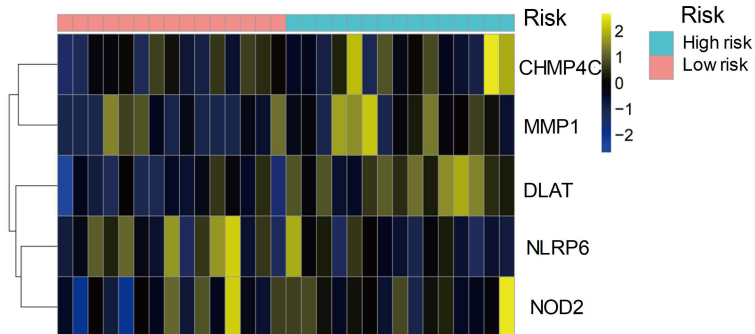

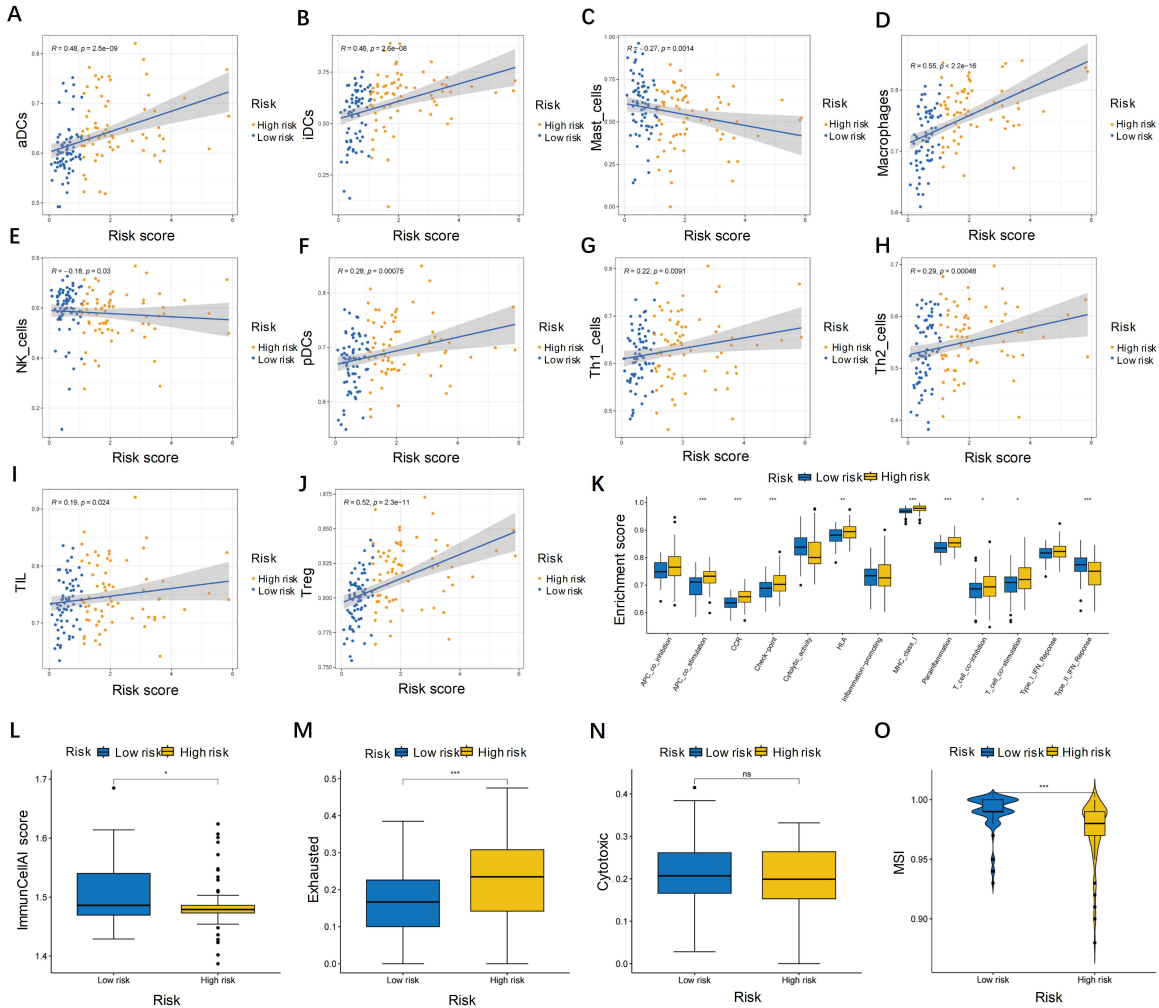

A

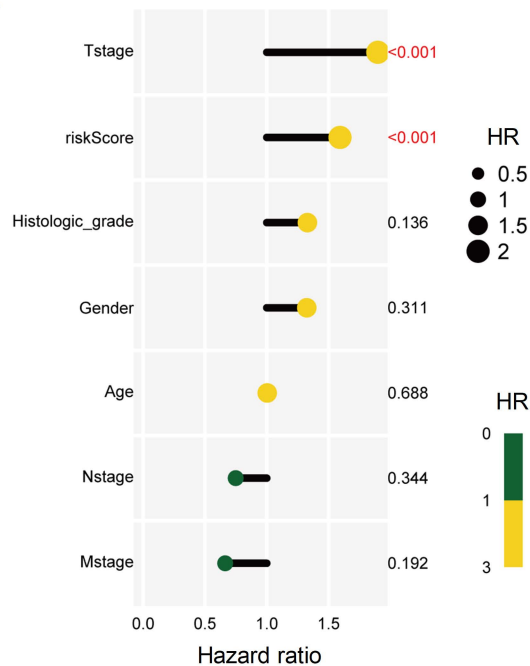

B

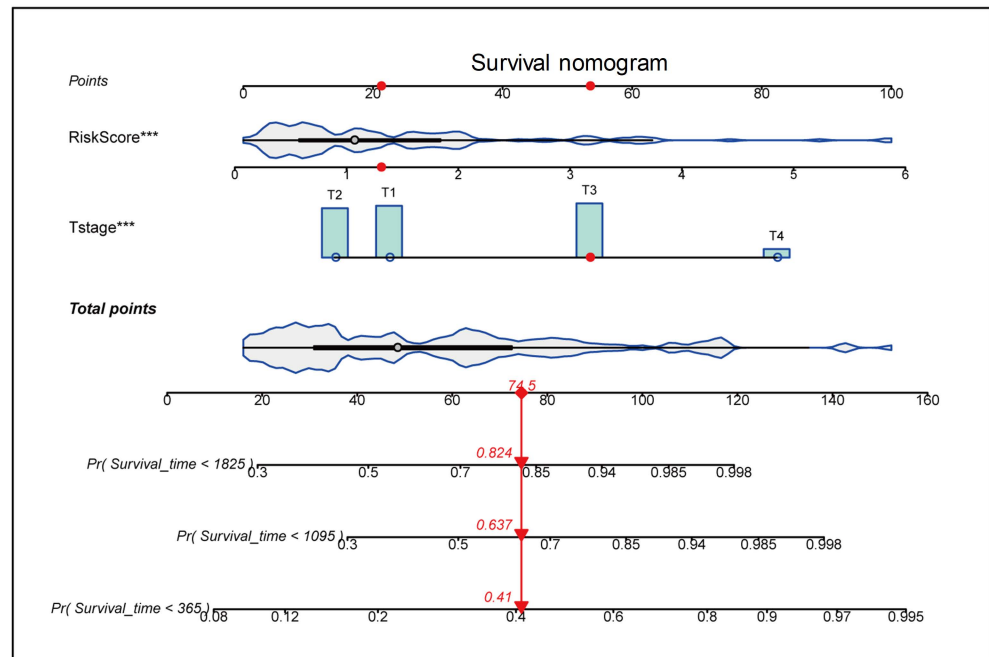

C

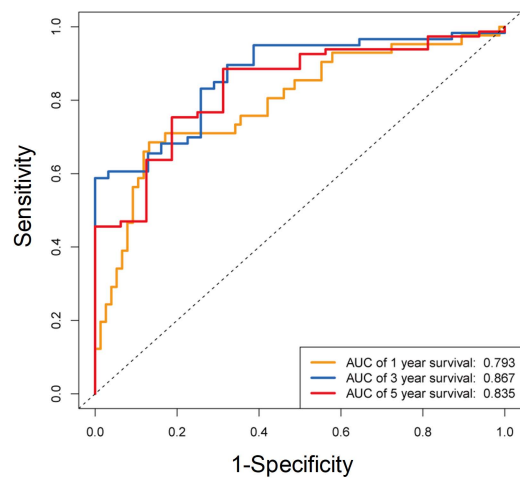

D

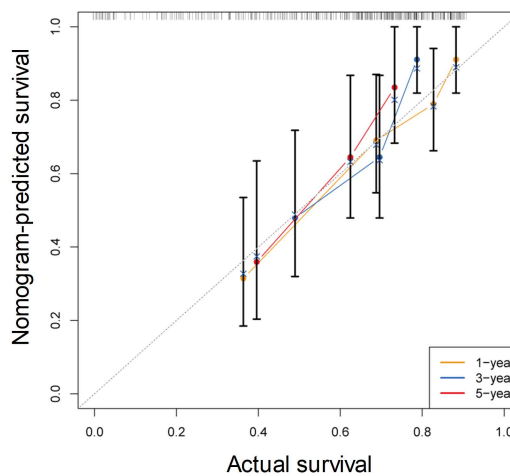

E

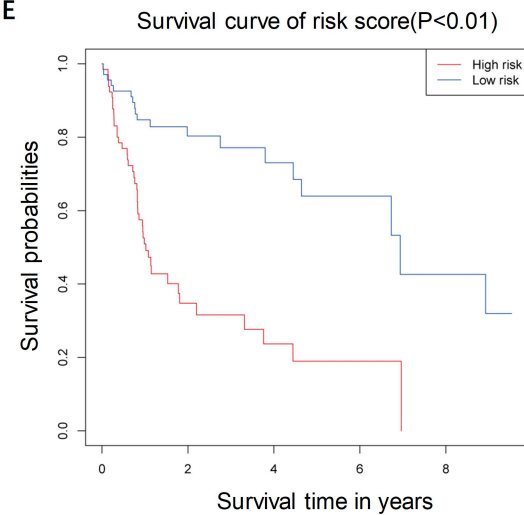

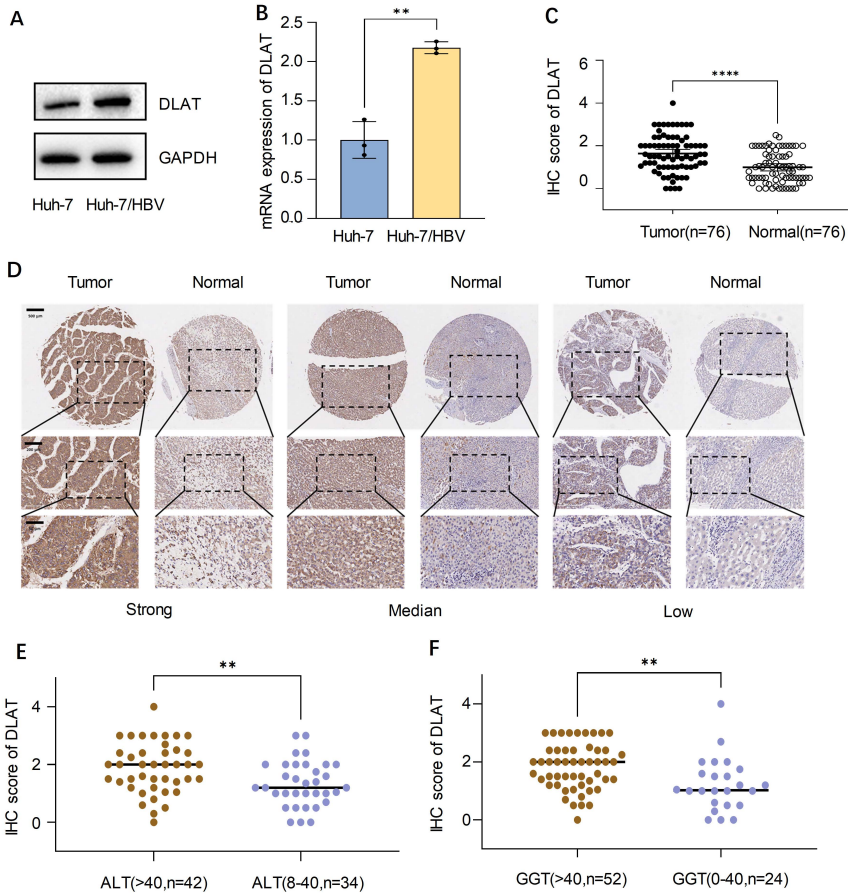

Supplement: Supplementary file 8 [file DataSheet_8.zip › figures/Figures.pdf]
