# Supplementary material for: Hepatitis B-related hepatocellular carcinoma: classification and prognostic model based on programmed cell death genes
Source: Front Immunol. 2024 May 10;15:1411161. doi: 10.3389/fimmu.2024.1411161 (PMC11116790; doi:10.3389/fimmu.2024.1411161)
Supplement: Supplementary file 9 [file DataSheet_9.zip › supplement materials/supplement image.docx]

**Supplement material**


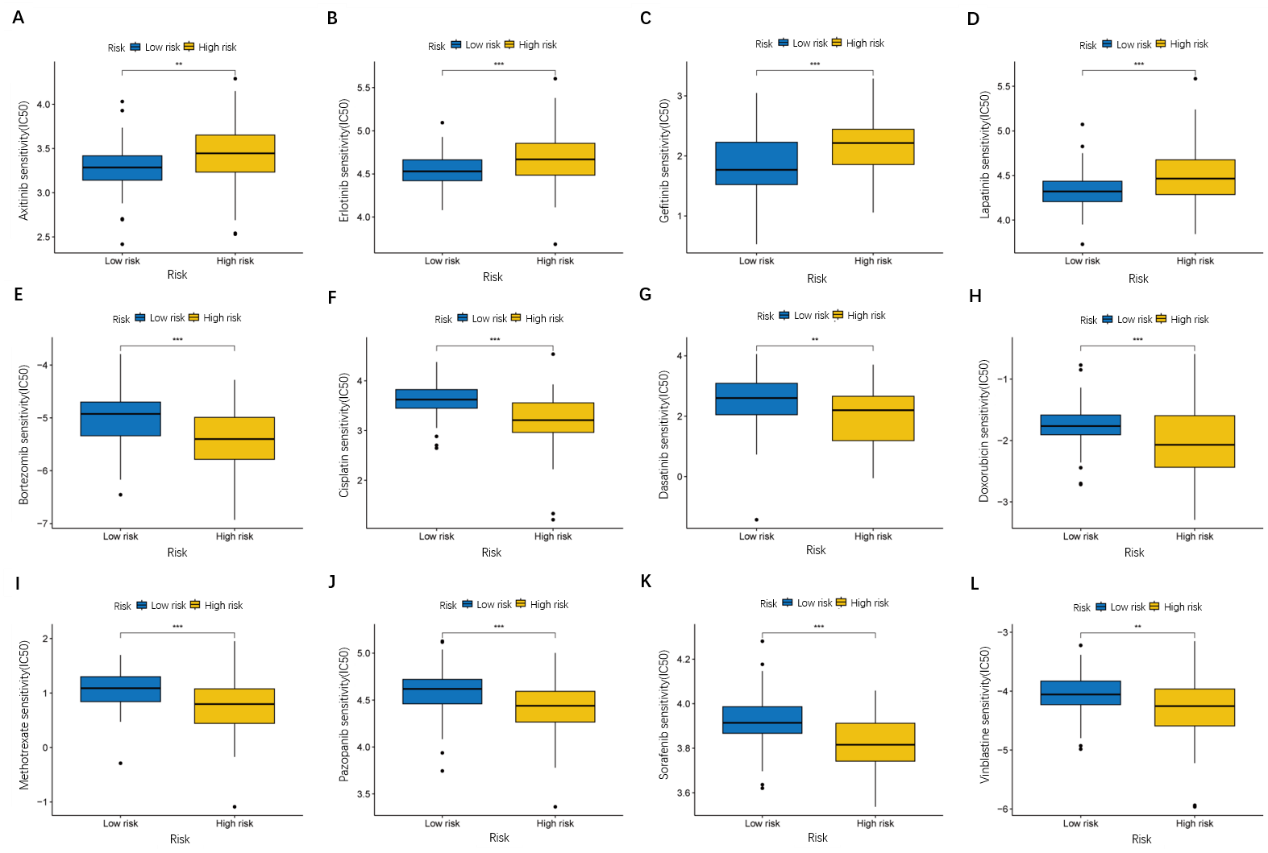


**Supplementary Fig.1 Drug sensitivity analysis of the high- and low-risk groups in HBV-HCC patients.** The box plots represent IC50 values of 12 drugs in high- and low-risk HBV-HCC groups. The IC50 values of 4 drugs were significantly lower in the low-risk group, while the IC50 values of 8 drugs were significantly lower in the high-risk group.
